# Novel C-2 Aromatic Heterocycle-Substituted Triterpenoids Inhibit Hedgehog Signaling in GLI1 Overexpression Cancer Cells

**DOI:** 10.1021/acsomega.4c11479

**Published:** 2025-03-04

**Authors:** Ivo Frydrych, Barbora Choma, Lucie Slavíková, Jan Pokorný, Nikola Jakubcová, Sandra Ludha, Soňa Gurská, Jiří Řehulka, Barbora Lišková, Petr Džubák, Marián Hajdúch, Milan Urban

**Affiliations:** † Institute of Molecular and Translational Medicine, Faculty of Medicine and Dentistry, Palacký University Olomouc and University Hospital Olomouc, Hněvotínská 1333/5, Olomouc 779 00, Czech Republic; ‡ Department of Organic Chemistry, Faculty of Science, Palacký University Olomouc, 17. listopadu 1192/12, Olomouc 771 46, Czech Republic; § Laboratory of Medicinal and Organic Chemistry, Institute of Molecular and Translational Medicine, Faculty of Medicine and Dentistry, Palacký University Olomouc, Hněvotínská 1333/5, Olomouc 779 00, Czech Republic; ∥ Laboratory of Experimental Medicine, Institute of Molecular and Translational Medicine, Czech Advanced Technology and Research Institute, Palacký University Olomouc, Šlechtitelů 241/27, Olomouc-Holice 783 71, Czech Republic

## Abstract

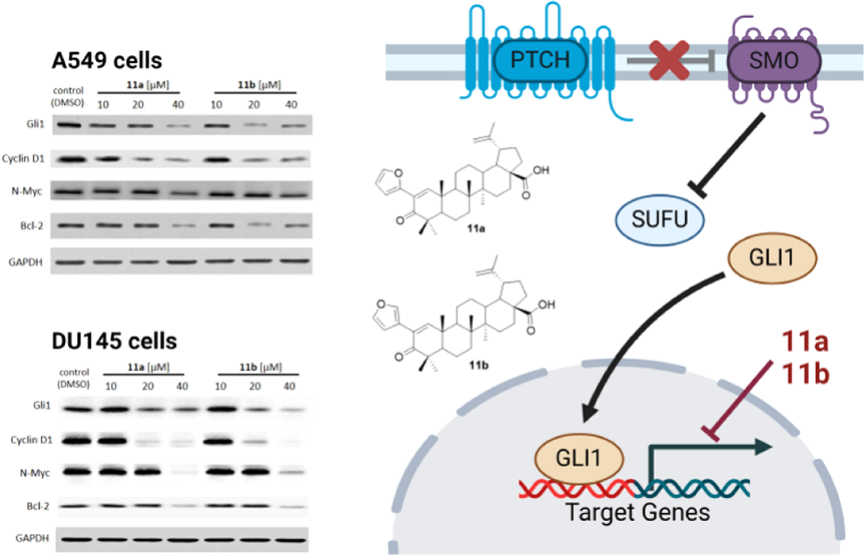

The hedgehog signaling pathway plays an important role in vertebrate embryonic development, tissue homeostasis, and tumorigenesis. Constitutive activation of Hh signaling in various human tumors leads to GLI-mediated transcription and tumor progression. Based on the preliminary screening of a large library of known triterpenes that exhibited interesting Hh inhibitory activity, we designed and synthesized a new series of triterpenoid analogues containing aromatic heterocyclic substituents at position C-2 to enhance their interference with Hh signaling. In this study, we evaluated the effect of 15 synthesized triterpenoids on cell proliferation and Hh pathway activity in relevant cancer cell lines. Among these compounds, two derivatives, **11a** and **11b**, both featuring a furan ring at position C-2, demonstrated potent inhibitory effects on proliferation and induced cell death in nonsmall cell lung cancer (NSCLC) and prostate cancer cell lines exhibiting hyper-activated Hh signaling. Moreover, these compounds significantly reduced GLI-mediated transcription in cell-based reporter assays. Detailed immunoblot analyses revealed that compounds **11a** and **11b** decreased the expression of endogenous GLI1 protein and its target genes associated with tumor progression and proliferation, such as Cyclin D1, N-Myc, and Bcl-2, in A549 and DU-145 cancer cells. These findings suggest that the antiproliferative effects of **11a** and **11b** are mediated through inhibition of the Hh signaling pathway and are promising candidates for the development of new anticancer therapies targeting Hh-dependent tumors.

## Introduction

The hedgehog (Hh) signaling pathway is essential for embryonic development and plays a vital role in tissue homeostasis and maintenance of somatic stem cells. Activation of the Hh pathway begins when the paracrine signaling factor Sonic Hedgehog (Shh) binds to the Patched 1 (Ptch1) receptor. In the absence of Shh, Ptch1 inhibits the activity of the seven-transmembrane protein Smoothened (Smo). However, once Shh binds to the Ptch1 receptor, this inhibition is relieved, allowing Smo to translocate to the primary cilium, a microtubule-based organelle essential for Hh signal transduction, where it undergoes activation processes.^1^ Activated Smo then regulates the Gli family of transcription factors (Gli1, Gli2, and Gli3). In contrast to the transcriptional activator Gli1, Gli2 and Gli3 can function as both activators and repressors, with Gli3 predominantly serving as a repressor. The activity of Gli proteins is further modulated by Suppressor of Fused (Sufu), which can inhibit their translocation to the nucleus.^1,2^

Hh signaling controls the expression of genes involved in cell cycle regulation, such as Cyclin D, E, and Myc, as well as developmental regulators such as Fgf4 and Hhip. Moreover, target genes Ptch1/2 and Gli1 provide a regulatory feedback loop mechanism.^2^ It has been shown that deregulation of the Hh pathway is associated with carcinogenesis, driving tumorigenesis and malignancy.^3^ Approximately 25% of human tumors require Hh signaling to maintain proliferative activity.^4^ Cancers such as basal cell carcinoma and medulloblastoma are associated with constitutive pathway activation or mutations in key genes like Ptch1, Smo, or Sufu.^5^ Other tumors, including nonsmall cell lung cancer (NSCLC) and prostate cancer, show hyperactivated Hh signaling, leading to aggressive growth and reduced sensitivity to known Hh antagonists due to overexpression of Gli1.^6^ Current antagonists targeting the Hh pathway include Gli,^7^ Smo,^8^ and Hh acyltransferase inhibitors.^9^ Nevertheless, the development of resistance, particularly to Smo inhibitors, has limited their long-term efficacy in anticancer therapy.^8,10,11^

In addition to resistance, issues related to drug administration, pharmacokinetics, and the heterogeneous mechanisms of Hh pathway activation across cancer subtypes limit the clinical translation of existing inhibitors.^12,13^ Therefore, there is a need for novel Hh inhibitors that are effective in cancers with constitutive Hh activation and Gli1 overexpression.

Pentacyclic triterpenoids, a large class of natural compounds, exhibit a variety of biological activities, including selective anticancer effects through multiple mechanisms.^14−23^ Betulinic acid, one of the important triterpenes, was reported to have inhibitory activity against the Hh signaling pathway in rhabdomyosarcoma,^24^ which motivated us to perform this study, in which we describe the synthesis, efficacy evaluation, and study of the mechanism of action of novel 2-substituted triterpenoids in lung and prostate cancer cell lines with constitutive and ligand-independent Hedgehog pathway activation. This work follows our earlier discovery of Hh inhibition in a set of known triterpenoids from our laboratory; here, we describe new compounds whose structures and possible use in therapy were patented.^25^ The preliminary experiments revealed that the majority of the compounds that interfere with the Hh pathway contained a substituent in position C-2 of the triterpenoid skeleton, and therefore here we designed a small library of 2-substituted lupane and 18α-oleanane derivatives. Various heterocycles were used to modify the position C-2 since heterocyclic analogues of pentacyclic triterpenoids have shown high selective cytotoxicity in cancer cells in many literature precedents.^26−28^

## Results and Discussion

### Chemistry

**Scheme 1:**
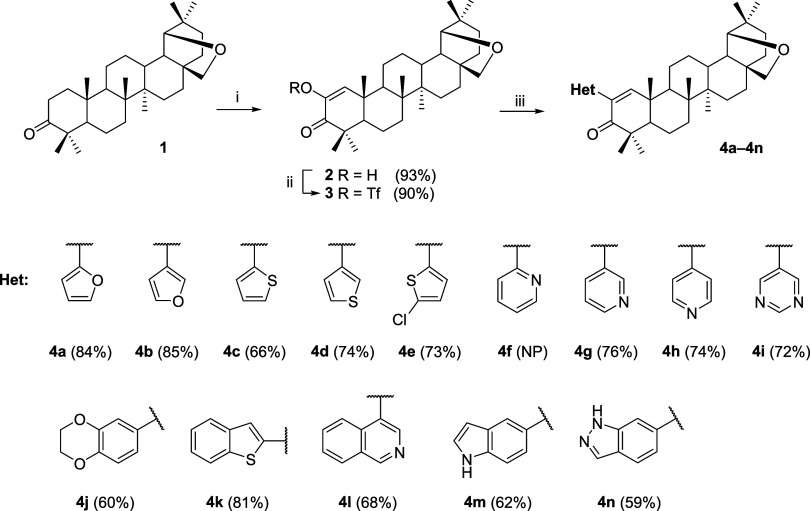
Synthesis of Heterocyclic Derivatives **4a**–**4n** Reagents and conditions: (i) *t*-BuOK, *t*-BuOH, O_2_ (air), 40 °C, 4–8 h; (ii) Tf_2_NPh, DMAP, TEA, DCM, r.t., 90 min; (iii) boronic acid **a**–**n**, K_2_CO_3_, PdCl_2_(PPh_3_)_2_, 1,4-dioxane*i*-PrOH/H_2_O 2:2:1, 80 °C, 2 h–overnight. NP = Not prepared.

**Scheme 2:**
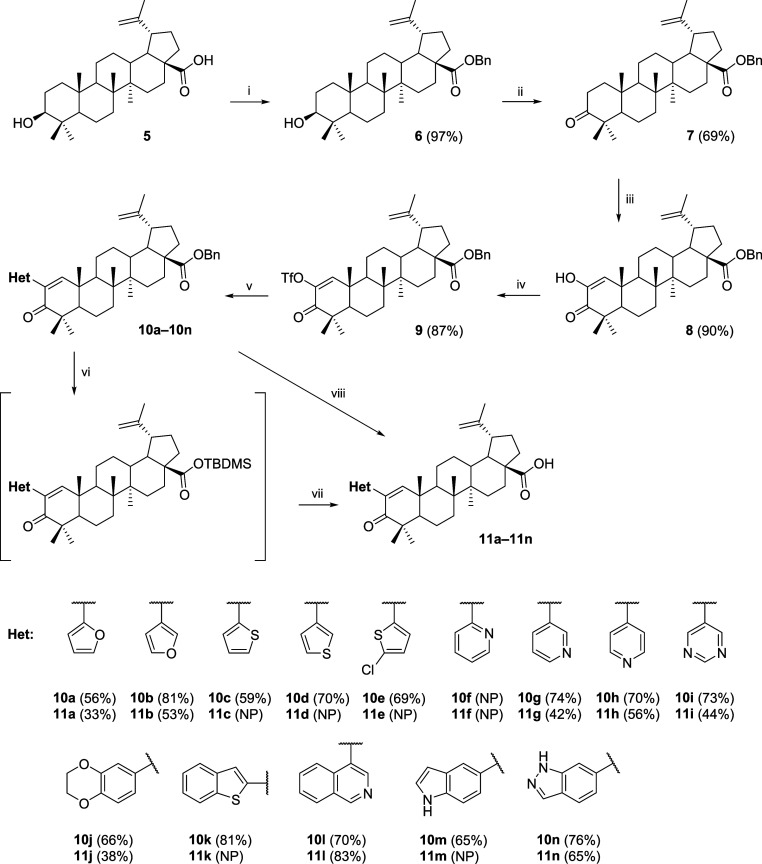
Preparation of Protected Heterocyclic Derivatives **10a**–**10n** and Nonprotected Analogs **11a**–**11n** Reagents and conditions: (i) BnBr, K_2_CO_3_, DMF, acetonitrile, 60 °C; (ii) Na_2_Cr_2_O_7_·2H_2_O, CH_3_COONa·3H_2_O, AcOH, (CH_3_CO)_2_O, 1,4-dioxane, r.t.; (iii) *t*-BuOK, *t*-BuOH, O_2_ (air), 40 °C; (iv) Tf_2_NPh, DMAP, TEA, DCM, r.t.; (v) boronic acid **a**–**n**, K_2_CO_3_, PdCl_2_(PPh_3_)_2_, 1,4-dioxane/*i*-PrOH/H_2_O 2:2:1, 80 °C, 2 h–overnight; (vi) TBDMSH, Pd(OAc)_2_, TEA, DCE, 60 °C, 4 h; (vii) TBAF in THF, 1,4-dioxane, r.t., 1 h; (viii) 1,3-cyclohexadiene, Pd/C, EtOH, 45 °C. NP = Not prepared.

Allobetulone **1** was used as a starting material for the first set of compounds since it usually does not undergo any side reactions, even under harsh conditions. All reactions were optimized at this stage (Scheme 1) and then used for the modification of betulonic acid **5** (Scheme 2). First, allobetulone **1** was oxidized to diosphenol **2** using a literature procedure.^29^ In the following step, the enol group was transformed into a corresponding triflate **3** using N-Phenyl-bis(trifluoromethanesulfonimide) under basic conditions. Triflate **3** was then subjected to a set of Suzuki-Miyaura cross-couplings with corresponding boronic acids under catalysis with 2% (molar) of bis(triphenylphosphine)palladium(II) dichloride in the presence of K_2_CO_3_ in a procedure modified from the literature.^30^ The reaction usually provided moderate to high yields of compounds **4a**–**4n** (59–85%) with one exception—we were not able to obtain *o*-pyridinyl derivative **4f**; all attempts for this product led to decomposition and a complicated mixture of compounds that we were not able to separate and characterize (Scheme 1). The same reaction scheme was used for betulinic acid **5** that had to be first protected as benzyl ester **6** and then converted to benzyl betulonate **7**. Following oxidation of the A-ring gave diosphenol **8**, which was then transformed into a triflate **9** and subjected to the analogous Suzuki-Miyaura cross-coupling reactions as previously with compound **3** (Scheme 2). The yields of 2-substituted compounds **10a**–**10n** were again between 56% and 81%. Once again, the *o*-pyridinyl derivative was not obtained due to the decomposition of the reaction mixture under all conditions tested. The crux of this pathway, however, was the deprotection of benzyl esters to free acids **11a**–**11n**. First attempts to deprotect compound **10a** using standard catalytic hydrogenation (H_2_ or 1,3-cyclohexadiene, Pd/C, various solvents) always yielded products with a partly hydrogenated double bond 1(2), and therefore an alternative procedure was used.^31^ In the first step, the benzyl ester moiety is replaced by a silyl ester. Then, the silyl ester is cleaved by TBAF to form a carboxylic acid. Despite that, compounds **11c**, **11d**, and **11e** were not obtained because they decomposed using this procedure. Curiously, compound **11n** was obtained only by catalytic hydrogenation.

### Biology

#### Cytotoxicity Assay

Based on the preliminary results and tests of a larger library of known triterpenoids, a small set of 2-substituted lupane and 18α-oleanane derivatives was designed and synthesized. As the first step, the cytotoxic activity of the synthesized derivatives was assessed using an MTS assay following 72 h of treatment (Table 1). Selected cell lines A549 (lung adenocarcinoma) and DU-145 (prostate cancer) are both dependent on Hh signaling. In addition, K562 (chronic myelogenous leukemia) was included as a cell line sensitive to Hh inhibitors^32^ and a type of hematological malignancy with active Hh signaling.^33^ MRC-5 lung fibroblasts and BJ human skin fibroblasts cell lines were used to evaluate the toxicity against nontumor cells. Compounds were also tested using the standard panel of other cancer cell lines, as described in our earlier work (Table 1).^27^ Analogues of allobetulone were inactive except for **4n,** with the indazole substituent displaying weak cytotoxicity against the tested cell lines, including normal human fibroblasts. Betulinic acid derivatives **11a**–**11n** exhibited moderate to high activity in the standard CCRF-CEM cell line; however, in this work, more attention was given to the activity in Hh-dependent cell lines. The best structures, **11a** – **11n,** showed cytotoxic activity in the A549 and K562 cancer cell lines. The IC_50_ values indicated that the presence of a free carboxyl moiety at C-28 of the structure is essential for cytotoxic activity, except for pyrimidine-substituted structures (like **11i**). As expected, the cytotoxicity of all benzylesters **10a**–**10n** was below the detection limit. Nonmalignant fibroblasts had sensitivity below the detection limit for compounds **11g**, **11h**, and **11l**. To conclude, in the MTS tests of cytotoxic activity, the most active compounds displayed comparable activity and selectivity as betulinic acid **5**; however, the following experiments revealed that, unlike in acid **5,** this activity is likely associated with activity against the Hh signaling pathway. Based on the lowest IC_50_ values of **11a** and **11b** against Hh-dependent cancer cell lines (A549, DU-145, and K562), these structures were selected for further evaluation of their activity against this signaling pathway.

**Table 1:** Cytotoxic Activities of Final Compounds on Nine Cancer and Two Normal Fibroblast Derived Cell Lines

	IC_50_ (μmo I/L)^a^											
Comp.	A549	DU-145	k562	K562-TAX	HCT 116	HCT116 p53–/–	U2OS	CCRF-CEM	CEM-DNR	BJ	MRC-5	SI^b^
**4g**	>50	43	>50	19	>50	>50	>50	25	16	>50	>50	>2.0
**4h**	>50	22	>50	18	>50	>50	>50	19	19	>50	>50	>2.7
**4i**	>50	>50	>50	>50	>50	>50	>50	>50	27	>50	>50	>1.0
**4l**	>50	32	>50	>50	>50	>50	>50	>50	>50	>50	>50	>1.0
**4m**	>50	26	>50	>50	>50	>50	>50	26	>50	>50	>50	>1.9
**4n**	30	24	23	16	31	31	25	18	24	42	33	2.1
**11a**	12	7.9	6.3	14	11	12	21	8.7	15	39	27	3.8
**11b**	12	5.7	9.6	16	20	14	26	6	22	37	24	5
**11g**	35	11	>50	20	34	40	44	18	21	>50	>50	>2.8
**11h**	21	11	>50	11	25	31	28	5.2	17	>50	>50	>9.5
**11i**	>50	18	>50	25	35	41	>50	19.7	42	>50	>50	>2.5
**11j**	14	7	7.5	17	22	21	22	4.9	24	>50	28	>7.9
**11l**	14	8.3	>50	8.7	19	31	25	7.2	17	>50	>50	>7.0
**11n**	15	8.3	22	12	15	17	21	4.9	22	35	27	6.3
Betulinic acid **5**	9.4	9.6	4.3	14	16	16	21	8.1	14	24	28	3.2
**GANT-61**	NA	>50	NA	NA	NA	NA	NA	NA	NA	NA	NA	NA

a The concentration of drug needed to inhibit cell growth by 50%. The standard deviation in cytotoxicity assays is typically up to 15% of the average value.

b Selectivity index is calculated for IC_50_ of CCRF-CEM line vs average of both fibroblasts (BJ and MRC-5). All other compounds prepared in this work were also tested, but their activities on these 11 cell lines were higher than 50 μM (Full table is in the Supplementary File).

#### Effect of **11a** and **11b** on Gli Transcriptional Activity

**Figure 1:**
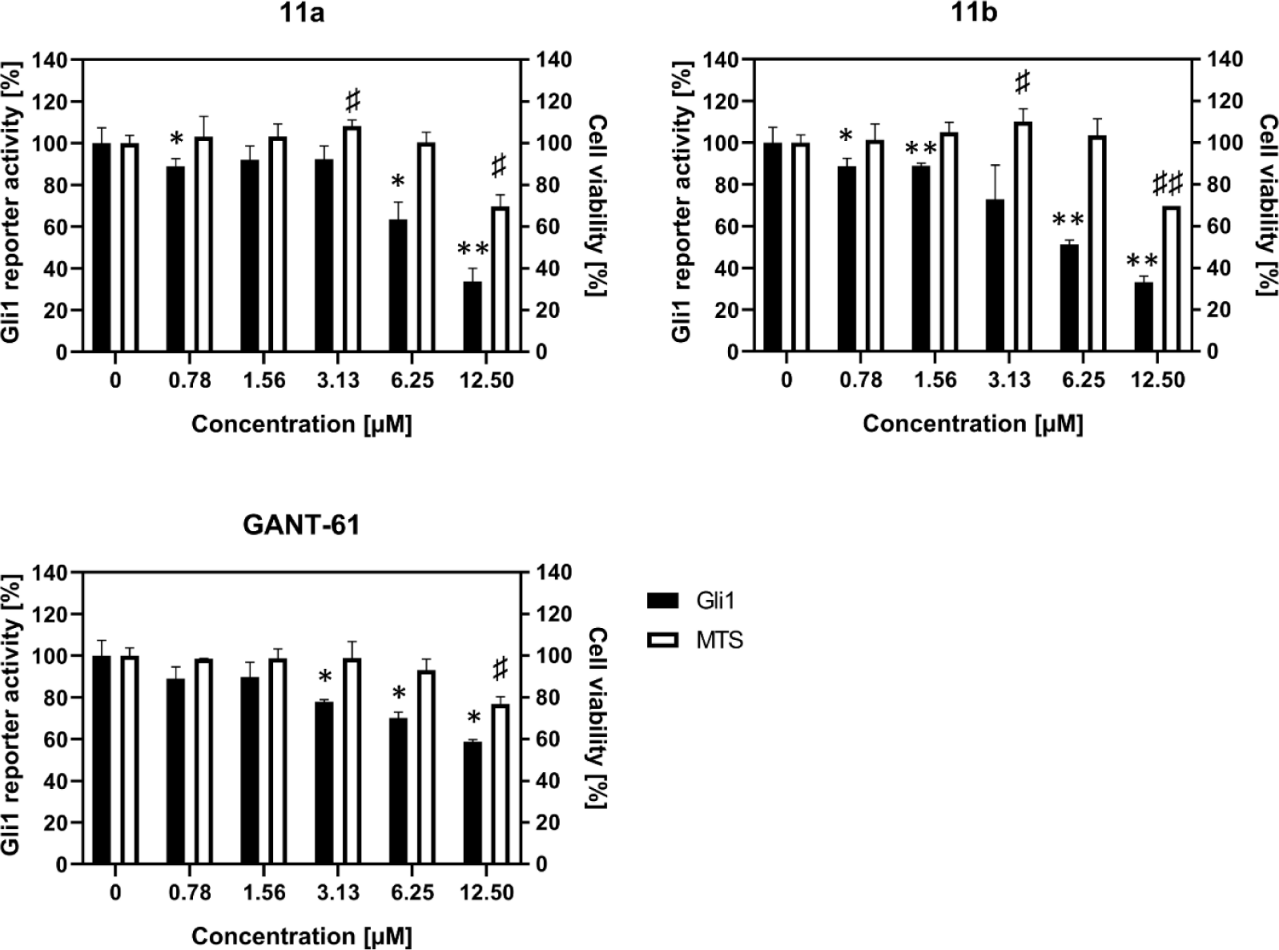
Effect of compounds **11a**, **11b,** and positive control GANT-61 on Gli1 activation and cell viability measured using the U-87 MG-Gli-FLuc reporter assay. Data are expressed as percentages of control (100%) and represent the mean of three independent experiments with the standard deviation. To distinguish specific inhibition of Gli activation from a nonspecific cellular response, an MTS assay was performed simultaneously under the same conditions in 96-well transparent plates. Statistical analysis of Gli1 activity was performed using one-sample Student’s *t*-test (mu = 100): *, *p* < 0.05; **, *p* < 0.01. The same analysis was used to assess cytotoxicity by MTS assay: #, *p* < 0.05; ##, *p* < 0.01.

To test the inhibitory activity of the most active compounds, **11a** and **11b,** on Gli-mediated transcription, we used a U-87MG-Gli firefly luciferase-based reporter derived from the human glioblastoma cell line. Compounds **11a** and **11b** inhibited Gli transcriptional activity in a dose-response manner and to a greater extent than the known Gli inhibitor GANT-61 at equimolar concentration (Figure 1). Importantly, the reduction of reporter activity by both compounds up to a dose of 6.25 μmol/L was not accompanied by inhibition of proliferation or cell death induction. Thus, specific inhibition of Gli-mediated transcription by compounds **11a** and **11b** clearly precedes inhibition of proliferation or induction of cell death. A possible direct inhibition of the reporter (Fluc) and luminescence itself, which could lead to false positive results, was also ruled out in a cellular as well as a noncellular model (data not shown).

To further validate the effect of new triterpenes on Hh signaling, we used the commercial reporter cell line NIH3T3–Gli reporter, which expresses the firefly luciferase (FLuc) gene under the control of a Gli-responsive element. Table 2 shows the IC_50_ values for the inhibition of mSHH or SAG-induced reporter activity by compounds **11a** and **11b**. The results proved that both compounds are potent inhibitors of Hh signaling. Only nontoxic concentrations of compounds **11a** and **11b** were used for the IC_50_ calculation.

**Table 2:** Inhibitory Effect of Compounds **11a** and **11b** on Endogenous Hh Signalingabcd

	mSHH stimulation		SAG stimulation	
Comp.	0.25 (μg/mL)	0.5 (μg/mL)	2 nM	4 nM
**11a**	3.5 ± 0.5	1.9 ± 0.2	4.5 ± 0.6	3.8 ± 0.4
**11b**	3.2 ± 0.3	2.4 ± 0.3	3.5 ± 0.2	4.1 ± 0.4

a IC_50_ values (μmol/L) represent the mean value ± SD from three independent experiments.

b The mSHH ligand at concentration of 0.25 and 0.5 μg/mL and SAG (SMO agonist) at a concentration of 2 and 4 nmol/L were added to the wells following 2 h preincubation with compounds at a concentration range 16–0.25 μmol/L.

c IC_50_ values were calculated from the differences in the measured luminescence values of mSHH/SAG stimulated and unstimulated cells in the presence of compounds **11a** and **11b**.

d The difference in luminescence values of mSHH/SAG stimulated and unstimulated cells in the absence of compounds **11a** and **11b** was set to 100%.

**Figure 2:**
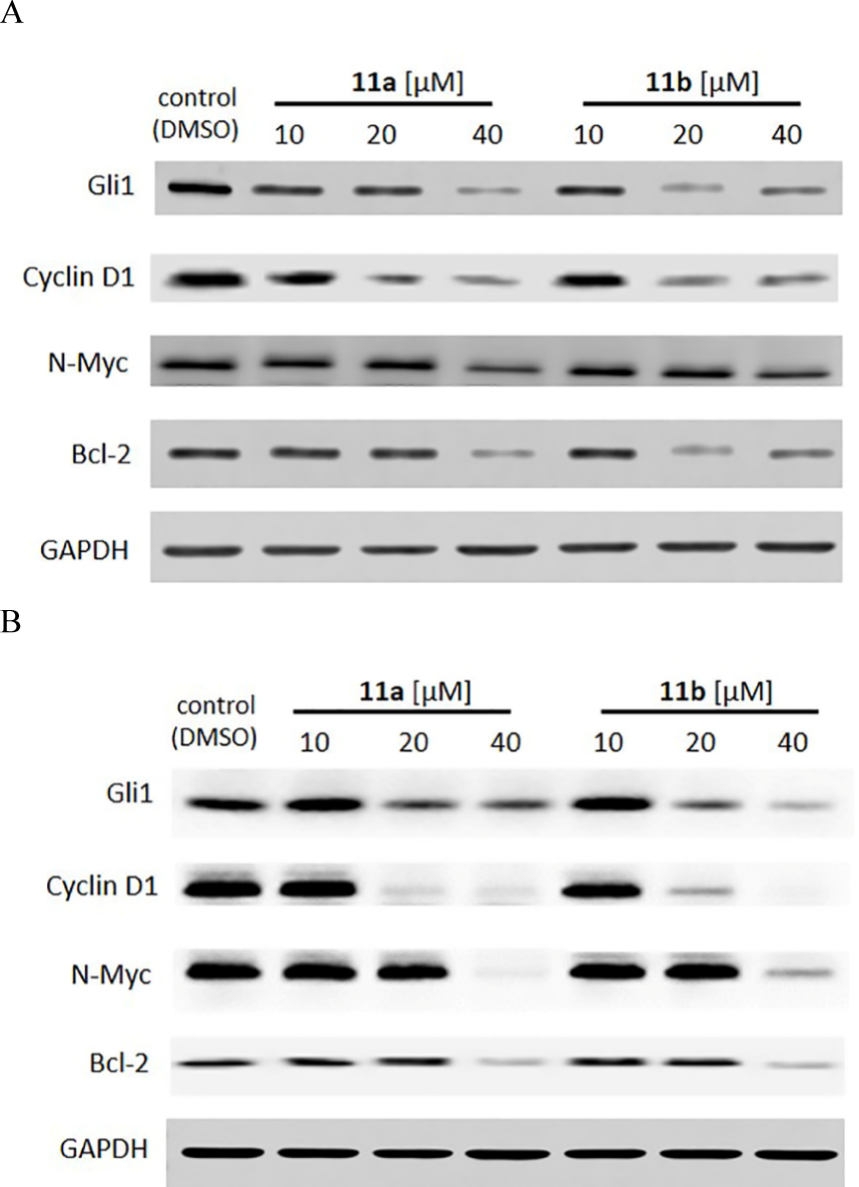
Effect of compounds **11a** and **11b** on the protein expression of the Gli1 transcription factor and its transcriptional targets in A549 (A) and DU145 (B) cells following 24 h of incubation. An antibody against GAPDH was used as an internal loading control.

In order to verify that compounds **11a** and **11b** inhibit the proliferation of A549 and DU-145 through Hh inhibition, we monitored the expression of Hh target genes in these cell lines (Figure 2). Compounds **11a** and **11b** inhibit the endogenous Gli1 protein level in a dose-dependent manner in both cell lines. The decreased protein expression of Gli1 targets Cyclin D1, N-Myc, and Bcl-2 strongly indicates inhibition of its transcriptional activity, and their downregulation correlates with the data obtained by the U-87 MG-Gli-FLuc reporter assay. Importantly, these proteins are involved in cell cycle progression, proliferation, and survival. N-Myc amplification or deregulation of transcription, stability, and degradation is associated with many malignancies,^34^ and the observed downregulation induced by **11a** and **11b** may reduce its oncogenic effect. According to previous research, compounds **11a** and **11b** displayed comparable efficacy to betulinic acid, which was used at a concentration of 21.8 μmol/L in rhabdomyosarcoma.^24^

**Figure 3:**
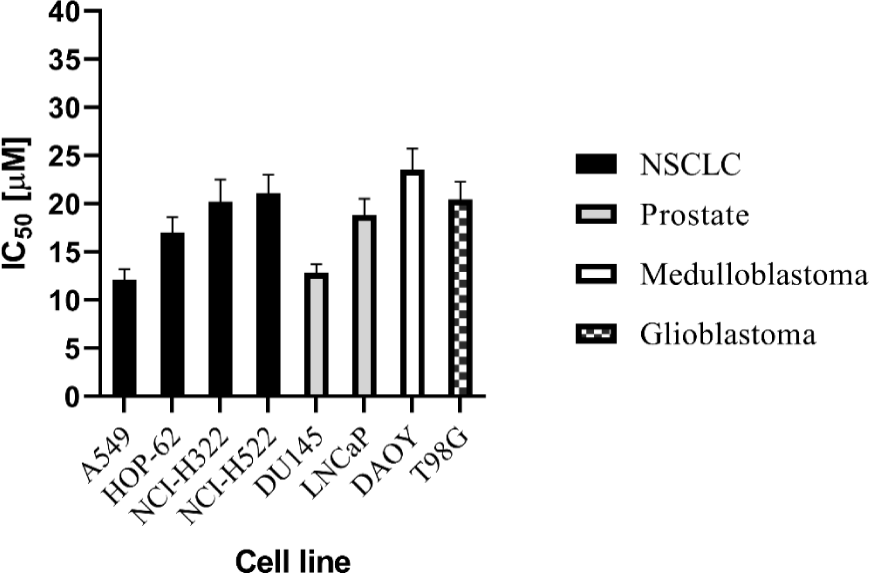
Cytotoxic activity of **11b** toward cell lines derived from different cancer types. The bar columns represent the mean of three independent experiments with standard deviation.

To assess the cytotoxicity of the most active compound **11b** on relevant cancer types with active Hedgehog signaling, we tested it across a panel of cell lines with different histogenetic origins (Figure 3). Concerning nonsmall cell lung carcinoma (NSCLC)-derived cell lines, we selected two subtype groups with different expression levels of GLI1, based on a study by Yuan et al.^35^ The first group includes cell lines where GLI1 is highly expressed (A549 and NCI-H522), while the second group includes low GLI1-expressing cell lines (HOP-62 and NCI-H322). It has been established that cell lines expressing high levels of GLI1 are relatively resistant to SMO antagonists due to a hyper-activated HH signaling pathway.^35^ Our results showed that among the entire panel of NSCLC-derived cell lines, A549 displayed the highest sensitivity toward **11b,** whereas IC_50_ values of HOP-62 and NCI-H322 were almost two times higher. These interesting findings may indicate the potential of **11b** to overcome hyper-activated HH signaling in particular NSCLC subsets. Regarding the sensitivity of prostate tumor-derived cell lines, the MTS assay indicated that the DU145 cell line was more sensitive than LNCaP cells. Lower activity was observed against medulloblastoma (DAYO) and glioblastoma (T98G)-derived cell lines with an IC_50_ above 20 μmol/L.

#### Pharmacological Parameters

Preclinical studies of absorption, distribution, metabolism, and excretion (ADME) are the link between the laboratory development of candidate compounds and the initiation of human clinical trials. In fact, the ADME parameters obtained from *in vitro* and *in vivo* models can further narrow the list of potential clinical candidates.

We subjected **11a** and **11b** to *in vitro* ADME analyses (Table 3), specifically plasma stability, which is important for the fast determination of the instability of test compounds in plasma that can lead to rapid *in vivo* clearance and poor pharmacokinetics.^36^ Both compounds were found to be stable in human plasma (more than 95% presence in plasma after 120 min). Data from a plasma protein binding study can inform about distribution into tissues of the body and reduction in the amount of drug available to be metabolized or cleared from the body. The measurement of plasma protein binding was performed using a Rapid Equilibrium Dialysis device; **11a** and **11b** compounds reported a percent of fraction bound around 90%. For the microsomal stability assay, human liver microsomes and NADPH cofactor were used to assess phase I oxidation by cytochrome P450 and flavin monooxygenases. The intrinsic clearance calculated from the microsomal stability assay indicated the medium category for both derivatives (Table 3).

The Parallel Artificial Membrane Permeability Assay (PAMPA) has emerged as a primary screen for determining passive transcellular permeability. Both tested derivatives, **11a** and **11b,** had low ability (−log *P*_app_ > 6 cm/s) to diffuse passively through an artificial cellular membrane, suggesting an alternative transport mechanism. The Caco-2 and MDCK-MDR1 permeability assays are established models of intestinal^37^ and blood-brain barriers, respectively.^38^ It can be concluded that molecules **11a** and **11b** showed low (*P*_app_AB < 5 × 10^–6^ cm/s) probability of intestinal absorption and of crossing blood-brain barriers (*P*_app_AB < 10 × 10^–6^ cm/s; CNS+). We assessed rates of transport across Caco-2 and MDCK-MDR1 monolayers in both directions (apical to basolateral (A–B) and basolateral to apical (B–A)) across the cell monolayer, which enabled us to determine the efflux ratio and showed if the compound undergoes active efflux. The studied compounds manifested that those values of efflux ratio were less than or very close to the limit of active and passive efflux (≤2), indicating that the compound cannot be clearly identified as substrates of the MDR1 efflux pump present in both cell types.

**Table 3:**
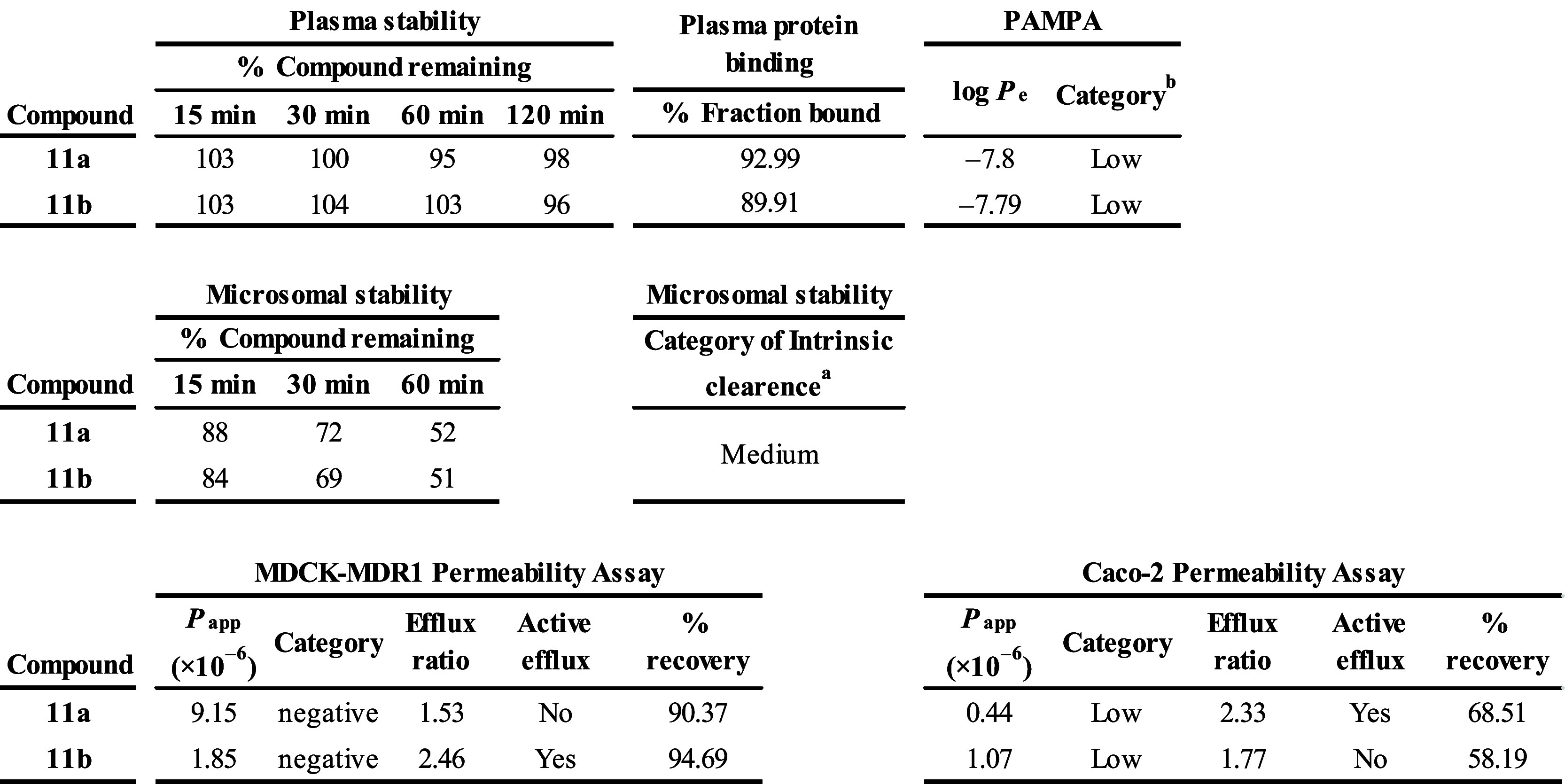
Pharmacological Parameters of Compounds **11a** and **11b**^i^

i ^a,b^ References (39) and (40); error deviations are within a range of values less than 10% (all experiments were done in triplicates, except for cell-based permeability assays, which were performed in duplicates).

## Conclusion

This study follows our previous screening of a large library of known triterpenoids that revealed an interesting inhibition of Hh signaling. Based on these findings, we designed and synthesized a new library of triterpenoid analogues containing an aromatic heterocyclic substituent at position C-2, with the aim of enhancing their inhibitory activity against the Hh pathway. The synthesized compounds were evaluated for their biological activity, including cytotoxicity and Hh inhibition assays. It was found that the new triterpenoid analogues displayed cytotoxic effects against cell lines derived from different cancer types with constitutive Hh signaling. The two most active compounds, **11a** and **11b**, both containing a furan heterocycle at position C-2, demonstrated superior efficacy compared to the established Gli1/2 inhibitor GANT-61 at equimolar concentrations. Data from reporter assays and expression analysis consistently indicated inhibition of endogenous Gli1 protein levels as well as proteins that promote cell proliferation and survival in A549 and DU-145 cancer cells. These results are highly promising for the development of new anticancer treatments targeting tumors dependent on Hh signaling. Therefore, the structures of compounds **11a** and **11b** and their potential therapeutic applications have been patented. These compounds will undergo *in vivo* screening in mouse models to evaluate their efficacy and safety profiles as prerequisites for potential clinical application.

## Experimental Section

### Chemistry

#### General Information

All reagents were of reagent grade and were used without further purification. Allobetulone (**1**) betulinic acid (**5**), benzyl betulinate (**6**), and benzyl betulonate (**7**) in purity 98%+ were purchased from the company Betulinines (www.betulinines.com). All other chemicals and solvents were purchased from Sigma-Aldrich (www.sigmaaldrich.com), Acros Organics (www.acros.com), and Fluorochem (www.fluorochem.co.uk). Dry solvents were dried over 4 Å molecular sieves or stored as received from commercial suppliers. The course of the reactions was monitored by TLC on Kieselgel 60 F_254_ plates (Merck, Germany) and detected by UV light (254 nm), followed by spraying with 10% aqueous H_2_SO_4_ and heating to 150–200 °C. Purification was performed using column chromatography on Silica gel 60 Merck 7734 (Merck, Germany). Melting points (MP) were determined only for crystalline compounds using the STUART SMP30 apparatus and are uncorrected. Optical rotations were measured using THF solutions on an Automatic Compact Polarimeter Atago Pol 1/2, and the concentration of each sample was 1 g/100 mL at 22 °C. IR spectra were recorded on a Thermo Nicolet AVATAR 370 FTIR. DRIFT stands for diffuse reflectance infrared Fourier Transform. NMR spectra were recorded on a JEOL ECX500 spectrometer at a magnetic field strength of 11.75 T with operating frequencies of 500.16 MHz for ^1^H and 125.77 MHz for ^13^C at ambient temperature (25 °C). Chemical shifts δ are reported in parts per million (ppm) and coupling constants *J* are reported in Hertz (Hz). The ^1^H and ^13^C NMR chemical shifts were referenced to the residual signals of CDCl_3_ (7.26 ppm for ^1^H and 77.16 ppm for ^13^C). HRMS analysis was performed using an LC-MS Orbitrap Elite high-resolution mass spectrometer with electrospray ionization (Dionex Ultimate 3000, Thermo Exactive plus, MA, USA) operating in positive and negative full scan mode in the range of *m*/*z* = 400–700. The settings for electrospray ionization were as follows: oven temperature of 150 °C and source voltage of 3.6 kV. The acquired data were internally calibrated with phthalate as a contaminant in MeOH (*m*/*z* = 297.15909). The samples were diluted to a final concentration of 0.1 mg/mL in MeOH and injected into the mass spectrometer over an autosampler after HPLC separation (Phenomenex Gemini column, C18, 50 × 2.00 mm, 2.6 μm particles) using the mobile phase isocratic MeOH/ammonium acetate 0.01 mol·L^–1^/HCOOH 95:5:0.1 and a flow rate of 0.3 mL/min.

##### Synthesis of 2-Hydroxy-19β,28-epoxyolean-1-en-3-one (**2**)

Compound **2** was prepared via oxidation of allobetulone (**1**) (3.0 g; 6.8 mmol) by O_2_ (air) with potassium *tert*-butoxide (3.0 g; 26.7 mmol) in *tert*-butanol (150 mL) after 4 h, monitored by TLC (Hex/EtOAc 8:1 + 0.1% CHCl_3_). The reaction mixture was extracted with EtOAc, washed with aqueous HCl (1:10), an aqueous solution of NaCl, and water to neutral pH. The organic phase was dried with anhydrous magnesium sulfate, filtered, and evaporated. After purification (Hex/EtOAc 8:1 + 0.1% CHCl_3_), 3.08 g (93%) of compound **2** was obtained. ^1^H NMR spectrum was consistent with the literature.^41^

##### Synthesis of 2-{[(trifluoromethyl)sulfonyl]oxy}-19β,28-epoxyolean-1-en-3-one (**3**)

To the solution of diosphenol **2** (800 mg; 1.76 mmol) in dry DCM (8 mL), Tf_2_NPh (943 mg; 2.64 mmol), DMAP (22 mg; 0.176 mmol), and TEA (387 μL; 5.28 mmol) were added. The reaction was performed in a Schlenk tube under a nitrogen atmosphere. A yellow reaction mixture became red while stirring at room temperature for 2 h. The reaction mixture was extracted with EtOAc, washed with an aqueous solution of NH_4_Cl, an aqueous solution of NaCl, and water to neutral pH. The organic phase was dried with anhydrous magnesium sulfate, filtered, and evaporated. After purification (Hex/EtOAc 6:1), 931 mg (90%) of compound **3** was obtained as a colorless oil. IR (DRIFT) ν_max_ 2926, 2855, 1704 (C=O), 1672 (C=C furan), 1204 (C–O), 1140 (C–O) cm^–1^; ^1^H NMR (500 MHz, CDCl_3_) δ 7.08 (s, 1H, H-1), 3.79 (d, *J* = 7.9 Hz, 1H, H-28a), 3.59 (s, 1H, H-19), 3.49 (d, *J* = 7.9 Hz, 1H, H-28b), 1.79–1.73 (m, 1H), 1.73–1.65 (m, 2H), 1.23 (s, 3H), 1.21 (s, 3H), 1.15 (s, 3H), 1.05 (s, 3H), 0.94 (s, 3H), 0.94 (s, 6H), 0.81 (s, 3H, 7 × CH_3_) ppm. Spectra are consistent with the literature.^42^

#### General Procedure for the Synthesis of Compounds **4a**–**4n** and **10a**–**10n** by the Suzuki–Miyaura Cross-Coupling

A round-bottom flask with a stirrer was annealed, cooled with a stream of nitrogen gas nitrogen, and closed with a septum. The starting compounds were dissolved in appropriate solvents and then injected into the prepared flask: 100 mg of triflate **3** or **9** was dissolved in 0.6 mL of 1,4-dioxane; 2 equivalents of the corresponding boronic acid were dissolved in 0.6 mL of isopropyl alcohol, and 2 equivalents of anhydrous potassium carbonate were dissolved in 0.3 mL of water. After that, PdCl_2_(PPh_3_)_2_ (2 mol %) was added. The flask was equipped with a reflux condenser, and the reaction mixture was stirred at 85 °C under a nitrogen atmosphere and monitored by TLC. The reaction time was from 2 h to overnight (depending on the respective derivative). After completion, the reaction mixture was extracted with EtOAc, washed with an aqueous solution of NH_4_Cl, an aqueous solution of NaCl, and with water to a neutral pH. The organic phase was dried with anhydrous magnesium sulfate, filtered, and evaporated. The residue was purified using column chromatography on silica gel (mobile phases are listed in each experiment).

##### 2-(Furan-2-yl)-19β,28-epoxyolean-1-en-3-one (**4a**)

Compound **4a** was prepared according to the general procedure from triflate **3** (100 mg; 0.17 mmol) and 2-furanylboronic acid (38.2 mg; 0.34 mmol). After the work-up and purification (Hex/EtOAc 12:1), white crystals of compound **4a** (72 mg; 84%) were obtained. MP 201 °C (Hex/EtOAc); [α]_D_ +54; IR (DRIFT) ν_max_ 1678 (C=O), 1218 (C–O–C furan), 1036 (C–O–C) cm^–1^; ^1^H NMR (500 MHz, CDCl_3_) δ 7.52 (s, 1H, H-1), 7.36–7.33 (m, 1H, H-furan), 6.84–6.81 (m, 1H, H-furan), 6.42–6.40 (m, 1H, H-furan), 3.82–3.76 (m, 1H, H-28a), 3.56 (s, 1H, H-19), 3.47 (d, *J* = 7.8 Hz, 1H, H-28b), 1.18 (s, 3H), 1.14 (s, 3H), 1.10 (s, 3H), 1.07 (s, 3H), 0.95 (s, 3H), 0.94 (s, 3H), 0.81 (s, 3H, 7 × CH_3_) ppm; ^13^C NMR (126 MHz, CDCl_3_) δ 202.76, 152.44, 149.53, 141.37, 126.04, 111.77, 109.60, 88.04, 71.44, 52.72, 46.90, 45.51, 45.47, 41.66, 41.64, 41.18, 39.03, 36.88, 36.44, 34.62, 33.33, 32.85, 29.85, 28.95, 28.88, 26.56, 26.41, 24.70, 21.82, 21.65, 19.91, 19.50, 16.32, 13.48 ppm; HRMS (ESI^+^) *m*/*z* calcd for C_34_H_49_O_3_ [M + H]^+^ 505.3676, found 505.3678.

##### 2-(Furan-3-yl)-19β,28-epoxyolean-1-en-3-one (**4b**)

Compound **4b** was prepared according to the general procedure from triflate **3** (100 mg; 0.17 mmol) and 3-furanylboronic acid (38.2 mg; 0.34 mmol). After the work-up and purification (Hex/EtOAc 10:1), 86 mg (85%) of crystalline **4b** was obtained. MP 158–160 °C (Hex/EtOAc); [α]_D_ +30; IR (DRIFT) ν_max_ 1678 (C=O), 1225 (C–O–C furan), 1035 (C–O–C) cm^–1^; ^1^H NMR (500 MHz, CDCl_3_) δ 7.98–7.95 (m, 1H, H-furan), 7.39–7.36 (m, 1H, H-furan), 7.20 (s, 1H, H-1), 6.53–6.50 (m, 1H, H-furan), 3.82–3.77 (m, 1H, H-28a), 3.57 (s, 1H, H-19), 3.47 (d, *J* = 7.8 Hz, 1H, H-28b), 1.18 (s, 3H), 1.14 (s, 3H), 1.10 (s, 3H), 1.08 (s, 3H), 0.96 (s, 3H), 0.95 (s, 3H), 0.82 (s, 3H, 7 × CH_3_) ppm; ^13^C NMR (126 MHz, CDCl_3_) δ 204.17, 153.53, 142.51, 141.79, 127.59, 120.79, 108.17, 88.02, 71.42, 52.82, 46.90, 45.47, 45.36, 41.64 (2C), 41.18, 39.19, 36.87, 36.44, 34.60, 33.35, 32.84, 28.95, 28.92, 26.58, 26.55, 26.40, 24.70, 21.78, 21.69, 20.00, 19.46, 16.32, 13.50 ppm; HRMS (ESI^+^) *m*/*z* calcd for C_34_H_49_O_3_ [M + H]^+^ 505.3676, found 505.3678.

##### 2-(Thiophene-2-yl)-19β,28-epoxyolean-1-en-3-one (**4c**)

Compound **4c** was prepared according to the general procedure from triflate **3** (100 mg; 0.17 mmol) and 2-thiopheneboronic acid (43.6 mg; 0.34 mmol). After the work-up and purification (Hex/EtOAc 7:1 and Hex/EtOAc 8:1), white crystals of compound **4c** (66%; 59 mg) were obtained. MP 242 °C (Hex/EtOAc); IR (DRIFT) ν_max_ 1685 (C=O), 1035 (C–O–C) cm^–1^; ^1^H NMR (500 MHz, CDCl_3_) δ 7.35 (s, 1H, H-1), 7.29–7.27 (m, 1H, H-thiophene), 7.26–7.24 (m, 1H, H-thiophene), 7.01–6.98 (m, 1H, H-thiophene), 3.82–3.77 (m, 1H, H-28a), 3.57 (s, 1H, H-19), 3.47 (d, *J* = 7.8 Hz, 1H, H-28b), 1.21 (s, 3H), 1.16 (s, 3H), 1.11 (s, 3H), 1.08 (s, 3H), 0.96 (s, 3H), 0.95 (s, 3H), 0.82 (s, 3H, 7 × CH_3_) ppm; ^13^C NMR (126 MHz, CDCl_3_) δ 203.55, 153.89, 129.63, 126.81, 125.83, 124.98, 88.03, 71.43, 52.75, 46.90, 45.45, 45.41, 41.68, 41.65, 41.20, 39.47, 36.88, 36.44, 34.62, 33.29, 32.84, 28.96 (2C), 26.57, 26.56, 26.40, 24.71, 21.83, 21.61, 19.84, 19.49, 16.32, 13.52 ppm; HRMS (ESI^+^) *m*/*z* calcd for C_34_H_49_O_2_S [M + H]^+^ 521.3448, found 521.3451.

##### 2-(Thiophene-3-yl)-19β,28-epoxyolean-1-en-3-one (**4d**)

Compound **4d** was prepared according to the general procedure from triflate **3** (100 mg; 0.17 mmol) and 3-thiopheneboronic acid (43.6 mg; 0.34 mmol). After the work-up and purification (Hex/EtOAc 8:1), 66 mg (74%) of compound **4d** was obtained as an amorphous solid. IR (DRIFT) ν_max_ 1671 (C=O), 1034 (C–O–C) cm^–1^; ^1^H NMR (500 MHz, CDCl_3_) δ 7.56–7.53 (m, 1H, H-thiophene), 7.27–7.24 (m, 2H, H-thiophene and H-1), 7.20–7.17 (m, 1H, H-thiophene), 3.81–3.76 (m, 1H, H-28a), 3.56 (s, 1H, H-9), 3.47 (d, *J* = 7.9 Hz, 1H, H-28b), 1.20 (s, 3H), 1.15 (s, 3H), 1.10 (s, 3H), 1.08 (s, 3H), 0.95 (s, 6H), 0.82 (s, 3H, 7 × CH_3_) ppm; ^13^C NMR (126 MHz, CDCl_3_) δ 204.59, 154.69, 137.21, 130.88, 126.91, 125.05, 123.13, 88.03, 71.43, 52.86, 46.90, 45.59, 45.51, 41.65, 41.63, 41.17, 39.23, 36.88, 36.44, 34.64, 33.31, 32.85, 28.96, 26.59, 26.56, 26.41, 24.71, 21.89, 21.57, 19.86, 19.58, 16.31, 13.50 ppm; HRMS (ESI^+^) *m*/*z* calcd for C_34_H_49_O_2_S [M + H]^+^ 521.3448, found 521.3450.

##### 2-(5-Chlorothiophen-2-yl)-19β,28-epoxyolean-1-en-3-one (**4e**)

Compound **4e** was prepared according to the general procedure from triflate **3** (100 mg; 0.17 mmol) and 5-chlorothiophene-2-boronic acid (55 mg; 0.34 mmol). After the work-up and purification (Hex/EtOAc 5:1), 69 mg (73%) of compound **4e** was obtained as an amorphous solid. IR (DRIFT) ν_max_ 1667 (C=O), 1034 (C–O–C), 812 (C–Cl) cm^–1^; ^1^H NMR (500 MHz, CDCl_3_) δ 7.30 (s, 1H, H-1), 7.03 (d, *J* = 4.0 Hz, 1H, H-thiophene), 6.80 (d, *J* = 4.0 Hz, 1H, H-thiophene), 3.79 (d, *J* = 7.9 Hz, 1H, H-28a), 3.56 (s, 1H, H-19), 3.47 (d, *J* = 7.9 Hz, 1H, H-8b), 1.19 (s, 3H), 1.15 (s, 3H), 1.10 (s, 3H), 1.07 (s, 3H), 0.96 (s, 3H), 0.95 (s, 3H), 0.82 (s, 3H, 7 × CH_3_) ppm; ^13^C NMR (126 MHz, CDCl_3_) δ 203.36, 153.59, 136.51, 130.88, 129.12, 125.50, 123.43, 88.02, 71.41, 52.74, 46.89, 45.34, 45.29, 41.73, 41.64, 41.21, 39.56, 36.86, 36.44, 34.58, 33.27, 32.83, 28.95, 28.89, 26.54 (2C), 26.39, 24.70, 21.77, 21.60, 19.82, 19.39, 16.33, 13.52 ppm; HRMS (ESI^+^) *m*/*z* calcd for C_34_H_48_ClO_2_S [M + H]^+^ 555.3058, found 555.3059.

##### 2-(Pyridin-3-yl)-19β,28-epoxyolean-1-en-3-one (**4g**)

Compound **4g** was prepared according to the general procedure from triflate **3** (100 mg; 0.17 mmol) and 3-pyridineboronic acid (42 mg; 0.34 mmol). After the work-up and purification (Hex/EtOAc 2:3), 67 mg (76%) of compound **4g** was obtained as an amorphous solid. IR (DRIFT) ν_max_ 1671 (C=O), 1327 (arom. C–N), 1035 (C–O–C) cm^–1^; ^1^H NMR (500 MHz, CDCl_3_) δ 8.55–8.48 (m, 2H, H-pyridine), 7.69–7.64 (m, 1H, H-pyridine), 7.26–7.23 (m, 2H, H-1, H-pyridine), 3.81–3.76 (m, 1H, H-28a), 3.55 (s, 1H, H-19), 3.46 (d, *J* = 7.8 Hz, 1H, H-28b), 1.20 (s, 3H), 1.19 (s, 3H), 1.15 (s, 3H), 1.08 (s, 3H), 0.95 (s, 3H), 0.94 (s, 3H), 0.80 (s, 3H, 7 × CH_3_) ppm; ^13^C NMR (126 MHz, CDCl_3_) δ 157.61, 149.09, 148.78, 136.09, 133.48, 133.14, 122.90, 88.00, 71.39, 60.51, 53.17, 46.85, 45.42, 41.67, 41.62, 41.17, 39.55, 36.84, 36.41, 34.59, 33.30, 32.82, 28.92, 28.75, 26.53, 26.47, 26.37, 24.68, 21.81, 21.56, 19.82, 19.50, 16.33, 13.49 ppm; HRMS (ESI^+^) *m*/*z* calcd for C_35_H_50_O_2_N [M + H]^+^ 516.3836, found 516.3839.

##### 2-(Pyridine-4-yl)-19β,28-epoxyolean-1-en-3-one (**4h**)

Compound **4h** was prepared according to the general procedure from triflate **3** (100 mg; 0.17 mmol) and 4-pyridineboronic acid (41.9 mg; 0.34 mmol). After the work-up and purification (Hex/EtOAc 2:1 + 0.01% CH_3_COOH), 65 mg (74%) of compound **4h** was obtained as an amorphous solid. IR (DRIFT) ν_max_ = 1684 (C=O), 1313 (arom. C–N), 1036 (C–O–C) cm^–1^; ^1^H NMR (500 MHz, CDCl_3_) δ 8.58–8.54 (m, 2H, H-pyridine), 7.30 (s, 1H, H-1), 7.26–7.23 (m, 2H, H-pyridine), 3.81–3.76 (m, 1H, H-28a), 3.55 (s, 1H, H-19), 3.47 (d, *J* = 7.8 Hz, 1H, H-28b), 1.21 (s, 3H), 1.18 (s, 3H), 1.13 (s, 3H), 1.09 (s, 3H), 0.95 (s, 3H), 0.94 (s, 3H), 0.80 (s, 3H, 7 × CH_3_) ppm; ^13^C NMR (126 MHz, CDCl_3_) δ 203.82, 158.20, 149.84 (2C), 144.92, 134.38, 123.00 (2C), 88.00, 71.40, 53.04, 46.86, 45.56, 45.36, 41.68, 41.63, 41.17, 39.59, 36.85, 36.42, 34.60, 33.25, 32.81, 28.93, 28.73, 26.53, 26.49, 26.37, 24.67, 21.87, 21.48, 19.63, 19.52, 16.33, 13.48 ppm; HRMS (ESI^+^) *m*/*z* calcd for C_35_H_50_O_2_N [M + H]^+^ 516.3836, found 516.3839.

##### 2-(Pyrimidin-5-yl)-19β,28-epoxyolean-1-en-3-one (**4i**)

Compound **4i** was prepared according to the general procedure from triflate **3** (100 mg; 0.17 mmol) and 5-pyrimidineboronic acid (42 mg; 0.34 mmol). After the work-up and purification (Hex/EtOAc 5:1), 63 mg (72%) of compound **4i** was obtained as an amorphous solid. IR (DRIFT) ν_max_ 1670 (C=O), 1336 (arom. C–N), 1293 (arom. C–N), 1034 (C–O–C) cm^–1^; ^1^H NMR (500 MHz, CDCl_3_) δ 9.11 (s, 1H, H-pyrimidine), 8.69 (s, 2H, H-pyrimidine), 7.29 (s, 1H, H-1), 3.78 (d, *J* = 7.8 Hz, 1H, H-28a), 3.54 (s, 1H, H-19), 3.46 (d, *J* = 7.8 Hz, 1H, H-28b), 1.20 (s, 3H), 1.19 (s, 3H), 1.16 (s, 3H), 1.08 (s, 3H), 0.95 (s, 3H), 0.93 (s, 3H), 0.80 (s, 3H, 7 × CH_3_) ppm; ^13^C NMR (126 MHz, CDCl_3_) δ 158.96, 157.64, 156.15, 131.02, 130.62, 87.98, 71.36, 53.23, 46.83, 45.34, 41.73, 41.61, 41.18, 39.84, 36.82, 36.40, 34.55, 33.27, 32.79, 28.90, 28.65, 26.50, 26.41, 26.34, 24.66, 21.76, 21.59, 19.79, 19.40, 16.35, 13.48 ppm; HRMS (ESI^+^) *m*/*z* calcd for C_34_H_49_O_2_N_2_ [M + H]^+^ 517.3789, found 517.3790.

##### 2-(1,4-Benzodioxan-6-yl)-19β,28-epoxyolean-1-en-3-one (**4j**)

Compound **4j** was prepared according to the general procedure from triflate **3** (100 mg; 0.17 mmol) and 1,4-benzodioxane-6-boronic acid (61.4 mg; 0.34 mmol). After the work-up and purification (Hex/EtOAc 5:1), 59 mg (60%) of compound **4j** was obtained as an amorphous solid. IR (DRIFT) ν_max_ 1670 (C=O), 1035 (C–O–C) cm^–1^; ^1^H NMR (500 MHz, CDCl_3_) δ 7.10 (s, 1H, H-1), 6.86–6.77 (m, 3H, H-arom. ring), 4.28–4.23 (m, 4H, H-dioxane), 3.79 (d, *J* = 7.7 Hz, 1H, H-28a), 3.56 (s, 1H, H-19), 3.47 (d, *J* = 7.8 Hz, 1H, H-28b), 1.19 (s, 3H), 1.16 (s, 3H), 1.09 (s, 3H), 1.07 (s, 3H), 0.95 (s, 3H), 0.93 (s, 3H), 0.81 (s, 3H, 7 × CH_3_) ppm; ^13^C NMR (126 MHz, CDCl_3_) δ 204.79, 155.17, 143.37, 143.29, 135.77, 130.90, 121.55, 117.23, 117.07, 88.04, 71.42, 64.62, 64.51, 53.08, 46.88, 45.52, 45.48, 41.64, 41.57, 41.14, 39.20, 36.87, 36.42, 34.62, 33.32, 32.85, 28.94, 28.83, 26.54 (2C), 26.40, 24.70, 21.87, 21.44, 19.76, 19.60, 16.28, 13.47 ppm; HRMS (ESI^+^): *m*/*z* calcd for C_38_H_53_O_4_ [M + H]^+^ 573.3938, found 573.3935.

##### 2-(Benzo[b]thien-2-yl)-19β,28-epoxyolean-1-en-3-one (**4k**)

Compound **4k** was prepared according to the general procedure from triflate **3** (100 mg; 0.17 mmol) and benzo[b]thien-2-ylboronic acid (60.7 mg; 0.34 mmol). After the work-up and purification (Hex/EtOAc 9:1), 79 mg (81%) of compound **4k** was obtained as an amorphous solid. IR (DRIFT) ν_max_ 1673 (C=O), 1034 (C–O–C), 657 (C–S) cm^–1^; ^1^H NMR (500 MHz, CDCl_3_) δ 7.79–7.75 (m, 1H), 7.73–7.69 (m, 1H), 7.60 (s, 1H), 7.42 (s, 1H, H-1), 7.33–7.26 (m, 1H), 3.82–3.78 (d, *J* = 8.6 Hz, 1H, H-28a), 3.58 (s, 1H, H-19), 3.48 (d, *J* = 7.9 Hz, 1H, H-28b), 1.24 (s, 3H), 1.18 (s, 3H), 1.13 (s, 3H), 1.09 (s, 3H), 0.98 (s, 3H), 0.96 (s, 3H), 0.83 (s, 3H, 7 × CH_3_) ppm; ^13^C NMR (126 MHz, CDCl_3_) δ 203.42, 155.75, 140.23, 139.45, 138.98, 129.98, 124.57, 124.39, 123.77, 122.66, 122.03, 88.05, 71.42, 52.62, 46.90, 45.73, 45.36, 41.68, 41.65, 41.21, 39.69, 36.87, 36.44, 34.63, 33.23, 32.84, 29.02, 28.96, 26.59, 26.56, 26.39, 24.71, 21.93, 21.56, 19.74, 19.57, 16.30, 13.53 ppm; HRMS (ESI^+^): *m*/*z* calcd for C_38_H_51_O_2_S [M + H]^+^ 571.3604, found 571.3604.

##### 2-(Isoquinolin-4-yl)-19β,28-epoxyolean-1-en-3-one (**4l**)

Compound **4l** was prepared according to the general procedure from triflate **3** (100 mg; 0.17 mmol) and isoquinoline-4-boronic acid (58.9 mg; 0.34 mmol). After the work-up and purification (Hex/EtOAc 1:1), 59 mg (68%) of crystalline compound **4l** was obtained. MP 165–167 °C (Hex/EtOAc); [α]_D_ +40; IR (DRIFT) ν_max_ 1668 (C=O), 1034 (C–O–C) cm^–1^; ^1^H NMR (500 MHz, CDCl_3_) δ 9.20 (s, 1H, H-arom. ring), 8.27 (s, 1H, H-arom. ring), 8.00–7.96 (m, 1H, H-arom. ring), 7.68–7.63 (m, 1H, H-arom. ring), 7.62–7.55 (m, 2H, H-arom. ring), 7.29 (s, 1H, H-1), 3.79 (d, *J* = 7.7 Hz, 1H, H-28a), 3.53 (s, 1H, H-19), 3.47 (d, *J* = 7.7 Hz, 1H, H-28b), 1.32 (s, 3H), 1.31 (s, 3H), 1.26 (s, 3H), 1.12 (s, 3H), 0.97 (s, 3H), 0.93 (s, 3H), 0.78 (s, 3H, 7 × CH_3_) ppm; ^13^C NMR (126 MHz, CDCl_3_) δ 203.87, 160.58, 152.67, 143.28, 135.06, 133.23, 130.42, 129.64, 128.33, 128.03, 127.17, 124.62, 87.99, 71.39, 53.73, 53.55, 46.83, 45.46, 45.41, 41.75, 41.62, 41.19, 39.92, 36.84, 36.40, 34.56, 33.42, 32.82, 29.15, 28.90, 26.55, 26.37, 24.65, 21.89, 21.66, 20.25, 19.50, 16.41, 13.55 ppm; HRMS (ESI^+^): *m*/*z* calcd for C_39_H_52_O_2_N [M + H]^+^ 566.3993, found 566.3994.

##### 2-(Indol-5-yl)-19β,28-epoxyolean-1-en-3-one (**4m**)

Compound **4m** was prepared according to the general procedure from triflate **3** (100 mg; 0.17 mmol) and indole-5-boronic acid (55.3 mg; 0.34 mmol). After the work-up and purification (Hex/EtOAc 5:1), white crystals of compound **4m** (68 mg; 62%) were obtained. MP 147–148 °C (Hex/EtOAc); [α]_D_ +30; IR (DRIFT) ν_max_ 3353 (N–H), 1652 (C=O), 1033 (C–O–C) cm^–1^; ^1^H NMR (500 MHz, CDCl_3_) δ 8.17 (s, 1H, indole N–H), 7.60–7.57 (m, 1H, H-indole), 7.37–7.32 (m, 1H, H-indole), 7.20–7.17 (m, 2H, H-1, H-indole), 7.15–7.11 (m, 1H, H-indole), 6.55–6.51 (m, 1H, H-indole), 3.80 (d, *J* = 7.5 Hz, 1H, H-28a), 3.56 (s, 1H, H-19), 3.47 (d, *J* = 7.8 Hz, 1H, H-28b), 1.22 (s, 3H), 1.22 (s, 3H), 1.14 (s, 3H), 1.09 (s, 3H), 0.95 (s, 6H), 0.81 (s, 3H, 7 × CH_3_) ppm; ^13^C NMR (126 MHz, CDCl_3_) δ 205.38, 155.10, 137.34, 135.55, 129.36, 128.08, 124.62, 122.83, 120.53, 110.70, 103.11, 88.04, 71.43, 53.17, 46.89, 45.61, 45.52, 41.65, 41.57, 41.15, 39.24, 36.88, 36.43, 34.65, 33.39, 32.86, 29.85, 28.96, 28.91, 26.57, 26.42, 24.71, 21.90, 21.52, 19.88, 19.67, 16.30, 13.49 ppm; HRMS (ESI^+^) *m*/*z* calcd for C_38_H_52_O_2_N [M + H]^+^ 554.3993, found 554.3994.

##### 2-(Indazol-6-yl)-19β,28-epoxyolean-1-en-3-one (**4n**)

Compound **4n** was prepared according to the general procedure from triflate **3** (100 mg; 0.17 mmol) and indazole-6-boronic acid (55 mg; 0.34 mmol). After the work-up and purification (Hex/EtOAc 5:1), 56 mg (59%) of compound **4n** was obtained as an amorphous solid. IR (DRIFT) ν_max_ 3222 (N–H), 1670 (C=O), 1035 (C–O–C) cm^–1^; ^1^H NMR (500 MHz, CDCl_3_) δ 10.29 (s, 1H, indazole–N-H), 8.04–8.02 (m, 1H, H-indazole), 7.72–7.68 (m, 1H, H-indazole), 7.50–7.48 (m, 1H, H-indazole), 7.27 (s, 1H, H-1), 7.11–7.07 (m, 1H, H-indazole), 3.80 (d, *J* = 7.1 Hz, 1H, H-28a), 3.56 (s, 1H, H-19), 3.48 (d, *J* = 7.8 Hz, 1H, H-28b), 1.23 (s, 3H), 1.22 (s, 3H), 1.15 (s, 3H), 1.10 (s, 3H), 0.95 (s, 3H), 0.95 (s, 3H), 0.80 (s, 3H, 7 × CH_3_) ppm; ^13^C NMR (126 MHz, CDCl_3_) δ 204.99, 156.91, 140.40, 136.53, 136.19, 134.92, 122.72, 121.97, 120.52, 109.48, 88.02, 71.41, 53.10, 46.88, 45.62, 45.51, 41.63, 41.16, 39.44, 36.85, 36.42, 34.62, 33.31, 32.83, 29.84, 28.94, 28.89, 26.54, 26.38, 24.69, 21.90, 21.52, 19.80, 19.61, 16.31, 13.49 ppm; HRMS (ESI^+^) *m*/*z* calcd for C_37_H_51_O_2_N_2_ [M + H]^+^ 555.3946, found 555.3947.

##### Benzyl 2-Hydroxy-3-oxolupa-1,20(29)-dien-28-oate (**8**)

Diosphenol **8** was prepared via oxidation of benzyl betulonate (**7**, 1500 mg; 2.80 mmol) by O_2_ (air) with potassium *tert*-butoxide (1250 mg; 11.2 mmol) in *tert*-butanol (200 mL) after 3.5 h, monitored by TLC (Hex/EtOAc 10:1). The reaction mixture was extracted with EtOAc, washed with aqueous HCl (1:10), an aqueous solution of NaCl, and with water to neutral pH. The organic phase was dried with anhydrous magnesium sulfate, filtered, and evaporated. After purification (Hex/EtOAc 10:1), 1400 mg (90%) of compound **8** was obtained. ^1^H NMR spectrum was consistent with the literature.^43^

##### Benzyl 2-{[(trifluoromethyl)sulfonyl]oxy}-3-oxolupa-1,20(29)-dien-28-oate (**9**)

To the solution of diosphenol **8** (920 mg; 1.646 mmol) in dry DCM (10 mL), Tf_2_NPh (882 mg; 2.469 mmol), DMAP (20 mg; 0.165 mmol), and TEA (362 μL; 4.938 mmol) were added. The reaction was performed in a Schlenk tube under a nitrogen atmosphere. A yellow reaction mixture turned red while stirring at room temperature for 2 h. After completion, the mixture was extracted with EtOAc, washed with an aqueous solution of NH_4_Cl, an aqueous solution of NaCl, and with water to neutral pH. The organic phase was dried with anhydrous magnesium sulfate, filtered, and evaporated. After purification (Hex/EtOAc 8:1), 990 mg (87%) of triflate **9** was obtained as a colorless foam. ^1^H NMR (500 MHz, CDCl_3_) δ 7.42–7.29 (m, 5H, Ph), 7.03 (s, 1H, H-1), 5.16 (d, *J* = 12.2 Hz, 1H, PhCH_2_–a), 5.10 (d, *J* = 12.2 Hz, 1H, PhCH_2_–b), 4.79–4.70 (m, 1H, H-29a), 4.66–4.58 (m, 1H, H-29b), 3.03 (td, *J* = 11.0, 4.6 Hz, 1H, H-18), 2.35–2.22 (m, 2H), 1.97–1.85 (m, 2H), 1.85–1.77 (m, 1H), 1.69 (s, 3H, H-30), 1.21 (s, 3H), 1.15 (s, 3H), 1.13 (s, 3H), 0.96 (s, 3H), 0.82 (s, 3H, 5× CH_3_) ppm; ^13^C NMR (126 MHz, CDCl_3_) δ 196.10, 175.83, 150.37, 147.73, 142.92, 136.57, 128.66, 128.47, 128.28, 118.75 (q, ^1^*J*_*C–F*_ = 320.5 Hz), 110.03, 65.97, 56.58, 53.22, 49.40, 47.02, 46.19, 44.83, 42.89, 41.92, 40.87, 38.34, 37.01, 33.67, 32.14, 30.64, 29.53, 27.55, 25.42, 21.51, 21.43, 19.47, 19.33, 18.89, 16.43, 14.68 ppm; HRMS (ESI^+^): *m*/*z* calcd for C_38_H_50_F_3_O_6_S [M + H]^+^ 691.3275, found 691.3278.

##### Benzyl 2-(2-Furanyl)-3-oxolupa-1,20(29)-dien-28-oate (**10a**)

Compound **10a** was prepared according to the general procedure from triflate **9** (200 mg; 0.289 mmol) and 2-furanylboronic acid (48.5 mg; 0.434 mmol). After the work-up and purification (Hex/EtOAc 15:1), 98 mg (56%) of compound **10a** was obtained as an amorphous solid. IR (DRIFT) ν_max_ 2939, 2862, 1729 (C=O), 1677 (C=C), 1134 (C–O), 740 cm^–1^; ^1^H NMR (500 MHz, CDCl_3_) δ 7.48 (s, 1H, H-1), 7.39–7.29 (m, 6H), 6.81 (d, *J* = 3.2 Hz, 1H, H-furan), 6.39 (dd, *J*_1_ = 3.3, *J*_2_ = 1.8 Hz, 1H, H-furan), 5.16 (d, *J* = 12.3 Hz, 1H, Ph–CH_2_–a), 5.10 (d, *J* = 12.3 Hz, 1H, Ph–CH_2_–b), 4.75 (s, 1H, H-29a), 4.62 (s, 1H, H-29b), 3.04 (td, *J*_1_ = 10.9, *J*_2_ = 4.8 Hz, 1H, H-19), 1.69 (s, 3H, 30-CH_3_ group), 1.16 (s, 3H), 1.12 (s, 3H), 1.04 (s, 3H), 0.96 (s, 3H), 0.84 (s, 3H, 5 × CH_3_) ppm; ^13^C NMR (126 MHz, CDCl_3_) δ 202.69, 175.90, 152.47, 150.57, 149.53, 141.31, 136.62, 128.66 (2C), 128.46 (2C), 128.26, 125.95, 111.71, 109.92, 109.53, 65.95, 56.68, 52.55, 49.52, 47.06, 45.47, 45.02, 42.87, 41.73, 38.93, 38.65, 37.09, 33.74, 32.24, 30.71, 29.64, 28.86, 25.82, 21.74, 21.66, 19.53 (3C), 16.38, 14.71 ppm; HRMS (ESI^+^): *m*/*z* calcd for C_41_H_53_O_4_ [M + H]^+^ 609.3938, found 609.3942.

##### Benzyl 2-(3-Furanyl)-3-oxolupa-1,20(29)-dien-28-oate (**10b**)

Compound **10b** was prepared according to the general procedure from triflate **9** (200 mg; 0.289 mmol) and 3-furanylboronic acid (48.5 mg; 0.434 mmol). After the work-up and purification (Hex/EtOAc 10:1), 143 mg (81%) of compound **10b** was obtained as an amorphous solid. IR (DRIFT) ν_max_ 2942, 2868, 1723 (C=O), 1672 (C=C), 1149, 1125 (C–O) cm^–1^; ^1^H NMR (500 MHz, CDCl_3_) δ 7.98–7.93 (m, 1H, H-furan), 7.40–7.30 (m, 6H), 7.17 (s, 1H, H-1), 6.53–6.48 (m, 1H, H-furan), 5.17 (d, *J* = 12.3 Hz, 1H, PhCH_2_–a), 5.11 (d, *J* = 12.3 Hz, 1H, PhCH_2_–b), 4.76 (s, 1H, H-29a), 4.64 (s, 1H, H-29b), 3.05 (td, *J*_1_ = 10.9, *J*_2_ = 4.7 Hz, 1H, H-19), 1.71 (s, 3H, 30-CH_3_ group), 1.17 (s, 3H), 1.12 (s, 3H), 1.04 (s, 3H), 0.98 (s, 3H), 0.85 (s, 3H, 5 × CH_3_) ppm; ^13^C NMR (126 MHz, CDCl_3_) δ 204.15, 175.89, 153.55, 150.64, 142.47, 141.75, 136.61, 128.66 (2C), 128.45 (2C), 128.27, 127.53, 120.80, 109.87, 108.17, 65.96, 56.68, 52.64, 49.51, 47.04, 45.33, 45.00, 42.87, 41.71, 39.10, 38.61, 37.08, 33.75, 32.22, 30.73, 29.63, 28.91, 25.81, 21.70 (2C), 19.62, 19.57, 19.50, 16.37, 14.73 ppm; HRMS (ESI^+^) *m*/*z* calcd for C_41_H_53_O_4_ [M + H]^+^ 609.3938, found 609.3944.

##### Benzyl 2-(2-Thiophenyl)-3-oxolupa-1,20(29)-dien-28-oate (**10c**)

Compound **10c** was prepared according to the general procedure from triflate **9** (200 mg; 0.289 mmol) and 2-thiopheneboronic acid (55.5 mg; 0.434 mmol). After the work-up and purification (Hex/EtOAc 12:1), 106 mg (59%) of compound **10c** was obtained as an amorphous solid. IR (DRIFT) ν_max_ 2943, 2868, 1723 (C=O), 1671 (C=C), 1171, 1149, 1125, 696 cm^–1^; ^1^H NMR (500 MHz, CDCl_3_) δ 7.40–7.32 (m, 5H), 7.31 (s, 1H, H-1), 7.27–7.24 (m, 2H), 7.00–6.96 (m, 1H, H-thiophene), 5.17 (d, *J* = 12.3 Hz, 1H, PhCH_2_–a), 5.11 (d, *J* = 12.3 Hz, 1H, PhCH_2_–b), 4.76 (s, 1H, H-29a), 4.64 (s, 1H, H-29b), 1.71 (s, 3H, 30-CH_3_ group), 1.19 (s, 3H), 1.14 (s, 3H), 1.06 (s, 3H), 0.98 (s, 3H), 0.85 (s, 3H, 5 × CH_3_) ppm; ^13^C NMR (126 MHz, CDCl_3_) δ 203.51, 175.89, 153.86, 150.59, 138.41, 136.61, 129.55, 128.66 (2C), 128.46 (2C), 128.27, 126.75, 125.80, 124.93, 109.89, 65.96, 56.68, 52.57, 49.49, 47.04, 45.40, 44.92, 42.87, 41.73, 39.38, 38.61, 37.07, 33.68, 32.22, 30.72, 29.63, 28.95, 25.80, 21.73, 21.61, 19.57, 19.51, 19.46, 16.36, 14.73 ppm; HRMS (ESI^+^): *m*/*z* calcd for C_41_H_53_O_3_S [M + H]^+^ 625.3710, found 625.3715.

##### Benzyl 2-(3-Thiophenyl)-3-oxolupa-1,20(29)-dien-28-oate (**10d**)

Compound **10d** was prepared according to the general procedure from triflate **9** (200 mg; 0.289 mmol) and 3-thiopheneboronic acid (44.4 mg; 0.347 mmol; 1.2 equiv). After the work-up and purification (Hex/EtOAc 10:1), 126 mg (70%) of compound **10d** was obtained as an amorphous solid. IR (DRIFT) ν_max_ 2940, 2866, 1726 (C=O), 1673 (C=C), 1143, 752, 657 cm^–1^; ^1^H NMR (500 MHz, CDCl_3_) δ 7.55 (dd, *J*_1_ = 3.0, *J*_2_ = 1.2 Hz, 1H, H-thiophene), 7.40–7.30 (m, 5H, Ph group), 7.26–7.24 (m, 1H, H-thiophene), 7.24 (s, 1H, H-1), 7.20–7.17 (m, 1H, H-thiophene), 5.17 (d, *J* = 12.3 Hz, 1H, PhCH_2_–a), 5.11 (d, *J* = 12.3 Hz, 1H, PhCH_2_–b), 4.75 (s, 1H, H-29a), 4.63 (s, 1H, H-29b), 3.05 (td, *J*_1_ = 10.9, *J*_2_ = 4.8 Hz, 1H, H-19), 1.70 (s, 3H, 30-CH_3_ group), 1.18 (s, 3H), 1.13 (s, 3H), 1.05 (s, 3H), 0.98 (s, 3H), 0.85 (s, 3H, 5 × CH_3_) ppm; ^13^C NMR (126 MHz, CDCl_3_) δ 204.54, 175.89, 154.69, 150.58, 137.17, 136.61, 130.77, 128.66 (2C), 128.45 (2C), 128.27, 126.89, 124.97, 123.07, 109.89, 65.95, 56.68, 52.67, 49.50, 47.04, 45.55, 45.04, 42.85, 41.69, 39.11, 38.64, 37.08, 33.72, 32.23, 30.71, 29.63, 28.97, 25.83, 21.79, 21.57, 19.60, 19.55, 19.46, 16.35, 14.71 ppm; HRMS (ESI^+^) *m*/*z* calcd for C_41_H_53_O_3_S [M + H]^+^ 625.3710, found 625.3713.

##### Benzyl 2-(5-Chlorthiophene-3-yl)-3-oxolupa-1,20(29)-dien-28-oate (**10e**)

Compound **10e** was prepared according to the general procedure from triflate **9** (200 mg; 0.289 mmol) and 5-chlorothiophene-2-boronic acid (70.5 mg; 0.347 mmol; 1.2 equiv). After the work-up and purification (Hex/EtOAc 12:1), 132 mg (69%) of compound **10e** was obtained as an amorphous solid. IR (DRIFT) ν_max_ 2942, 2868, 1723, 1668, 1453, 1149, 1124, 752, 697 cm^–1^; ^1^H NMR (500 MHz, CDCl_3_) δ 7.40–7.30 (m, 5H, Ph group), 7.27 (s, 1H, H-1), 7.02 (d, *J* = 4.0 Hz, 1H, H-chlorothiophene), 6.78 (d, *J* = 4.0 Hz, 1H, H-chlorothiophene), 5.17 (d, *J* = 12.3 Hz, 1H, PhCH_2_–a), 5.10 (d, *J* = 12.3 Hz, 1H, PhCH_2_–b), 4.76 (s, 1H, H-29a), 4.64 (s, 1H, H-29b), 3.05 (td, *J*_1_ = 10.9, *J*_2_ = 4.7 Hz, 1H, H-19), 1.71 (s, 3H, 30-CH_3_ group), 1.17 (s, 3H), 1.13 (s, 3H), 1.05 (s, 3H), 0.98 (s, 3H), 0.84 (s, 3H, 5 × CH_3_) ppm; ^13^C NMR (126 MHz, CDCl_3_) δ 203.33, 175.87, 153.60, 150.55, 136.60, 136.48, 130.85, 129.04, 128.66 (2C), 128.46 (2C), 128.27, 125.45, 123.38, 109.93, 65.96, 56.65, 52.57, 49.48, 47.04, 45.23, 44.87, 42.89, 41.79, 39.46, 38.58, 37.06, 33.68, 32.21, 30.71, 29.62, 28.88, 25.77, 21.68, 21.60, 19.55, 19.43 (2C), 16.38, 14.73 ppm; HRMS (ESI^+^) *m*/*z* calcd for C_41_H_52_ClO_3_S [M + H]^+^ 659.3320, found 659.3323.

##### Benzyl 2-(3-Pyridinyl)-3-oxolupa-1,20(29)-dien-28-oate (**10g**)

Compound **10g** was prepared according to the general procedure from triflate **9** (200 mg; 0.289 mmol) and 3-pyridineboronic acid (53.3 mg; 0.434 mmol). After the work-up and purification (Hex/EtOAc 2:1), 133 mg (74%) of crystalline compound **10g** was obtained. MP 113–115 °C (Hex/EtOAc); [α]_D_ +17; IR (DRIFT) ν_max_ 2939, 2868, 1722 (C=O), 1670 (C=C), 1124, 713 cm^–1^; ^1^H NMR (500 MHz, CDCl_3_) δ 8.52–8.50 (m, 1H, H-pyridine), 8.50–8.49 (m, 1H, H-pyridine), 7.65–7.61 (m, 1H, H-pyridine), 7.40–7.30 (m, 5H, Ph group), 7.25–7.21 (m, 1H, H-pyridine), 7.20 (s, 1H, H-1), 5.17 (d, *J* = 12.3 Hz, 1H, PhCH_2_–a), 5.11 (d, *J* = 12.3 Hz, 1H, PhCH_2_–b), 4.74 (s, 1H, H-29a), 4.61 (s, 1H, H-29b), 3.04 (td, *J*_1_ = 11.0, *J*_2_ = 4.7 Hz, 1H, H-19), 1.68 (s, 3H, 30-CH_3_ group), 1.19 (s, 3H), 1.18 (s, 3H), 1.10 (s, 3H), 0.98 (s, 3H), 0.86 (s, 3H, 5 × CH_3_ group) ppm; ^13^C NMR (126 MHz, CDCl_3_) δ 204.15, 175.88, 157.61, 150.43, 149.18, 148.76, 136.60, 135.99, 133.45, 133.12, 128.66 (2C), 128.45 (2C), 128.26, 122.86, 109.95, 65.95, 56.64, 53.02, 49.47, 47.03, 45.39, 44.99, 42.85, 41.74, 39.46, 38.59, 37.06, 33.72, 32.21, 30.67, 29.61, 28.73, 25.73, 21.72, 21.56, 19.53, 19.50, 19.41, 16.39, 14.71 ppm; HRMS (ESI^+^): *m*/*z* calcd for C_42_H_54_NO_3_ [M + H]^+^ 620.4098, found 620.4099.

##### Benzyl 2-(4-Pyridinyl)-3-oxolupa-1,20(29)-dien-28-oate (**10h**)

Compound **10h** was prepared according to the general procedure from triflate **9** (400 mg; 0.579 mmol) and 4-pyridineboronic acid (107 mg; 0.869 mmol). After the work-up and purification (Hex/EtOAc 3:2), 251 mg (70%) of crystalline compound **10h** was obtained. MP 184–186 °C (Hex/EtOAc); [α]_D_ +17; IR (DRIFT) ν_max_ 2940, 2865, 1725 (C=O), 1674 (C=C), 1381 (C–N), 776, 745 (Ph) cm^–1^; ^1^H NMR (500 MHz, CDCl_3_) δ 8.58–8.52 (m, 2H, H-pyridine), 7.40–7.30 (m, 5H, Ph group), 7.27 (s, 1H, H-1), 7.25–7.23 (m, 2H, H-pyridine), 5.17 (d, *J* = 12.3 Hz, 1H, PhCH_2_–a), 5.10 (d, *J* = 12.3 Hz, 1H, PhCH_2_–b), 4.74 (s, 1H, H-29a), 4.63 (s, 1H, H-29b), 3.04 (td, *J*_1_ = 11.0, *J*_2_ = 4.6 Hz, 1H, H-19), 1.68 (s, 3H, 30-CH_3_ group), 1.19 (s, 3H), 1.17 (s, 3H), 1.08 (s, 3H), 0.97 (s, 3H), 0.86 (s, 3H, 5 × CH_3_) ppm; ^13^C NMR (126 MHz, CDCl_3_) δ 203.80, 175.86, 158.33, 150.47, 149.69 (2C), 145.02, 136.59, 134.25, 128.66 (2C), 128.45 (2C), 128.27, 123.02 (2C), 109.93, 65.96, 56.63, 52.87, 49.46, 47.02, 45.54, 44.90, 42.86, 41.76, 39.50, 38.59, 37.05, 33.66, 32.19, 30.67, 29.84, 29.60, 28.71, 25.74, 21.78, 21.48, 19.55, 19.51, 19.23, 16.37, 14.69 ppm; HRMS (ESI^+^) *m*/*z* calcd for [M + H]^+^ C_42_H_54_NO_3_ 620.4098, found 620.4102.

##### Benzyl 2-(5-Pyrimidinyl)-3-oxolupa-1,20(29)-dien-28-oate (**10i**)

Compound **10i** was prepared according to the general procedure from triflate **9** (200 mg; 0.289 mmol) and 5-pyrimidineboronic acid (39.9 mg; 0.434 mmol). After the work-up and purification (Hex/EtOAc 3:1), 131 mg (73%) of crystalline compound **10i** was obtained. MP 161–162 °C (Hex/EtOAc); [α]_D_ +28; IR (DRIFT) ν_max_ 2942, 2868, 1722, 1670, 1454, 1149, 1125, 751, 726, 697 cm^–1^; ^1^H NMR (500 MHz, CDCl_3_) δ 9.11 (s, 1H, H-pyrimidine), 8.68 (s, 2H, H-pyrimidine), 7.40–7.30 (m, 5H, Ph group), 7.26 (s, 1H, H-1), 5.17 (d, *J* = 12.3 Hz, 1H, PhCH_2_–a), 5.11 (d, *J* = 12.3 Hz, 1H, PhCH_2_–b), 4.74 (s, 1H, H-29a), 4.61 (s, 1H, H-29b), 3.04 (td, *J*_1_ = 11.0, *J*_2_ = 4.7 Hz, 1H, H-19), 1.68 (s, 3H, 30-CH_3_ group), 1.20 (s, 3H), 1.19 (s, 3H), 1.12 (s, 3H), 0.98 (s, 3H), 0.86 (s, 3H, 5 × CH_3_) ppm; ^13^C NMR (126 MHz, CDCl_3_) δ 203.47, 175.86, 159.00, 157.58, 156.14 (2C), 150.39, 136.59, 131.04, 130.55, 128.66 (2C), 128.46 (2C), 128.27, 109.99, 65.96, 56.62, 53.09, 49.45, 47.02, 45.33, 44.89, 42.88, 41.82, 39.77, 38.54, 37.04, 33.69, 32.19, 30.65, 29.59, 28.64, 25.68, 21.68, 21.61, 19.49, 19.44, 19.40, 16.41, 14.71 ppm; HRMS (ESI^+^) *m/*z calcd for C_41_H_53_N_2_O_3_ [M + H]^+^ 621.4051, found 621.4053.

##### Benzyl 2-(1,4-Benzodioxan)-3-oxolupa-1,20(29)-dien-28-oate (**10j**)

Compound **10j** was prepared according to the general procedure from triflate **9** (200 mg; 0.289 mmol) and 1,4-benzodioxane-6-boronic acid (62.4 mg; 0.347 mmol). After the work-up and purification (Hex/EtOAc 4:1), 129 mg (66%) of compound **10j** was obtained as an amorphous solid. IR (DRIFT) ν_max_ 2941, 2868, 1725 (C = O), 1671 (C = C), 1208, 1144, 1126, 750 cm^–1^; ^1^H NMR (500 MHz, CDCl_3_) δ 7.40–7.34 (m, 5H, Ph group), 7.07 (s, 1H, H-1), 6.82–6.76 (m, 3H, H-arom. ring), 5.17 (d, *J* = 12.3 Hz, 1H, PhCH_2_–a), 5.11 (d, *J* = 12.3 Hz, 1H, PhCH_2_–b), 4.74 (s, 1H, H-29a), 4.61 (s, 1H, H-29b), 4.25–4.21 (m, 4H, H-dioxane), 3.04 (td, *J*_1_ = 11.0, *J*_2_ = 4.7 Hz, 1H, H-19), 1.68 (s, 3H, 30-CH_3_ group), 1.17 (s, 3H), 1.14 (s, 3H), 1.04 (s, 3H), 0.96 (s, 3H), 0.84 (s, 3H, 5 × CH_3_) ppm; ^13^C NMR (126 MHz, CDCl_3_) δ 204.92, 175.96, 155.31, 150.46, 143.31, 143.25, 136.59, 135.64, 130.83, 129.71, 128.65 (2C), 128.43 (2C), 128.26, 127.52, 123.74, 121.49, 117.18, 117.00, 109.93, 65.96, 64.60, 64.47, 56.66, 52.89, 49.48, 47.04, 45.47, 45.06, 42.81, 41.63, 39.08, 38.66, 37.07, 33.74, 32.23, 30.67, 29.62, 28.78, 25.78, 21.76, 21.42, 19.61, 19.47, 19.34, 16.32, 14.68 ppm; HRMS (ESI^+^): *m*/*z* calcd for [M + H]^+^ C_45_H_57_O_5_ 677.4201, found 677.4203.

##### Benzyl 2-(Benzo[b]thien-2-yl)-3-oxolupa-1,20(29)-dien-28-oate (**10k**)

Compound **10k** was prepared according to the general procedure from triflate **9** (200 mg; 0.289 mmol) and benzo[b]thien-2-ylboronic acid (77.3 mg; 0.434 mmol). After the work-up and purification (Hex/EtOAc 12:1), 159 mg (81%) of compound **10k** was obtained as an amorphous solid. IR (DRIFT) ν_max_ 2943, 2868, 1723 (C=O), 1671 (C=C), 1150, 1126, 744 cm^–1^; ^1^H NMR (500 MHz, CDCl_3_) δ 7.78–7.74 (m, 1H), 7.72–7.68 (m, 1H), 7.60–7.58 (m, 1H), 7.40–7.31 (m, 6H, Ph group, H-1), 7.31–7.26 (m, 1H), 5.17 (d, *J* = 12.3 Hz, 1H, PhCH_2_–a), 5.11 (d, *J* = 12.3 Hz, 1H, PhCH_2_–b), 4.77 (s, 1H, H-29a), 4.65 (s, 1H, H-29b), 3.06 (td, *J*_1_ = 10.9, *J*_2_ = 4.8 Hz, 1H, H-19), 1.72 (s, 3H, 30-CH_3_ group), 1.22 (s, 3H), 1.16 (s, 3H), 1.08 (s, 3H), 1.00 (s, 3H), 0.86 (s, 3H, 5 × CH_3_) ppm; ^13^C NMR (126 MHz, CDCl_3_) δ 203.40, 175.89, 155.76, 150.58, 140.20, 139.50, 139.00, 136.61, 129.89, 128.67 (2C), 128.46 (2C), 128.28, 124.53, 124.37, 123.74, 122.55, 122.03, 109.94, 65.97 56.68, 52.45, 49.50, 47.06, 45.69, 44.88, 42.89, 41.76, 39.60, 38.63, 37.08, 33.63, 32.22, 30.72, 29.64, 29.02, 25.82, 21.85, 21.57, 19.60, 19.57, 19.38, 16.35, 14.75 ppm; HRMS (ESI^+^) *m*/*z* calcd for C_45_H_55_O_3_S [M + H]^+^ 675.3866, found 675.3871.

##### Benzyl 2-(4-Isoquinoline)-3-oxolupa-1,20(29)-dien-28-oate (**10l**)

Compound **10l** was prepared according to the general procedure from triflate **9** (200 mg; 0.289 mmol) and isoquinoline-4-boronic acid (75.1 mg; 0.434 mmol). After the work-up and purification (Hex/EtOAc 2:1), 136 mg (70%) of compound **10l** was obtained as an amorphous solid. IR (DRIFT) ν_max_ 2945, 2868, 1725 (C=O), 1668 (C=C), 1126, 748 cm^–1^; ^1^H NMR (500 MHz, CDCl_3_) δ 9.19 (s, 1H), 8.23 (s, 1H), 7.99–7.94 (m, 1H), 7.67–7.61 (m, 1H), 7.60–7.54 (m, 2H), 7.41–7.30 (m, 5H, Ph group), 7.25 (s, 1H, H-1), 5.17 (d, *J* = 12.3 Hz, 1H, PhCH_2_–a), 5.11 (d, *J* = 12.3 Hz, 1H, PhCH_2_–b), 4.69 (s, 1H, H-29a), 4.55 (s, 1H, H-29b), 3.02 (td, *J*_1_ = 11.0, *J*_2_ = 4.7 Hz, 1H, H-19), 1.64 (s, 3H, 30-CH_3_ group), 1.30 (s, 3H), 1.25 (s, 3H), 1.24 (s, 3H), 1.00 (s, 3H), 0.89 (s, 3H, 5 × CH_3_) ppm; ^13^C NMR (126 MHz, CDCl_3_) δ 203.89, 175.90, 160.64, 152.52, 150.33, 143.17, 136.62, 135.07, 133.11, 130.45, 129.65, 128.67 (2C), 128.45 (2C), 128.30, 128.27, 128.05, 127.16, 124.60, 109.95, 65.95, 56.62, 53.58, 49.46, 47.02, 45.44, 44.97, 42.88, 41.83, 39.83, 38.57, 37.05, 33.85, 32.23, 30.64, 29.63, 29.12, 25.63, 21.89, 21.57, 19.84, 19.53, 19.43, 16.46, 14.77 ppm; HRMS (ESI^+^) *m*/*z* calcd for C_46_H_56_NO_3_ [M + H]^+^ 670.4255, found 670.4255.

##### Benzyl 2-(Indol-5-yl)-3-oxolupa-1,20(29)-dien-28-oate (**10m**)

Compound **10m** was prepared according to the general procedure from triflate **9** (200 mg; 0.289 mmol) and indole-5-boronic acid (69.9 mg; 0.434 mmol). After the work-up and purification (Hex/EtOAc 5:1), 123 mg (65%) of compound **10m** was obtained as an amorphous solid. IR (DRIFT) ν_max_ 3400 (N–H), 2941, 2867, 1723 (C=O), 1665 (C=C), 1123, 724 cm^–1^; ^1^H NMR (500 MHz, CDCl_3_) δ 8.14 (s, 1H, indole N–H), 7.57–7.55 (m, 1H, H-indole), 7.41–7.29 (m, 6H), 7.18–7.14 (m, 2H), 7.13–7.08 (m, 1H), 6.53–6.49 (m, 1H), 5.18 (d, *J* = 12.3 Hz, 1H, PhCH_2_–a), 5.11 (d, *J* = 12.3 Hz, 1H, PhCH_2_–b), 4.74 (s, 1H, H-29a), 4.61 (s, 1H, H-29b), 3.05 (td, *J*_1_ = 10.9, *J*_2_ = 4.8 Hz, 1H, H-19), 1.68 (s, 3H, 30-CH_3_ group), 1.21 (s, 3H), 1.20 (s, 3H), 1.09 (s, 3H), 0.97 (s, 3H), 0.87 (s, 3H, 5 × CH_3_) ppm; ^13^C NMR (126 MHz, CDCl_3_) δ 205.38, 175.93, 154.99, 150.53, 137.22, 136.62, 135.53, 129.26, 128.66 (2C), 128.45 (2C), 128.26, 128.06, 124.55, 122.76, 120.47, 110.67, 109.89, 103.10, 65.94, 56.68, 52.96, 49.50, 47.05, 45.52, 45.15, 42.83, 41.63, 39.12, 38.69, 37.08, 33.79, 32.24, 30.70, 29.65, 28.89, 25.83, 21.82, 21.50, 19.70, 19.49 (2C), 16.34, 14.70 ppm; HRMS (ESI^+^) *m*/*z* calcd for C_45_H_56_NO_3_ [M + H]^+^ 658.4255, found 658.4260.

##### Benzyl 2-(Indazol-6-yl)-3-oxolupa-1,20(29)-dien-28-oate (**10n**)

Compound **10n** was prepared according to the general procedure from triflate **9** (200 mg; 0.289 mmol) and indazol-6-boronic acid (70.3 mg; 0.434 mmol). After the work-up and purification (Hex/EtOAc 2:1), 144 mg (76%) of compound **10n** was obtained as an amorphous solid. IR (DRIFT) ν_max_ 3344 (N–H), 2942, 2867, 1723 (C=O), 1668 (C=C), 1124, 1149, 734, 697 cm^–1^; ^1^H NMR (500 MHz, CDCl_3_) δ 10.05 (s, 1H, indazole N–H), 8.05–8.01 (m, 1H, H-indazole), 7.71–7.66 (m, 1H, H-indazole), 7.49–7.45 (m, 1H, H-indazole), 7.41–7.30 (m, 5H, Ph group), 7.23 (s, 1H, H-1), 7.09–7.05 (m, 1H, H-indazole), 5.17 (d, *J* = 12.3 Hz, 1H, PhCH_2_–a), 5.11 (d, *J* = 12.3 Hz, 1H, PhCH_2_–b), 4.74 (s, 1H, H-29a), 4.60 (s, 1H, H-29b), 3.04 (td, *J*_1_ = 11.0, *J*_2_ = 4.7 Hz, 1H, H-19), 1.67 (s, 3H, 30-CH_3_ group), 1.21 (s, 3H), 1.20 (s, 3H), 1.10 (s, 3H), 0.98 (s, 3H), 0.87 (s, 3H, 5 × CH_3_) ppm; ^13^C NMR (126 MHz, CDCl_3_) δ 204.92, 175.90, 156.96, 150.52, 140.37, 136.61, 136.37, 136.31, 134.86, 128.66 (2C), 128.45 (2C), 128.27, 122.66, 122.07, 120.50, 109.90, 109.41, 65.96, 56.66, 52.93, 49.48, 47.03, 45.61, 45.07, 42.85, 41.71, 39.35, 38.64, 37.07, 33.73, 32.22, 30.69, 29.63, 28.86, 25.80, 21.82, 21.52, 19.64, 19.51, 19.41, 16.37, 14.71 ppm; HRMS (ESI^+^) *m*/*z* calcd for C_44_H_55_N_2_O_3_ [M + H]^+^ 659.4207, found 659.4211.

#### General Procedures for the Deprotection of Derivatives **10a**–**10n**

(A) The first method for the deprotection of benzyl ester is based on a two-step reaction described in the literature.^31^ In the first step, the benzyl ester moiety is exchanged for a silyl ester. Then, the silyl ester is cleaved by TBAF to form carboxylic acid.

A reaction vial equipped with a stirrer was annealed, cooled with a stream of nitrogen, and covered with a lid with a septum. Benzyl betulonate derivative **10a**–**10n** was dissolved in dry DCE (1–2 mL) and injected into the prepared vial. Then, TBDMSH (4 equiv), TEA (3.2 equiv), and Pd(OAc)_2_ (0.5 equiv) were added. The reaction mixture was stirred at 60 °C under a nitrogen atmosphere and monitored by TLC. After completion, the reaction mixture was filtered through a small amount of silica gel with a Celite pad on the top, washed with MeOH/CHCl_3_ 1:1, and evaporated. The crude product was subsequently dissolved in 1,4-dioxane (2–3 mL) in a round-bottom flask equipped with a stirrer. Then, the required volume of TBAF in THF was added. The reaction mixture was stirred at room temperature and monitored by TLC. After completion, the reaction mixture was extracted with EtOAc, washed with an aqueous solution of NH_4_Cl, an aqueous solution of NaCl, and with water to neutral pH. The organic phase was dried with anhydrous magnesium sulfate, filtered, evaporated, and chromatographed on silica gel.

(B) The second method used for deprotection was reductive debenzylation with cyclohexa-1,3-diene. To a degassed solution of starting benzyl betulonate derivative **10a**–**10n** dissolved in anhydrous EtOH in a reaction vial equipped with a stirrer, cyclohexa-1,3-diene (7 equiv) and Pd/C (10%) were added. The reaction mixture was stirred at 50 °C and monitored by TLC. After the specified time, the solvent was evaporated, and the product was chromatographed on silica gel with a Celite pad on the top.

##### 2-(Furan-2-yl)-3-oxolupa-1,20(29)-dien-28-oic acid (**11a**)

Compound **11a** was prepared according to general procedure A from benzyl betulonate **10a** (86 mg; 0.141 mmol). After 24 h, 2 equivalents of TBDMSH and 1.6 equivalents of TEA were added, as traces of the starting compound were still unreacted. After 28 h, standard work-up was done, the solvent was evaporated, the crude product was dissolved in 1,4-dioxane (3 mL), and TBAF in THF (40 μL) was added. After the work-up and purification on a column of silica gel (Hex/EtOAc 2:1 + 1% THF + 0.5% CH_3_COOH; note, this complex mixture was developed due to the low solubility of the product in standard elution mixtures), 24 mg (33%) of compound **11a** was obtained as an amorphous solid. IR (DRIFT) ν_max_ 2940, 1691, 1454, 1399, 885, 816, 730 cm^–1^; ^1^H NMR (500 MHz, CDCl_3_) δ 7.50 (s, 1H, H-1), 7.34–7.31 (m, 1H, H-furan), 6.84–6.81 (m, 1H, H-furan), 6.42–6.38 (m, 1H, H-furan), 4.78 (s, 1H, H-29a), 4.65 (s, 1H, H-29b), 3.04 (td, *J*_1_ = 10.7, *J*_2_ = 4.8 Hz, 1H, H-19), 1.72 (s, 3H, 30-CH_3_ group), 1.17 (s, 3H), 1.14 (s, 3H), 1.08 (s, 3H), 1.05 (s, 3H), 1.01 (s, 3H, 5 × CH_3_) ppm; ^13^C NMR (126 MHz, CDCl_3_) δ 202.66, 181.53, 152.32, 150.36, 149.50, 141.33, 126.01, 111.74, 110.08, 109.59, 56.52, 52.54, 49.33, 47.04, 45.48, 44.99, 42.92, 41.78, 38.95, 38.90, 37.21, 33.76, 32.27, 30.69, 29.85, 29.79, 28.87, 25.77, 21.73, 21.65, 19.56, 19.53, 16.56, 14.76 ppm; HRMS (ESI^+^) *m*/*z* calcd for C_34_H_47_O_4_ [M + H]^+^ 519.3469, found 519.3470.

##### 2-(Furan-3-yl)-3-oxolupa-1,20(29)-dien-28-oic acid (**11b**)

Compound **11b** was prepared according to general procedure A from benzyl betulonate **10b** (64 mg; 0.105 mmol). In the second step, 3 mL of 1,4-dioxane and 40 μL of TBAF in THF were added. After the work up and purification (Hex/EtOAc 6:1 + 0.5% CH_3_COOH), 29 mg (53%) of compound **11b** was obtained as an amorphous solid. IR (DRIFT) ν_max_ 2932, 2867, 1690, 1674, 1451, 1188, 884, 797, 754 cm^–1^; ^1^H NMR (500 MHz, CDCl_3_) δ 7.97–7.95 (m, 1H, H-furan), 7.38–7.36 (m, 1H, H-furan), 7.18 (s, 1H, H-1), 6.53–6.49 (m, 1H, H-furan), 4.78 (s, 1H, H-29a), 4.66 (s, 1H, H-29b), 3.04 (td, *J*_1_ = 10.8, *J*_2_ = 4.8 Hz, 1H, H-19), 1.73 (s, 3H, 30-CH_3_ group), 1.17 (s, 3H), 1.13 (s, 3H), 1.07 (s, 3H), 1.04 (s, 3H), 1.02 (s, 3H, 5 × CH_3_) ppm; ^13^C NMR (126 MHz, CDCl_3_) δ 204.12, 180.74, 153.42, 150.43, 142.49, 141.79, 127.61, 120.78, 110.03, 108.17, 56.47, 52.64, 49.31, 47.01, 45.34, 44.97, 42.93, 41.76, 39.13, 38.83, 37.19, 33.78, 32.25, 30.69, 29.85, 29.78, 28.92, 25.77, 21.68, 19.65, 19.56, 19.49, 16.56, 14.77 ppm; HRMS (ESI^+^) *m*/*z* calcd for C_34_H_47_O_4_ [M + H]^+^ 519.3469, found 519.3475.

##### 2-(Pyridin-3-yl)-3-oxolupa-1,20(29)-dien-28-oic acid (**11g**)

Compound **11g** was prepared according to general procedure A from benzyl betulonate **10g** (50 mg; 0.081 mmol). In the second step, 2 mL of 1,4-dioxane and 40 μL of TBAF in THF were added. After the work-up and purification (Hex/EtOAc 1:1 + 0.5% CH_3_COOH), 14 mg (42%) of crystalline compound **11g** was obtained. MP 204–206 °C (Hex/EtOAc); [α]_D_ +35; IR (DRIFT) ν_max_ 2936, 2868, 1698, 1660, 1454, 1185, 912, 726 cm^–1^; ^1^H NMR (500 MHz, CDCl_3_) δ 8.56–8.49 (m, 2H, H-pyridine), 7.70–7.64 (m, 1H, H-pyridine), 7.29–7.24 (m, 1H, H-pyridine), 7.23 (s, 1H, H-1), 4.76 (s, 1H, H-29a), 4.62 (s, 1H, H-29b), 3.05 (td, *J*_1_ = 10.8, *J*_2_ = 4.9 Hz, 1H, H-19), 1.70 (s, 3H, 30-CH_3_ group), 1.19 (s, 3H), 1.19 (s, 3H), 1.13 (s, 3H), 1.07 (s, 3H), 1.02 (s, 3H, 5 × CH_3_) ppm; ^13^C NMR (126 MHz, CDCl_3_) δ 204.10, 180.39, 157.72, 150.38, 148.72, 148.35, 136.42, 133.38, 133.30, 123.04, 110.04, 56.44, 53.03, 49.30, 47.03, 45.42, 44.98, 42.94, 41.83, 39.53, 38.79, 37.24, 33.77, 32.34, 30.69, 29.80, 28.75, 25.72, 21.74, 21.57, 19.54, 19.51, 19.45, 16.61, 14.76 ppm; HRMS (ESI^+^) *m*/*z* calcd for C_35_H_48_NO_3_ [M + H]^+^ 530.3629, found 530.3635.

##### 2-(Pyridin-4-yl)-3-oxolupa-1,20(29)-dien-28-oic acid (**11h**)

Compound **11h** was prepared according to general procedure A from benzyl betulonate **10h** (50 mg; 0.081 mmol). In the second step, 2 mL of 1,4-dioxane and 40 μL of TBAF in THF were added. After the work-up and purification (Hex/EtOAc 1:1 + 0.5% CH_3_COOH), 24 mg (56%) of compound **11h** was obtained as an amorphous solid. IR (DRIFT) ν_max_ 2933, 2867, 1673, 1603, 1452, 1382, 1180, 883, 833, 749 cm^–1^; ^1^H NMR (500 MHz, CDCl_3_) δ 8.63–8.51 (m, 2H, H-pyridine), 7.33–7.27 (m, 3H), 4.76 (s, 1H, H-29a), 4.63 (s, 1H, H-29b), 3.04 (td, *J*_1_ = 10.9, *J*_2_ = 4.8 Hz, 1H, H-19), 1.70 (s, 3H, 30-CH_3_ group), 1.20 (s, 3H), 1.18 (s, 3H), 1.11 (s, 3H), 1.06 (s, 3H), 1.02 (s, 3H, 5 × CH_3_) ppm; ^13^C NMR (126 MHz, CDCl_3_) δ 203.72, 180.14, 158.52, 150.41, 149.11, 145.56, 134.19, 123.23, 110.03, 56.42, 52.88, 49.28, 47.00, 45.58, 44.89, 42.94, 41.84, 39.59, 38.77, 37.21, 33.70, 32.29, 30.67, 29.85, 29.78, 28.72, 25.73, 21.80, 21.49, 19.56, 19.52, 19.26, 16.59, 14.74 ppm; HRMS (ESI^+^) *m*/*z* calcd for C_35_H_48_NO_3_ [M + H]^+^ 530.3629, found 530.3633.

##### 2-(Pyrimidin-5-yl)-3-oxolupa-1,20(29)-dien-28-oic acid (**11i**)

Compound **11i** was prepared according to general procedure A from benzyl betulonate **10i** (50 mg; 0.0805 mmol). In the second step, 2 mL of 1,4-dioxane and 50 μL of TBAF in THF were added. After the work-up and purification (Hex/EtOAc 1:1 + 0.5% HCOOH), 19 mg (44%) of compound **11i** was obtained as an amorphous solid. IR (DRIFT) ν_max_ 2950, 2923, 2853, 1705, 1672, 1452, 1180, 755, 728 cm^–1^; ^1^H NMR (500 MHz, CDCl_3_) δ 9.13 (s, 1H, H-pyrimidine), 8.70 (s, 2H, H-pyrimidine), 7.29 (s, 1H, H-1), 4.76 (s, 1H, H-29a), 4.63 (s, 1H, H-29b), 3.04 (td, *J*_1_ = 10.8, *J*_2_ = 4.9 Hz, 1H, H-19), 1.70 (s, 3H, 30-CH_3_ group), 1.20 (s, 6H), 1.15 (s, 3H), 1.07 (s, 3H), 1.03 (s, 3H, 5 × CH_3_) ppm; ^13^C NMR (126 MHz, CDCl_3_) δ 203.40, 181.24, 158.99, 157.44, 156.14, 150.26, 131.09, 130.55, 110.11, 56.45, 53.08, 49.25, 47.02, 45.33, 44.86, 42.95, 41.88, 39.81, 38.76, 37.22, 33.73, 32.28, 30.65, 29.83, 29.76, 28.64, 25.64, 21.67, 21.61, 19.48, 19.42, 16.62, 14.75 ppm; HRMS (ESI^+^) *m*/*z* calcd for C_34_H_47_N_2_O_3_ [M + H]^+^ 531.3581, found 531.3588.

##### 2-(1,4-Benzodioxan)-3-oxolupa-1,20(29)-dien-28-oic acid (**11j**)

Compound **11j** was prepared according to general procedure A from benzyl betulonate **10j** (72 mg; 0.106 mmol). In the second step, 2 mL of 1,4-dioxane and 40 μL of TBAF in THF were added. After the work-up and purification (Hex/EtOAc 2:1 + 1% THF + 0.5% CH_3_COOH), 24 mg (38%) of compound **11j** was obtained as an amorphous solid. IR (DRIFT) ν_max_ 2930, 2869, 1692, 1669, 1506, 1455, 1281, 1069, 886, 752 cm^–1^; ^1^H NMR (500 MHz, CDCl_3_) δ 7.08 (s, 1H, H-1), 6.83–6.77 (m, 3H, H-arom. ring), 4.76 (s, 1H, H-29a), 4.63 (s, 1H, H-29b), 4.26–4.22 (m, 4H, H-dioxane), 3.03 (td, *J*_1_ = 10.7, *J*_2_ = 5.0 Hz, 1H, H-19), 1.70 (s, 3H, 30-CH_3_ group), 1.17 (s, 3H), 1.15 (s, 3H), 1.06 (s, 3H), 1.04 (s, 3H), 1.00 (s, 3H, 5 × CH_3_) ppm; ^13^C NMR (126 MHz, CDCl_3_) δ 204.70, 181.47, 155.07, 150.28, 143.34, 143.27, 135.69, 130.84, 121.50, 117.19, 117.02, 110.09, 64.62, 64.49, 56.49, 52.91, 49.30, 47.02, 45.47, 45.06, 42.88, 41.69, 39.10, 38.89, 37.20, 33.78, 32.27, 30.65, 29.77, 28.80, 25.75, 21.76, 21.43, 19.61, 19.48, 19.37, 16.52, 14.74 ppm; HRMS (ESI^+^) *m*/*z* calcd for C_38_H_52_O_5_ [M + H]^+^ 587.3731, found 587.3731.

##### 2-(Isoquinoline)-3-oxolupa-1,20(29)-dien-28-oic acid (**11l**)

Compound **11l** was prepared according to general procedure A from benzyl betulonate **10l** (55 mg; 0.0821 mmol). In the second step, 2 mL of 1,4-dioxane and 40 μL of TBAF in THF were added. After the work-up and purification (Hex/EtOAc 1:1 + 0.5% CH_3_COOH), 40 mg (83%) of compound **11l** was obtained as an amorphous solid. IR (DRIFT) ν_max_ 2949, 2923, 2867, 1903, 1702, 1662, 1458, 1182, 874, 775 cm^–1^; ^1^H NMR (500 MHz, CDCl_3_) δ 9.23 (s, 1H, H-isoquinoline), 8.25 (s, 1H, H-isoquinoline), 8.01–7.97 (m, 1H, H-isoquinoline), 7.69–7.64 (m, 1H, H-isoquinoline), 7.62–7.56 (m, 2H, H-isoquinoline), 7.28 (s, 1H, H-1), 4.73 (s, 1H, H-29a), 4.57 (s, 1H, H-29b), 3.04 (td, *J*_1_ = 11.0, *J*_2_ = 5.0 Hz, 1H, H-19), 1.66 (s, 3H, 30-CH_3_ group), 1.31 (s, 3H), 1.29 (s, 3H), 1.25 (s, 3H), 1.11 (s, 3H), 1.05 (s, 3H, 5 × CH_3_) ppm; ^13^C NMR (126 MHz, CDCl_3_) δ 203.84, 180.48, 160.69, 152.24, 150.34, 142.59, 135.23, 133.07, 130.68, 129.91, 128.32, 128.19, 127.30, 124.63, 110.00, 56.44, 53.60, 49.30, 47.03, 45.46, 44.97, 42.98, 41.92, 39.89, 38.76, 37.26, 33.91, 32.40, 30.68, 29.83, 29.13, 25.63, 21.90, 21.60, 19.87, 19.55, 19.45, 16.71, 14.82 ppm; HRMS (ESI+) *m*/*z* calcd for C_39_H_50_NO_3_ [M + H]^+^ 580.3785, found 580.3792.

##### 2-(Indazol-6-yl)-3-oxolupa-1,20(29)-dien-28-oic acid (**11n**)

Compound **11n** was prepared according to general procedure B from benzyl betulonate **10n** (61 mg; 0.093 mmol) dissolved in dry EtOH (2.0 mL). Cyclohexa-1,3-diene (79 μL; 0.651 mmol) and Pd/C (15.0 mg) were added. After the standard work-up and purification (Hex/EtOAc 3:1 + 0.5% CH_3_COOH to Hex/EtOAc 1:1 + 0.5% CH_3_COOH), 39 mg (65%) of compound **11n** was obtained as an amorphous solid. IR (DRIFT) ν_max_ 2932, 2868, 1670, 1452, 1184, 944, 752, 668 cm^–1^; ^1^H NMR (500 MHz, CDCl_3_) δ 8.05–8.02 (m, 1H, H-indazole), 7.72–7.67 (m, 1H, H-indazole), 7.55–7.51 (m, 1H, H-indazole), 7.30 (s, 1H, H-1), 7.09–7.04 (m, 1H, H-indazole), 4.79 (s, 1H, H-29a), 4.63 (s, 1H, H-29b), 3.08 (td, *J*_1_ = 10.9, *J*_2_ = 4.8 Hz, 1H, H-19), 1.71 (s, 3H, 30-CH_3_ group), 1.22 (s, 3H), 1.21 (s, 3H), 1.13 (s, 3H), 1.06 (s, 3H), 1.02 (s, 3H, 5 × CH_3_) ppm; ^13^C NMR (126 MHz, CDCl_3_) δ 204.93, 180.59, 156.91, 150.52, 140.02, 136.89, 136.68, 133.90, 122.73, 122.47, 120.54, 109.98, 109.51, 56.47, 52.90, 49.25, 47.01, 45.61, 45.09, 42.93, 41.78, 39.40, 38.68, 37.27, 33.75, 32.35, 30.66, 29.85, 28.95, 25.85, 21.96, 21.50, 19.68, 19.57, 19.52, 16.60, 14.71 ppm; HRMS (ESI^+^) *m*/*z* calcd for C_37_H_49_N_2_O_3_ [M + H]^+^ 569.3738, found 569.3742.

#### Cell Culture and MTS Cytotoxicity Assay

The human prostate tumor-derived cell line DU145 was obtained from ATCC (American Type Culture Collection) and cultured in RPMI 1640 (Roswell Park Memorial Institute) medium supplemented with 10% (v/v) fetal bovine serum (FBS). Human prostate tumor-derived LNCaP cells were obtained from ATCC and cultured in RPMI 1640 medium supplemented with 10% FBS. The human nonsmall cell lung carcinoma (NSCLC)-derived cell line HOP-62 was obtained from the DSMZ (German Collection of Microorganisms and Cell Cultures) and cultured in RPMI 1640 medium supplemented with 10% FBS. The human nonsmall cell lung carcinoma-derived cell line NCI-H322 was obtained from ECACC (European Collection of Authenticated Cell Cultures) and cultured in RPMI 1640 medium supplemented with 10% FBS. The human nonsmall cell lung carcinoma-derived cell line NCI-H522 was obtained from ATCC and cultured in RPMI 1640 medium supplemented with 10% FBS. The human lung tumor-derived cell line A549 was obtained from ATCC and cultured in Dulbecco’s Modified Eagle’s Medium (DMEM) supplemented with 10% FBS. The human medulloblastoma-derived cell line DAOY was obtained from ATCC and cultured in Eagle’s Minimum Essential Medium (EMEM) supplemented with 10% FBS. The human glioblastoma-derived cell line T98G was obtained from ATCC and cultured in EMEM medium supplemented with 10% FBS, 1 mmol/L sodium pyruvate, and 1 mmol/L nonessential amino acid mixture. All the above-mentioned cell lines were cultured at 37 °C in an atmosphere of 5% CO_2_ with 95% humidity. The cytotoxicity MTS assays were performed using MTS (3-(4,5-dimethylthiazol-2-yl)-5-(3-carboxymethoxyphenyl)-2-(4-sulfophenyl)-2H-tetrazolium) tetrazolium salt. Briefly, cells were seeded in a 384-well transparent plate in a volume of 25 μL and a concentration of 15,000 cells/mL (DU145, LNCaP, HOP-62, A549, DAOY, T98G) or 30,000 cells/mL (NCI-H322, NCI-H522). The next day, cells were exposed to compounds in a concentration range of 50–0.0975 μmol/L (dilution factor 2) and cultured in the presence of the compounds for 72 h. After 72 h, 5 μL of MTS/phenazine methosulfate solution was pipetted into each well and incubation continued for 1–4 h. Finally, the absorbance at 490 nm was measured using an EnVision multifunctional modular reader (PerkinElmer). The IC_50_ value was determined using Dotmatics software.

#### Reporter Assays

The U-87 MG-Gli-FLuc reporter cell line was developed from the human glioblastoma line U-87 MG obtained from ATCC and cultured in EMEM medium supplemented with 10% FBS, 1 mmol/L sodium pyruvate, and a 1 mmol/L nonessential amino acid mixture. Briefly, U-87 MG cells were transduced with a commercial lentivector expressing Gli-responsive luciferase and subsequently cultured for several days in the presence of puromycin to obtain a heterogeneous population of cells stably expressing Gli-responsive luciferase. The assay was performed in a 96-well white, opaque microplate. U-87 MG-Gli-FLuc cells were transferred to Opti-MEM media with a reduced content of 0.5% (v/v) FBS and plated on a panel in a volume of 100 μL suspension per well and a concentration of 8 × 10^4^/ml. The next day, cells were treated with compounds in a concentration range of 12.5–0.78 μmol/L (dilution factor 2) and cultivation continued for another 24 h. Control cells were treated with DMSO at a concentration of 0.1% (v/v) as a negative control and GANT-61, a specific inhibitor of Gli1 and Gli2, as a positive control. After 24 h, the panels were brought to room temperature for 30 min, and then 100 μL of Britelite Plus reagent (PerkinElmer) was added to each well. Panels were placed on a shaker to achieve complete cell lysis, and luminescence was measured after 3 min using an EnVision plate reader.

Gli Reporter-NIH3T3 (BPS Bioscience) cells were cultured in DMEM medium supplemented with 10% iron-supplemented bovine calf serum and under the selection pressure of Geneticin (500 μg/mL) at 37 °C in an atmosphere of 5% CO_2_ with 95% humidity. The Gli Reporter-NIH3T3 cell line expresses the firefly luciferase (FLuc) gene under the control of a Gli responsive element stably integrated into NIH3T3 cells. The cell line was validated through response to mSHH stimulation and the effect of commercially available Hh inhibitors (Cyclopamine). The assay was performed in 384-well white opaque microplates. Gli Reporter-NIH3T3 cells were transferred to Opti-MEM medium with a reduced content of 0.5% (v/v) iron-supplemented bovine calf serum, supplemented with a 1% solution of nonessential amino acids, 1 mmol/L sodium pyruvate, and 10 mmol/L HEPES, and seeded on the panel in a volume of 25 μL of cell suspension per well at a concentration of 2.5 × 10^5^/mL. The following day, compounds were transferred at the concentration range of 16–0.25 μmol/L (dilution factor 2) and incubated for 2 h. Then, the mSHH ligand at concentrations of 0.25 and 0.5 μg/mL and SAG (a synthetic SMO agonist) at concentrations of 2 and 4 nmol/L were added to the wells. Controls representing DMSO-treated cells were also included at a concentration of 0.1% (v/v). Cells were incubated for another 24–30 h and then kept for 30 min at room temperature, and finally, 25 μL of Britelite plus reagent was added to each well. Plates were placed on a shaker to achieve complete cell lysis, and luminescence was measured after 3 min on an EnVision plate reader.

#### Western Blot

Cells were washed with PBS and lysed on ice in 200 μL of RIPA (Radioimmunoprecipitation Assay) buffer for 30 min. Then, cell lysates were clarified by centrifugation (15000 rpm, 20 min, 4 °C), and the protein concentration in the collected supernatants was determined using a BCA method (Bicinchoninic Acid test). Supernatants were mixed with 4× concentrated Laemmli SDS buffer and boiled for 5 min at 95 °C. 40 μg of total protein was loaded on a 10% polyacrylamide gel. The proteins were then blotted off the gel onto a nitrocellulose membrane. The membrane was blocked for 2 h in a 5% BSA (bovine serum albumin) solution in TBS/T (Tris-buffered saline with 0.1% (v/v) Tween 20). Membranes were then incubated in the presence of specific primary antibodies against Gli1, Cyclin D1, N-MYC, Bcl-2, and GAPDH (all Abcam) diluted 1:1000 in TBS/T overnight at 4 °C. The following day, the membranes were washed 4× for 5 min in TBS/T solution and incubated with rabbit secondary antibody diluted 1:10000 in TBS/T solution for 1 h at room temperature. After the incubation period, the membranes were washed 4 times for 5 min in TBS/T solution and incubated for 5 min in the presence of chemiluminescence reagent ECL Prime (Amersham). Luminescence signal detection was performed by using an Odyssey FC instrument (LI-COR Biosciences).

## Supporting Information Available

The Supporting Information file contains characterization spectral data for all new compounds (pictures of ^1^H NMR, ^13^C NMR, and HRMS spectra) (PDF)
