# Supplementary material for: Novel C-2 Aromatic Heterocycle-Substituted Triterpenoids Inhibit Hedgehog Signaling in GLI1 Overexpression Cancer Cells
Source: ACS Omega. 2025 Mar 4;10(10):10617–32. doi: 10.1021/acsomega.4c11479 (PMC11923649; doi:10.1021/acsomega.4c11479)
Supplement: Supplementary file 1 — ao4c11479_si_001.pdf [file ao4c11479_si_001.pdf]

Novel C-2 aromatic heterocycle-substituted triterpenoids inhibit Hedgehog signaling in GLI1 overexpression cancer cells

Ivo Frydrych,[†,1] Barbora Choma,[‡,1] Lucie Slavíková,[‡] Jan Pokorný,[‡,§] Nikola Jakubcová,[‡,§] Sandra Ludha,[‡] Soňa Gurská,[†] Jiří Řehulka,[†] Barbora Lišková,[†] Petr Džubák,[†,⊥] Marián Hajdúch,[*,†,⊥] Milan Urban[*‡,§]

[†]Institute of Molecular and Translational Medicine, Faculty of Medicine and Dentistry, Palacký University Olomouc and University Hospital Olomouc, Hněvotínská 1333/5, 779 00 Olomouc, Czech Republic.

[‡]Department of Organic Chemistry, Faculty of Science, Palacký University Olomouc, 17. listopadu 1192/12, 771 46 Olomouc, Czech Republic.

[§]Laboratory of Medicinal and Organic Chemistry, Institute of Molecular and Translational Medicine, Faculty of Medicine and Dentistry, Palacký University Olomouc, Hněvotínská 1333/5, 779 00 Olomouc, Czech Republic.

[⊥]Laboratory of Experimental Medicine, Institute of Molecular and Translational Medicine, Czech Advanced Technology and Research Institute, Palacký University Olomouc, Šlechtitelů 241/27, Olomouc-Holice, 783 71 Czech Republic

*E-mail: milan.urban@upol.cz, phone: +420 585 632 197; marian.hajduch@upol.cz

[1]These authors contributed equally

Contents

Figure S1. ^{1}H NMR spectrum of the compound **4a** ($CDCl_3$, 500 MHz)....5

Figure S2. ^{13}C NMR spectrum of the compound **4a** ($CDCl_3$, 126 MHz). 5

Figure S3. ^{1}H NMR spectrum of the compound **4b** ($CDCl_3$, 500 MHz). 6

Figure S4. ^{13}C NMR spectrum of the compound **4b** ($CDCl_3$, 126 MHz). 6

Figure S5. ^{1}H NMR spectrum of the compound **4c** ($CDCl_3$, 500 MHz)....7

Figure S6. ^{13}C NMR spectrum of the compound **4c** ($CDCl_3$, 126 MHz)....7

Figure S7. ^{1}H NMR spectrum of the compound **4d** ($CDCl_3$, 500 MHz). 8

Figure S8. ^{13}C NMR spectrum of the compound **4d** ($CDCl_3$, 126 MHz). 8

Figure S9. ^{1}H NMR spectrum of the compound **4e** ($CDCl_3$, 500 MHz)....9

Figure S10. ^{13}C NMR spectrum of the compound **4e** ($CDCl_3$, 126 MHz)....9

Figure S11. ^{1}H NMR spectrum of the compound **4g** ($CDCl_3$, 500 MHz)....10

Figure S12. ^{13}C NMR spectrum of the compound **4g** ($CDCl_3$, 126 MHz). 10

Figure S13. ^{1}H NMR spectrum of the compound **4h** ($CDCl_3$, 500 MHz). 11

Figure S14. ^{13}C NMR spectrum of the compound **4h** ($CDCl_3$, 126 MHz). 11

Figure S15. ^{1}H NMR spectrum of the compound **4i** ($CDCl_3$, 500 MHz). 12

Figure S16. ^{13}C NMR spectrum of the compound **4i** ($CDCl_3$, 126 MHz). 12

Figure S17. ^{1}H NMR spectrum of the compound **4j** ($CDCl_3$, 500 MHz). 13

Figure S18. ^{13}C NMR spectrum of the compound **4j** ($CDCl_3$, 126 MHz). 13

Figure S19. ^{1}H NMR spectrum of the compound **4k** ($CDCl_3$, 500 MHz). 14

Figure S20. ^{13}C NMR spectrum of the compound **4k** ($CDCl_3$, 126 MHz). 14

Figure S21. ^{1}H NMR spectrum of the compound **4l** ($CDCl_3$, 500 MHz). 15

Figure S22. ^{13}C NMR spectrum of the compound **4l** ($CDCl_3$, 126 MHz). 15

Figure S23. ^{1}H NMR spectrum of the compound **4m** ($CDCl_3$, 500 MHz). 16
Figure S24. ^{13}C NMR spectrum of the compound **4m** ($CDCl_3$, 126 MHz). 16
Figure S25. ^{1}H NMR spectrum of the compound **4n** ($CDCl_3$, 500 MHz). 17
Figure S26. ^{13}C NMR spectrum of the compound **4n** ($CDCl_3$, 126 MHz). 17
Figure S27. ^{1}H NMR spectrum of the compound **10a** ($CDCl_3$, 500 MHz)..18
Figure S28. ^{13}C NMR spectrum of the compound **10a** ($CDCl_3$, 126 MHz). 18
Figure S29. ^{1}H NMR spectrum of the compound **10b** ($CDCl_3$, 500 MHz). 19
Figure S30. ^{13}C NMR spectrum of the compound **10b** ($CDCl_3$, 126 MHz). 19
Figure S31. ^{1}H NMR spectrum of the compound **10c** ($CDCl_3$, 500 MHz)..20
Figure S32. ^{13}C NMR spectrum of the compound **10c** ($CDCl_3$, 126 MHz)..20
Figure S33. ^{1}H NMR spectrum of the compound **10d** ($CDCl_3$, 500 MHz). 21
Figure S34. ^{13}C NMR spectrum of the compound **10d** ($CDCl_3$, 126 MHz). 21
Figure S35. ^{1}H NMR spectrum of the compound **10e** ($CDCl_3$, 500 MHz)..22
Figure S36. ^{13}C NMR spectrum of the compound **10e** ($CDCl_3$, 126 MHz)..22
Figure S37. ^{1}H NMR spectrum of the compound **10g** ($CDCl_3$, 500 MHz)..23
Figure S38. ^{13}C NMR spectrum of the compound **10g** ($CDCl_3$, 126 MHz). 23
Figure S39. ^{1}H NMR spectrum of the compound **10h** ($CDCl_3$, 500 MHz). 24
Figure S40. ^{13}C NMR spectrum of the compound **10h** ($CDCl_3$, 126 MHz). 24
Figure S41. ^{1}H NMR spectrum of the compound **10i** ($CDCl_3$, 500 MHz). 25
Figure S42. ^{13}C NMR spectrum of the compound **10i** ($CDCl_3$, 126 MHz). 25
Figure S43. ^{1}H NMR spectrum of the compound **10j** ($CDCl_3$, 500 MHz). 26
Figure S44. ^{13}C NMR spectrum of the compound **10j** ($CDCl_3$, 126 MHz). 26
Figure S45. ^{1}H NMR spectrum of the compound **10k** ($CDCl_3$, 500 MHz). 27
Figure S46. ^{13}C NMR spectrum of the compound **10k** ($CDCl_3$, 126 MHz). 27
Figure S47. ^{1}H NMR spectrum of the compound **10l** ($CDCl_3$, 500 MHz). 28
Figure S48. ^{13}C NMR spectrum of the compound **10l** ($CDCl_3$, 126 MHz). 28
Figure S49. ^{1}H NMR spectrum of the compound **10m** ($CDCl_3$, 500 MHz). 29
Figure S50. ^{13}C NMR spectrum of the compound **10m** ($CDCl_3$, 126 MHz). 29
Figure S51. ^{1}H NMR spectrum of the compound **10n** ($CDCl_3$, 500 MHz). 30
Figure S52. ^{13}C NMR spectrum of the compound **10n** ($CDCl_3$, 126 MHz). 30
Figure S53. ^{1}H NMR spectrum of the compound **11a** ($CDCl_3$, 500 MHz)..31
Figure S54. ^{13}C NMR spectrum of the compound **11a** ($CDCl_3$, 126 MHz). 31
Figure S55. ^{1}H NMR spectrum of the compound **11b** ($CDCl_3$, 500 MHz). 32
Figure S56. ^{13}C NMR spectrum of the compound **11b** ($CDCl_3$, 126 MHz). 32
Figure S57. ^{1}H NMR spectrum of the compound **11g** ($CDCl_3$, 500 MHz)..33
Figure S58. ^{13}C NMR spectrum of the compound **11g** ($CDCl_3$, 126 MHz). 33
Figure S59. ^{1}H NMR spectrum of the compound **11h** ($CDCl_3$, 500 MHz). 34
Figure S60. ^{13}C NMR spectrum of the compound **11h** ($CDCl_3$, 126 MHz). 34
Figure S61. ^{1}H NMR spectrum of the compound **11i** ($CDCl_3$, 500 MHz). 35
Figure S62. ^{13}C NMR spectrum of the compound **11i** ($CDCl_3$, 126 MHz). 35
Figure S63. ^{1}H NMR spectrum of the compound **11j** ($CDCl_3$, 500 MHz). 36

Figure S64. ^{13}C NMR spectrum of the compound **11j** ($CDCl_3$, 126 MHz). 36
Figure S65. ^{1}H NMR spectrum of the compound **11l** ($CDCl_3$, 500 MHz). 37
Figure S66. ^{13}C NMR spectrum of the compound **11l** ($CDCl_3$, 126 MHz). 37
Figure S67. ^{1}H NMR spectrum of the compound **11n** ($CDCl_3$, 500 MHz). 38
Figure S68. ^{13}C NMR spectrum of the compound **11n** ($CDCl_3$, 126 MHz). 38
Table S1. Full table of all measured cytotoxic activities in all used cancer cells. 39
Figure S69. HRMS spectrum of the compound **4a**. 40
Figure S70. HRMS spectrum of the compound **4b**. 40
Figure S71. HRMS spectrum of the compound **4c**. 41
Figure S72. HRMS spectrum of the compound **4d**. 41
Figure S73. HRMS spectrum of the compound **4e**. 42
Figure S74. HRMS spectrum of the compound **4g**. 42
Figure S75. HRMS spectrum of the compound **4h**. 43
Figure S76. HRMS spectrum of the compound **4i**. 43
Figure S77. HRMS spectrum of the compound **4j**. 44
Figure S78. HRMS spectrum of the compound **4k**. 44
Figure S79. HRMS spectrum of the compound **4l**. 45
Figure S80. HRMS spectrum of the compound **4m**. 45
Figure S81. HRMS spectrum of the compound **4n**. 46
Figure S82. HRMS spectrum of the compound **10a**. 46
Figure S83. HRMS spectrum of the compound **10b**. 47
Figure S84. HRMS spectrum of the compound **10c**. 47
Figure S85. HRMS spectrum of the compound **10d**. 48
Figure S86. HRMS spectrum of the compound **10e**. 48
Figure S87. HRMS spectrum of the compound **10g**. 49
Figure S88. HRMS spectrum of the compound **10h**. 49
Figure S89. HRMS spectrum of the compound **10i**. 50
Figure S90. HRMS spectrum of the compound **10j**. 50
Figure S91. HRMS spectrum of the compound **10k**. 51
Figure S92. HRMS spectrum of the compound **10l**. 51
Figure S93. HRMS spectrum of the compound **10m**. 52
Figure S94. HRMS spectrum of the compound **10n**. 52
Figure S95. HRMS spectrum of the compound **11a**. 53
Figure S96. HRMS spectrum of the compound **11b**. 53
Figure S97. HRMS spectrum of the compound **11g**. 54
Figure S98. HRMS spectrum of the compound **11h**. 54
Figure S99. HRMS spectrum of the compound **11i**. 55
Figure S100. HRMS spectrum of the compound **11j**. 55
Figure S101. HRMS spectrum of the compound **11l**. 56
Figure S102. HRMS spectrum of the compound **11n**. 56

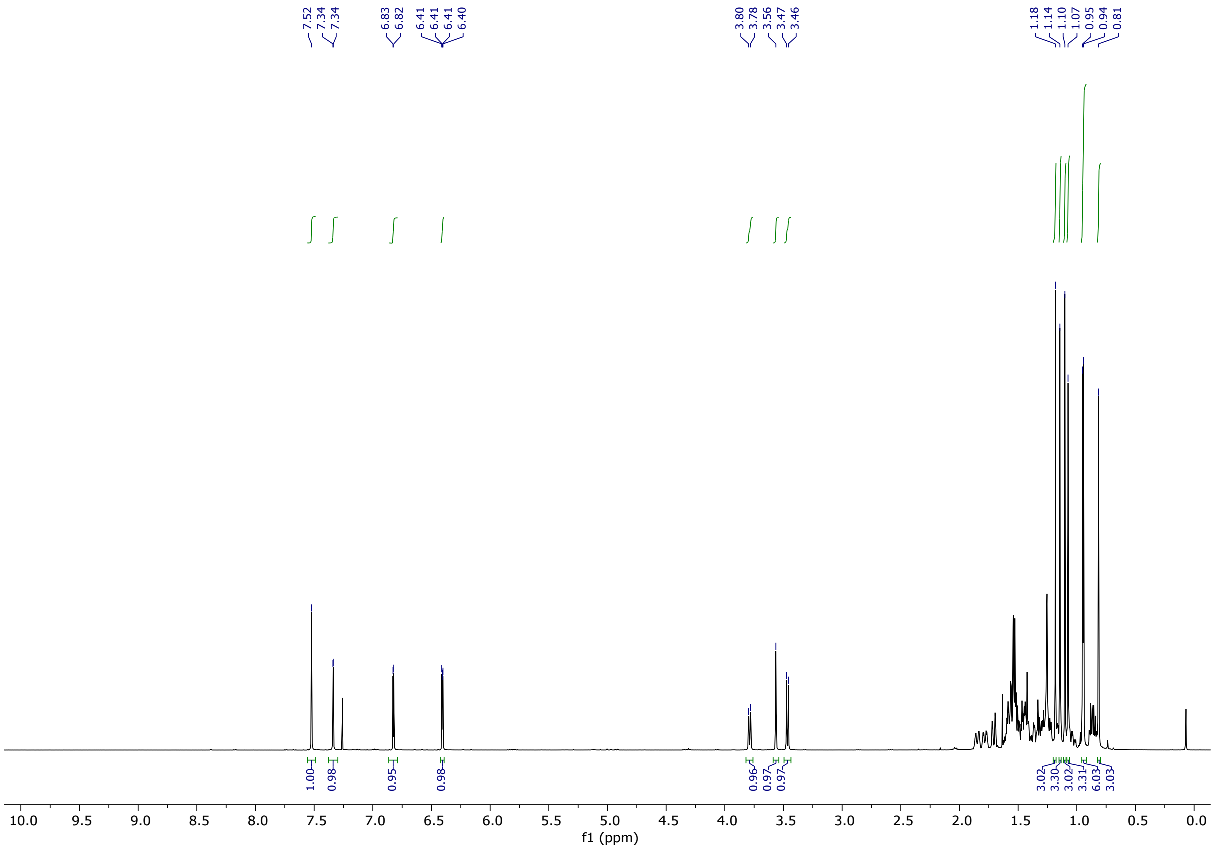

Figure S1. ^{1}H NMR spectrum of the compound **4a** ($CDCl_3$, 500 MHz).

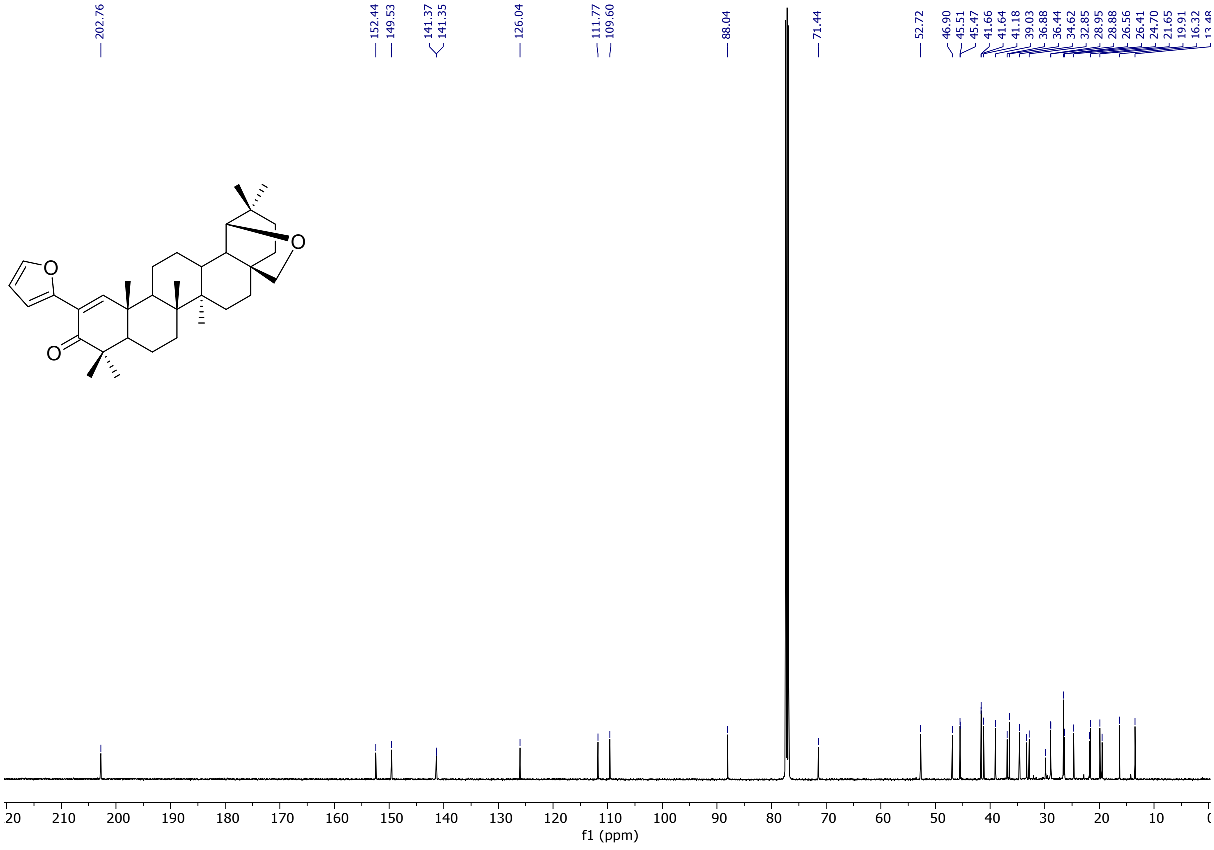

Figure S2. ^{13}C NMR spectrum of the compound **4a** ($CDCl_3$, 126 MHz).

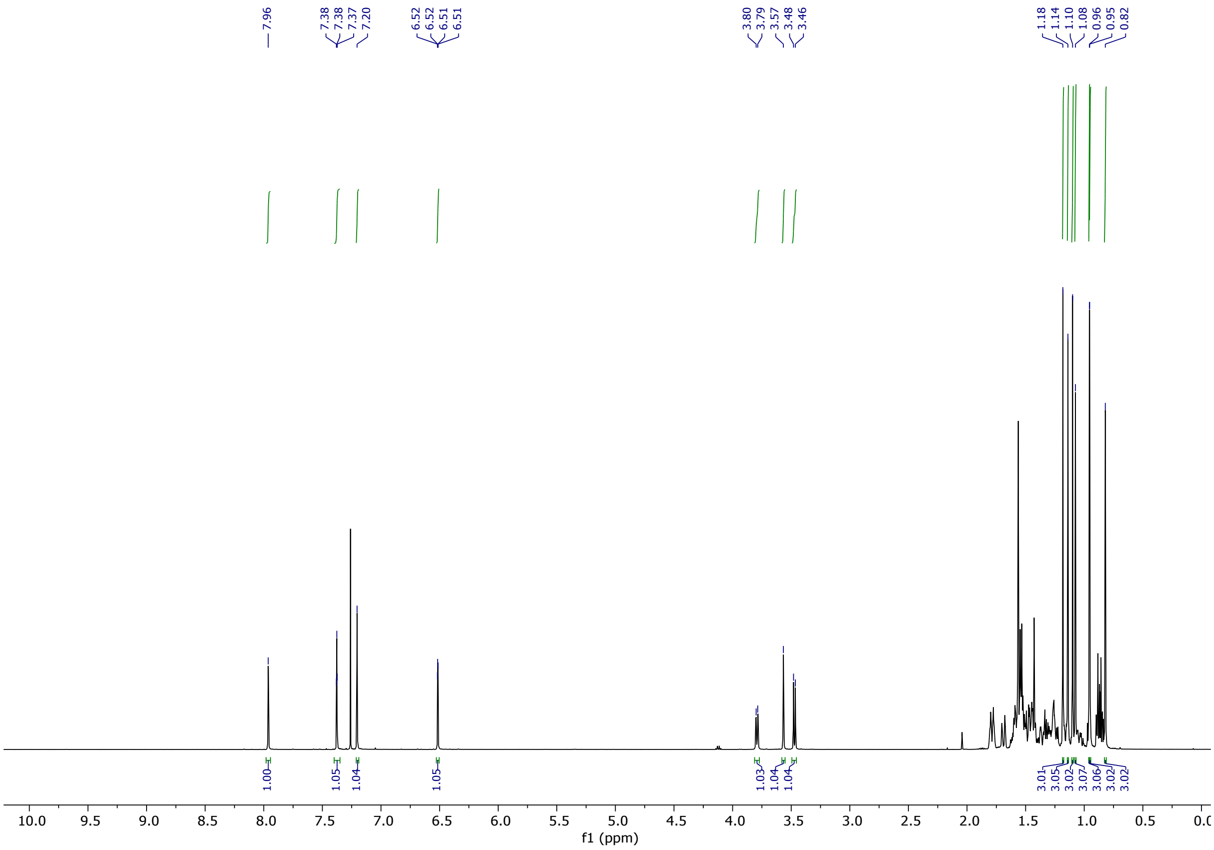

Figure S3. ^{1}H NMR spectrum of the compound **4b** ($CDCl_3$, 500 MHz).

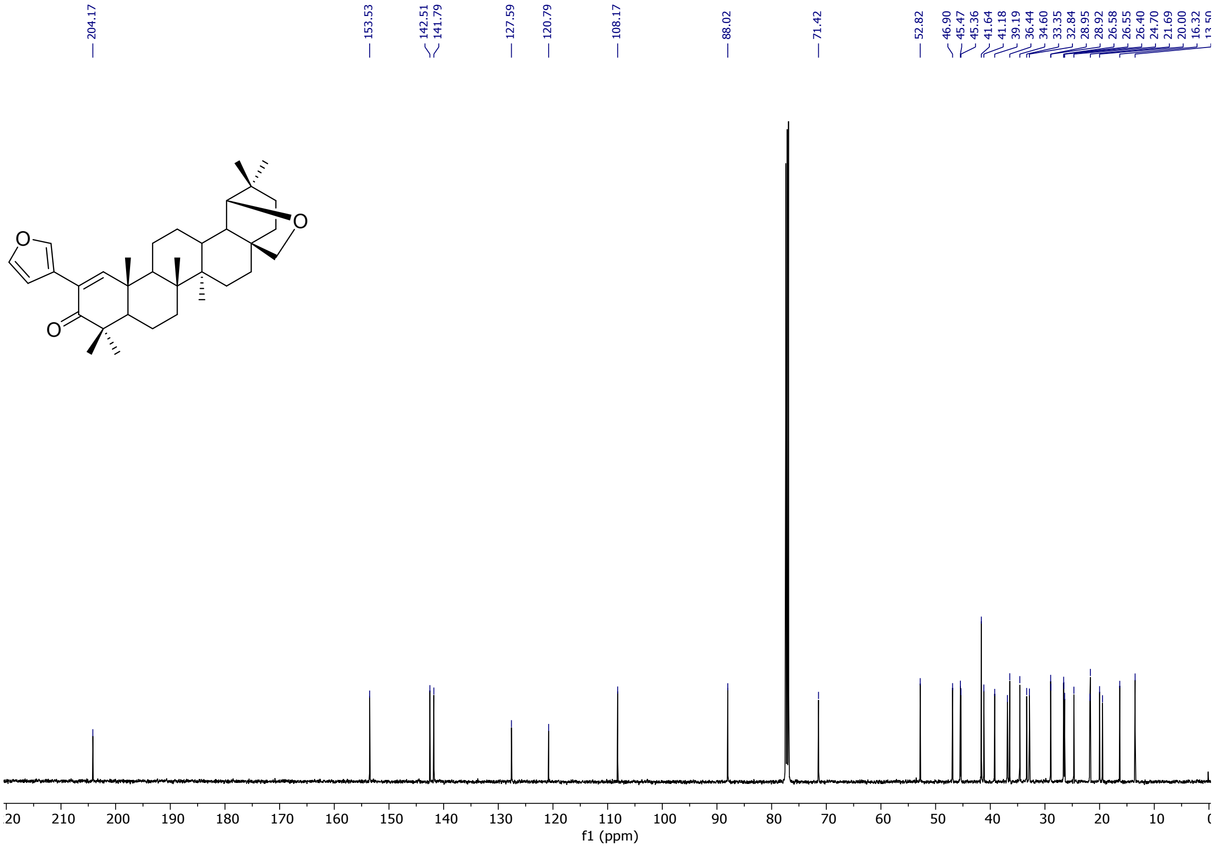

Figure S4. ^{13}C NMR spectrum of the compound **4b** ($CDCl_3$, 126 MHz).

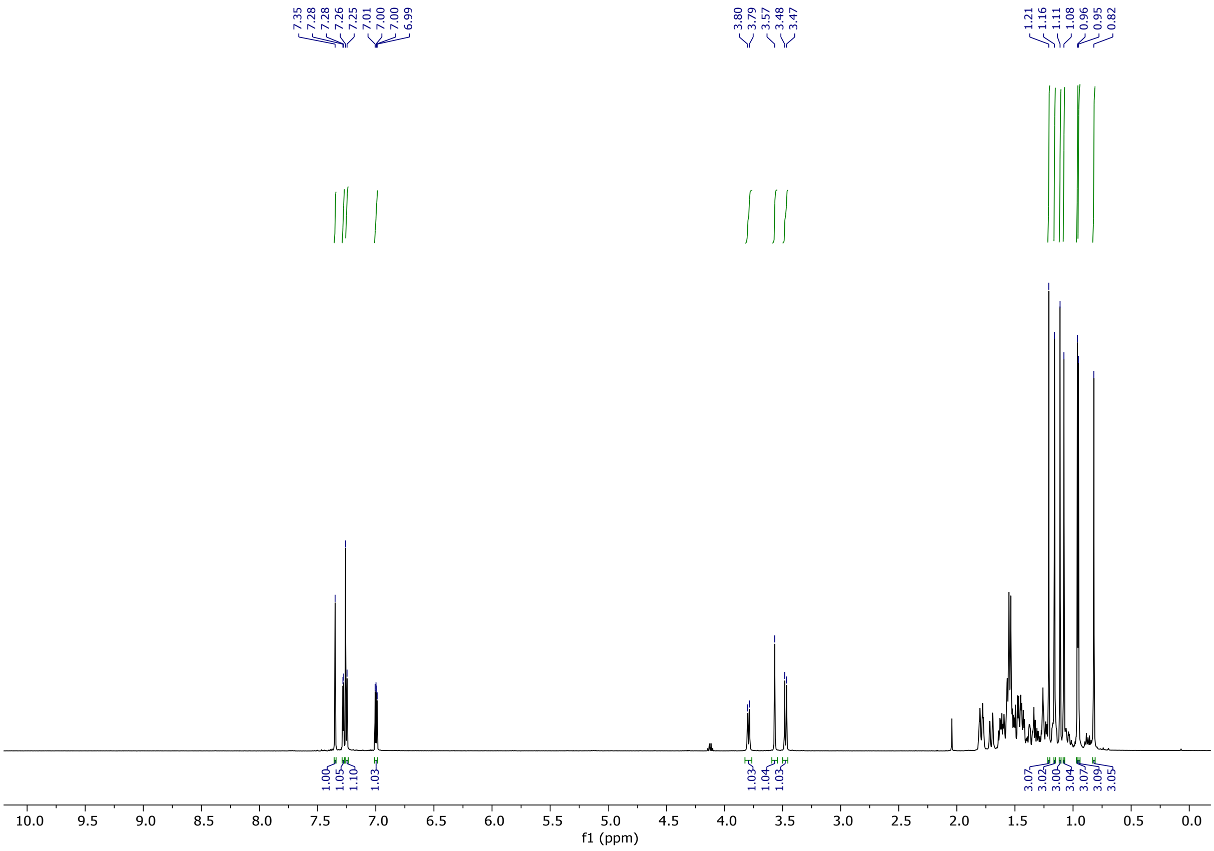

Figure S5. ^{1}H NMR spectrum of the compound **4c** ($CDCl_3$, 500 MHz).

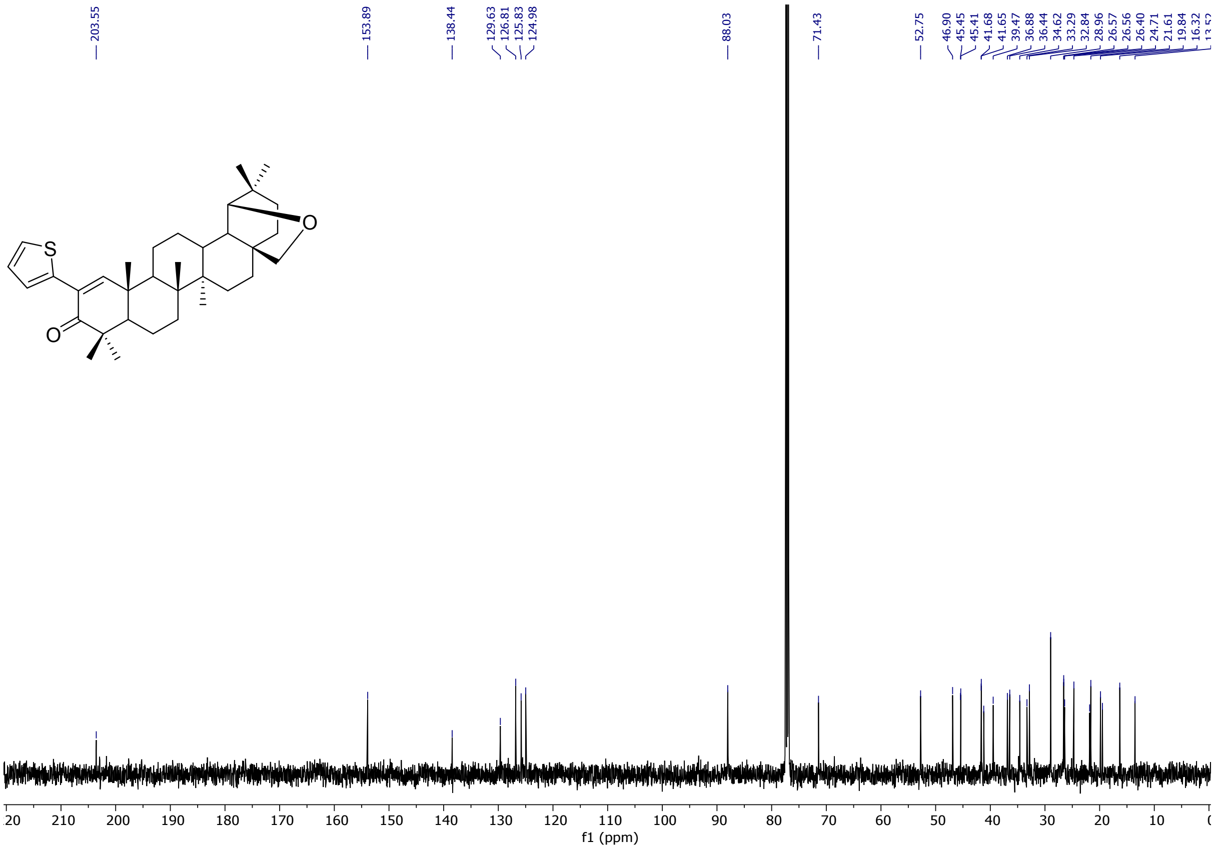

Figure S6. ^{13}C NMR spectrum of the compound **4c** ($CDCl_3$, 126 MHz).

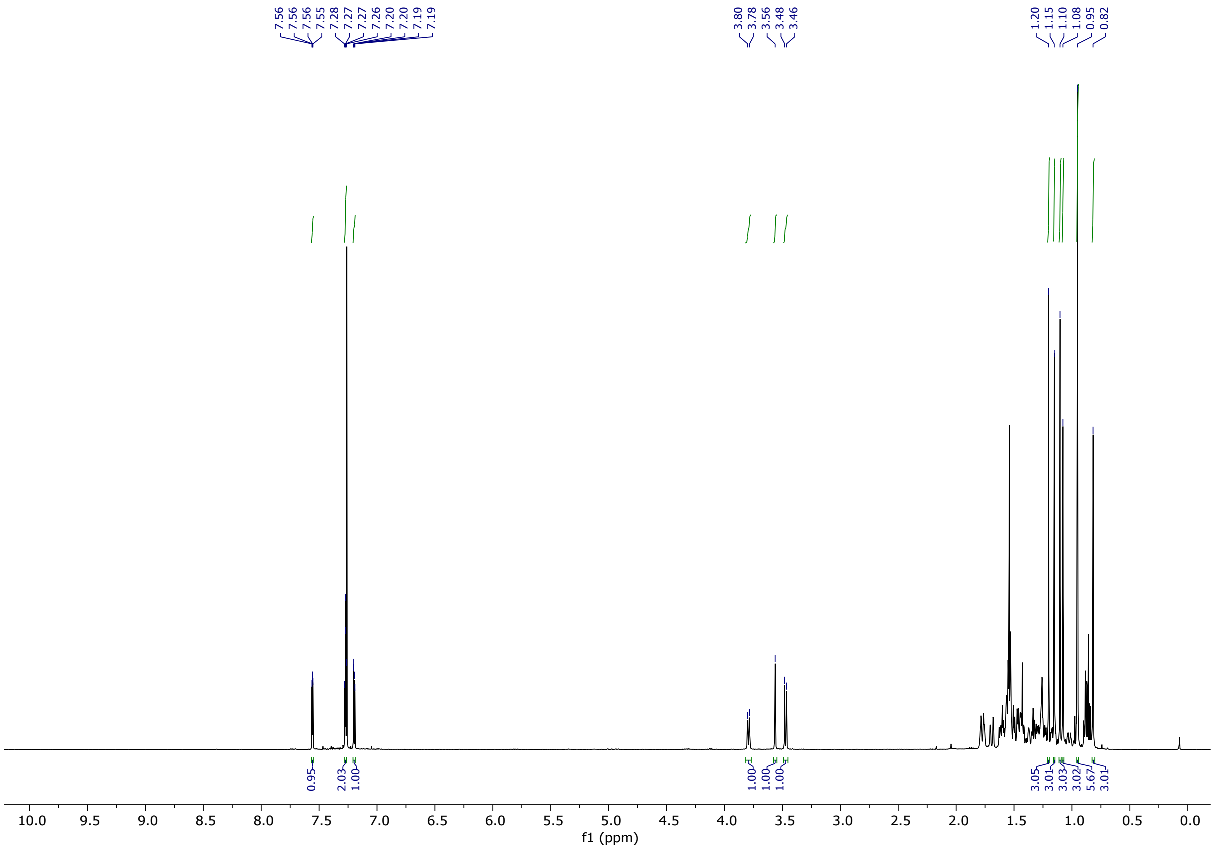

Figure S7. ^{1}H NMR spectrum of the compound **4d** ($CDCl_3$, 500 MHz).

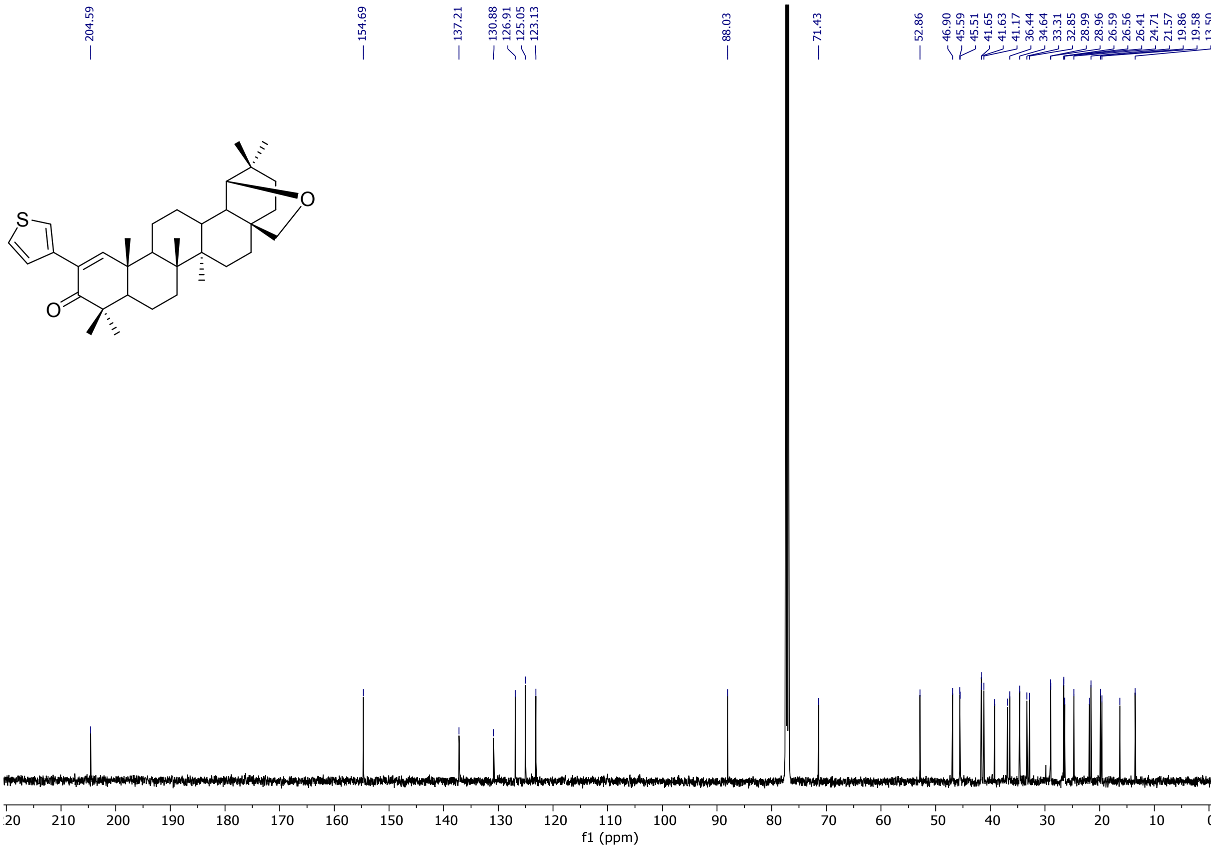

Figure S8. ^{13}C NMR spectrum of the compound **4d** ($CDCl_3$, 126 MHz).

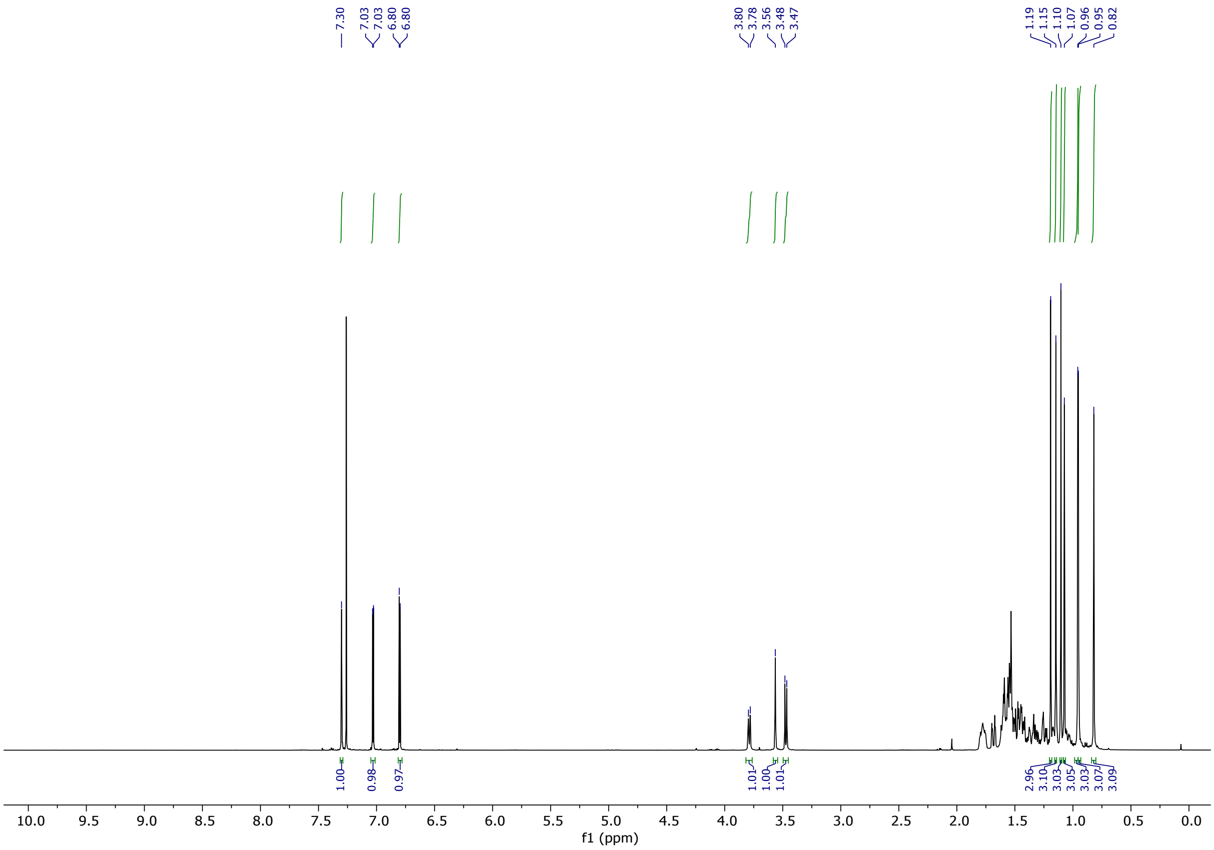

Figure S9. ^{1}H NMR spectrum of the compound **4e** ($CDCl_3$, 500 MHz).

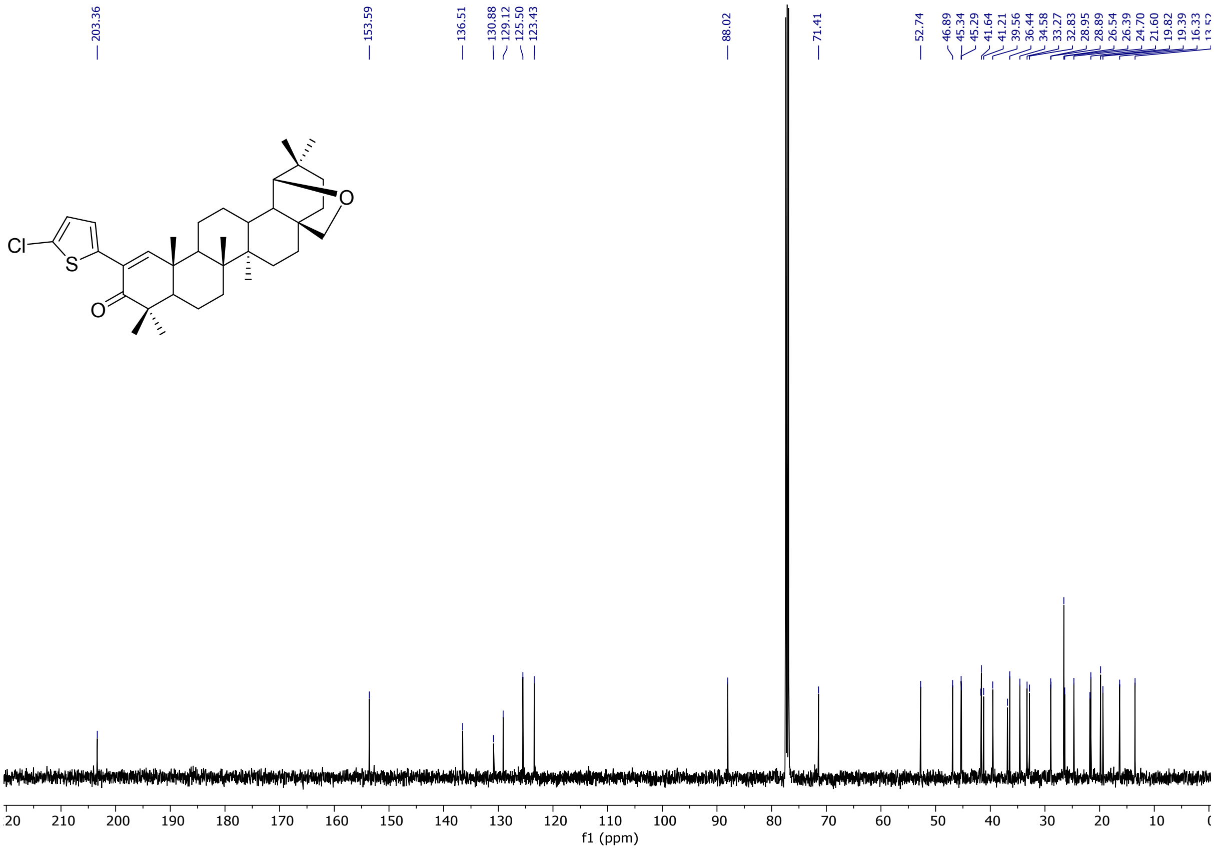

Figure S10. ^{13}C NMR spectrum of the compound **4e** ($CDCl_3$, 126 MHz).

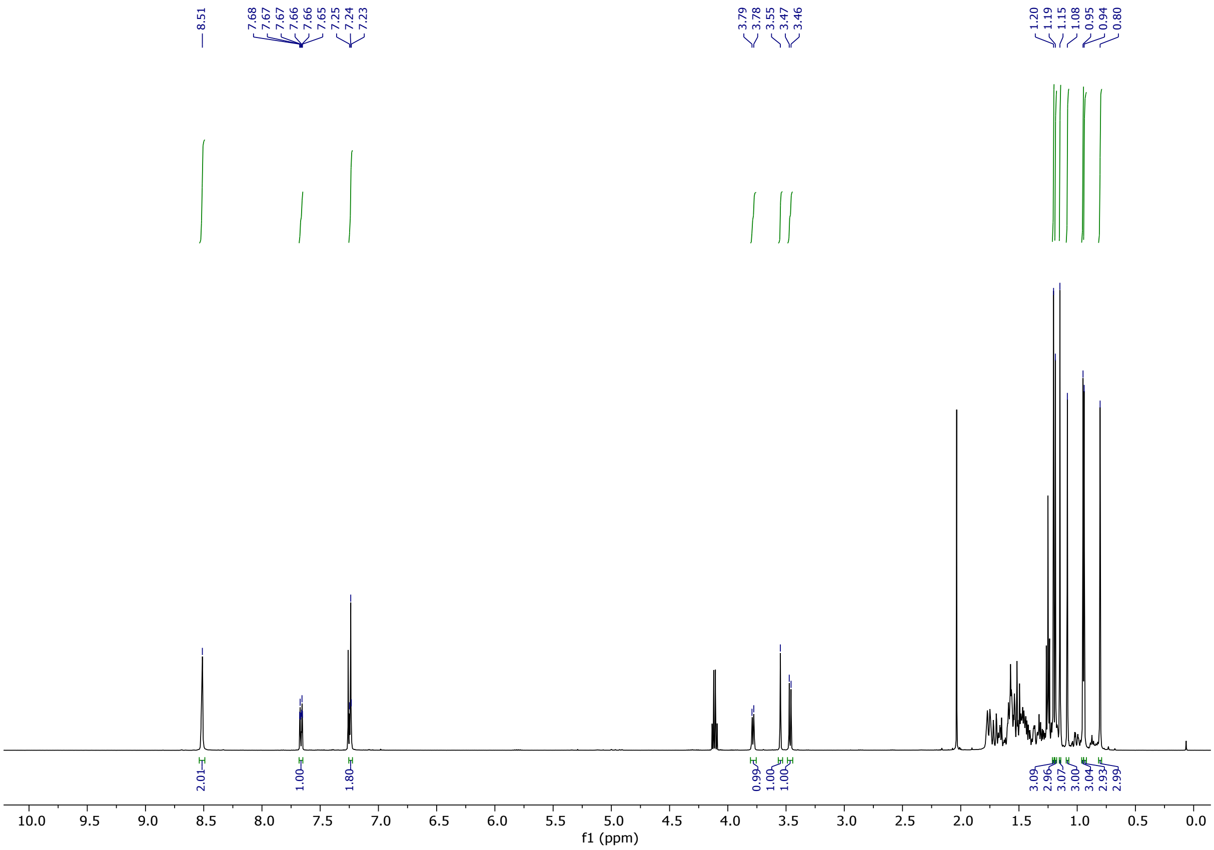

Figure S11. ^{1}H NMR spectrum of the compound **4g** ($CDCl_3$, 500 MHz).

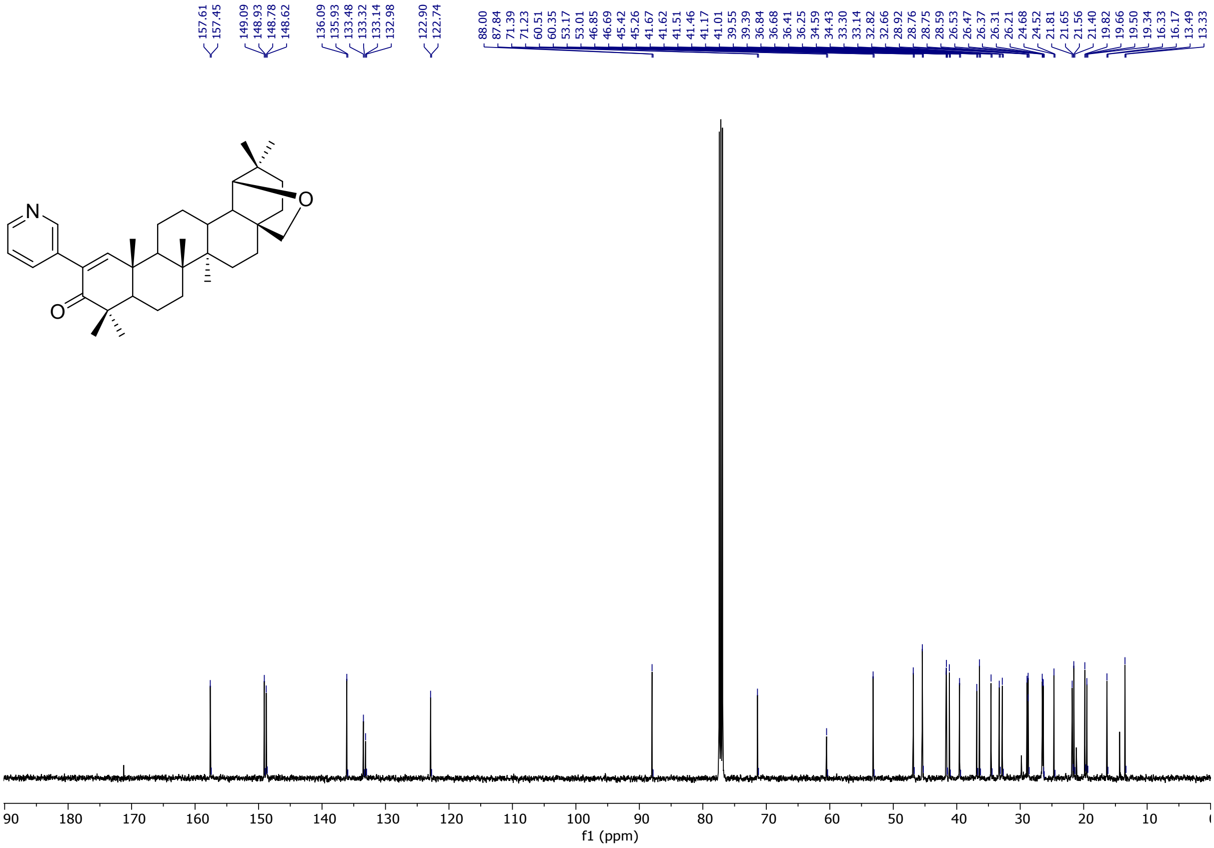

Figure S12. ^{13}C NMR spectrum of the compound **4g** ($CDCl_3$, 126 MHz).

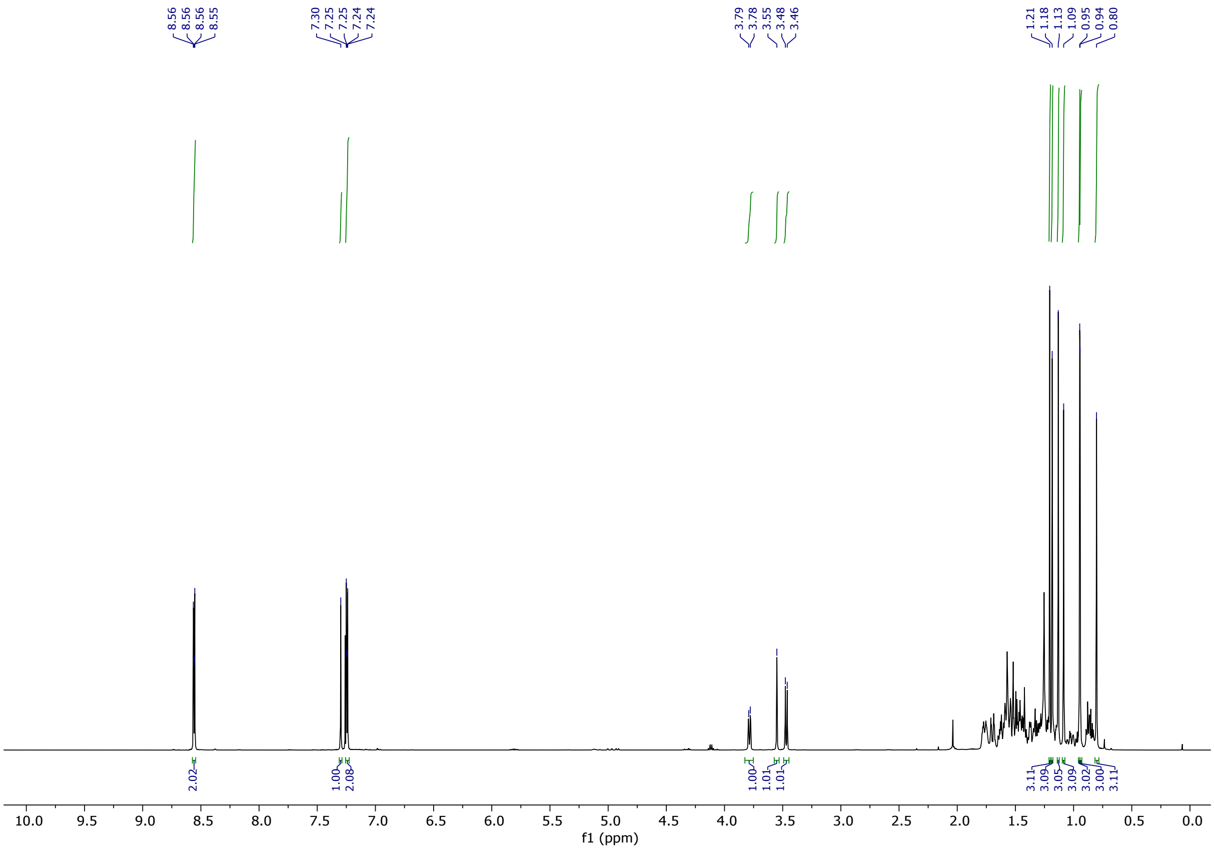

Figure S13. ^{1}H NMR spectrum of the compound **4h** ($CDCl_3$, 500 MHz).

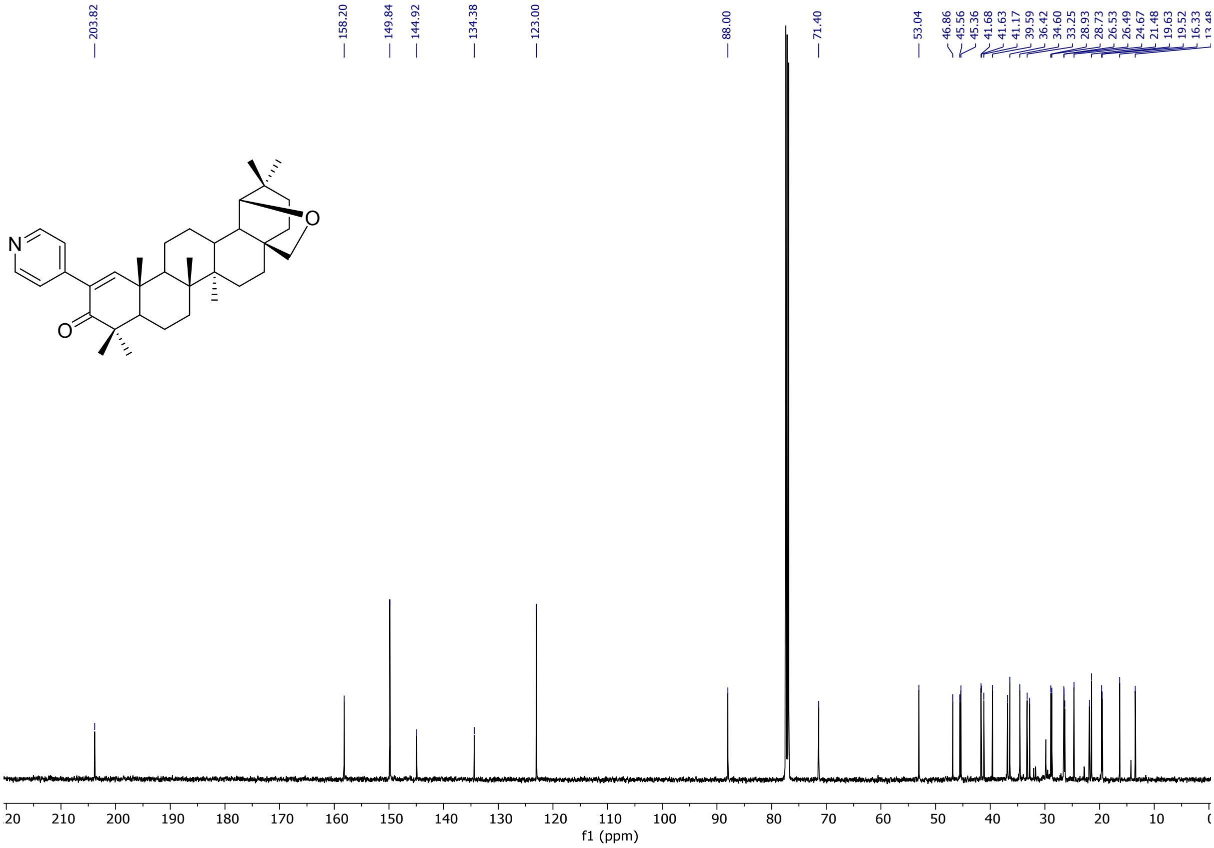

Figure S14. ^{13}C NMR spectrum of the compound **4h** ($CDCl_3$, 126 MHz).

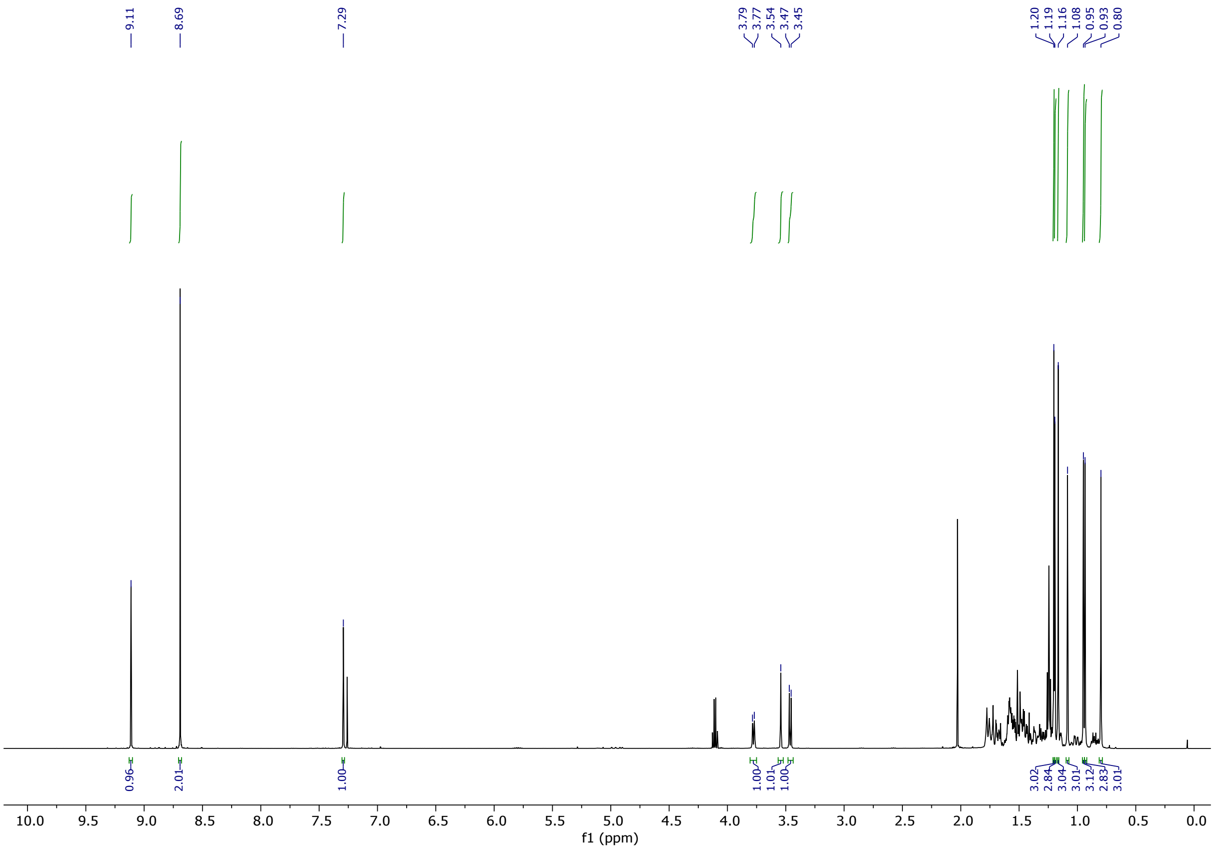

Figure S15. ^{1}H NMR spectrum of the compound **4i** ($CDCl_3$, 500 MHz).

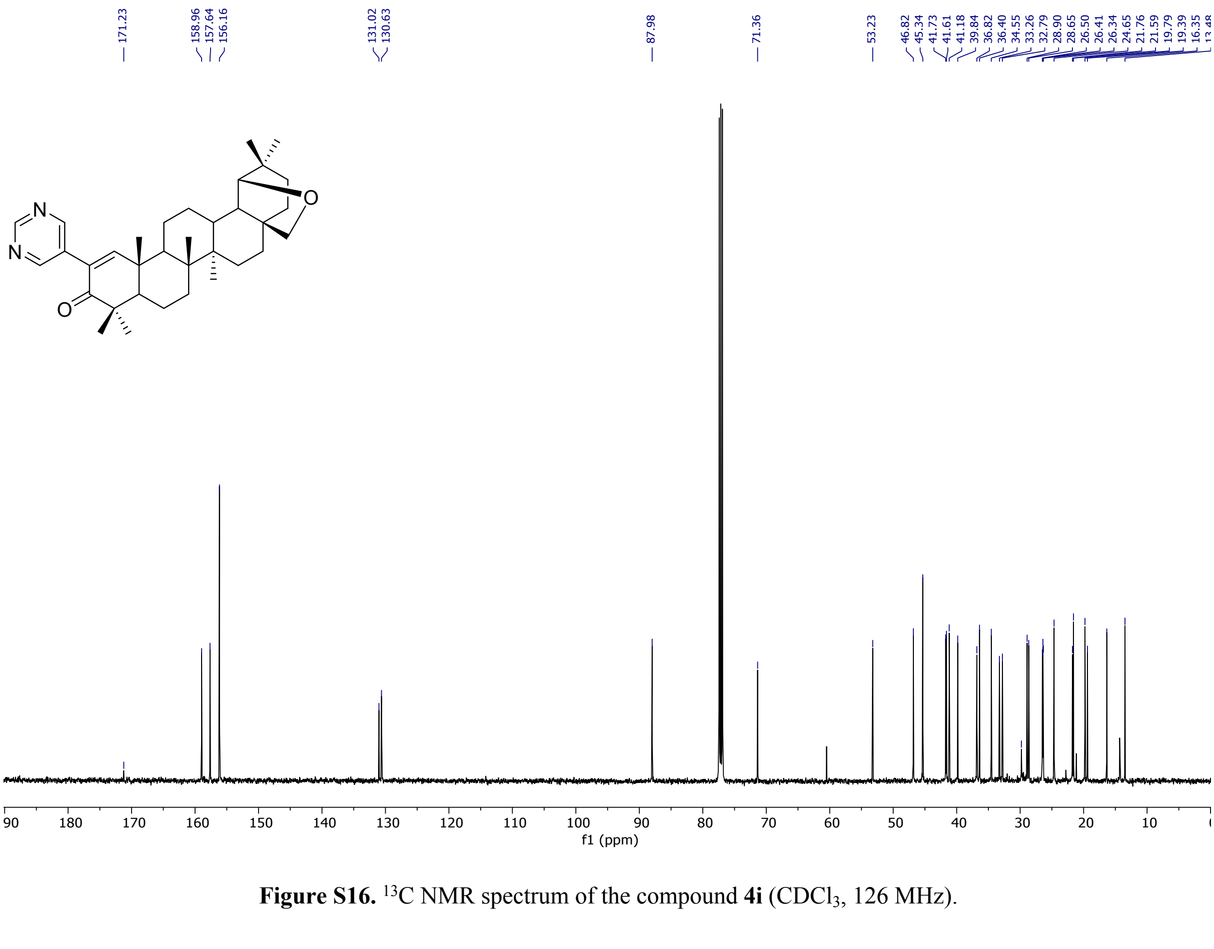

Figure S16. ^{13}C NMR spectrum of the compound **4i** ($CDCl_3$, 126 MHz).

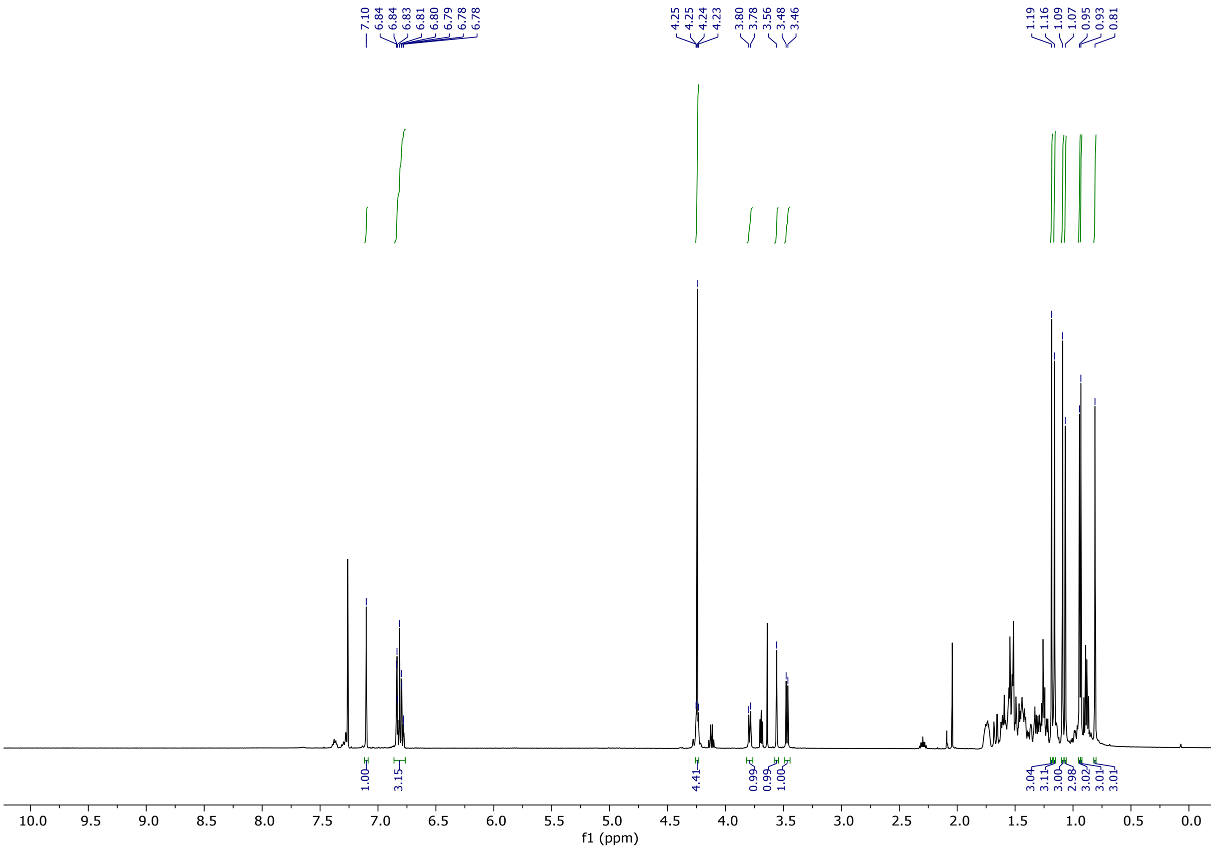

Figure S17. ^{1}H NMR spectrum of the compound **4j** ($CDCl_3$, 500 MHz).

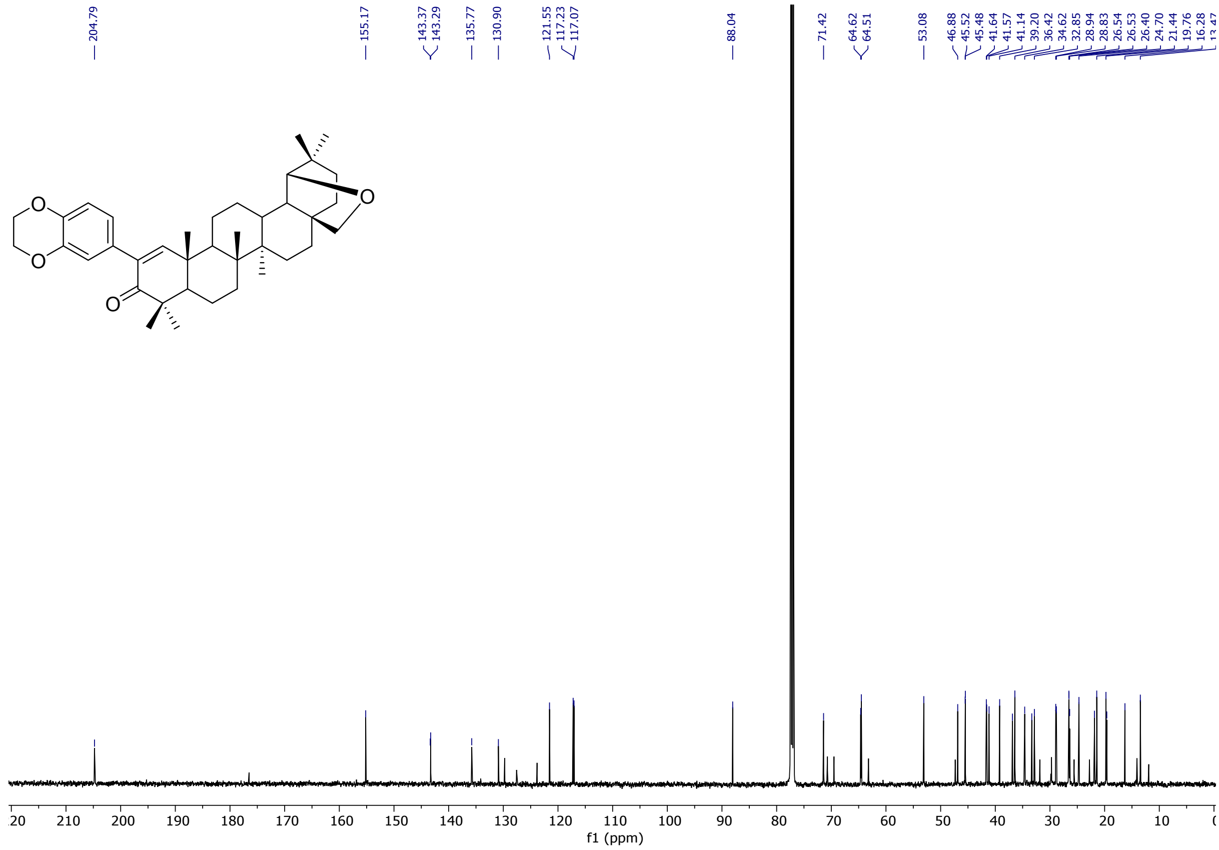

Figure S18. ^{13}C NMR spectrum of the compound **4j** ($CDCl_3$, 126 MHz).

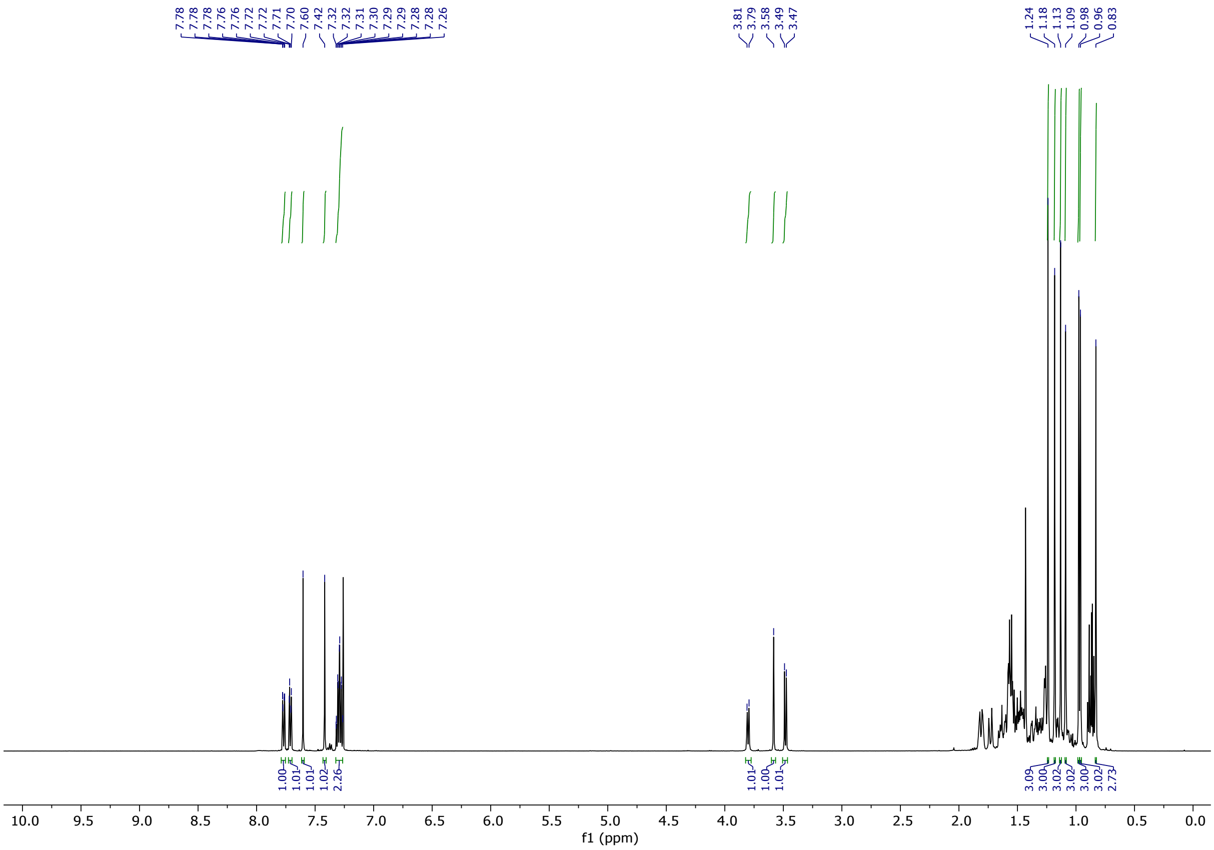

Figure S19. ^{1}H NMR spectrum of the compound **4k** ($CDCl_3$, 500 MHz).

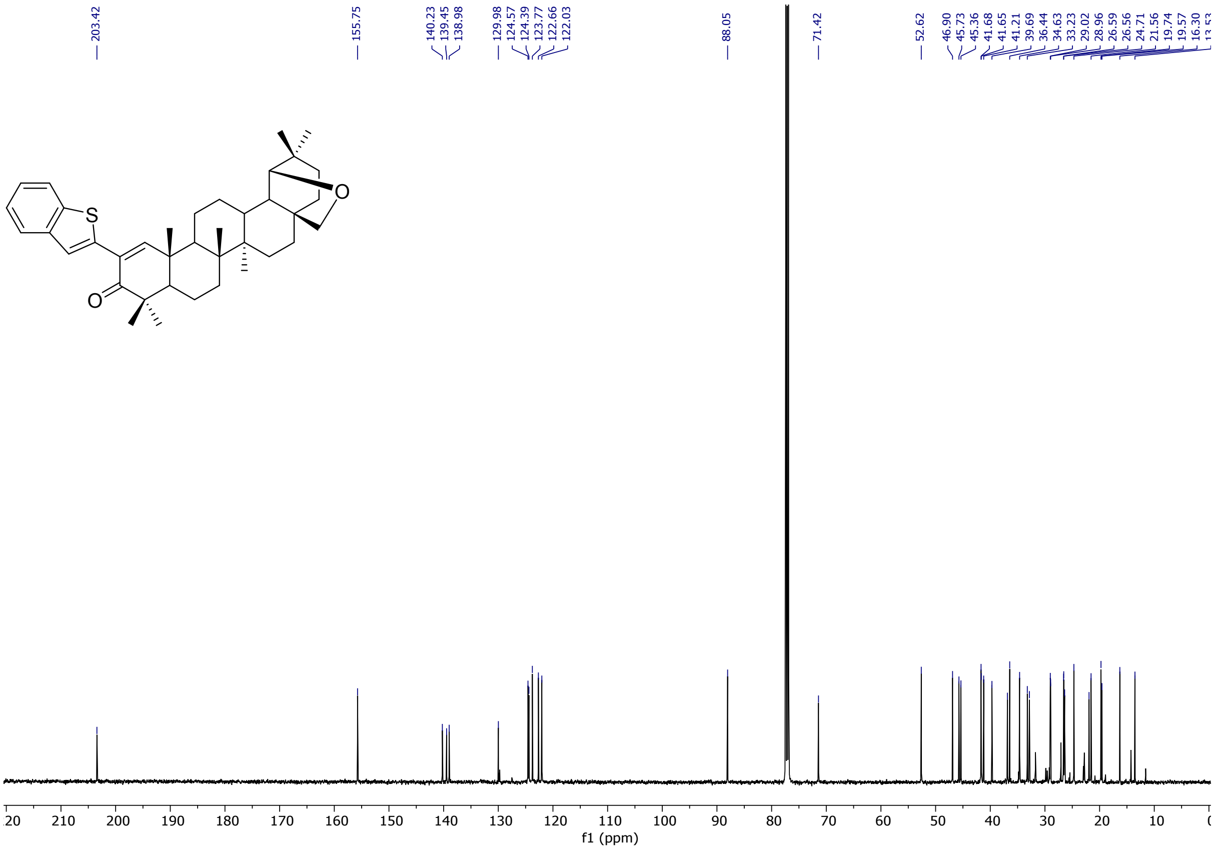

Figure S20. ^{13}C NMR spectrum of the compound **4k** ($CDCl_3$, 126 MHz).

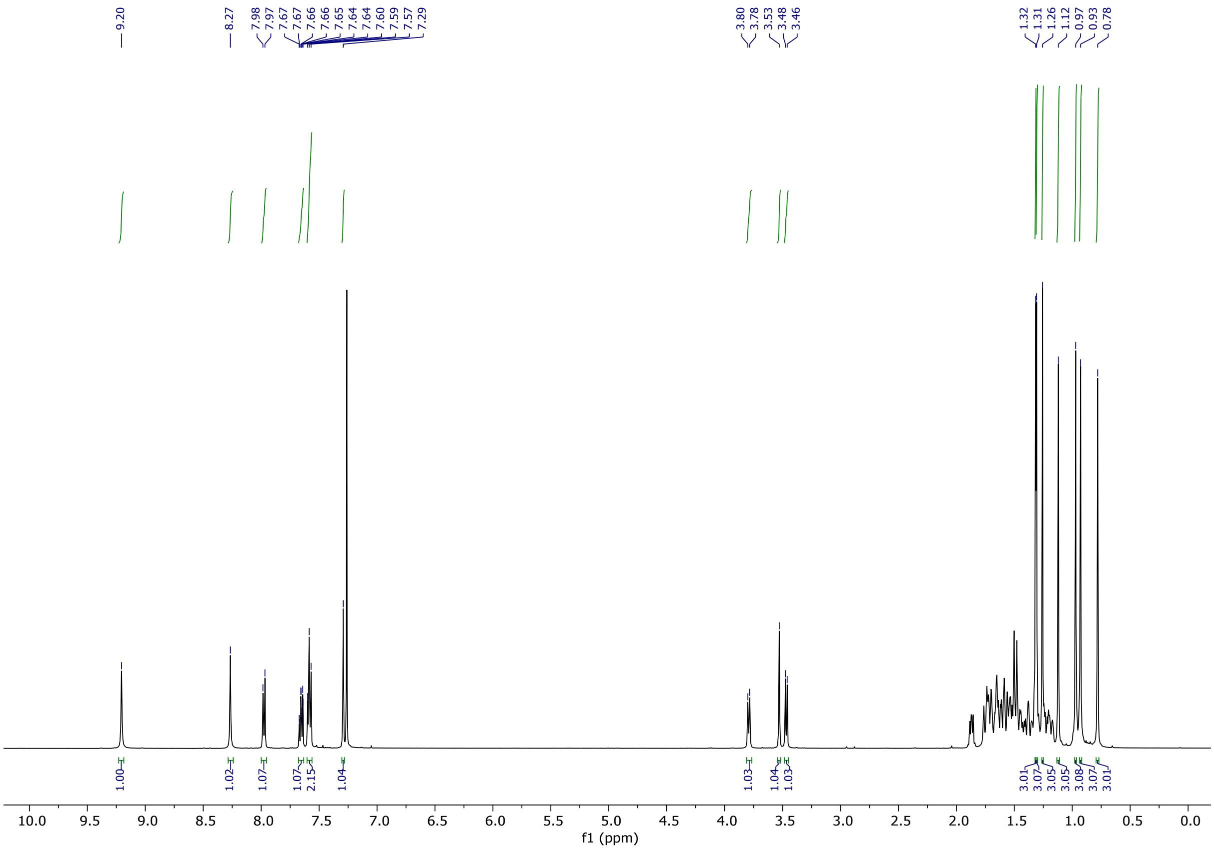

Figure S21. ^{1}H NMR spectrum of the compound **4l** ($CDCl_3$, 500 MHz).

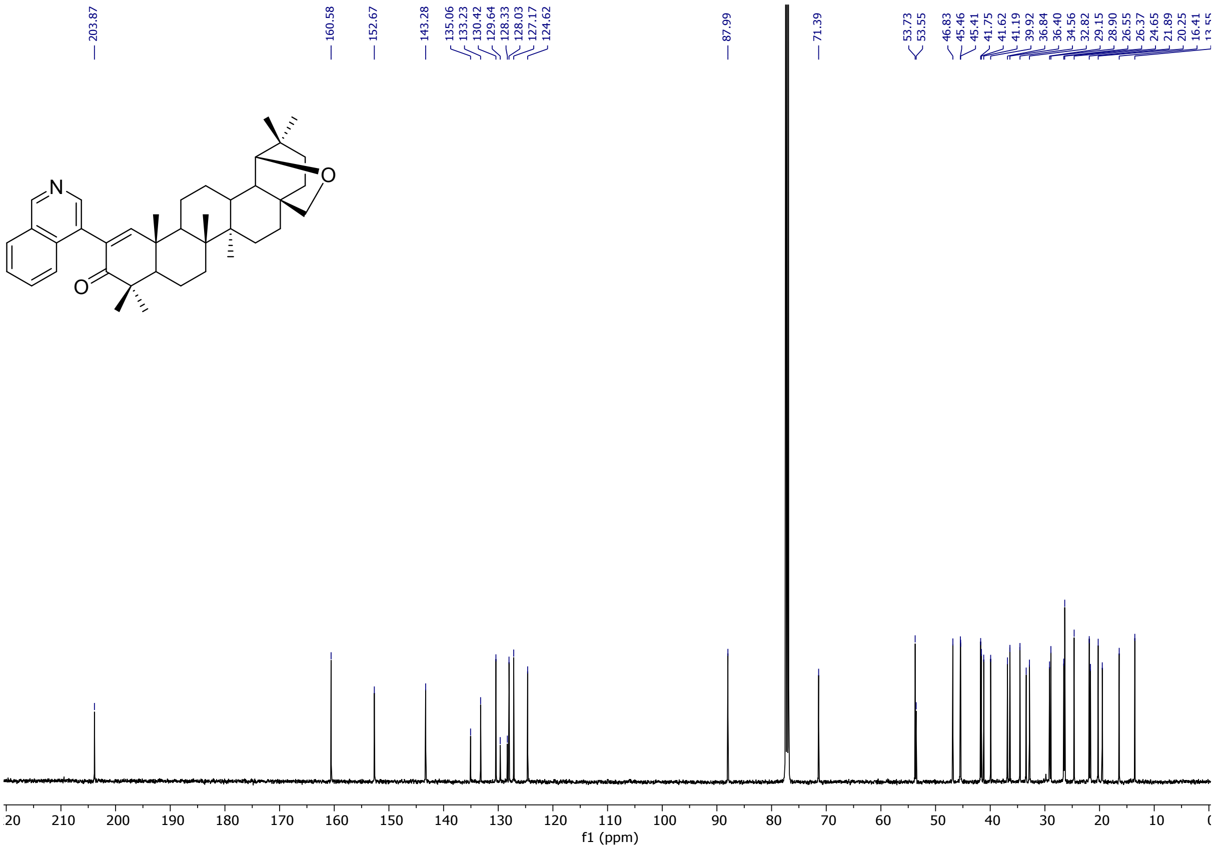

Figure S22. ^{13}C NMR spectrum of the compound **4l** ($CDCl_3$, 126 MHz).

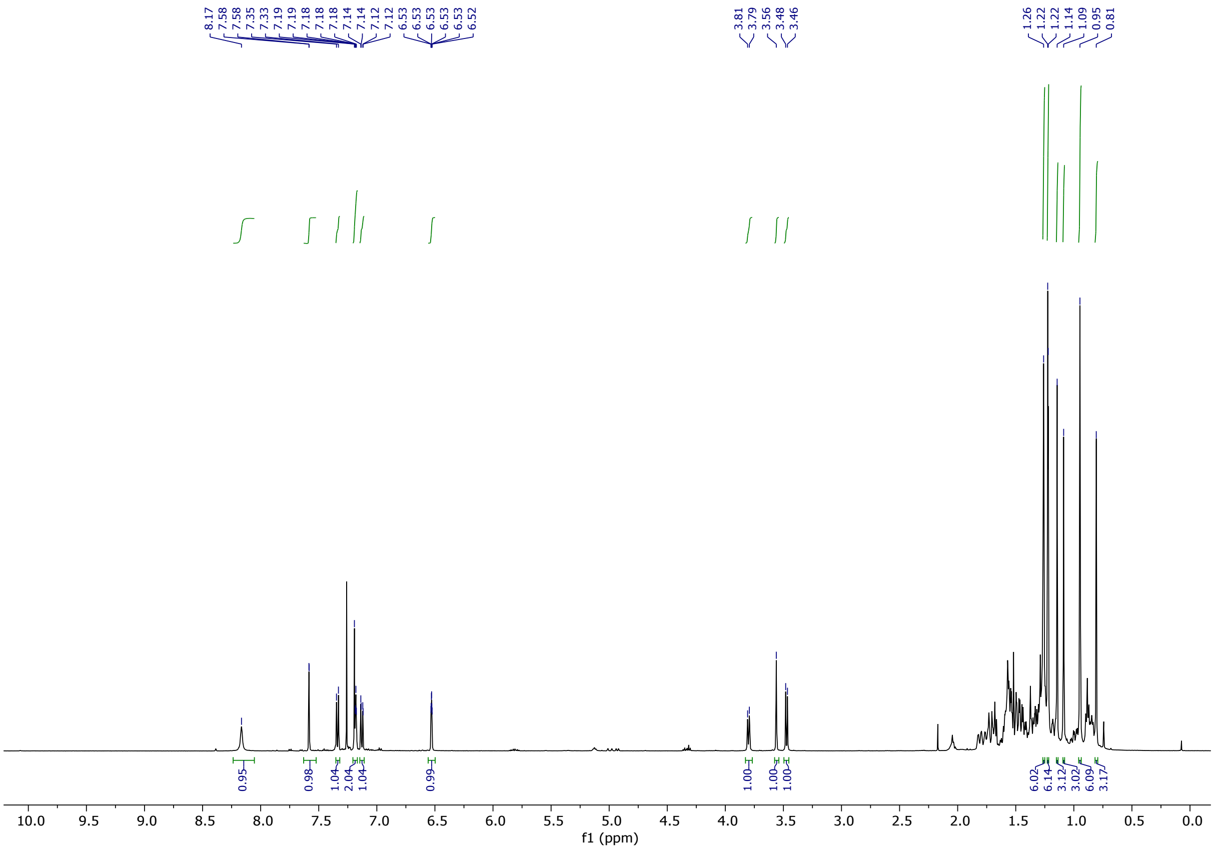

Figure S23. ^{1}H NMR spectrum of the compound **4m** ($CDCl_3$, 500 MHz).

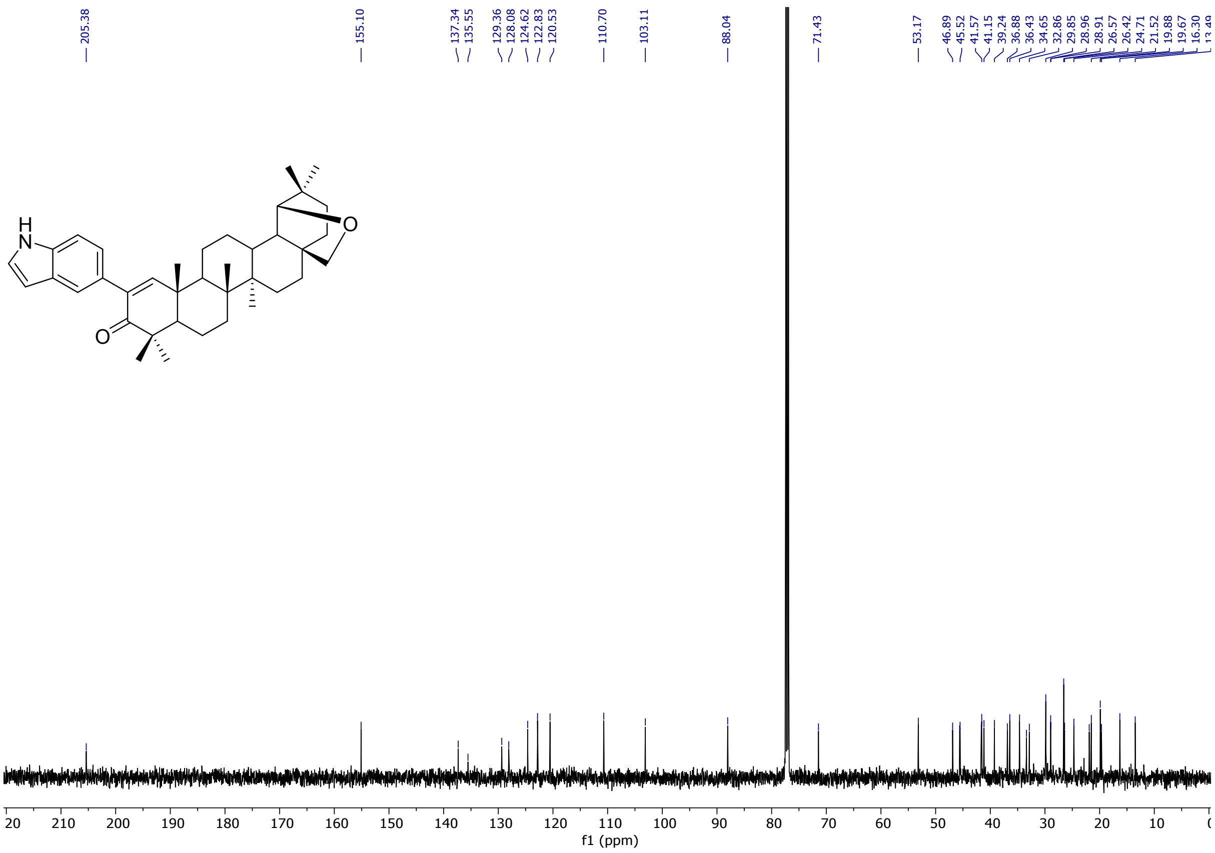

Figure S24. ^{13}C NMR spectrum of the compound **4m** ($CDCl_3$, 126 MHz).

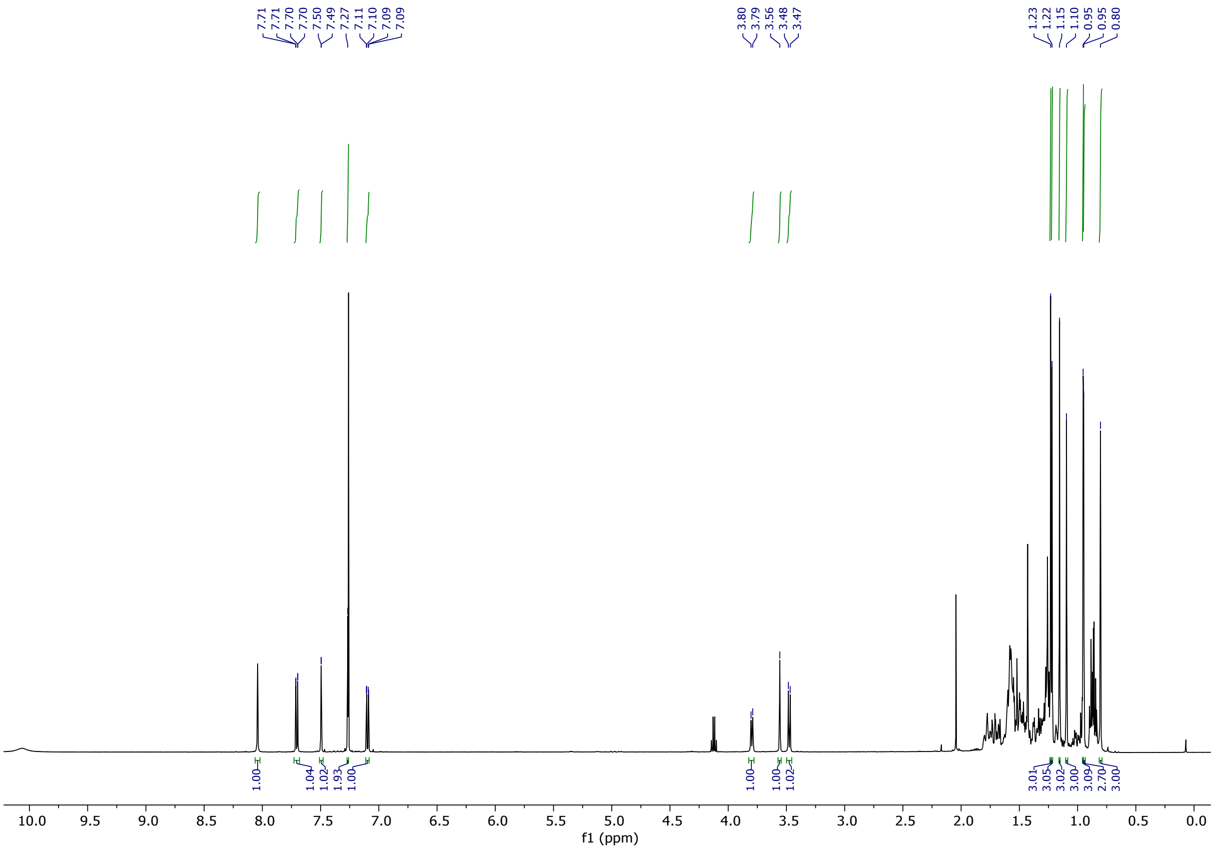

Figure S25. ^{1}H NMR spectrum of the compound **4n** ($CDCl_3$, 500 MHz).

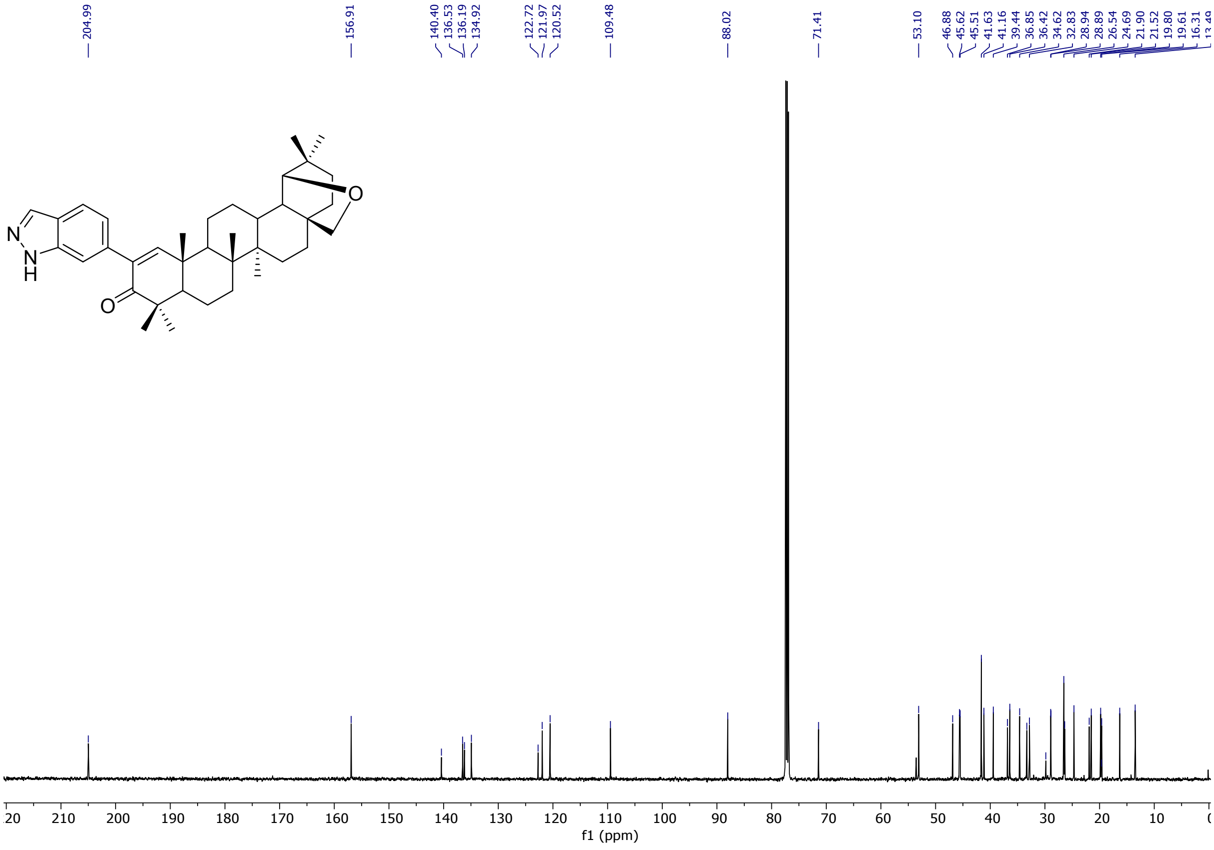

Figure S26. ^{13}C NMR spectrum of the compound **4n** ($CDCl_3$, 126 MHz).

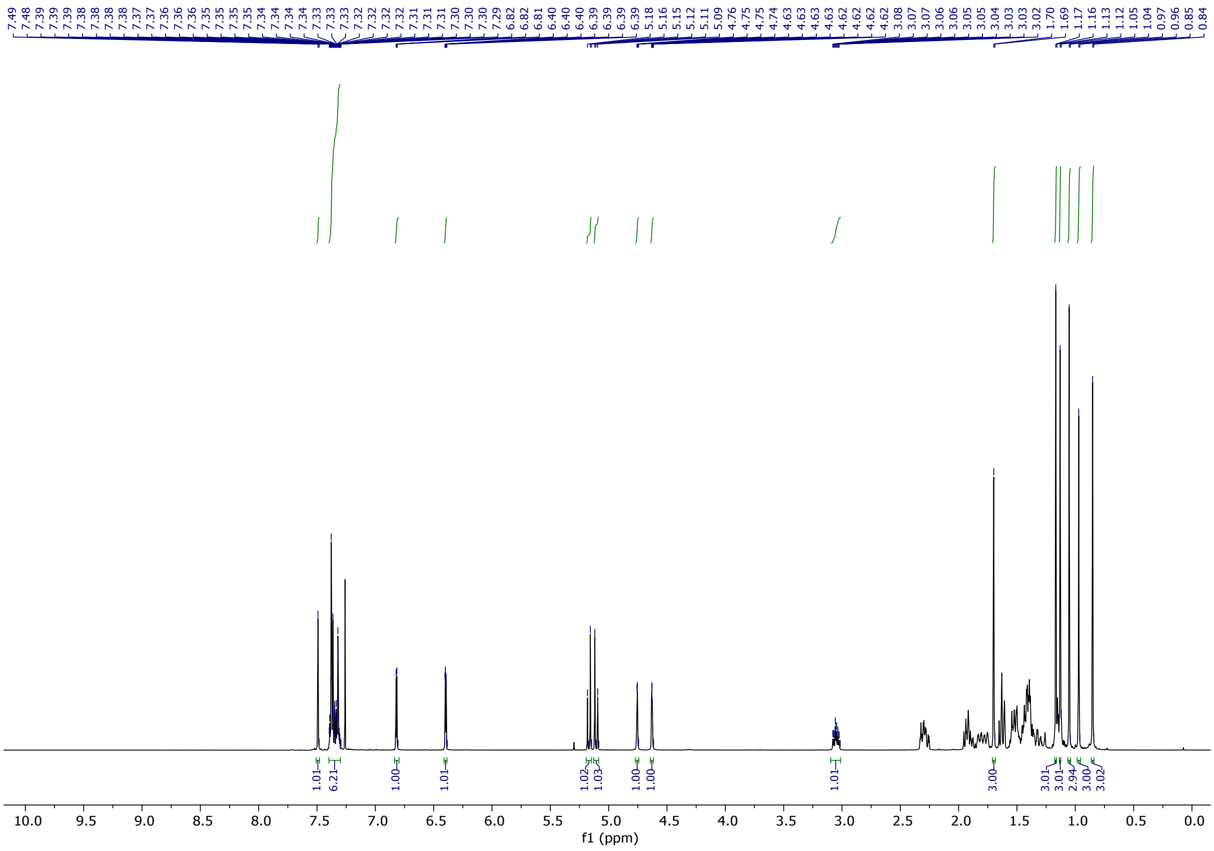

Figure S27. ^{1}H NMR spectrum of the compound **10a** ($CDCl_3$, 500 MHz).

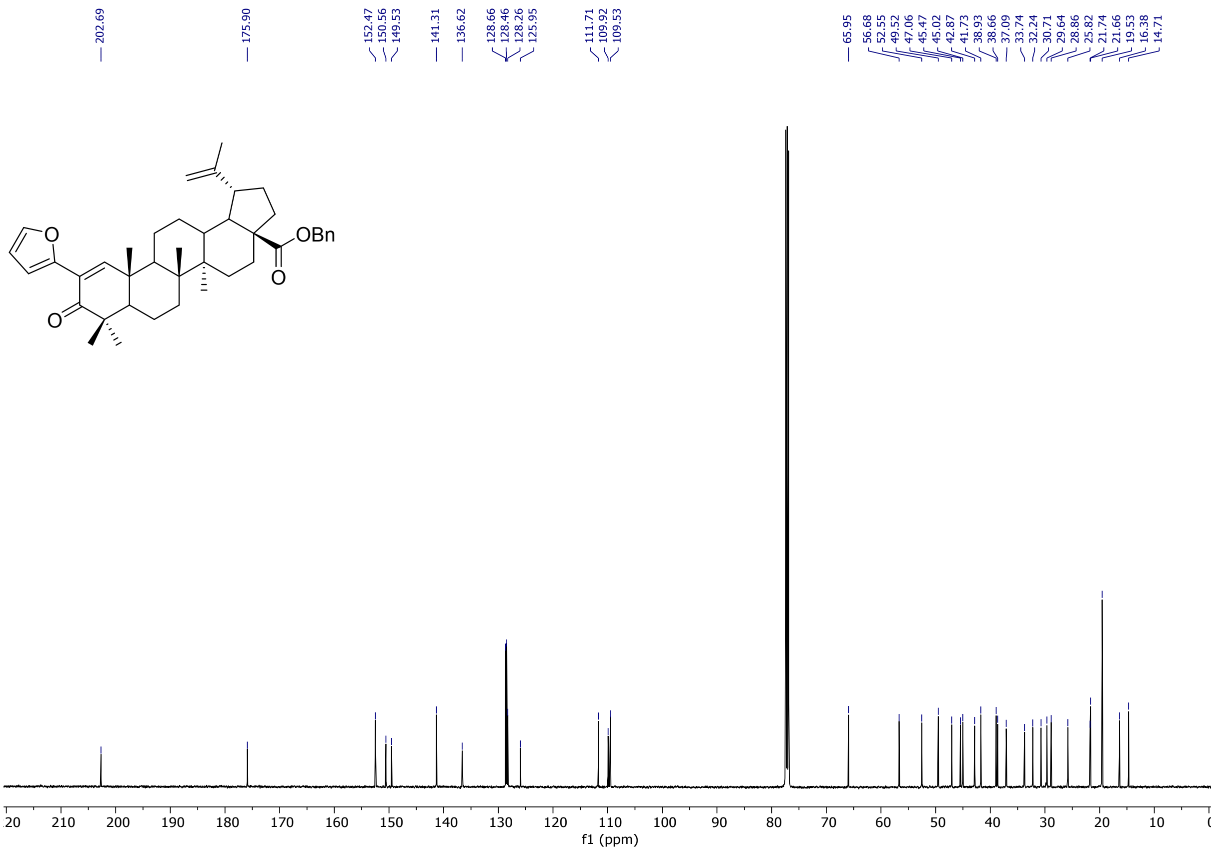

Figure S28. ^{13}C NMR spectrum of the compound **10a** ($CDCl_3$, 126 MHz).

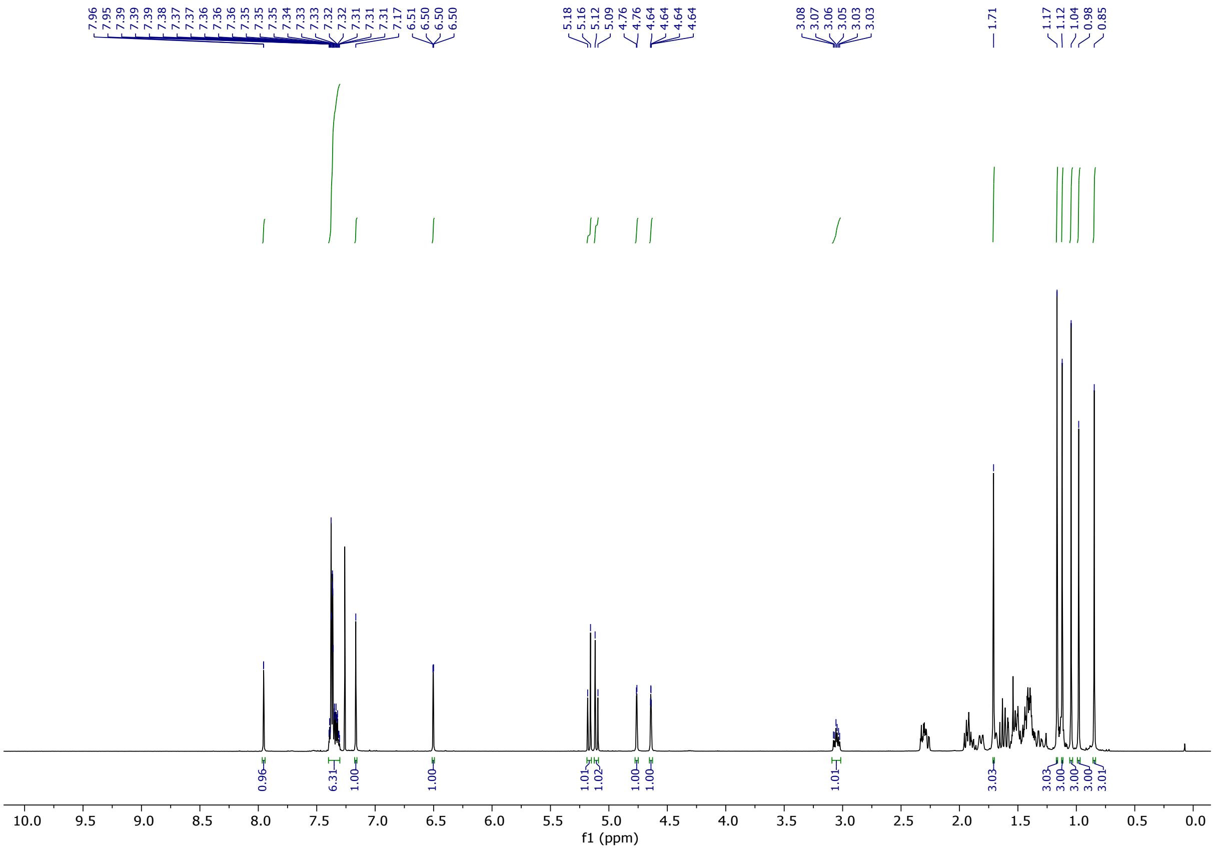

Figure S29. ^{1}H NMR spectrum of the compound **10b** ($CDCl_3$, 500 MHz).

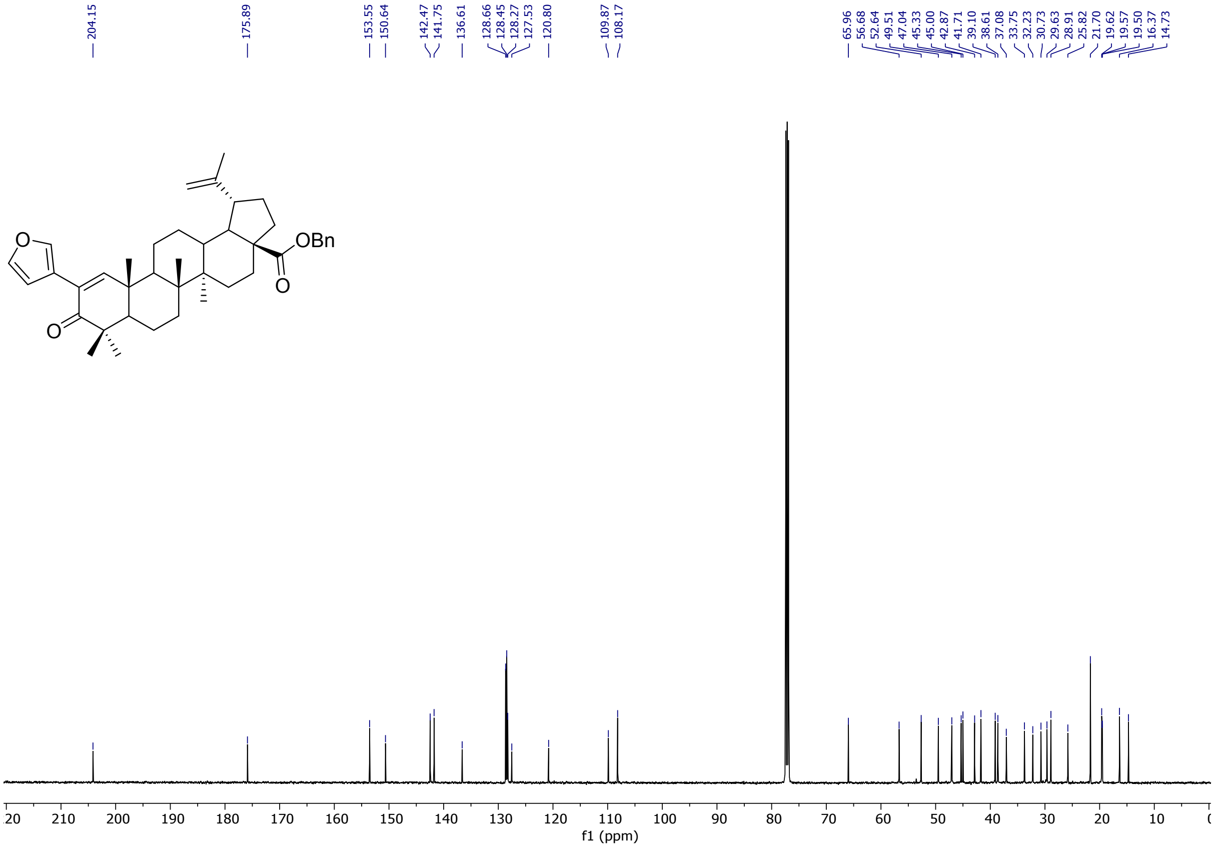

Figure S30. ^{13}C NMR spectrum of the compound **10b** ($CDCl_3$, 126 MHz).

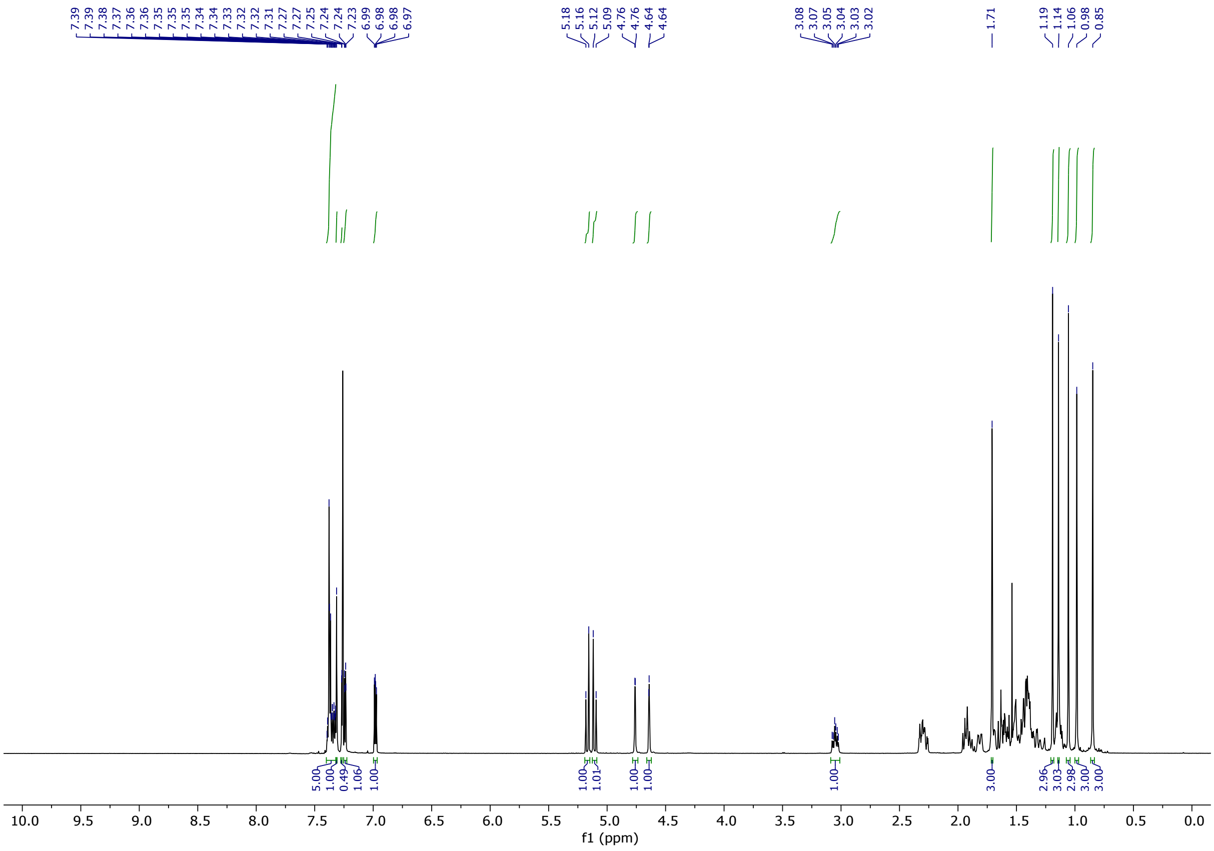

Figure S31. ^{1}H NMR spectrum of the compound **10c** ($CDCl_3$, 500 MHz).

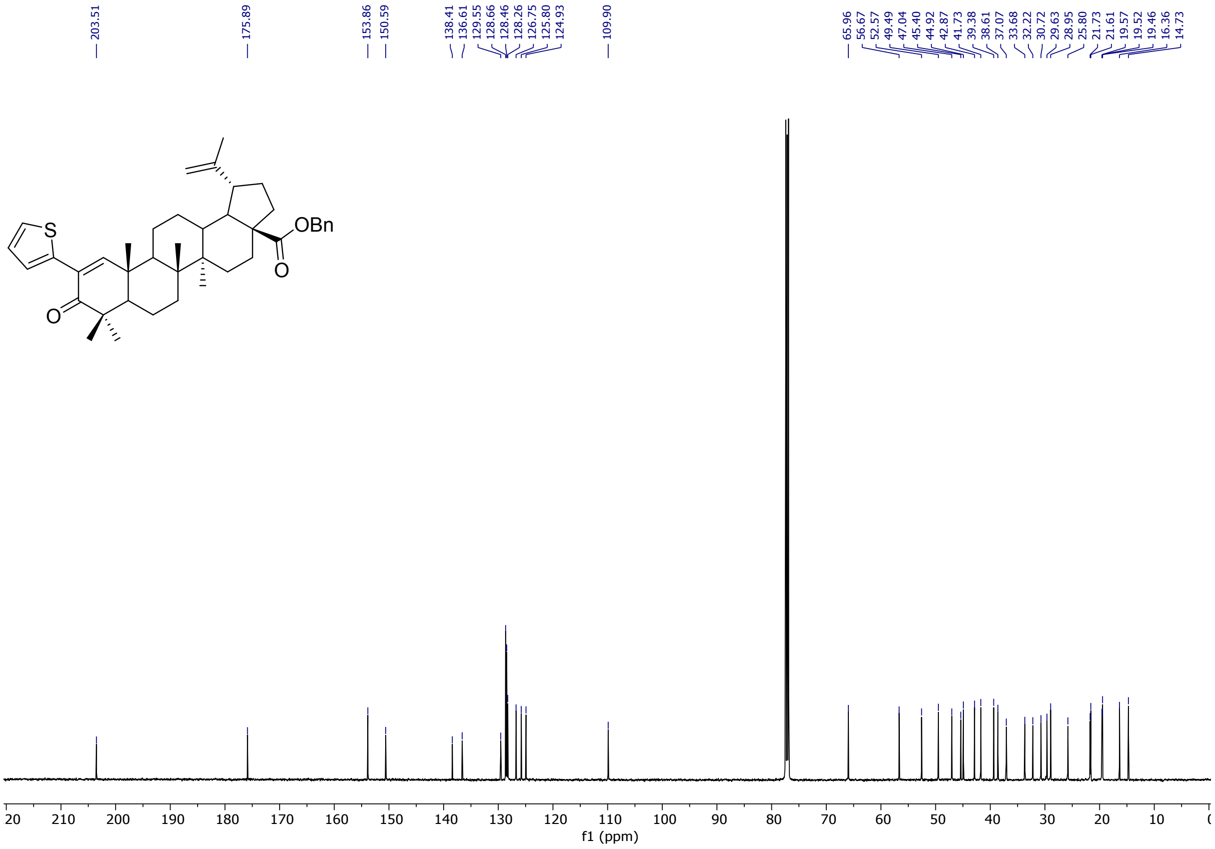

Figure S32. ^{13}C NMR spectrum of the compound **10c** ($CDCl_3$, 126 MHz).

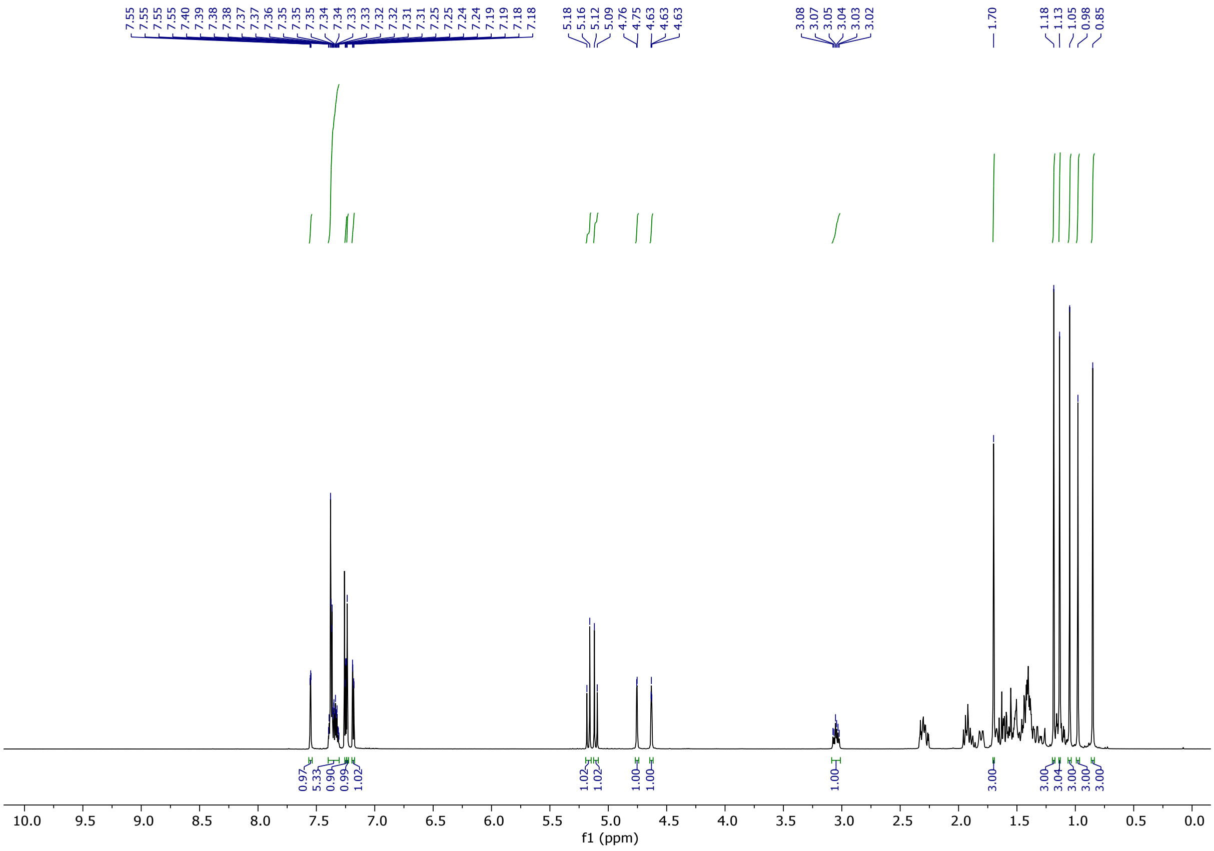

Figure S33. ^{1}H NMR spectrum of the compound **10d** ($CDCl_3$, 500 MHz).

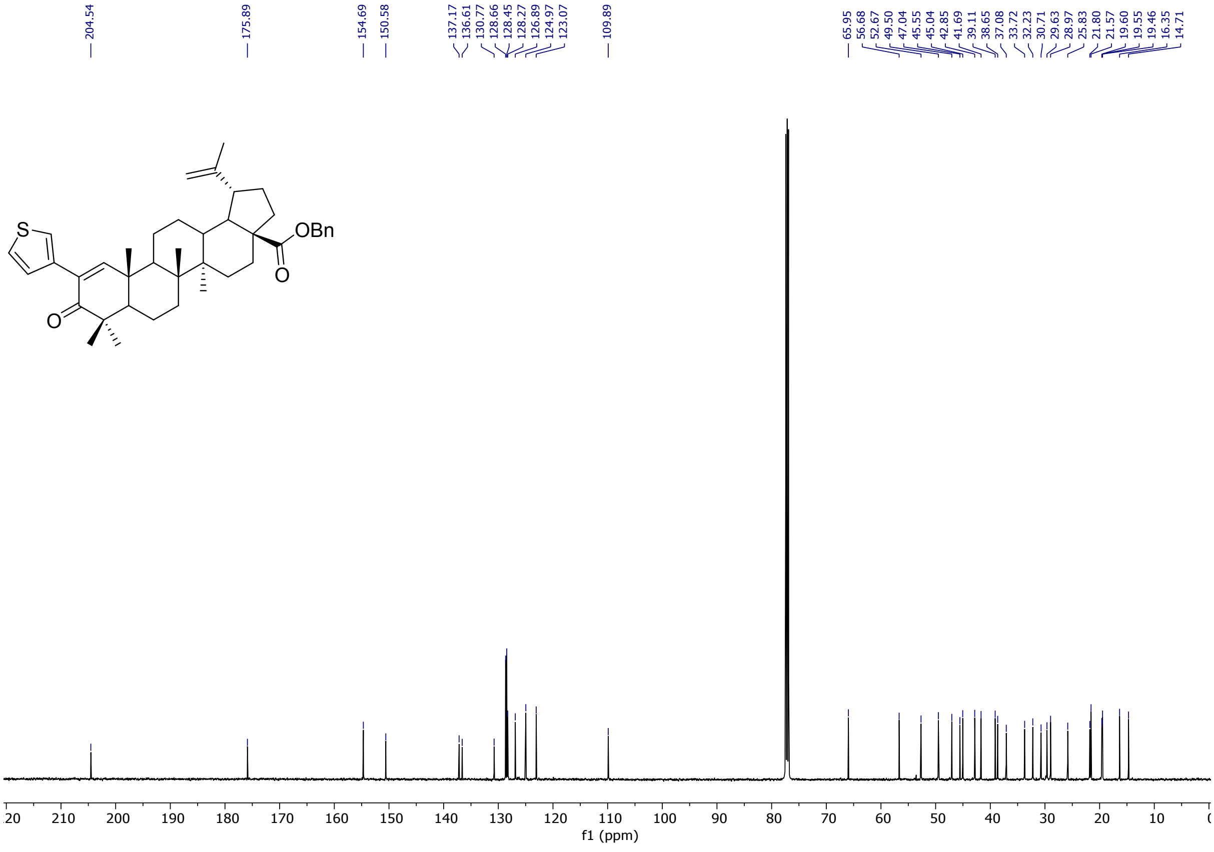

Figure S34. ^{13}C NMR spectrum of the compound **10d** ($CDCl_3$, 126 MHz).

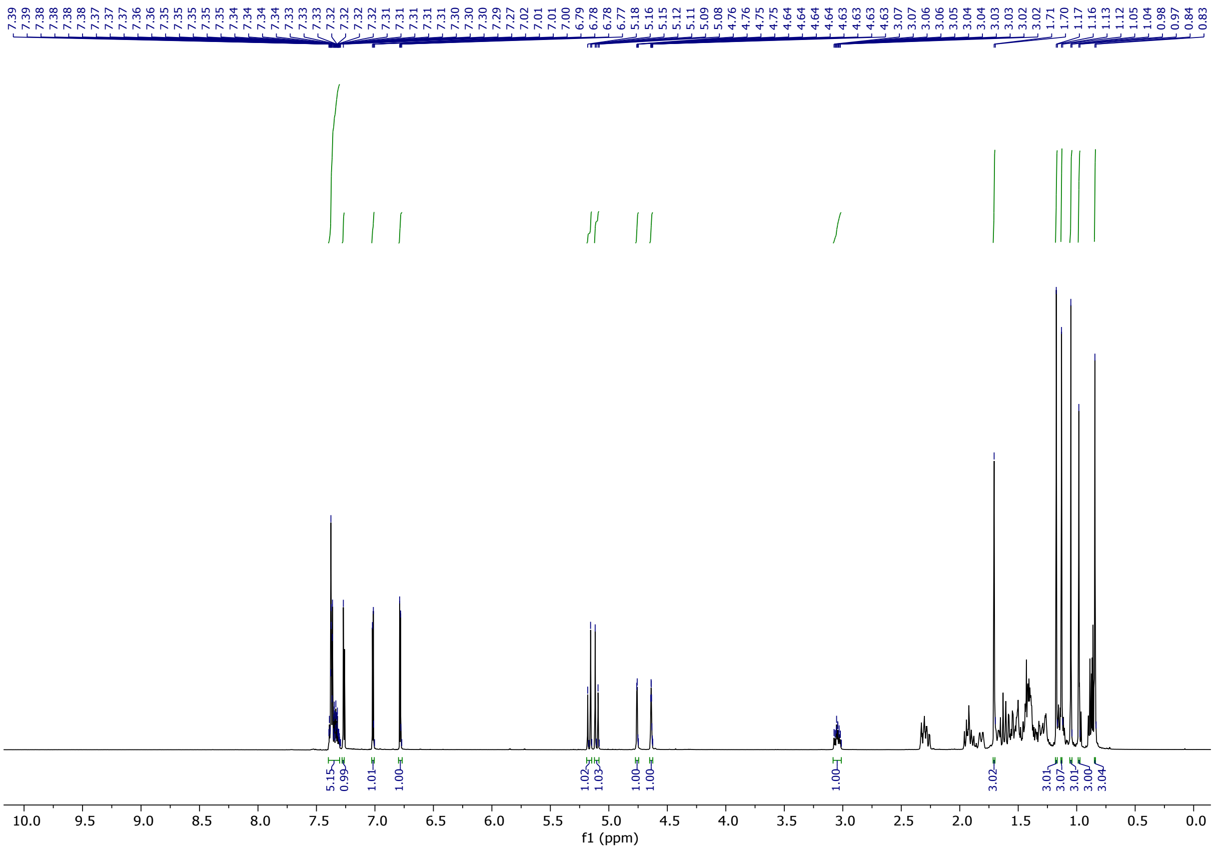

Figure S35. ^{1}H NMR spectrum of the compound **10e** ($CDCl_3$, 500 MHz).

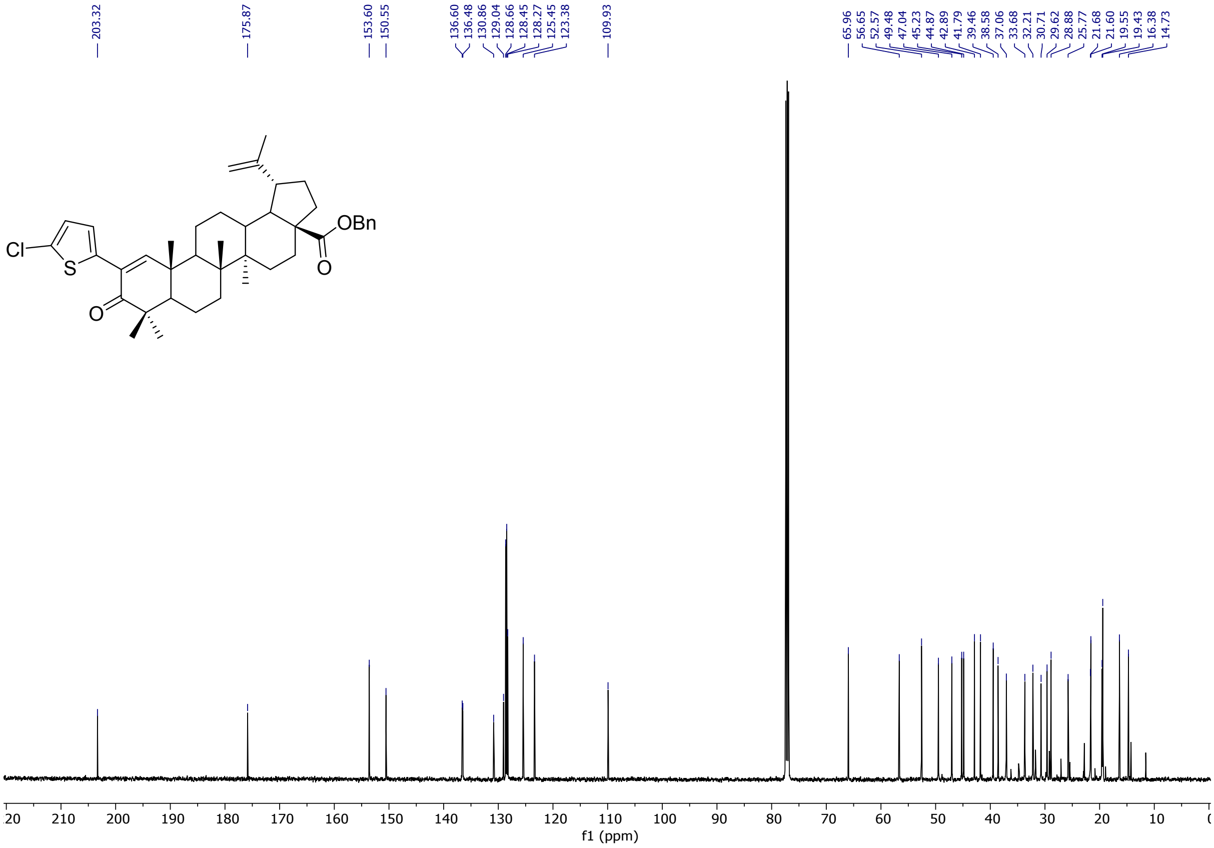

Figure S36. ^{13}C NMR spectrum of the compound **10e** ($CDCl_3$, 126 MHz).

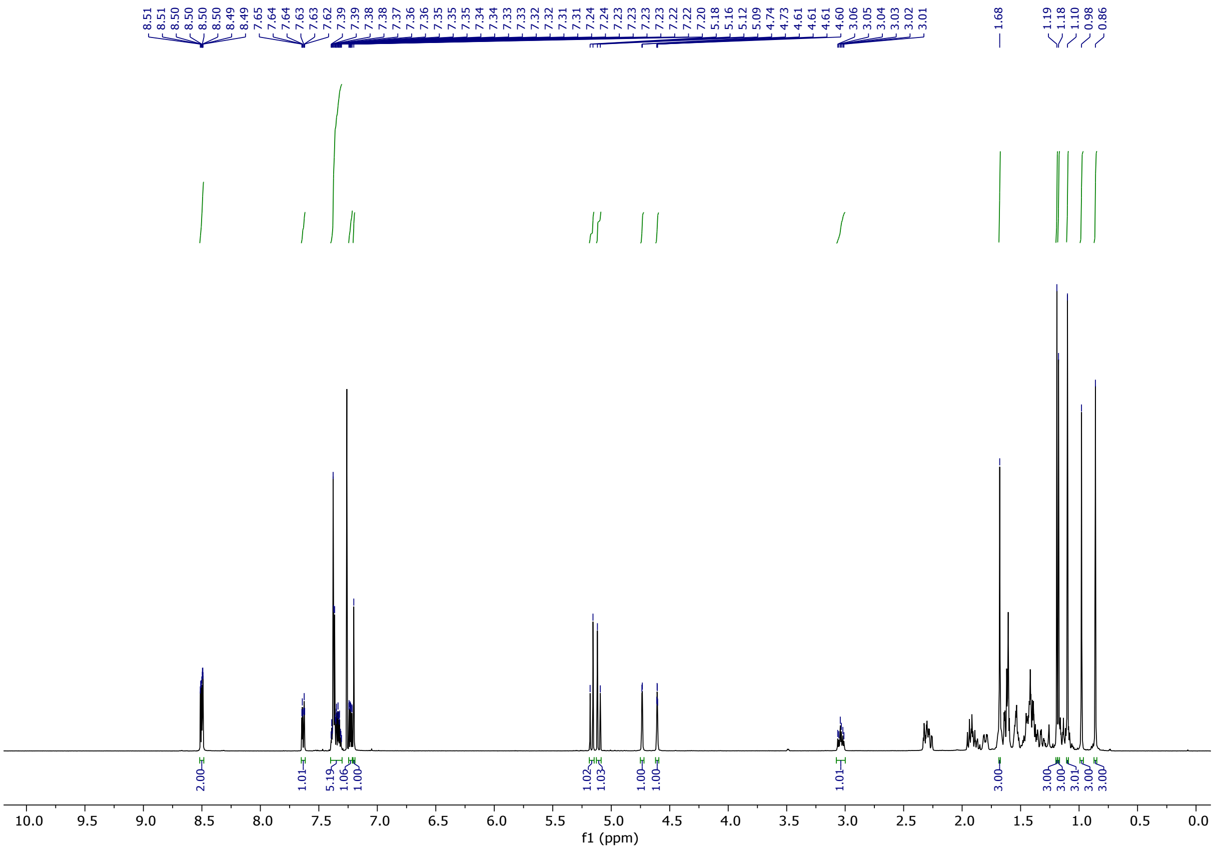

Figure S37. ^{1}H NMR spectrum of the compound **10g** ($CDCl_3$, 500 MHz).

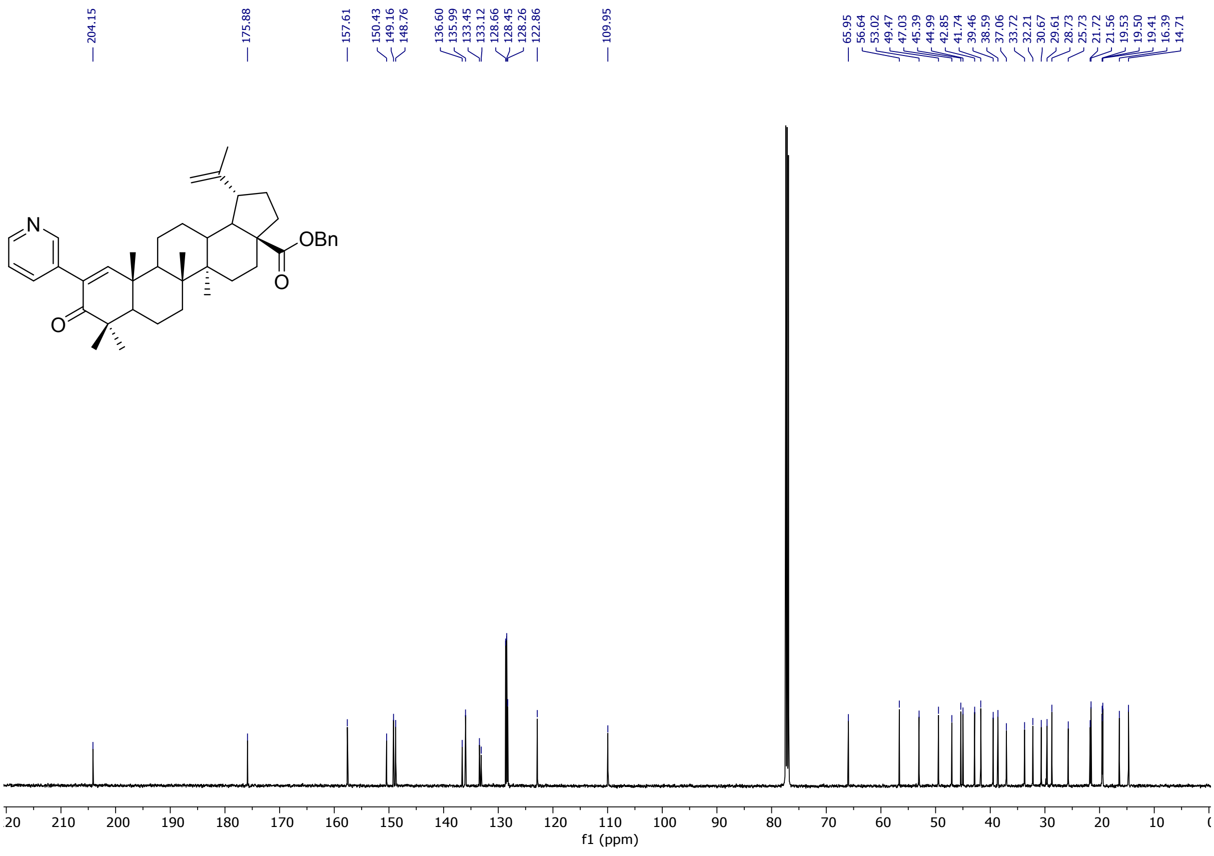

Figure S38. ^{13}C NMR spectrum of the compound **10g** ($CDCl_3$, 126 MHz).

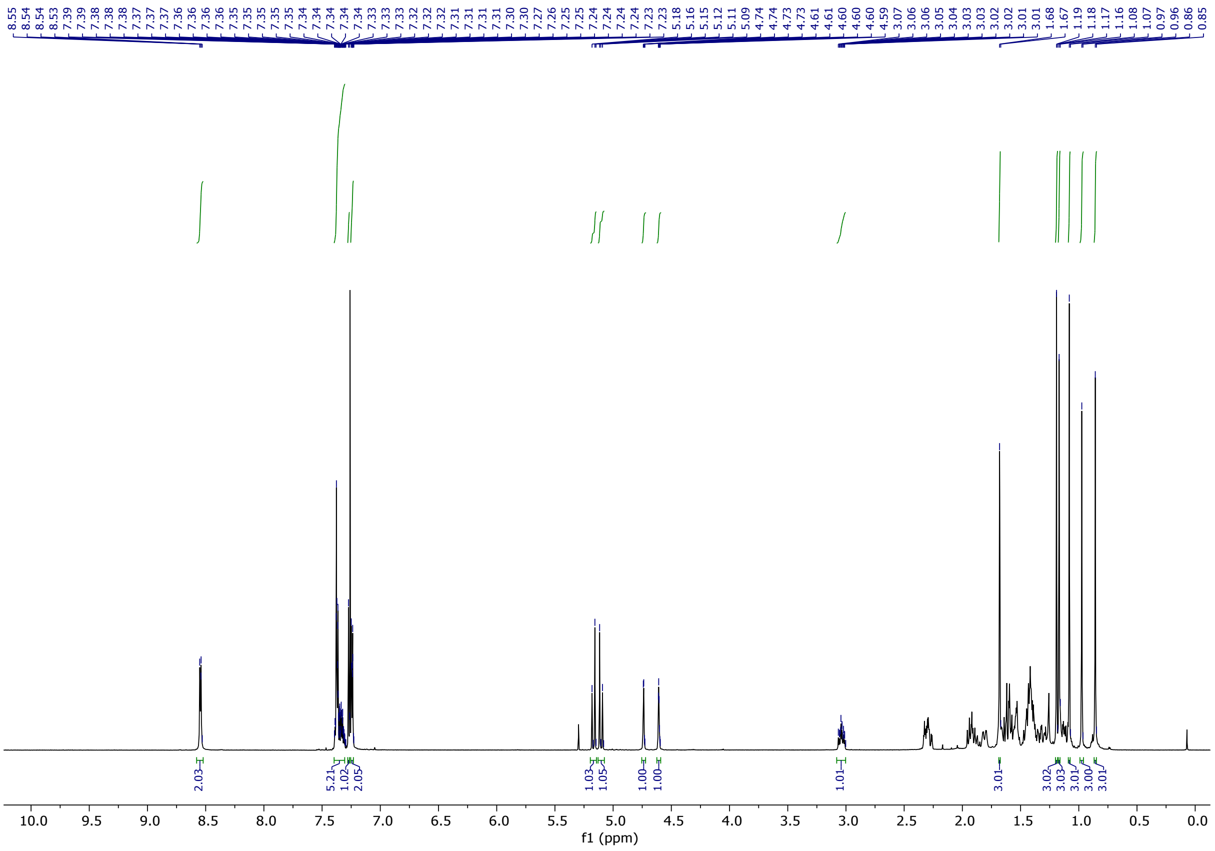

Figure S39. ^{1}H NMR spectrum of the compound **10h** ($CDCl_3$, 500 MHz).

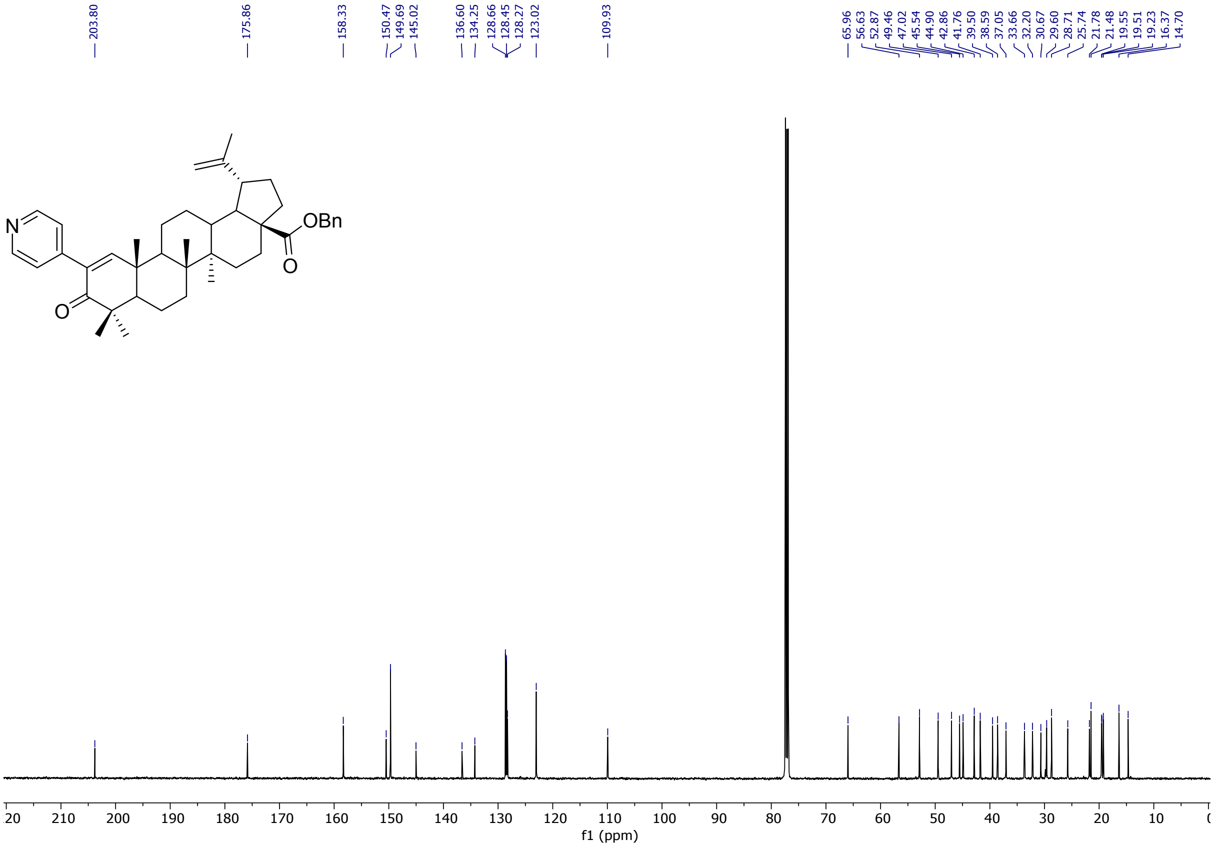

Figure S40. ^{13}C NMR spectrum of the compound **10h** ($CDCl_3$, 126 MHz).

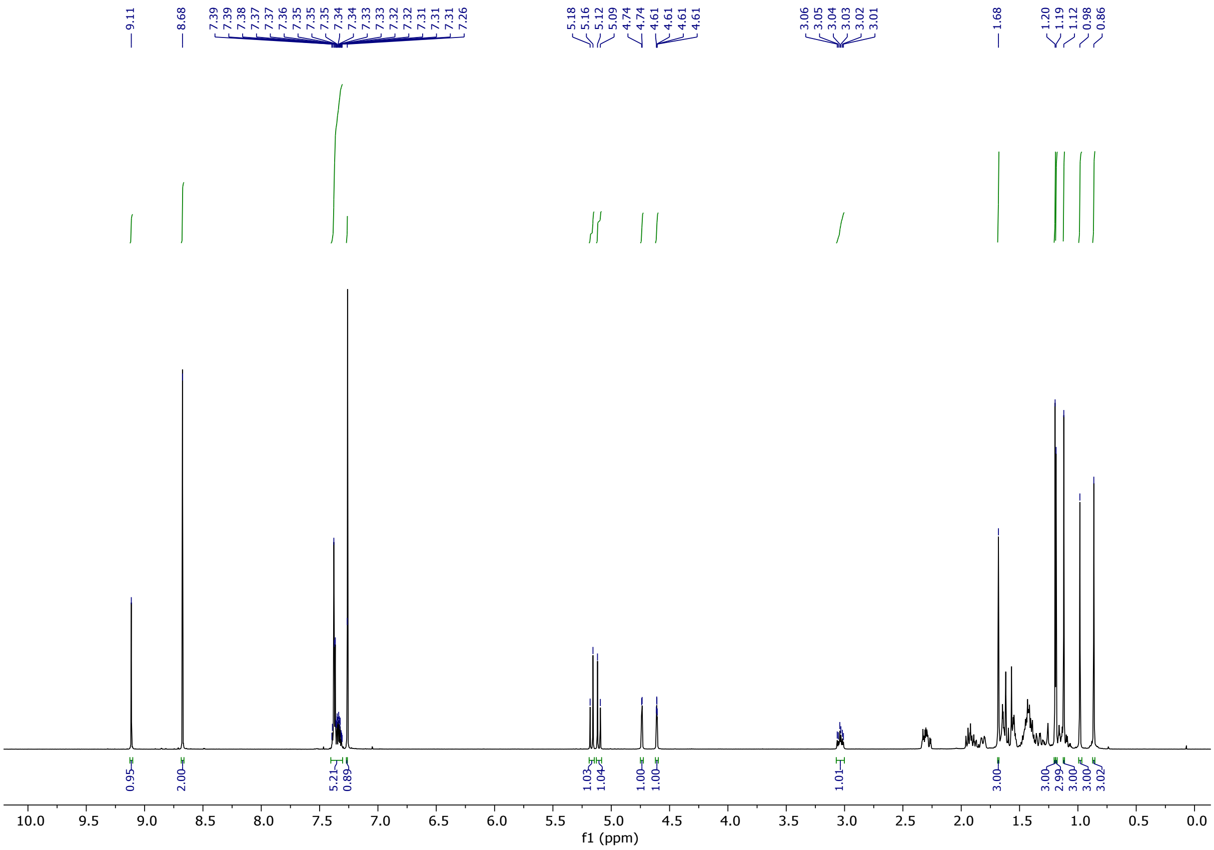

Figure S41. ^{1}H NMR spectrum of the compound **10i** ($CDCl_3$, 500 MHz).

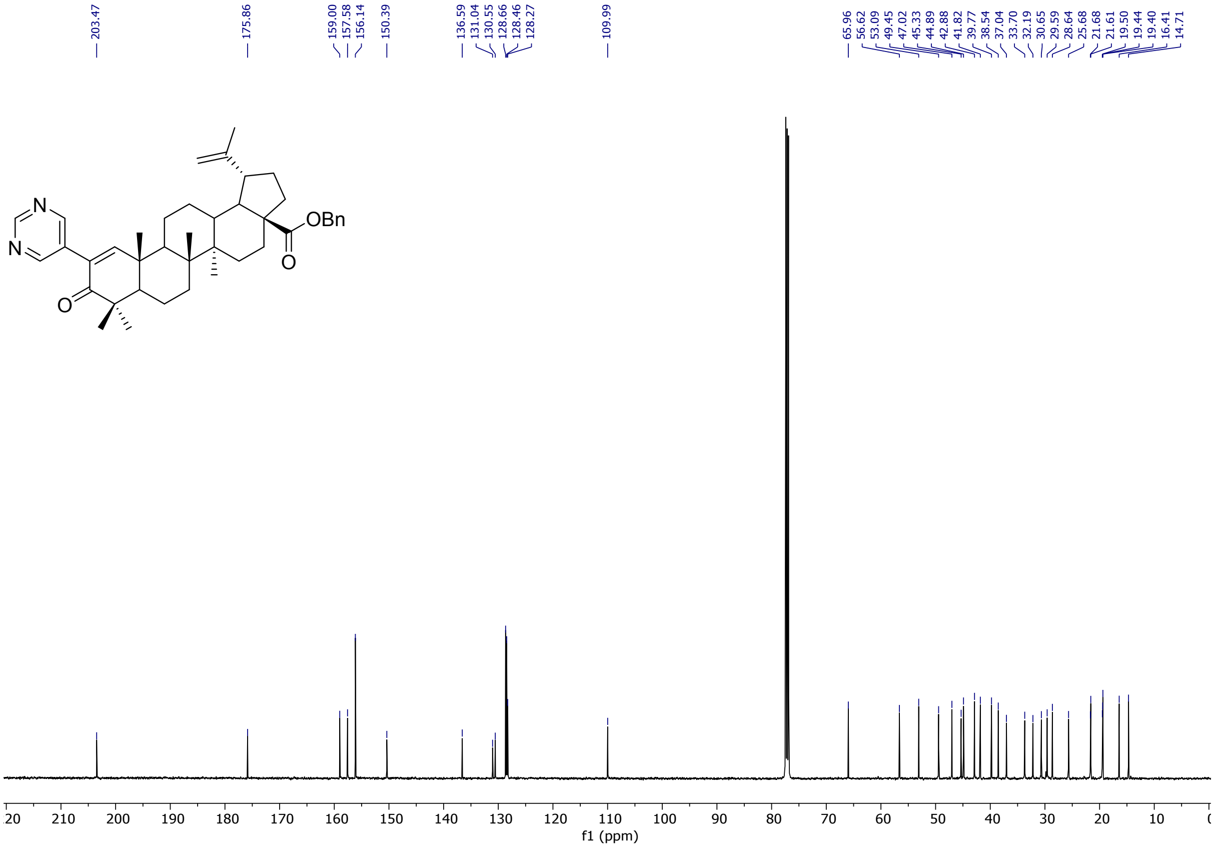

Figure S42. ^{13}C NMR spectrum of the compound **10i** ($CDCl_3$, 126 MHz).

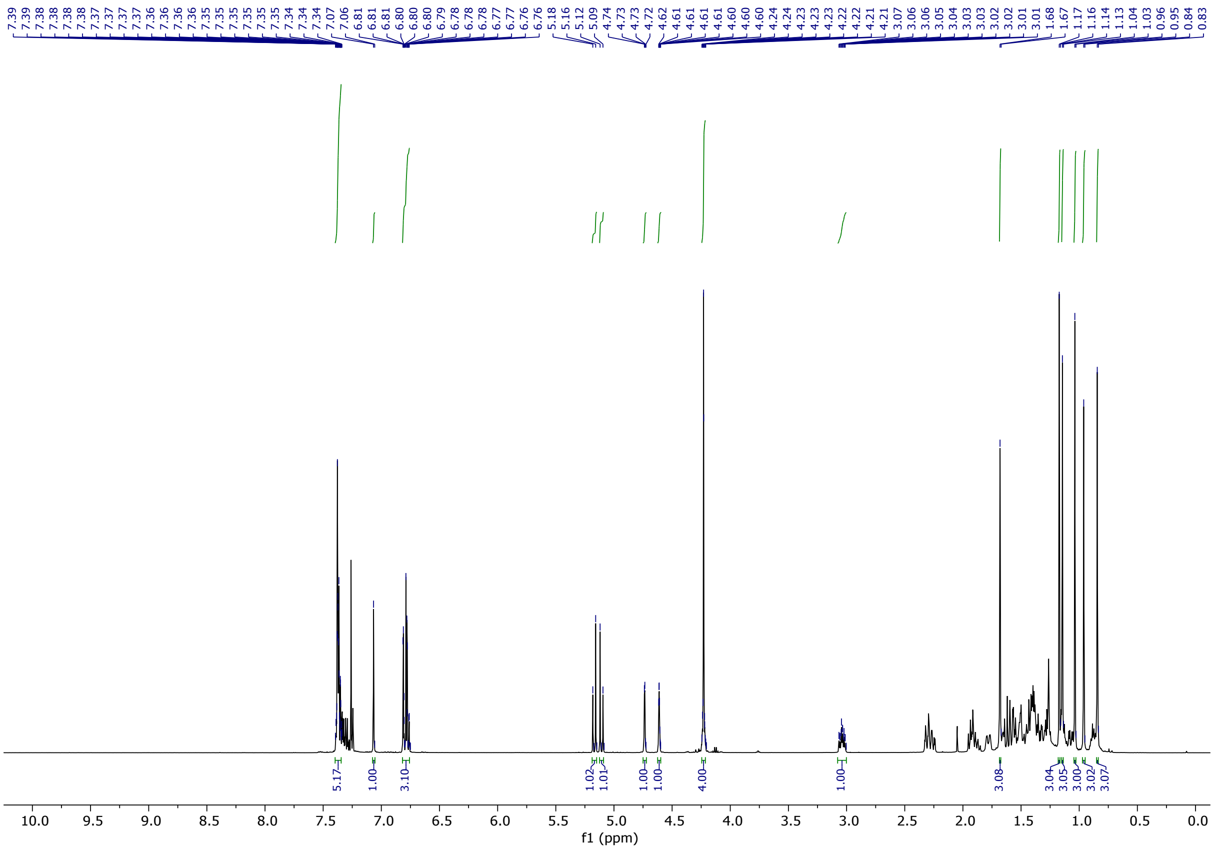

Figure S43. ^{1}H NMR spectrum of the compound **10j** ($CDCl_3$, 500 MHz).

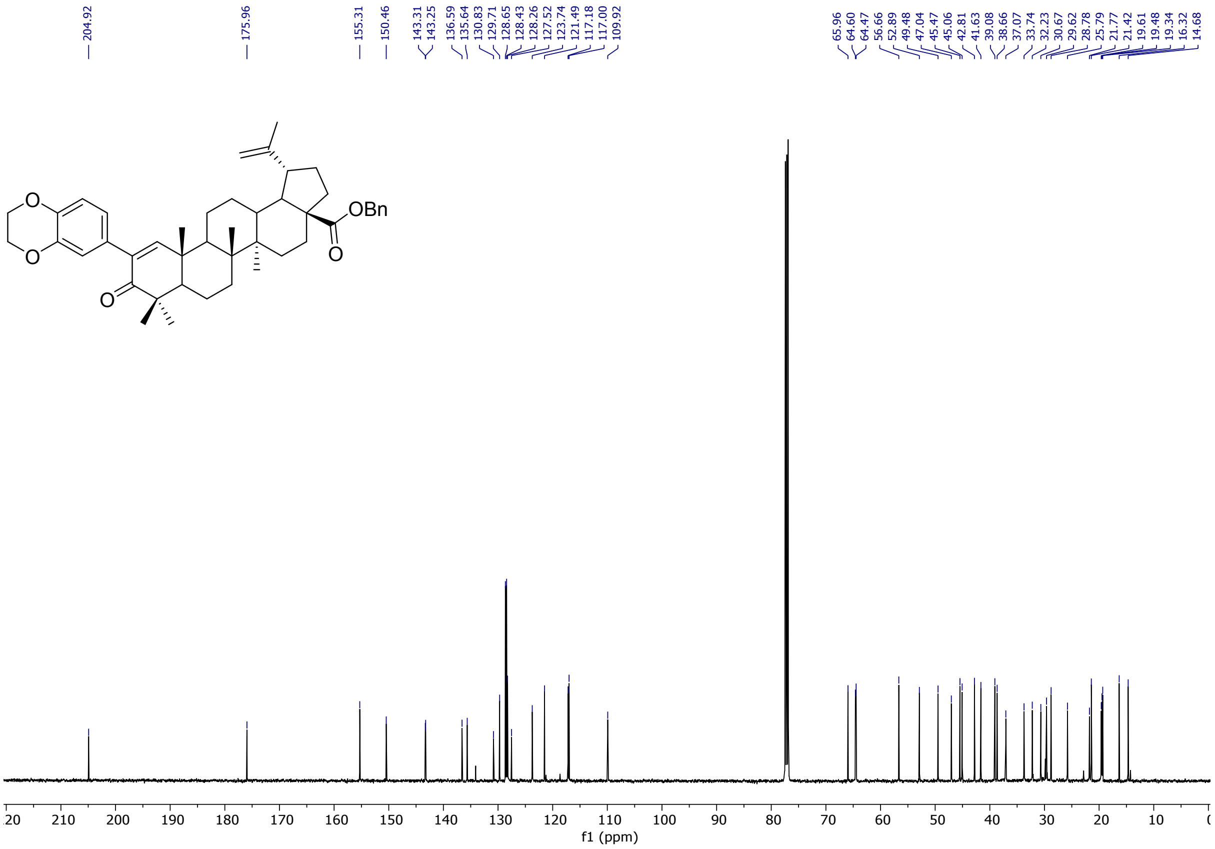

Figure S44. ^{13}C NMR spectrum of the compound **10j** ($CDCl_3$, 126 MHz).

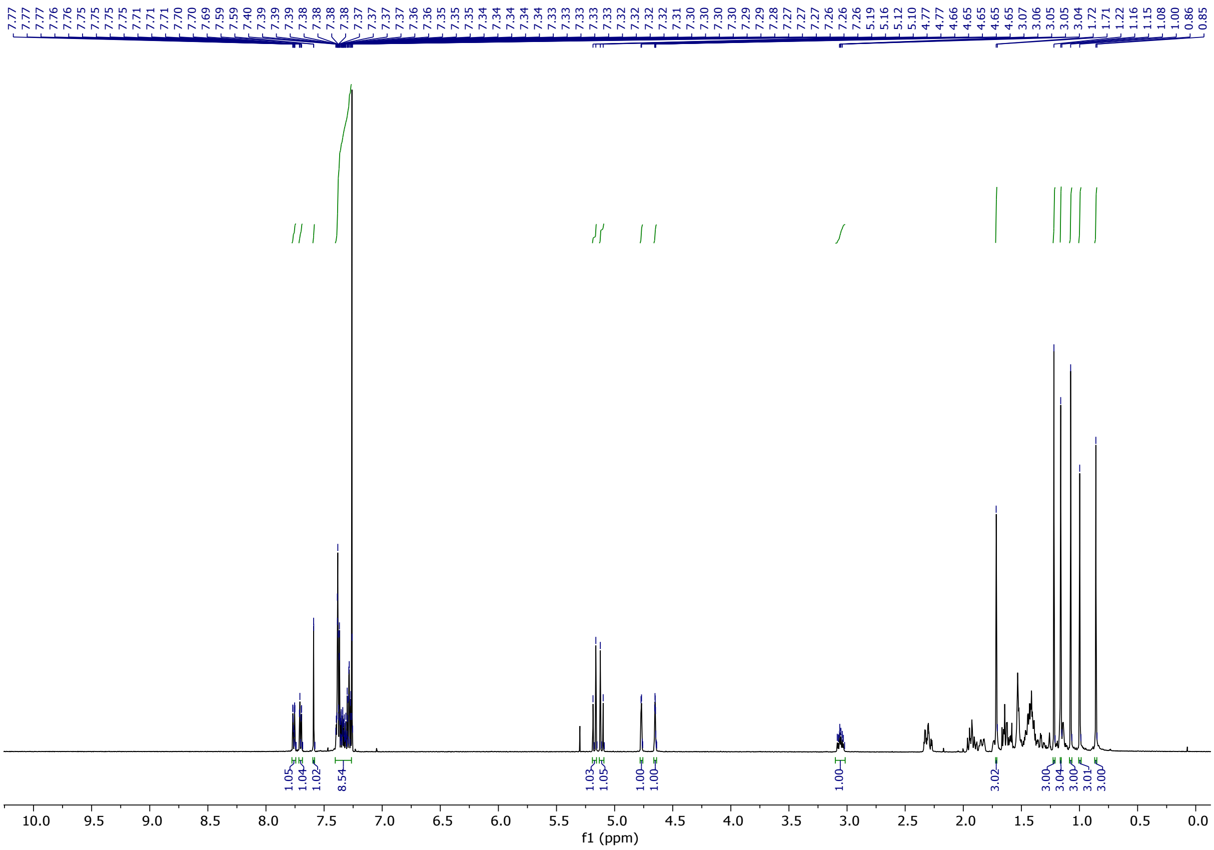

Figure S45. ^{1}H NMR spectrum of the compound **10k** ($CDCl_3$, 500 MHz).

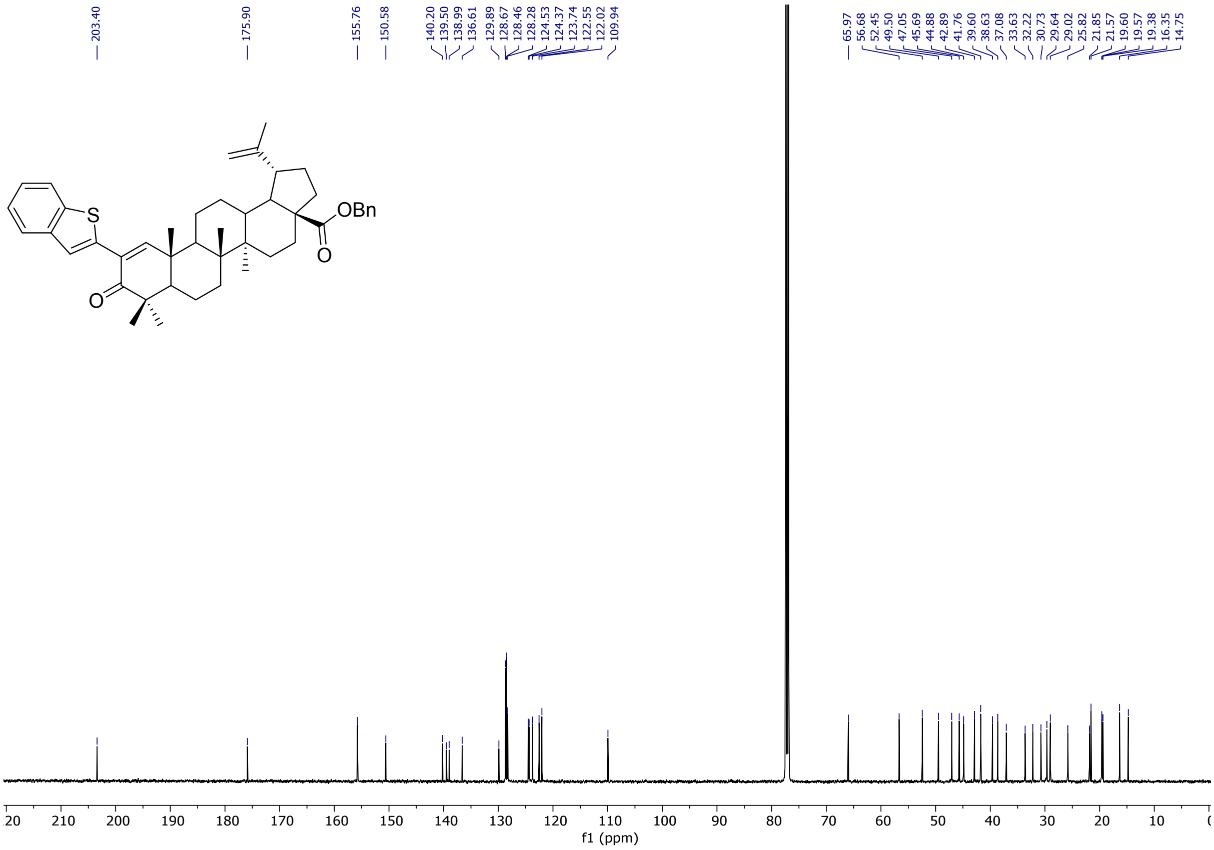

Figure S46. ^{13}C NMR spectrum of the compound **10k** ($CDCl_3$, 126 MHz).

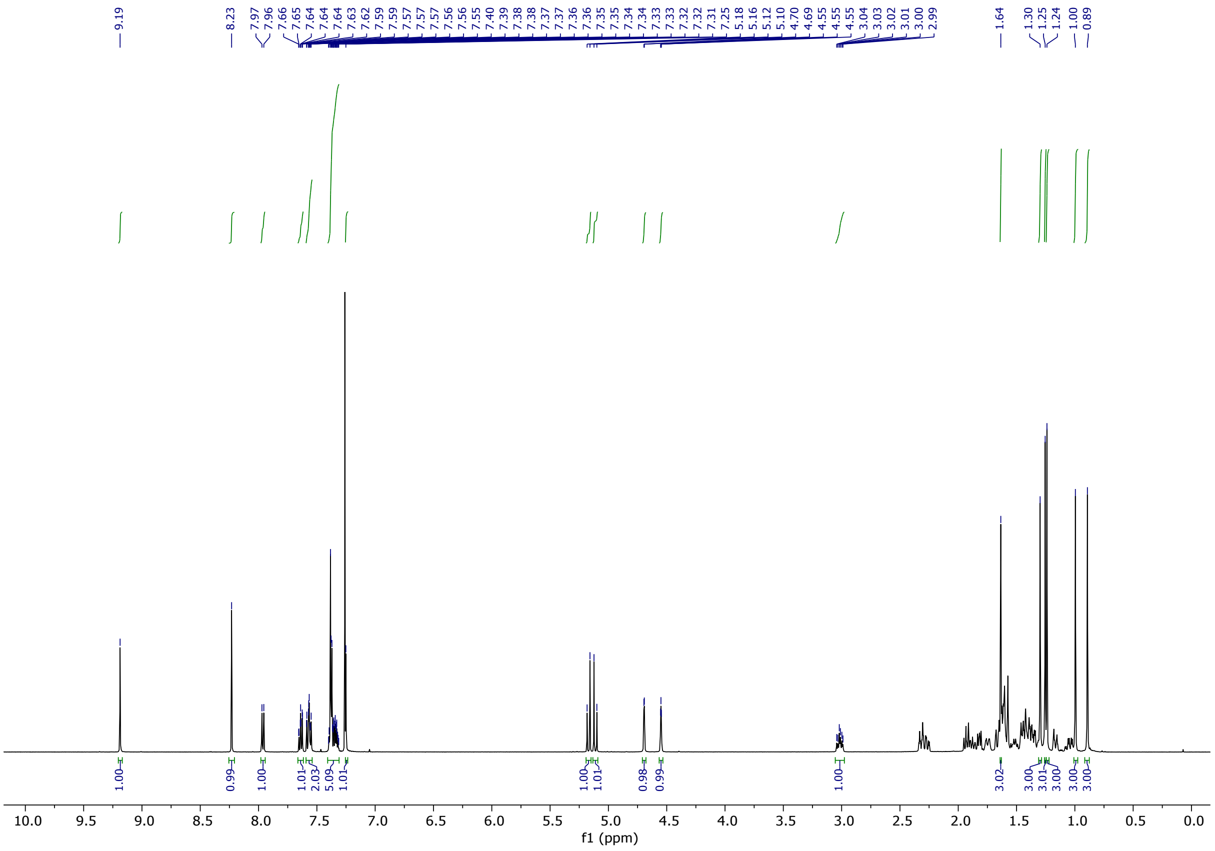

Figure S47. ^{1}H NMR spectrum of the compound **10l** ($CDCl_3$, 500 MHz).

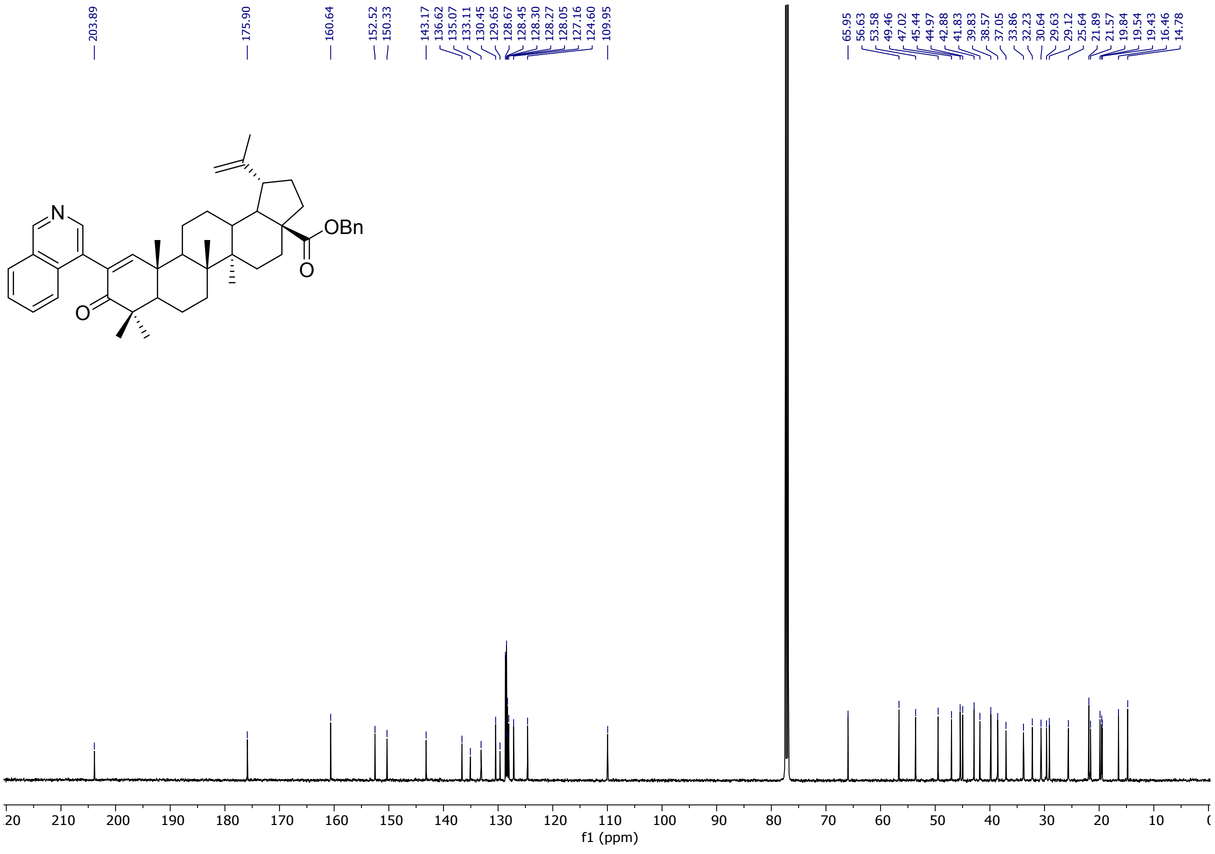

Figure S48. ^{13}C NMR spectrum of the compound **10l** ($CDCl_3$, 126 MHz).

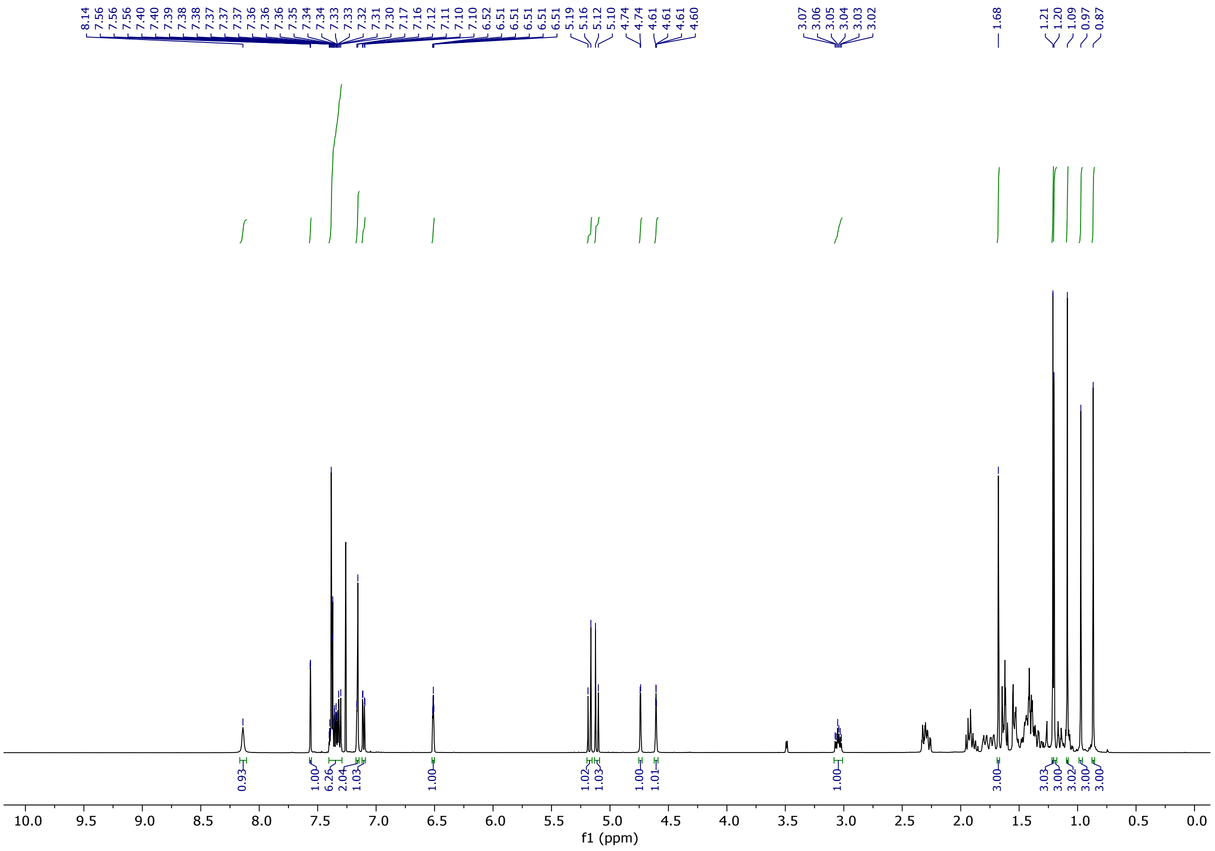

Figure S49. ^{1}H NMR spectrum of the compound **10m** ($CDCl_3$, 500 MHz).

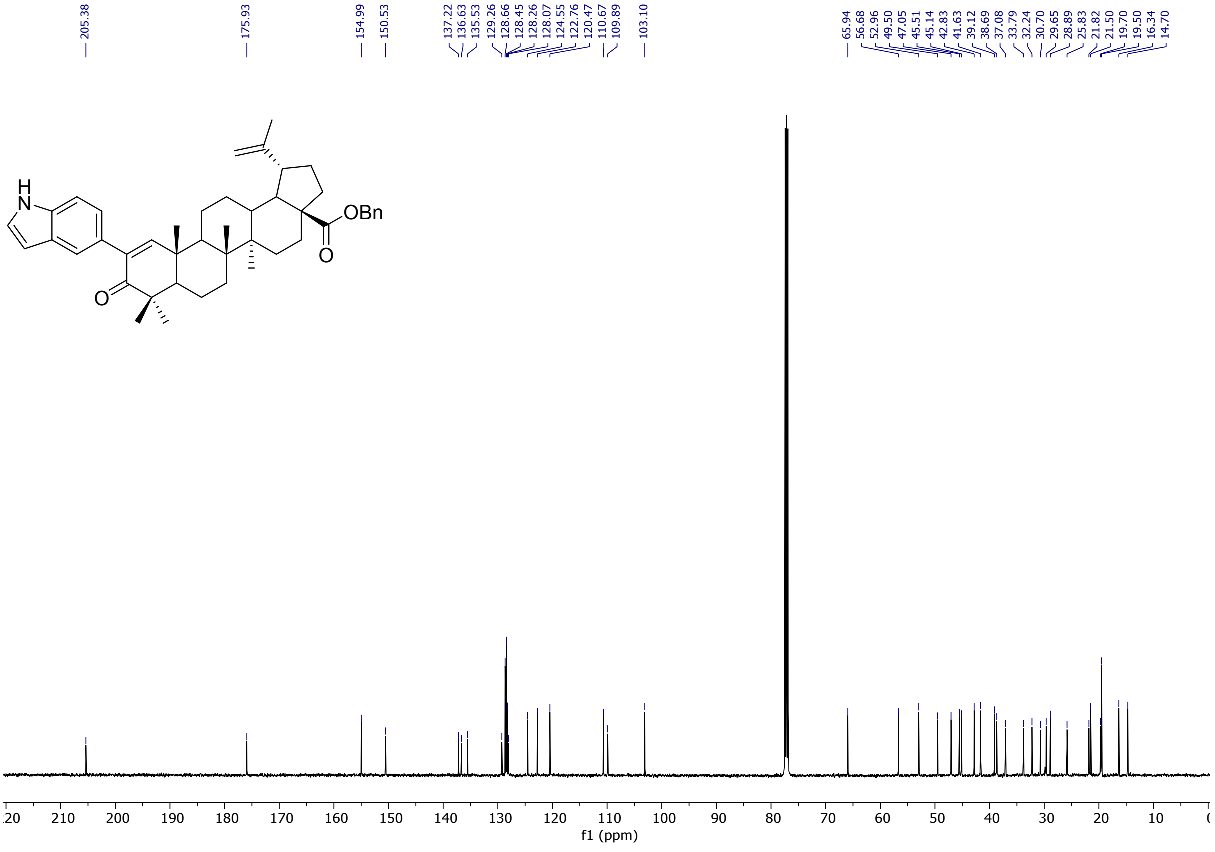

Figure S50. ^{13}C NMR spectrum of the compound **10m** ($CDCl_3$, 126 MHz).

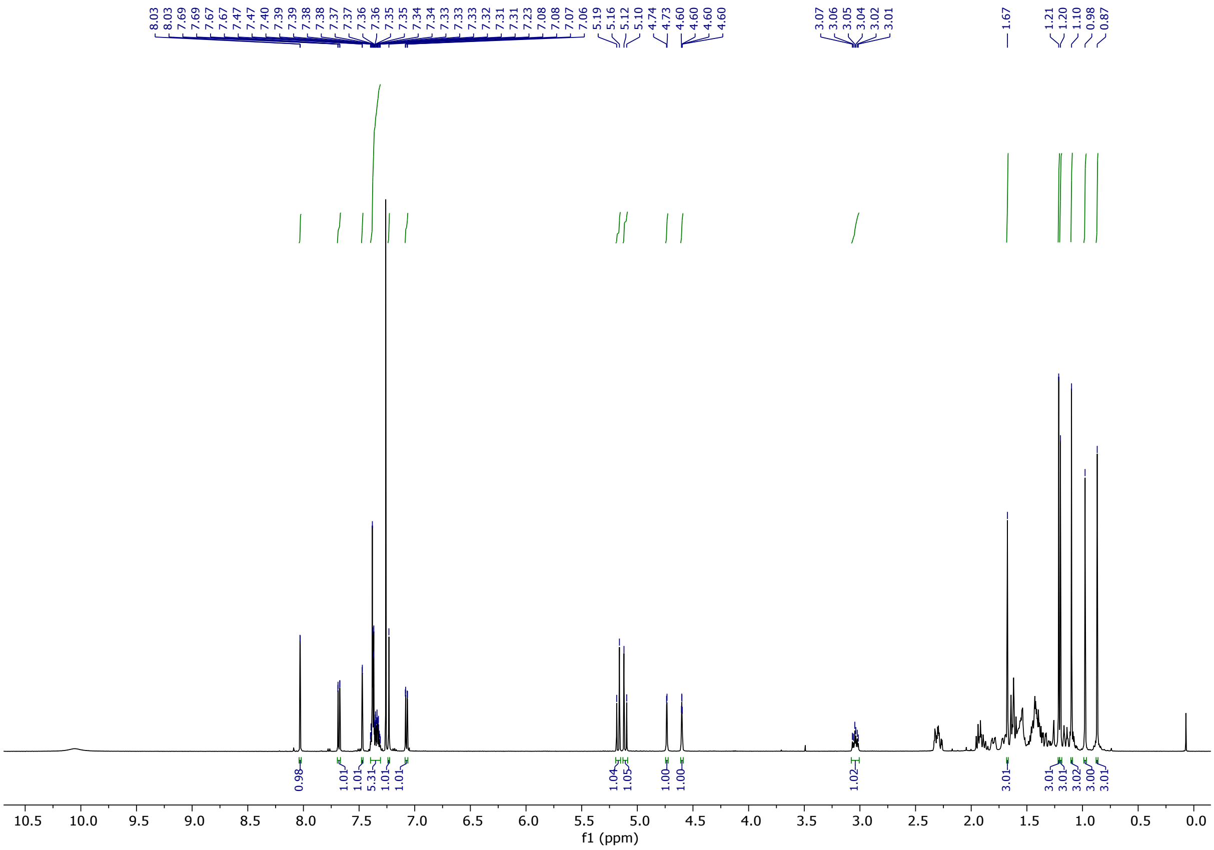

Figure S51. ^{1}H NMR spectrum of the compound **10n** ($CDCl_3$, 500 MHz).

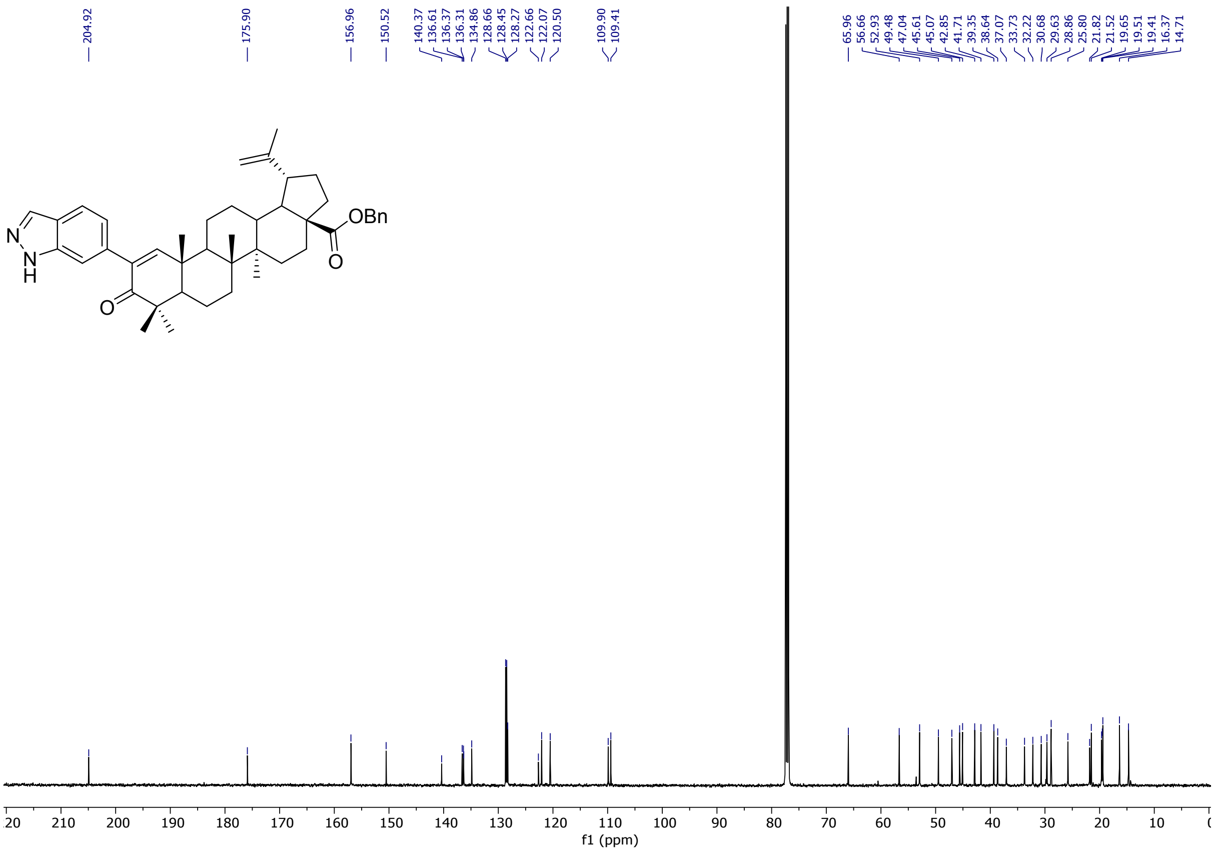

Figure S52. ^{13}C NMR spectrum of the compound **10n** ($CDCl_3$, 126 MHz).

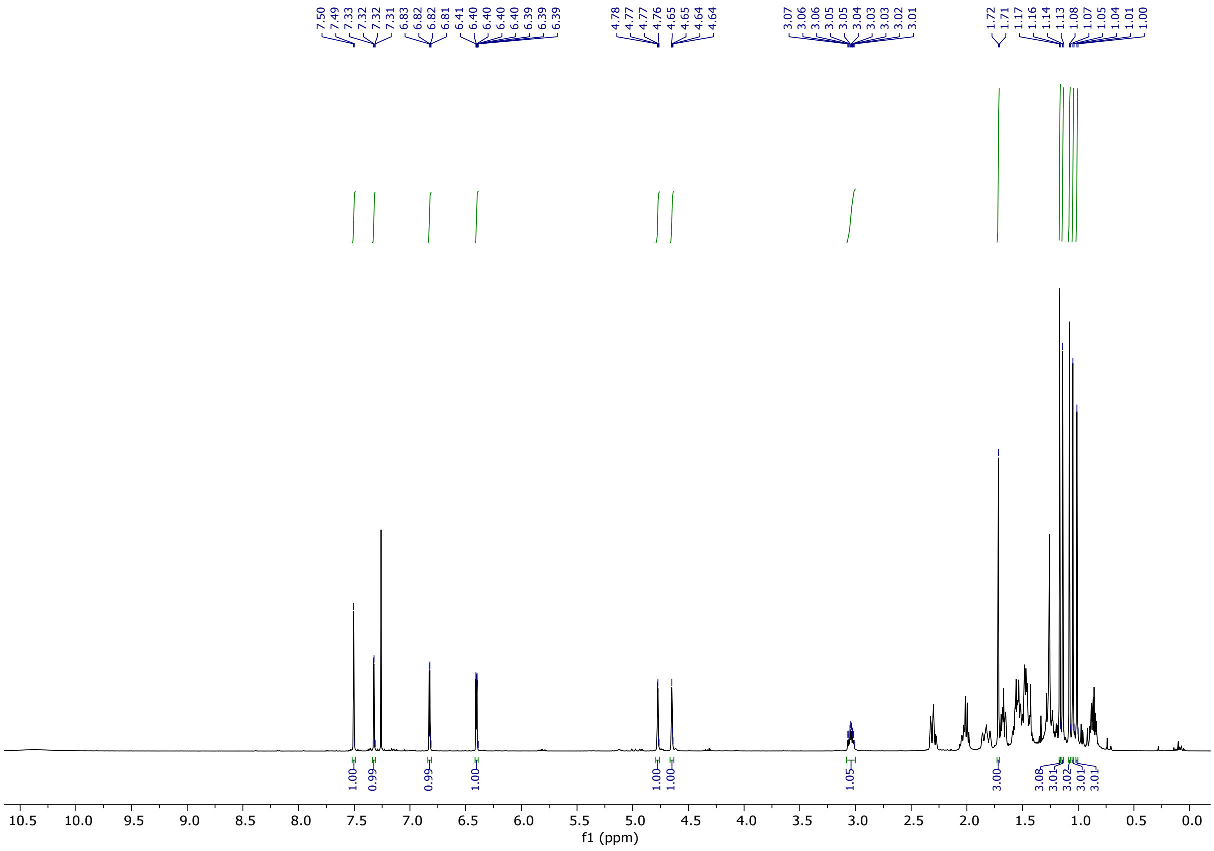

Figure S53. ^{1}H NMR spectrum of the compound **11a** ($CDCl_3$, 500 MHz).

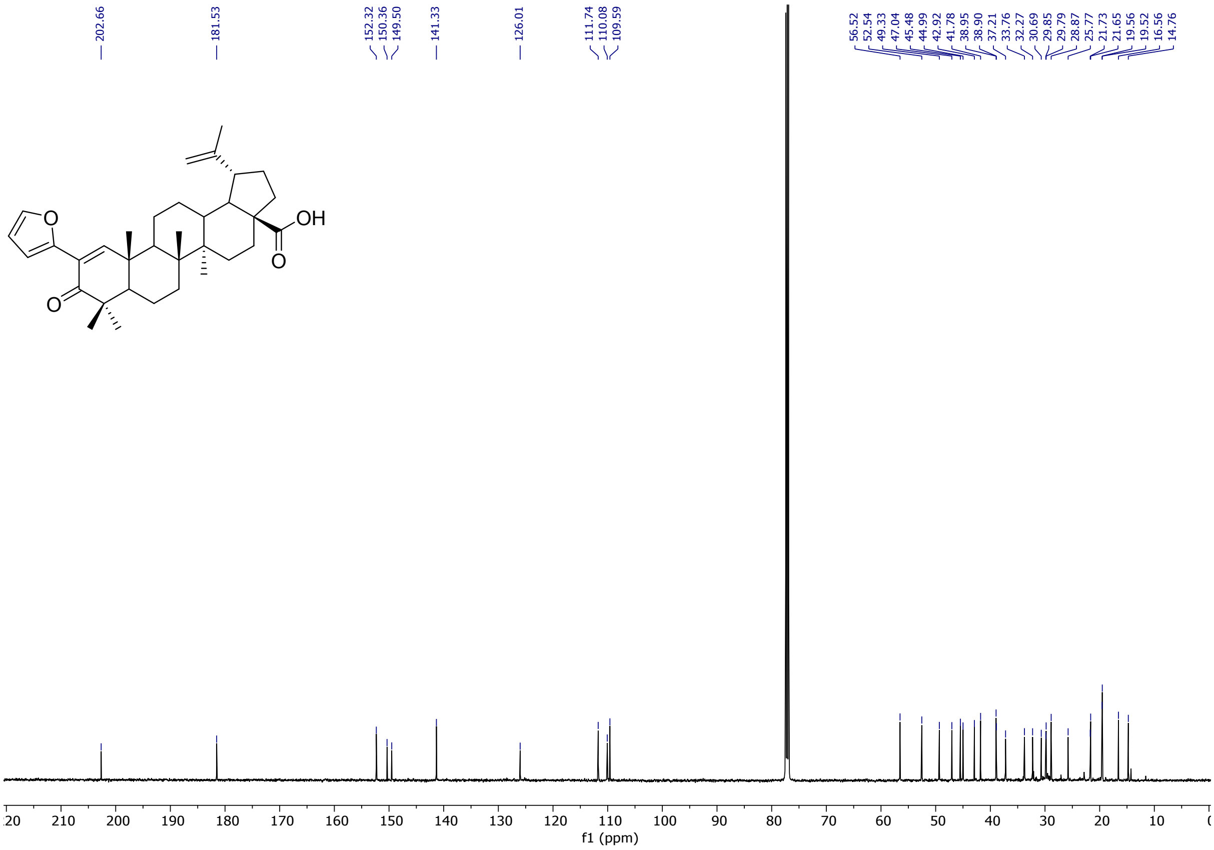

Figure S54. ^{13}C NMR spectrum of the compound **11a** ($CDCl_3$, 126 MHz).

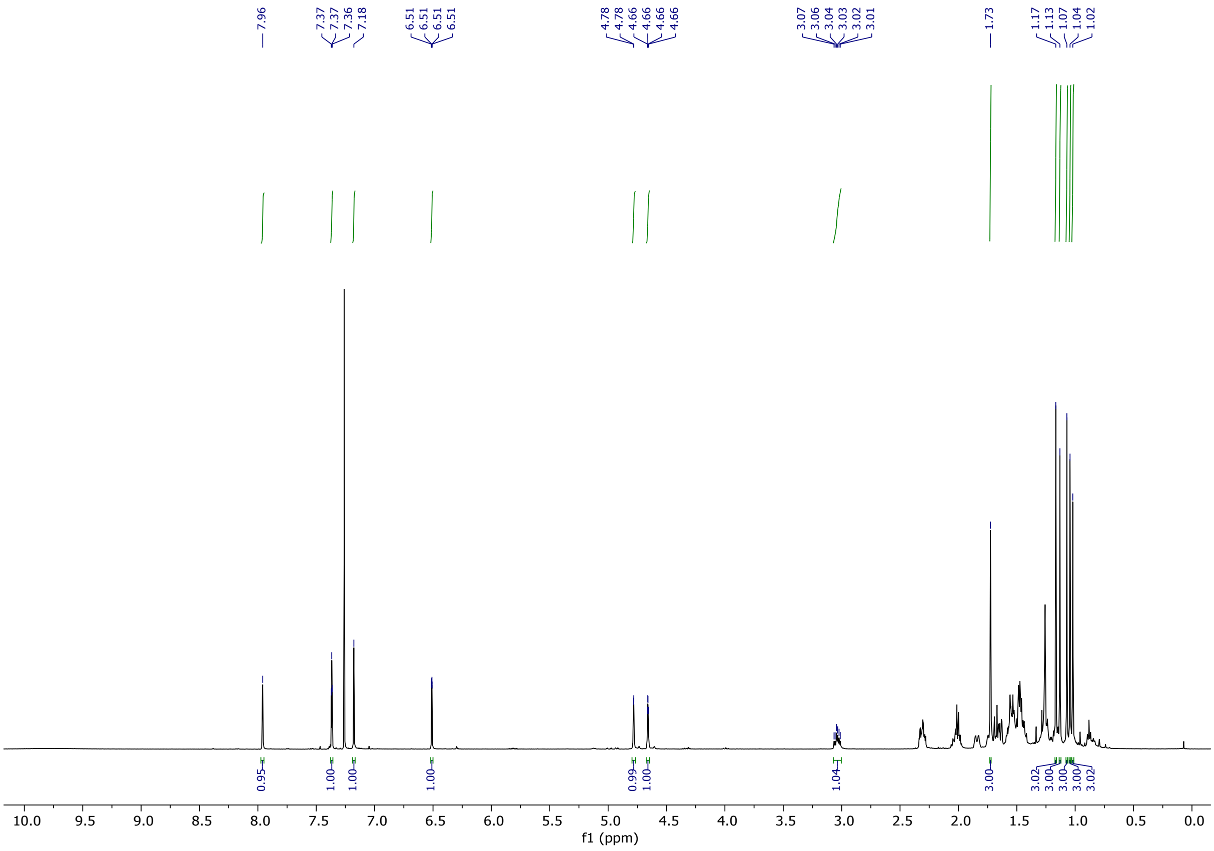

Figure S55. ^{1}H NMR spectrum of the compound **11b** ($CDCl_3$, 500 MHz).

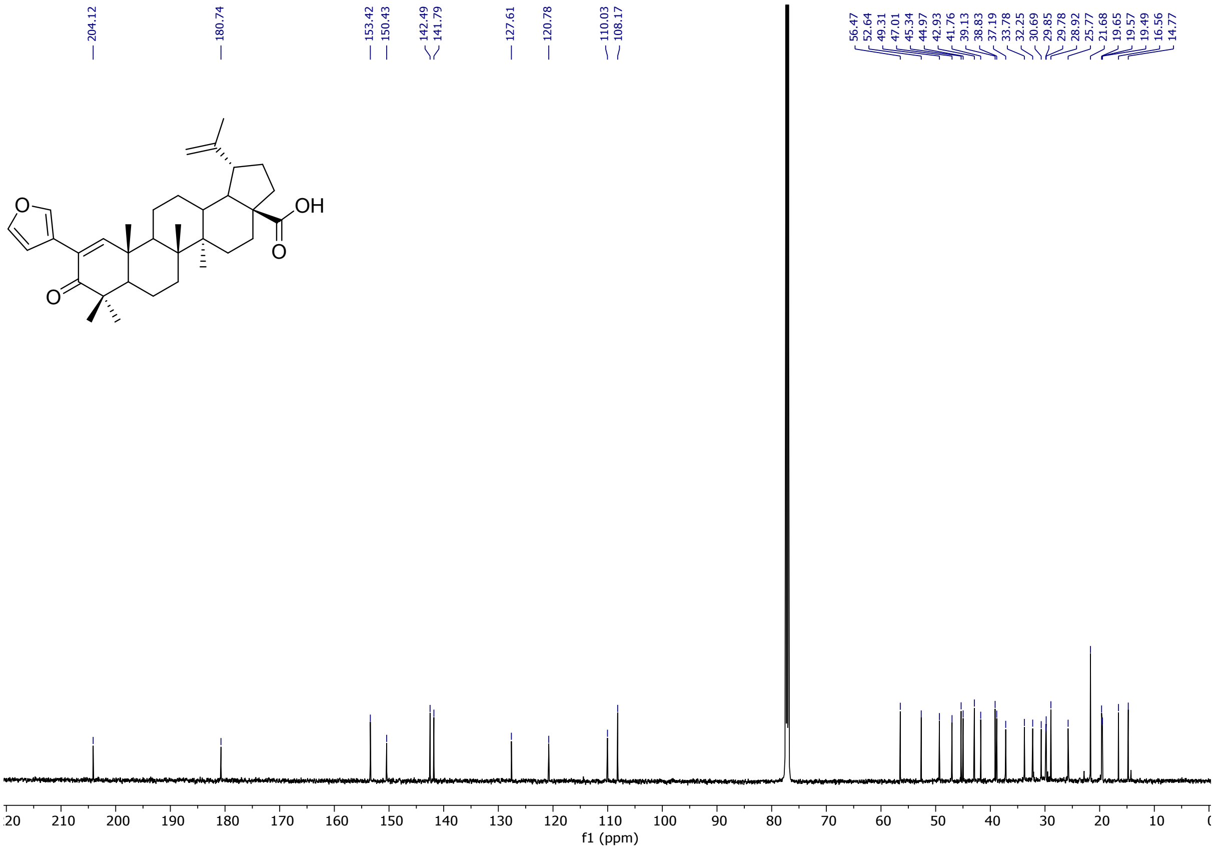

Figure S56. ^{13}C NMR spectrum of the compound **11b** ($CDCl_3$, 126 MHz).

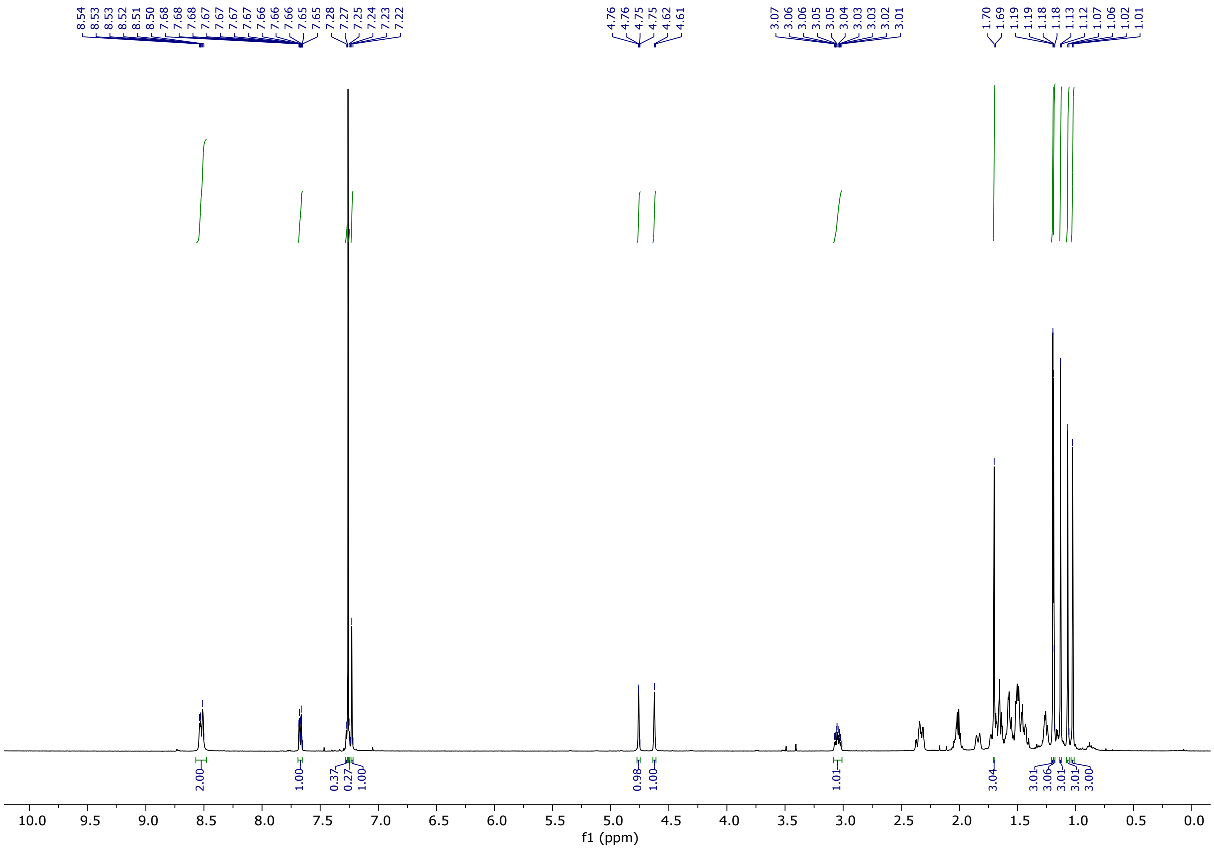

Figure S57. ^{1}H NMR spectrum of the compound **11g** ($CDCl_3$, 500 MHz).

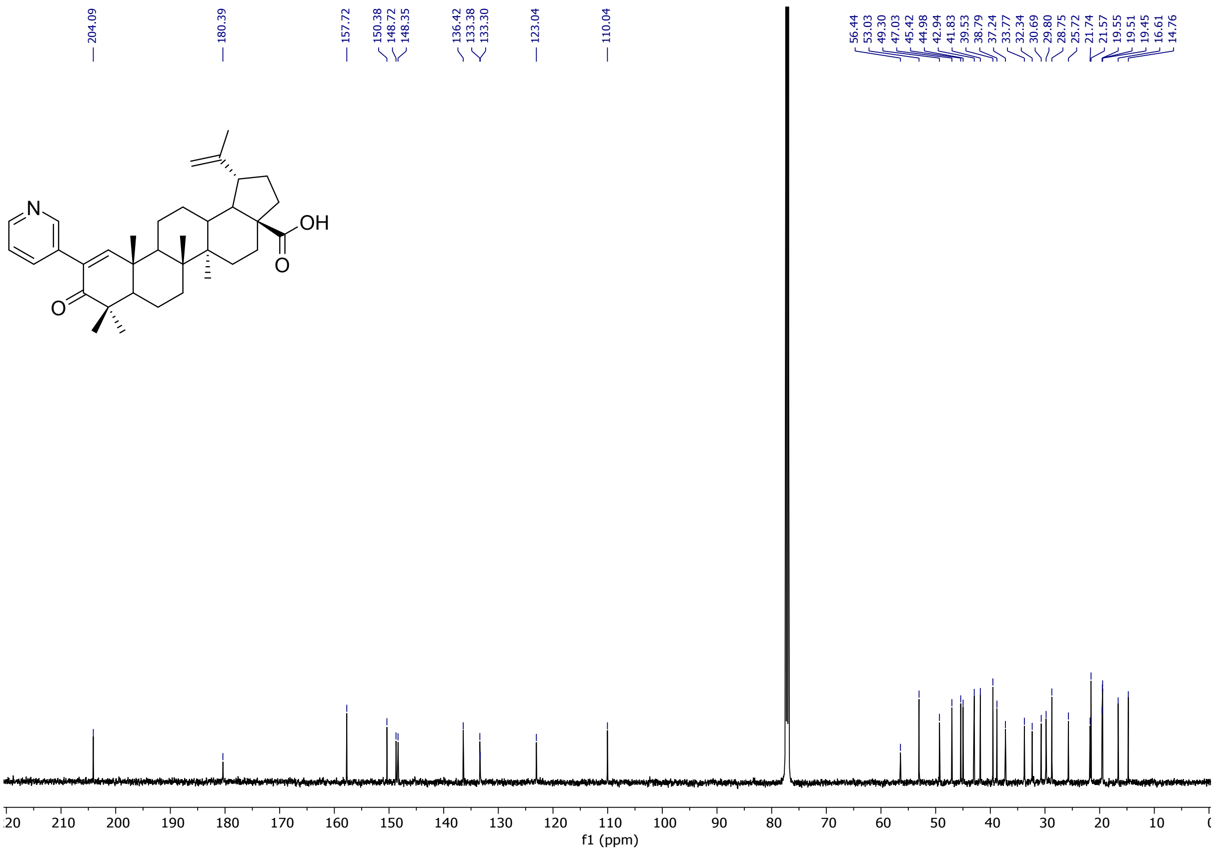

Figure S58. ^{13}C NMR spectrum of the compound **11g** ($CDCl_3$, 126 MHz).

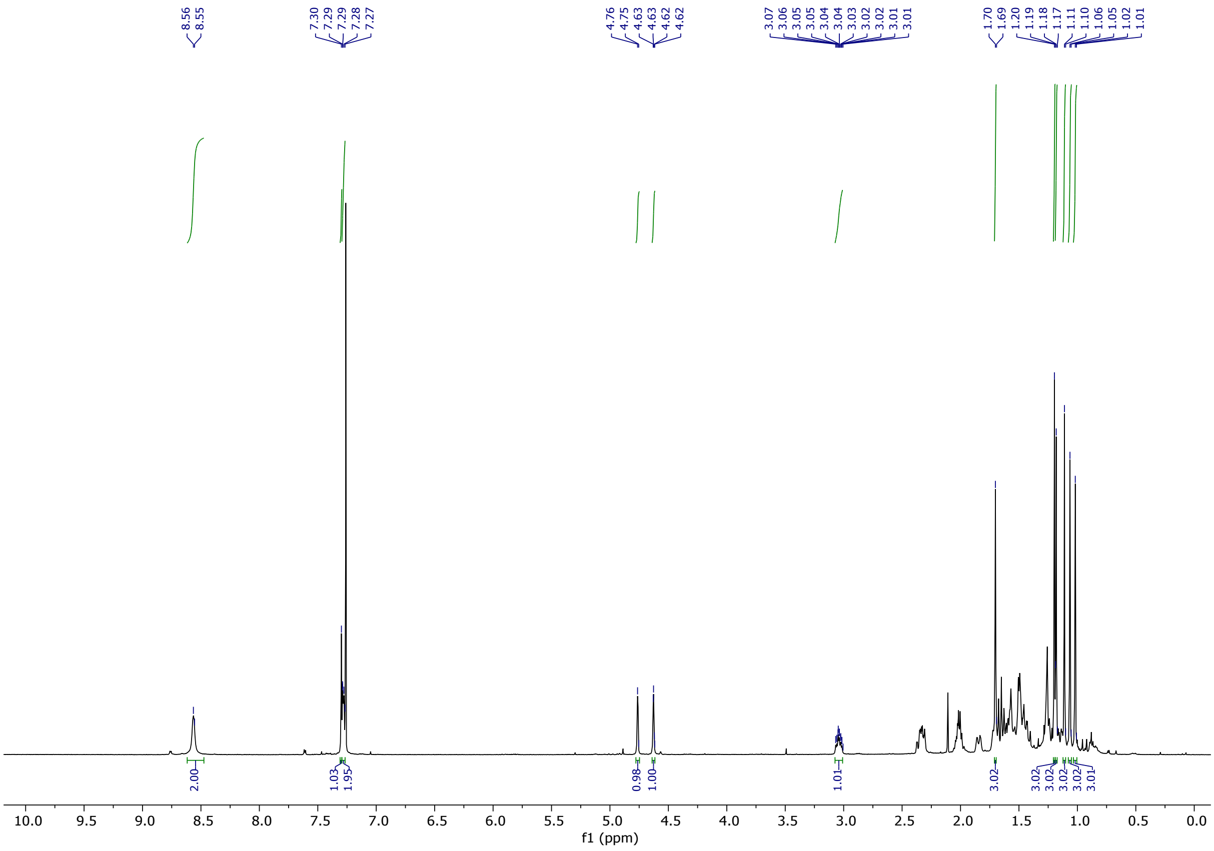

Figure S59. ^{1}H NMR spectrum of the compound **11h** ($CDCl_3$, 500 MHz).

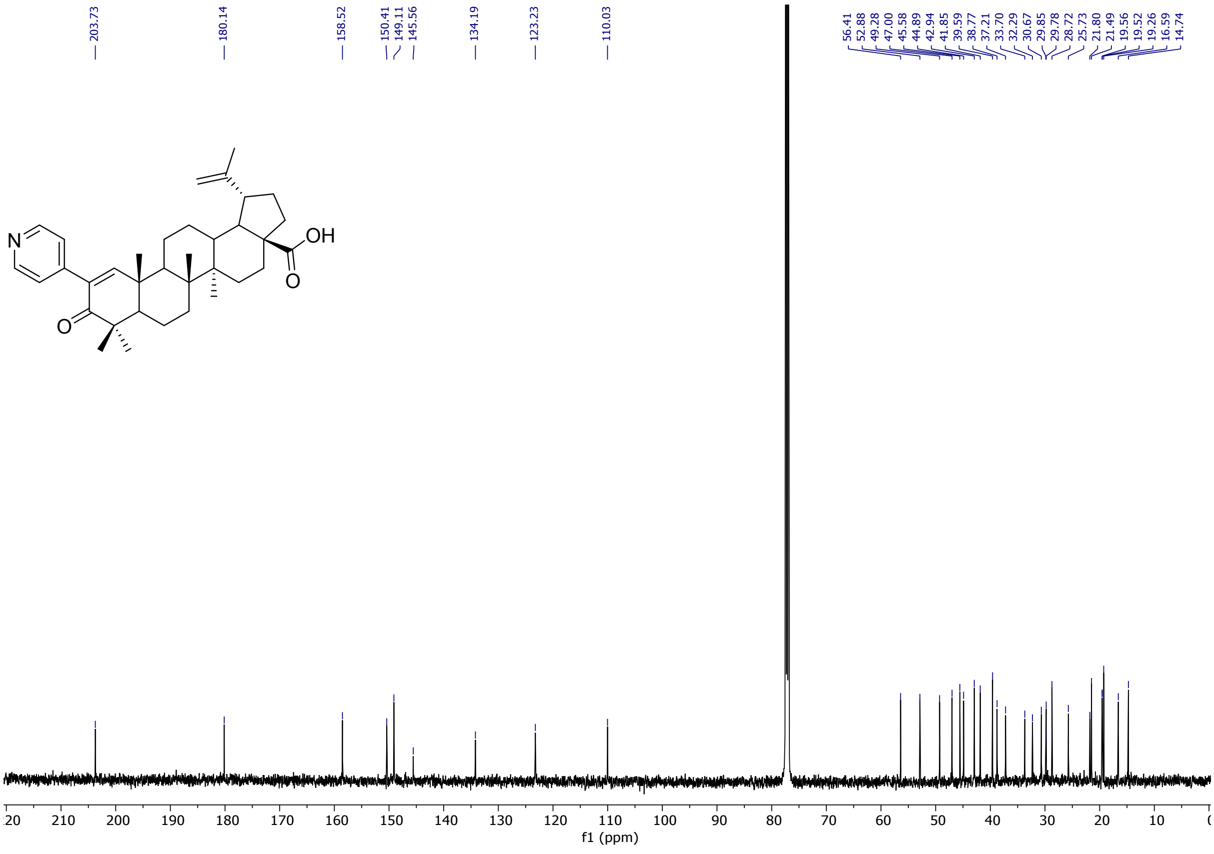

Figure S60. ^{13}C NMR spectrum of the compound **11h** ($CDCl_3$, 126 MHz).

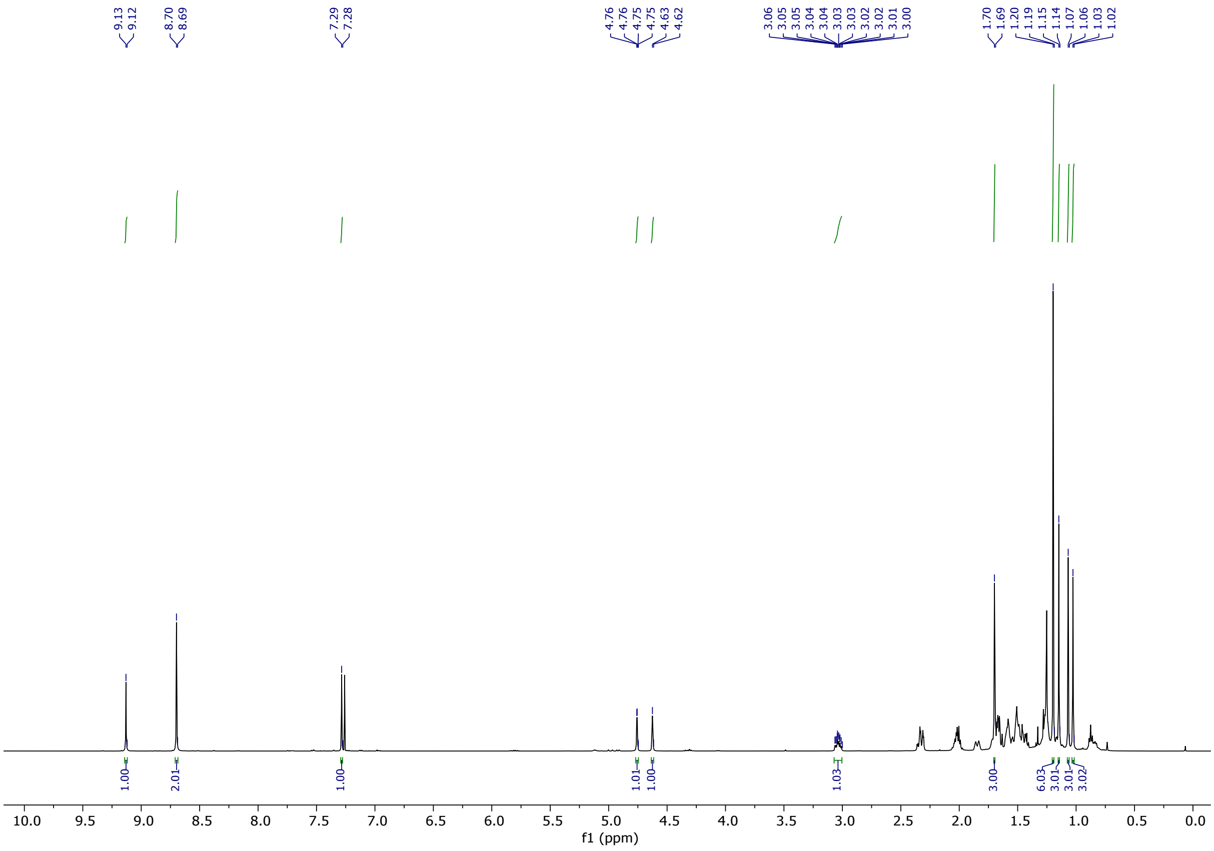

Figure S61. ^{1}H NMR spectrum of the compound **11i** ($CDCl_3$, 500 MHz).

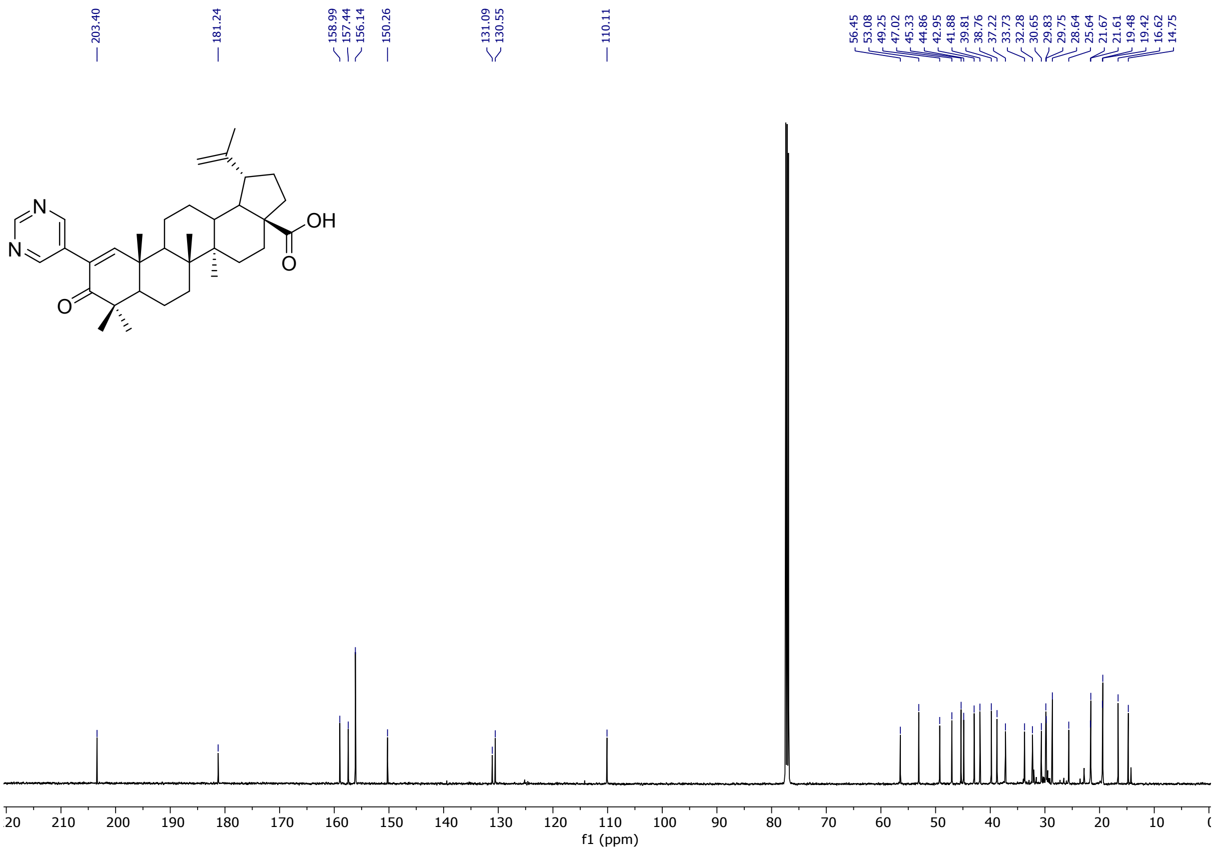

Figure S62. ^{13}C NMR spectrum of the compound **11i** ($CDCl_3$, 126 MHz).

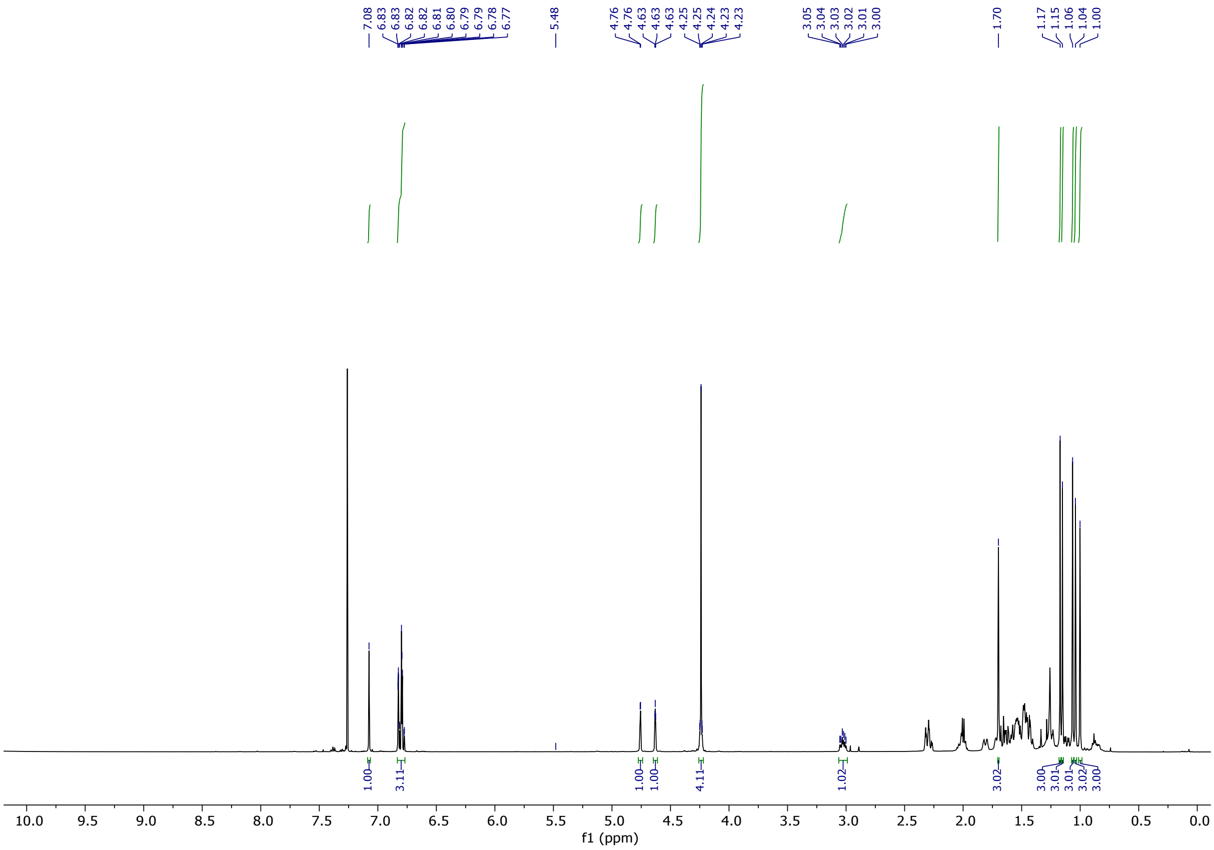

Figure S63. ^{1}H NMR spectrum of the compound **11j** ($CDCl_3$, 500 MHz).

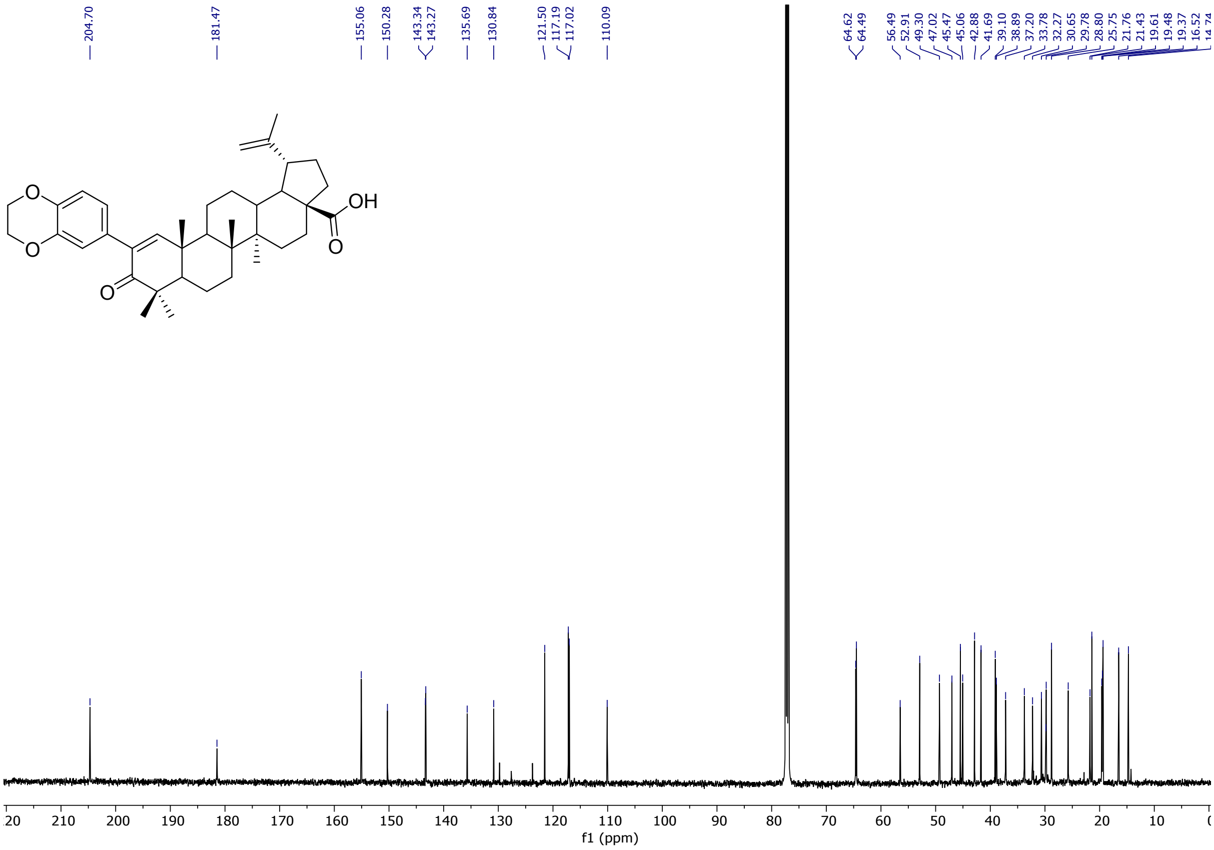

Figure S64. ^{13}C NMR spectrum of the compound **11j** ($CDCl_3$, 126 MHz).

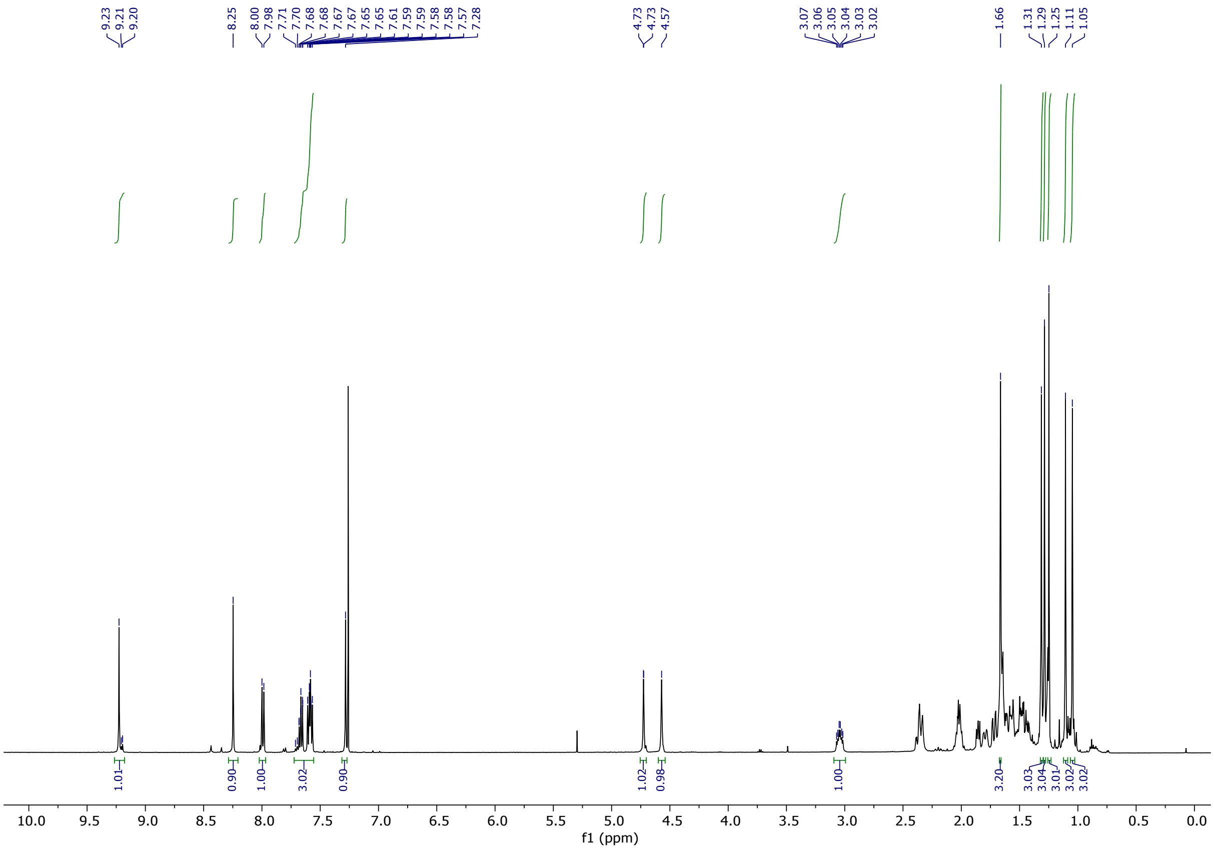

Figure S65. ^{1}H NMR spectrum of the compound **11l** ($CDCl_3$, 500 MHz).

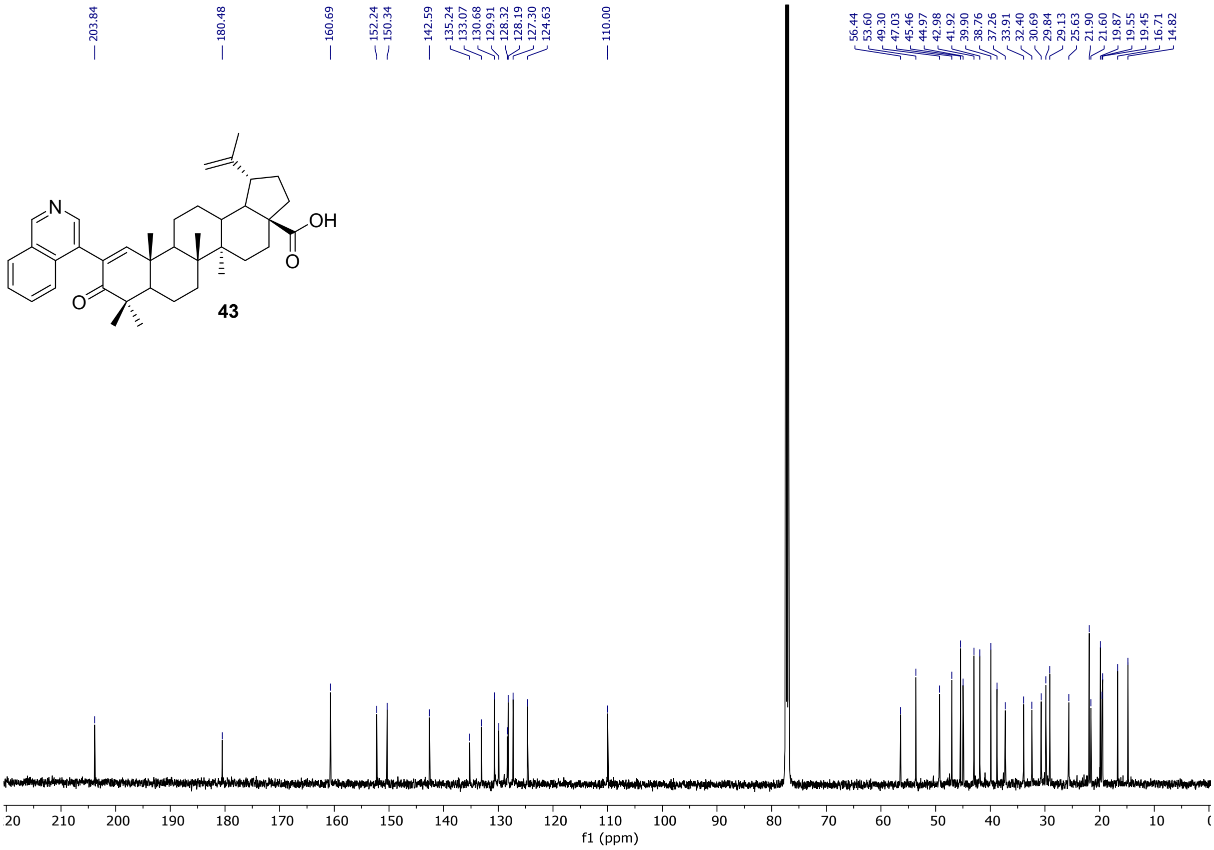

Figure S66. ^{13}C NMR spectrum of the compound **11l** ($CDCl_3$, 126 MHz).

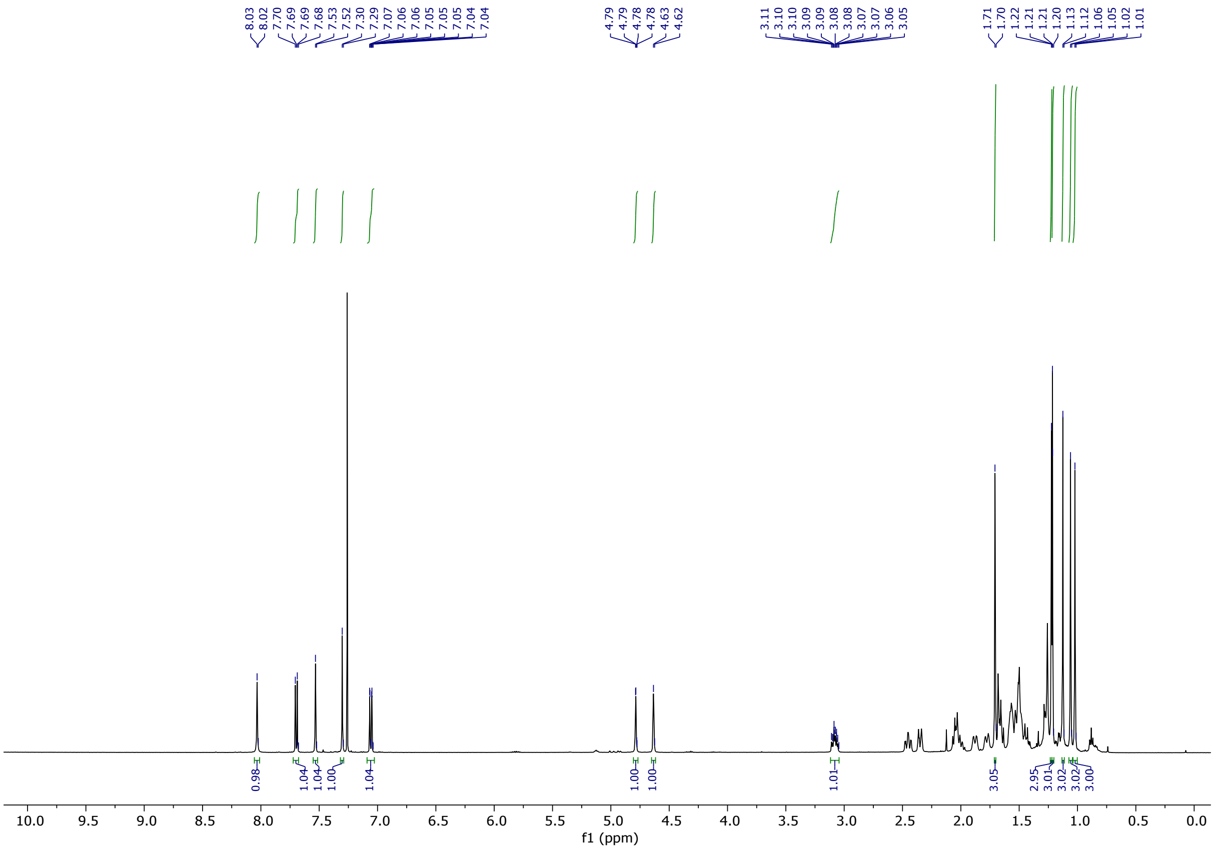

Figure S67. ^{1}H NMR spectrum of the compound **11n** ($CDCl_3$, 500 MHz).

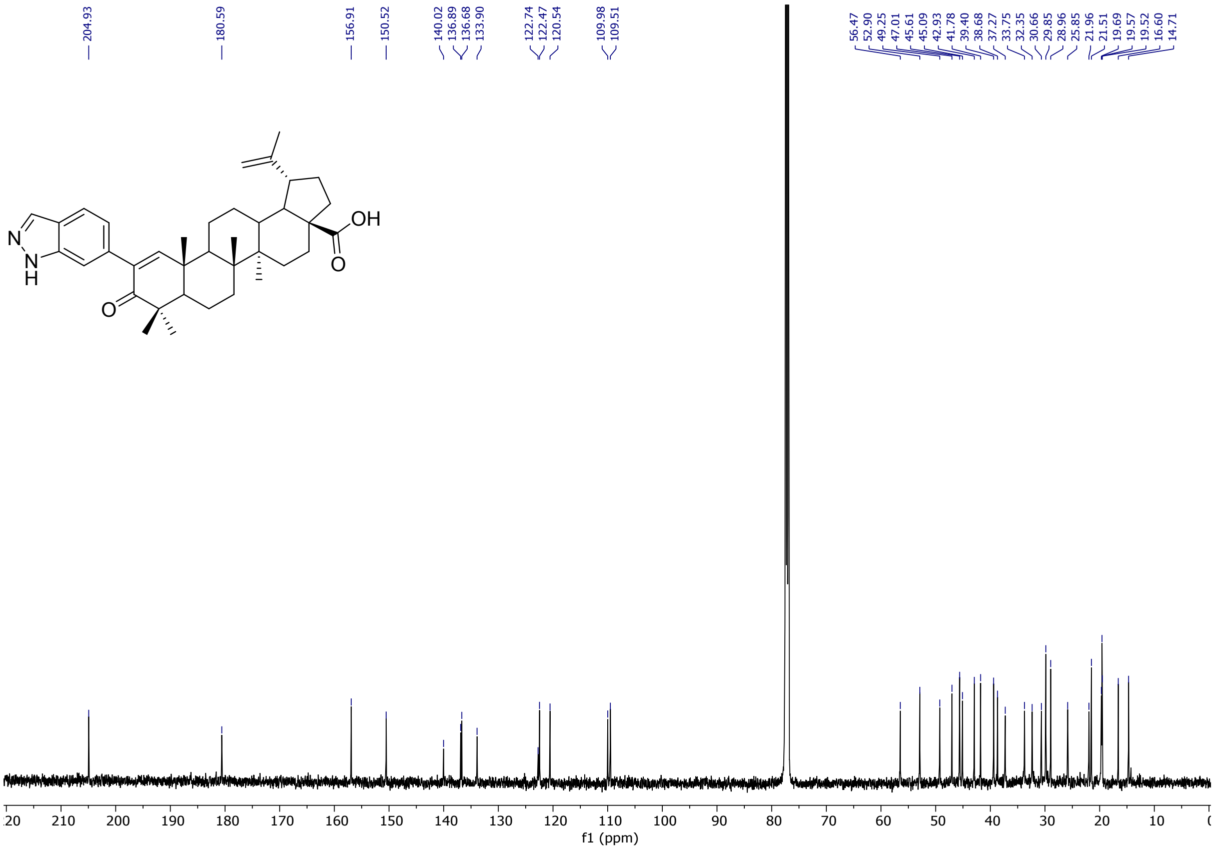

Figure S68. ^{13}C NMR spectrum of the compound **11n** ($CDCl_3$, 126 MHz).

Table S1. Full table of all measured cytotoxic activities in all used cancer cells.

**Comp.**	**IC$_{50}$ (μmol/L)[a]**											
	**A549**	**DU-145**	**K562**	**K562-TAX**	**HCT 116**	**HCT116 p53$^{-/-}$**	**U2OS**	**CCRF-CEM**	**CEM-DNR**	**BJ**	**MRC-5**	**SI[b]**
**4a**	>50	>50	>50	>50	>50	>50	>50	>50	>50	>50	>50	>1.0
**4b**	>50	>50	>50	>50	>50	>50	>50	>50	>50	>50	>50	>1.0
**4c**	>50	>50	>50	>50	>50	>50	>50	>50	>50	>50	>50	>1.0
**4d**	>50	>50	>50	>50	>50	>50	>50	>50	>50	>50	>50	>1.0
**4e**	>50	>50	>50	>50	>50	>50	>50	>50	>50	>50	>50	>1.0
**4g**	>50	43.3	>50	19	>50	>50	>50	25.4	15.5	>50	>50	>2.0
**4h**	>50	22.1	>50	18.1	>50	>50	>50	18.7	19	>50	>50	>2.7
**4i**	>50	>50	>50	>50	>50	>50	>50	>50	27.4	>50	>50	>1.0
**4j**	>50	>50	>50	>50	>50	>50	>50	>50	>50	>50	>50	>1.0
**4k**	>50	>50	>50	>50	>50	>50	>50	>50	>50	>50	>50	>1.0
**4l**	>50	32.1	>50	>50	>50	>50	>50	>50	>50	>50	>50	>1.0
**4m**	>50	25.8	>50	>50	>50	>50	>50	26.1	>50	>50	>50	>1.9
**4n**	29.5	23.9	22.5	16.8	30.6	30.6	24.5	18	24.1	42.2	33.2	2.1
**10a**	>50	>50	>50	>50	>50	>50	>50	>50	>50	>50	>50	>1.0
**10b**	>50	>50	>50	>50	>50	>50	>50	>50	>50	>50	>50	>1.0
**10c**	>50	>50	>50	>50	>50	>50	>50	>50	>50	>50	>50	>1.0
**10d**	>50	>50	>50	>50	>50	>50	>50	>50	>50	>50	>50	>1.0
**10e**	>50	>50	>50	>50	>50	>50	>50	>50	>50	>50	>50	>1.0
**10g**	>50	>50	>50	>50	>50	>50	>50	>50	>50	>50	>50	>1.0
**10h**	>50	>50	>50	>50	>50	>50	>50	>50	>50	>50	>50	>1.0
**10i**	>50	>50	>50	>50	>50	>50	>50	>50	>50	>50	>50	>1.0
**10j**	>50	>50	>50	>50	>50	>50	>50	>50	>50	>50	>50	>1.0
**10k**	>50	>50	>50	>50	>50	>50	>50	>50	>50	>50	>50	>1.0
**10l**	>50	>50	>50	>50	>50	>50	>50	>50	>50	>50	>50	>1.0
**10m**	>50	>50	>50	>50	>50	>50	>50	>50	>50	>50	>50	>1.0
**10n**	>50	>50	>50	>50	>50	>50	>50	>50	>50	>50	>50	>1.0
**11a**	12.1	7.9	6.3	13.7	11.4	12.4	20.9	8.7	15.3	39.1	26.8	3.8
**11b**	12.1	5.7	9.6	15.8	19.9	13.5	26.3	6	21.8	36.6	23.9	5
**11g**	34.8	11.1	>50	20	33.5	39.6	44.4	18.1	20.5	>50	>50	>2.8
**11h**	21.3	10.8	>50	10.8	25.1	30.6	28.4	5.2	17	>50	>50	>9.5
**11i**	>50	17.5	>50	25	34.6	41	>50	19.7	41.8	>50	>50	>2.5
**11j**	13.5	7	7.5	16.5	22.1	20.9	21.9	4.9	24	>50	27.8	>7.9
**11l**	13.9	8.3	>50	8.7	18.5	30.8	24.8	7.2	17	>50	>50	>7.0
**11n**	15.1	8.3	22.3	12.1	14.9	16.8	20.5	4.9	21.9	35.1	26.5	6.3
**5**	9.4	9.6	4.3	14.1	16	16	21	8.1	14	24	28	3.2
**GANT-61**	NA	>50	NA	NA	NA	NA	NA	NA	NA	NA	NA	NA

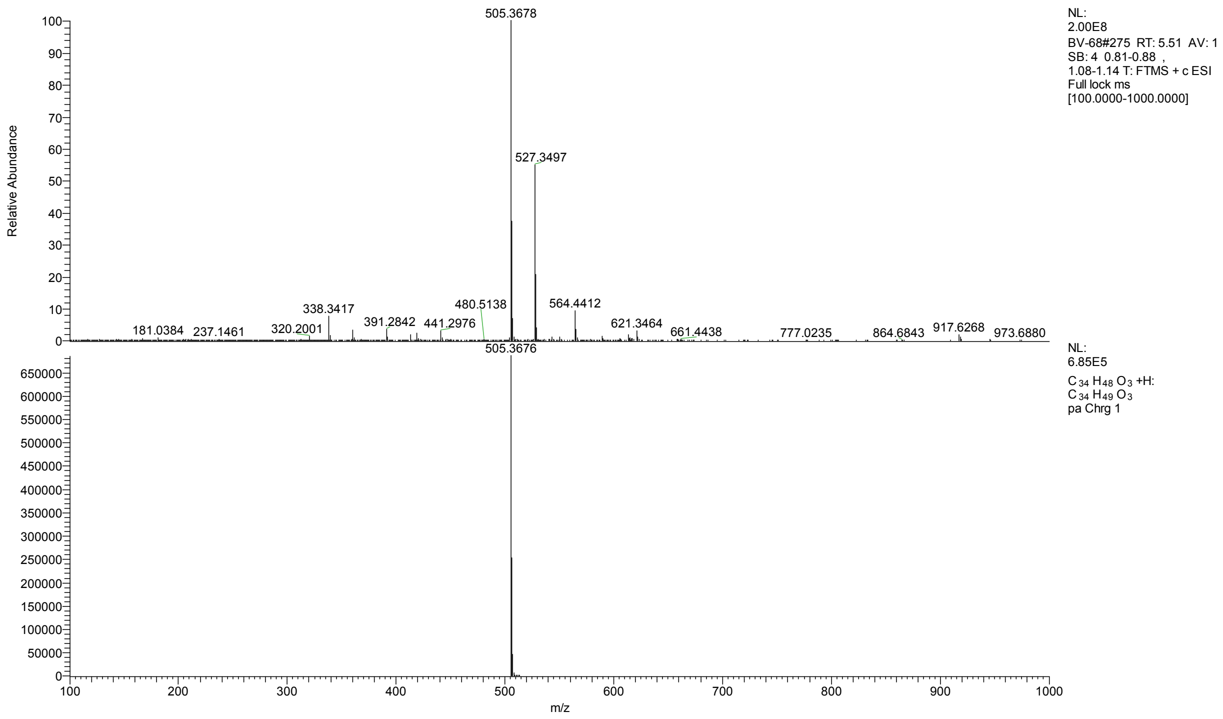

Figure S69. HRMS spectrum of the compound **4a**.

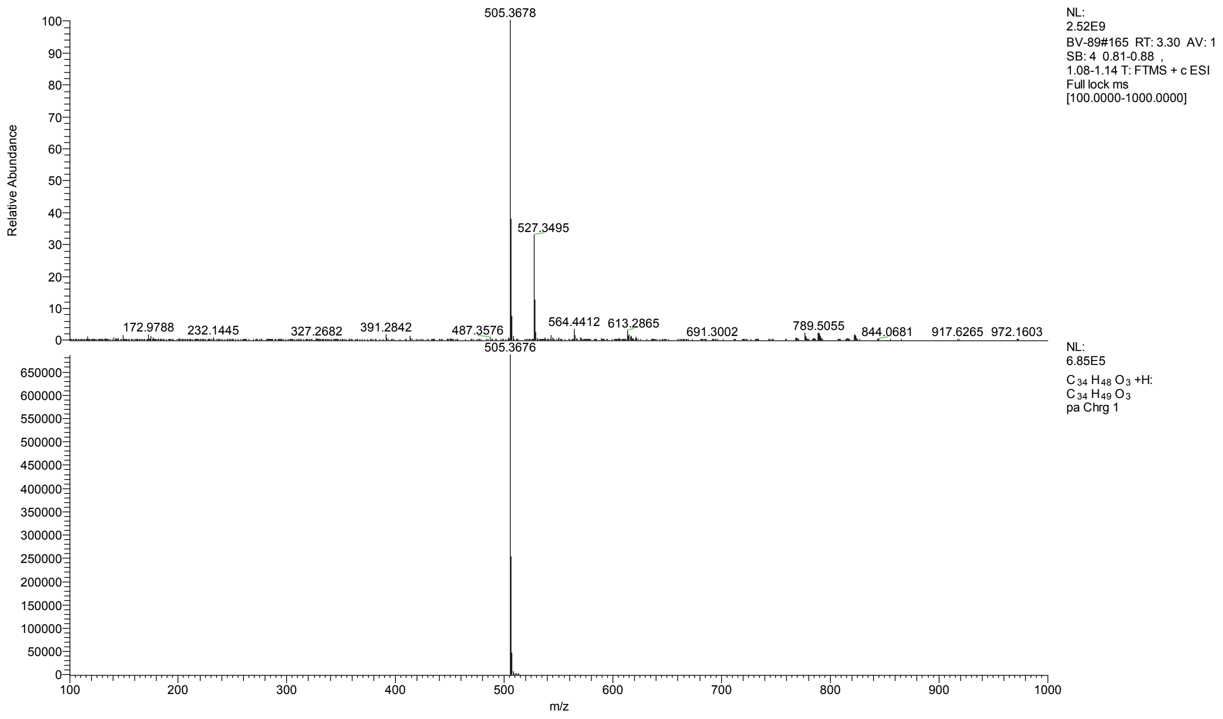

Figure S70. HRMS spectrum of the compound **4b**.

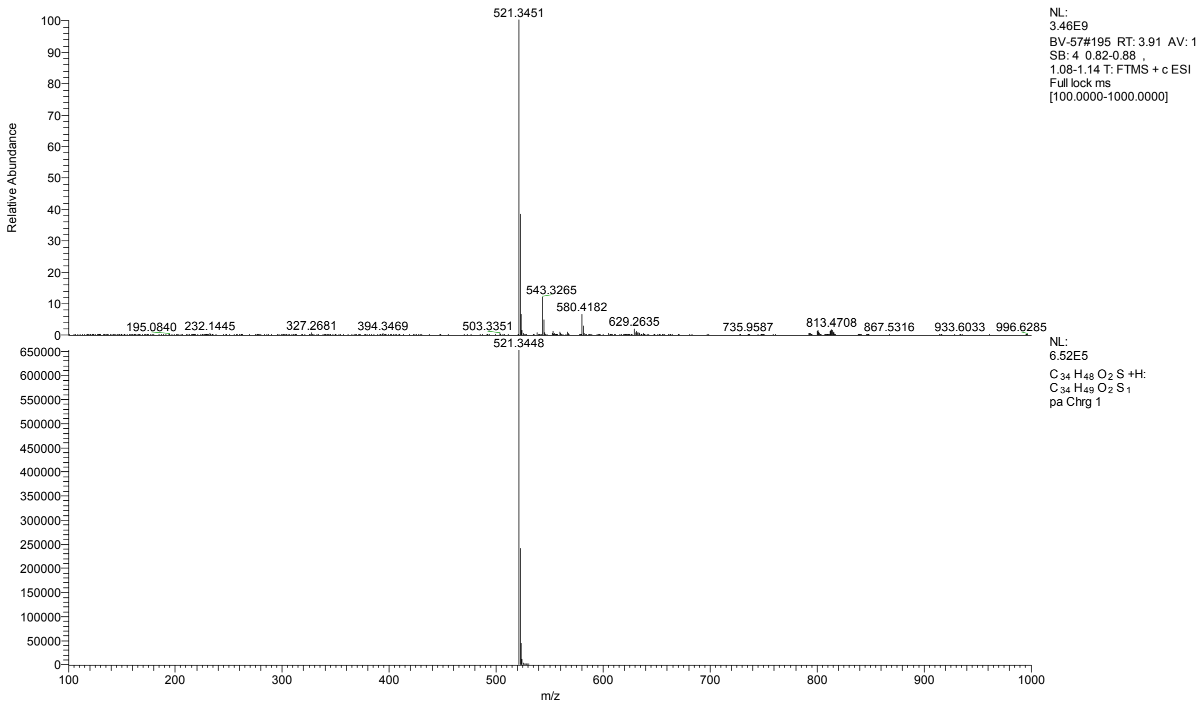

Figure S71. HRMS spectrum of the compound **4c**.

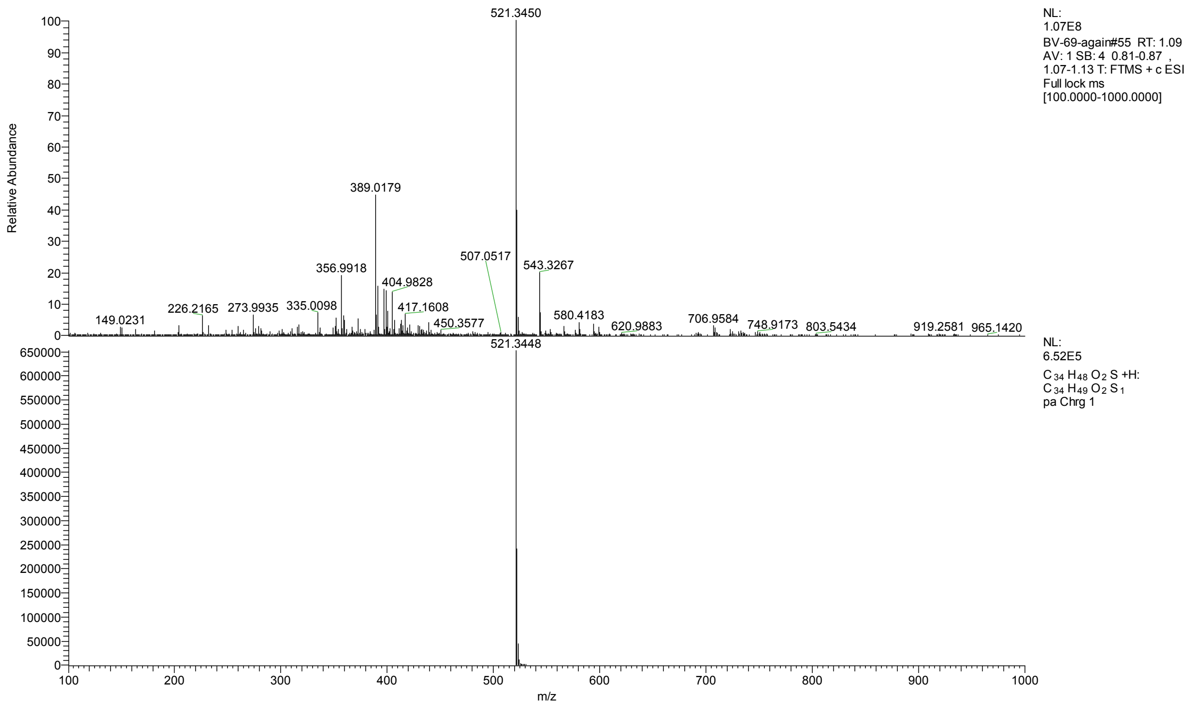

Figure S72. HRMS spectrum of the compound **4d**.

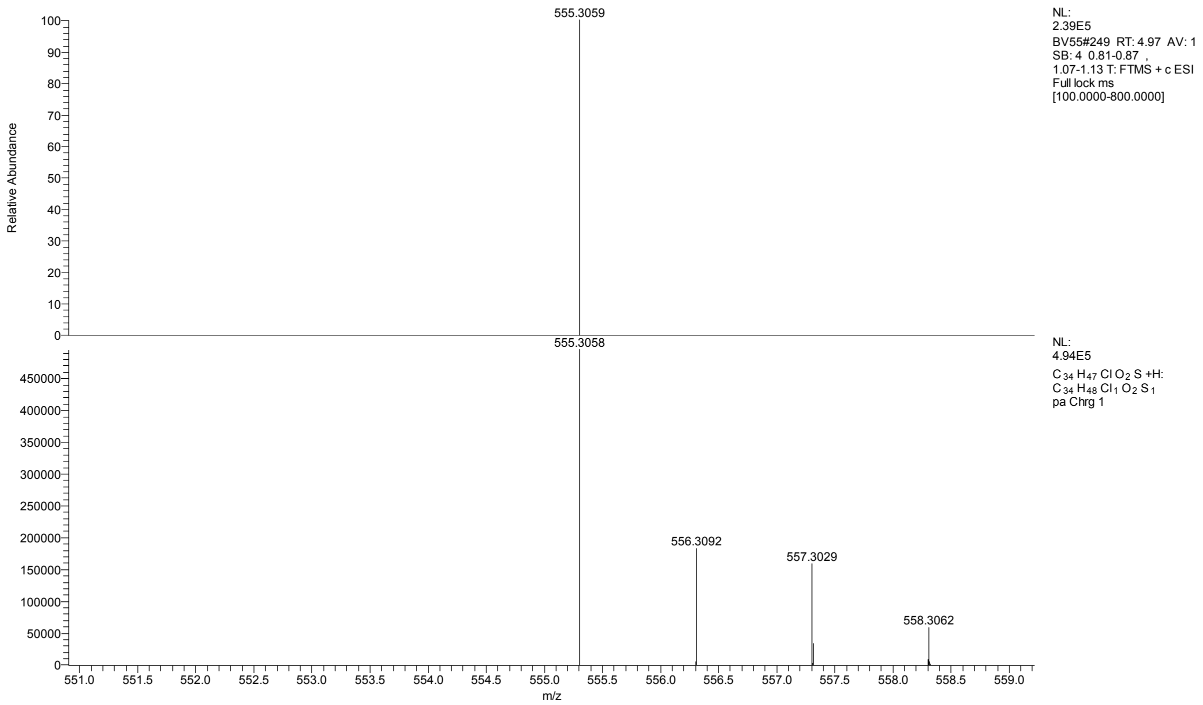

Figure S73. HRMS spectrum of the compound **4e**.

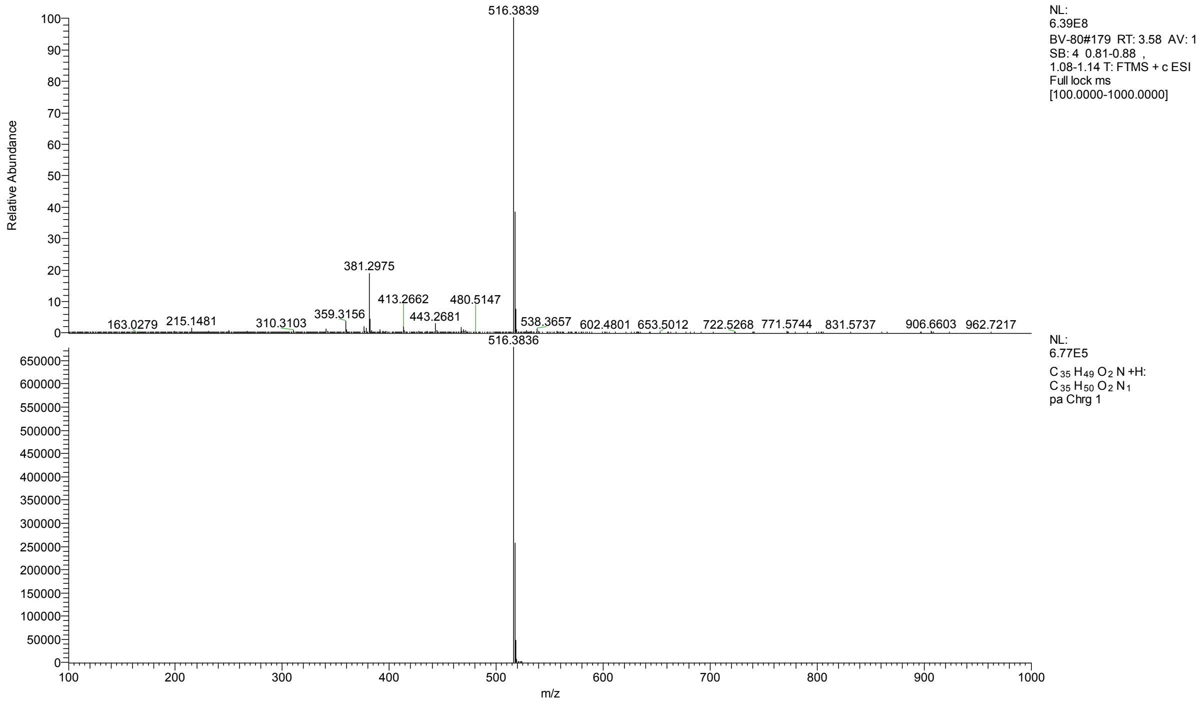

Figure S74. HRMS spectrum of the compound **4g**.

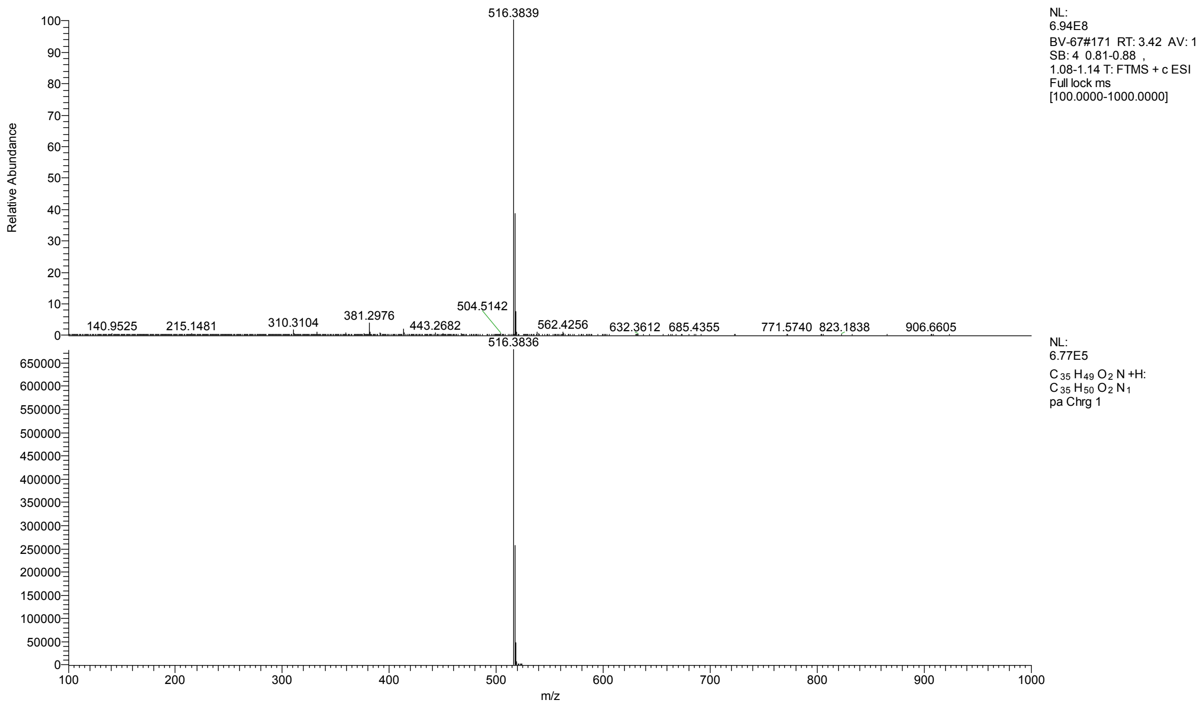

Figure S75. HRMS spectrum of the compound **4h**.

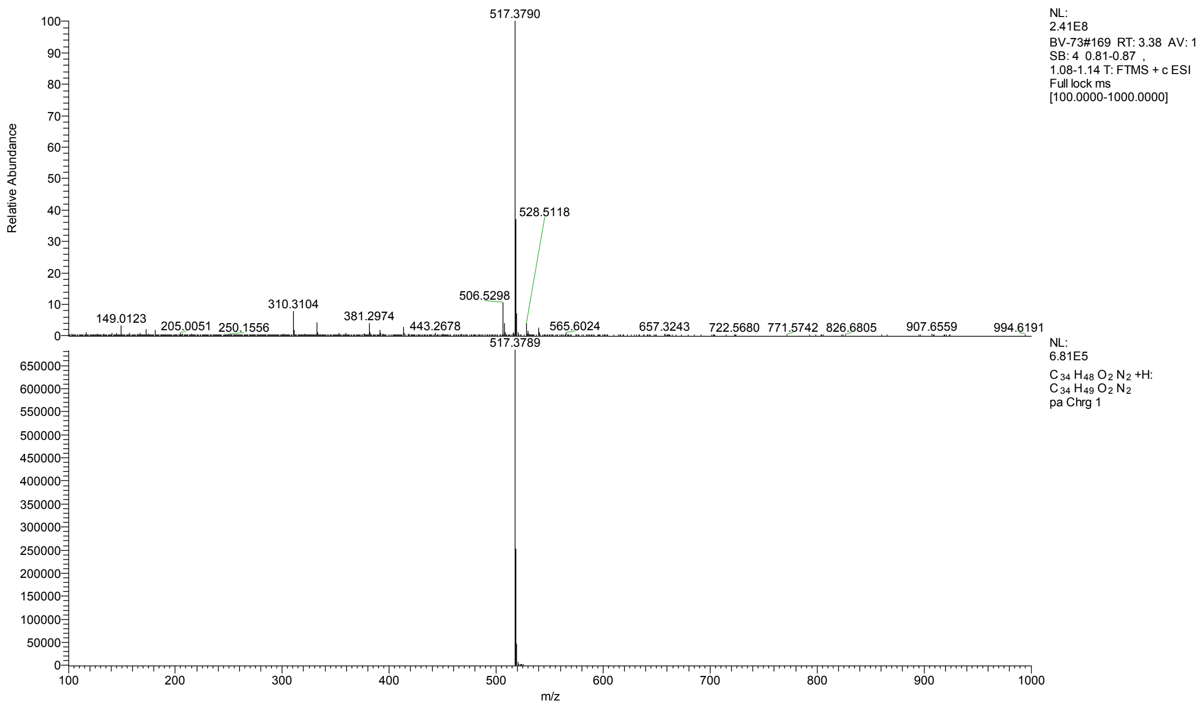

Figure S76. HRMS spectrum of the compound **4i**.

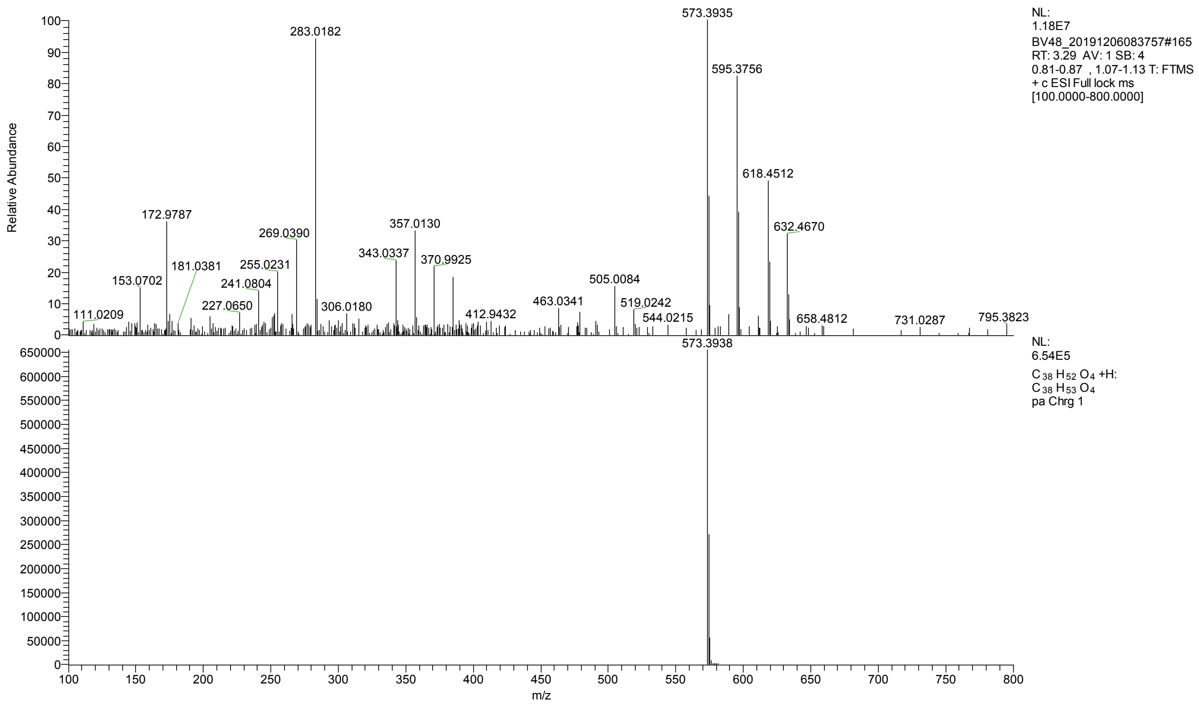

Figure S77. HRMS spectrum of the compound **4j**.

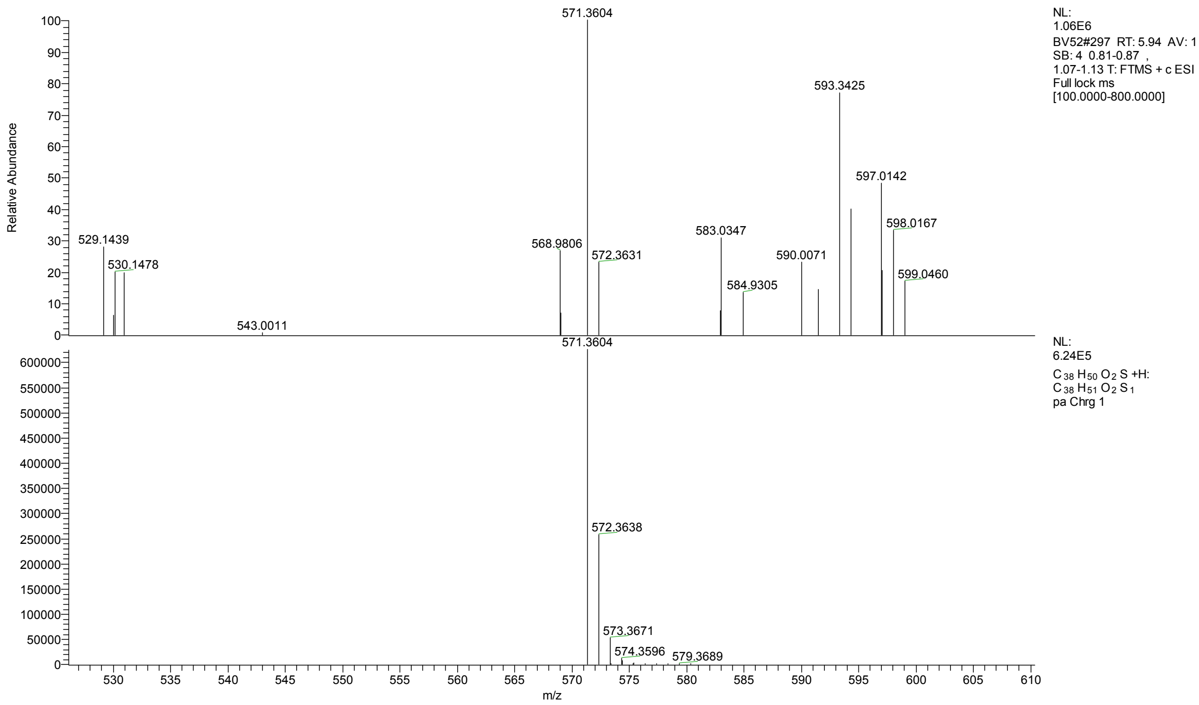

Figure S78. HRMS spectrum of the compound **4k**.

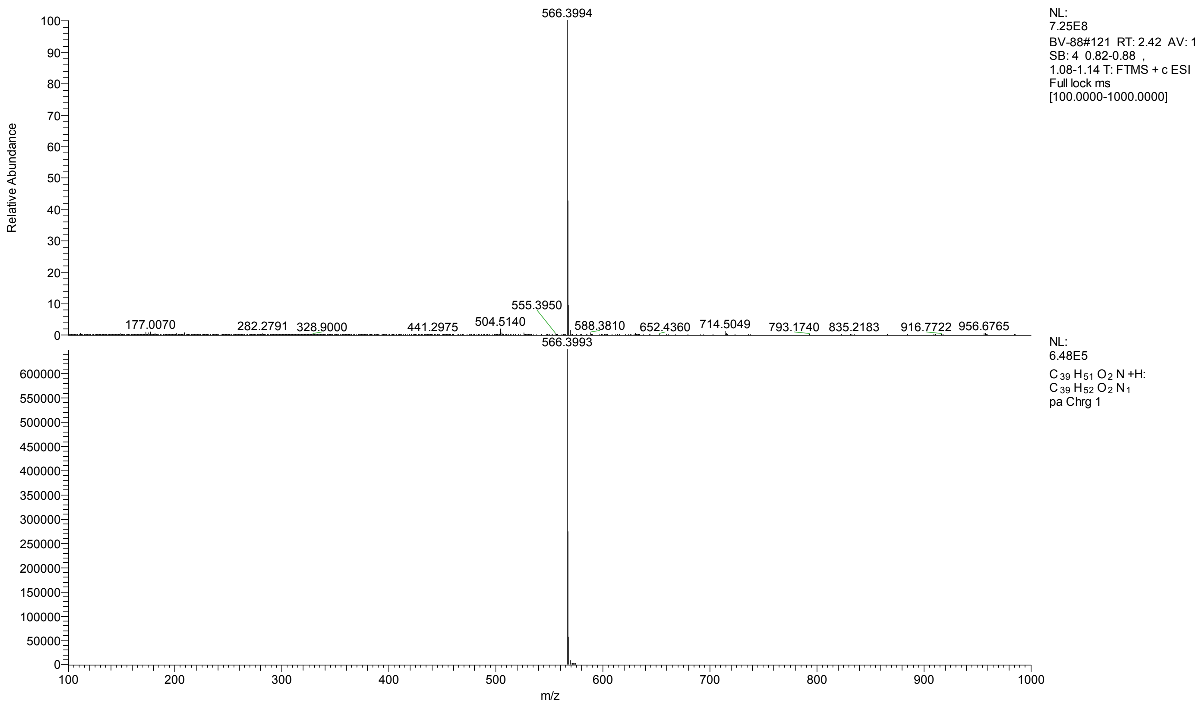

Figure S79. HRMS spectrum of the compound **4l**.

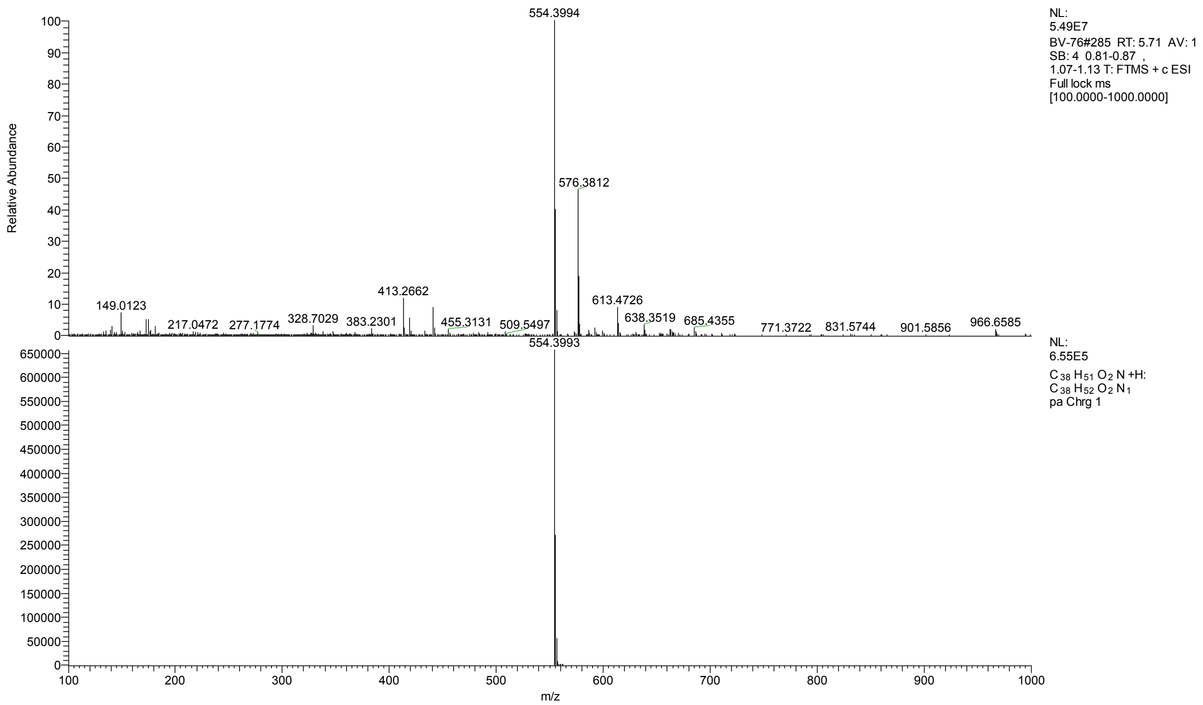

Figure S80. HRMS spectrum of the compound **4m**.

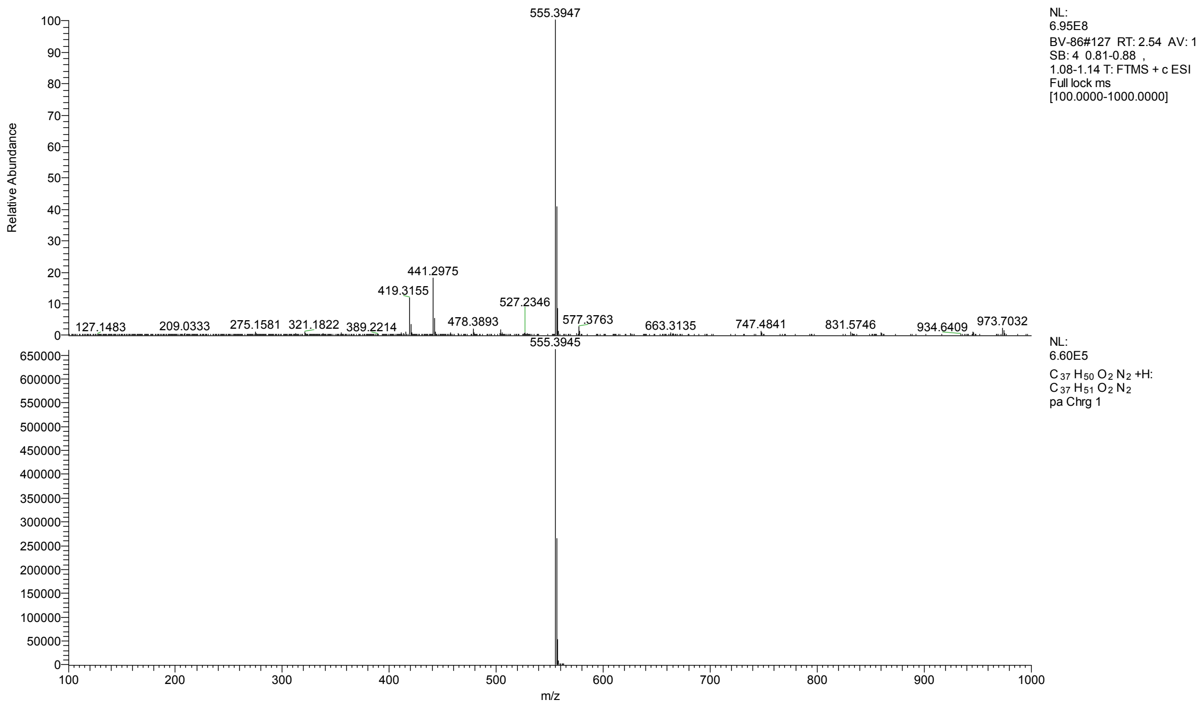

Figure S81. HRMS spectrum of the compound **4n**.

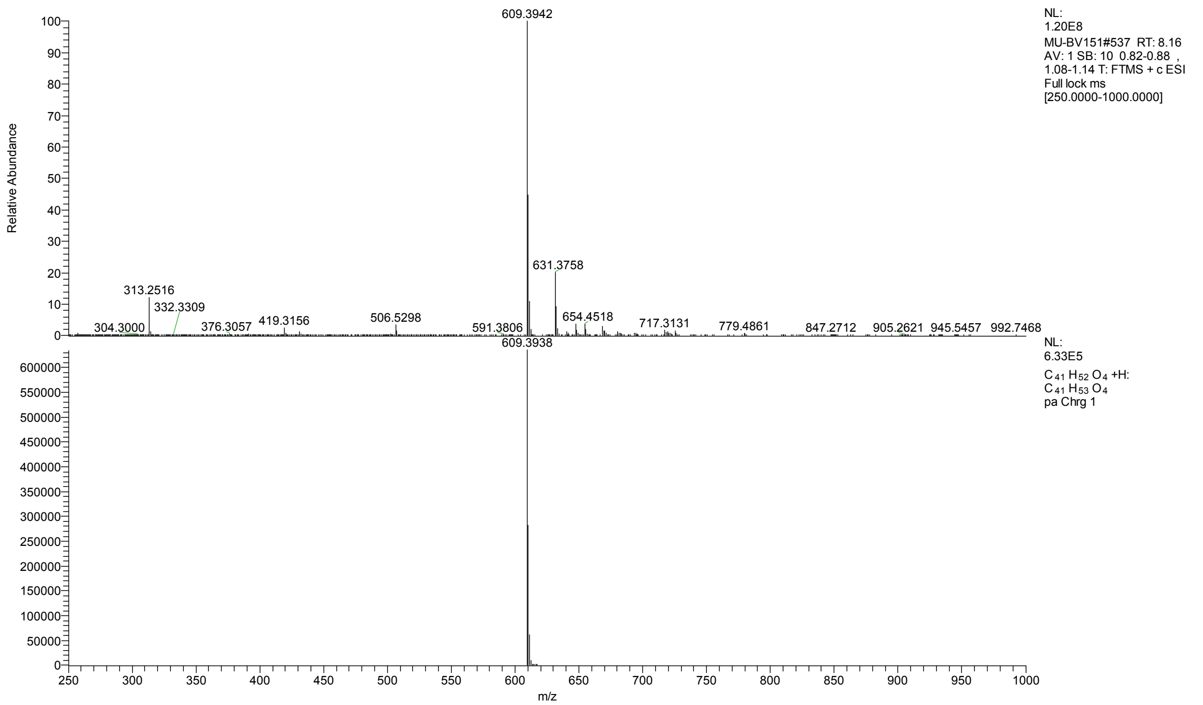

Figure S82. HRMS spectrum of the compound **10a**.

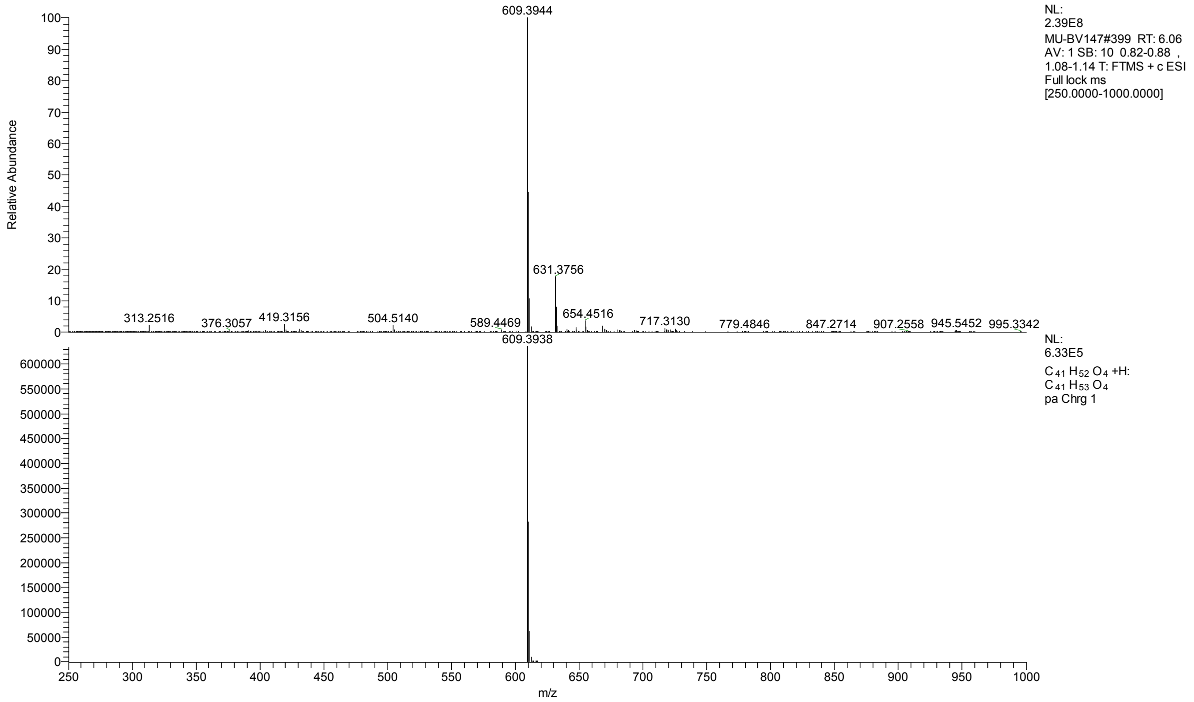

Figure S83. HRMS spectrum of the compound **10b**.

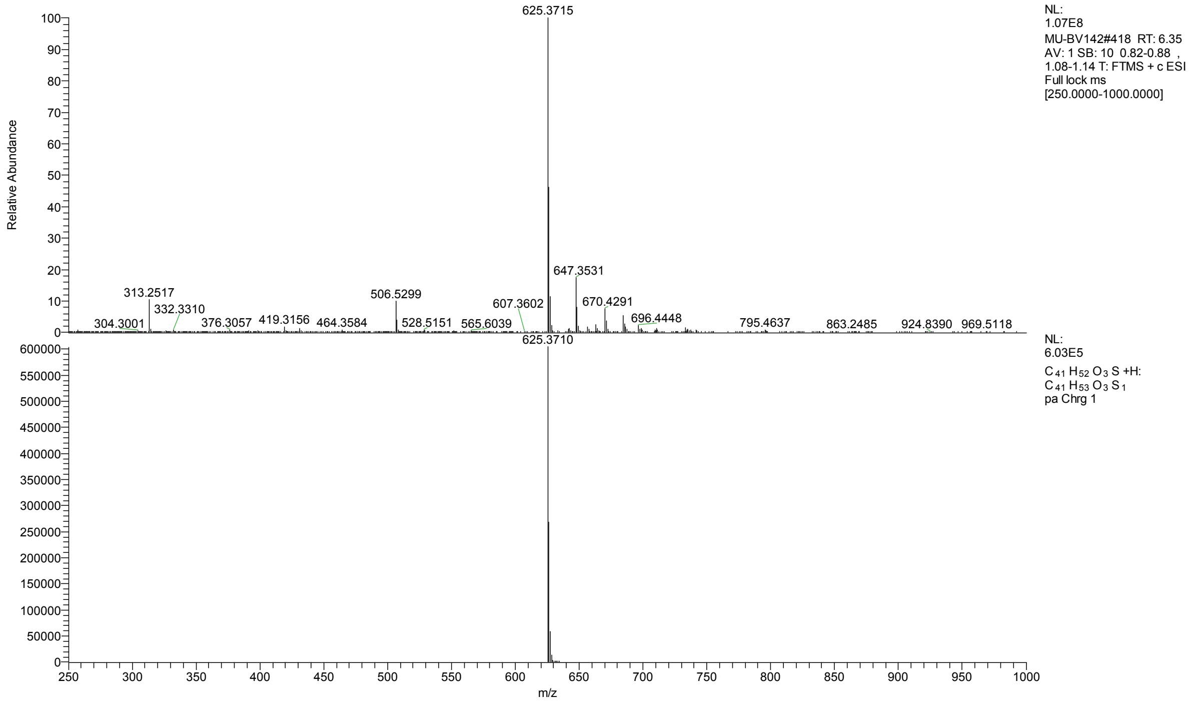

Figure S84. HRMS spectrum of the compound **10c**.

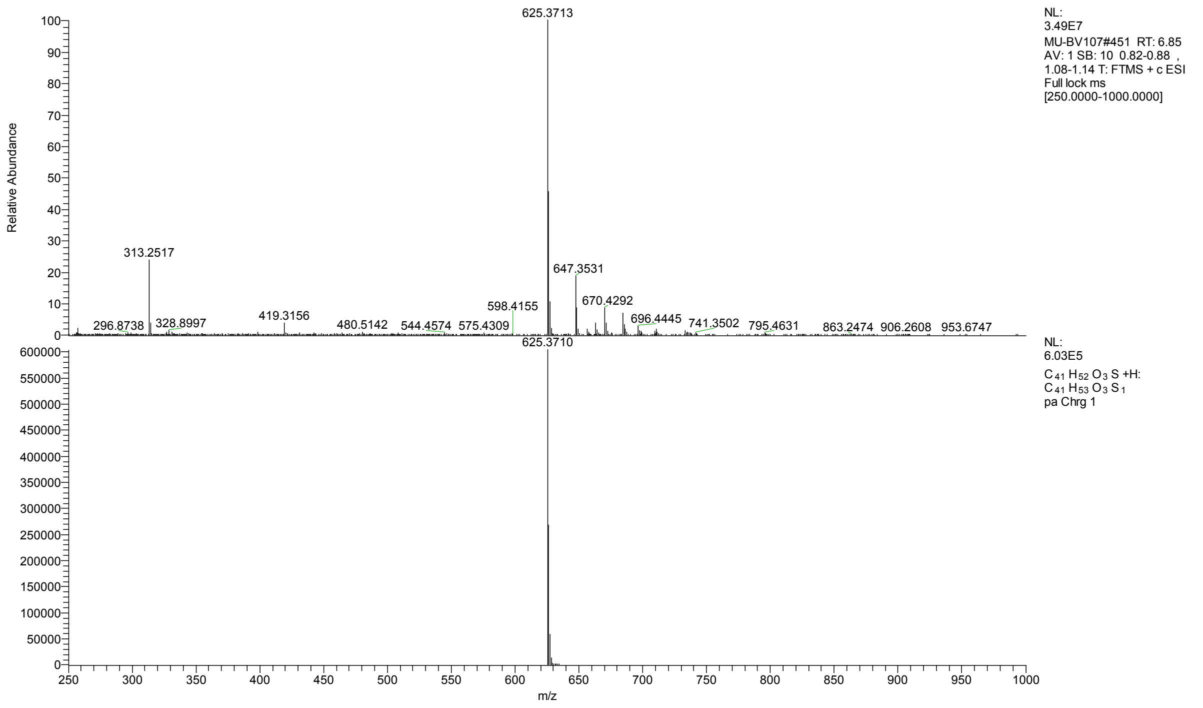

Figure S85. HRMS spectrum of the compound **10d**.

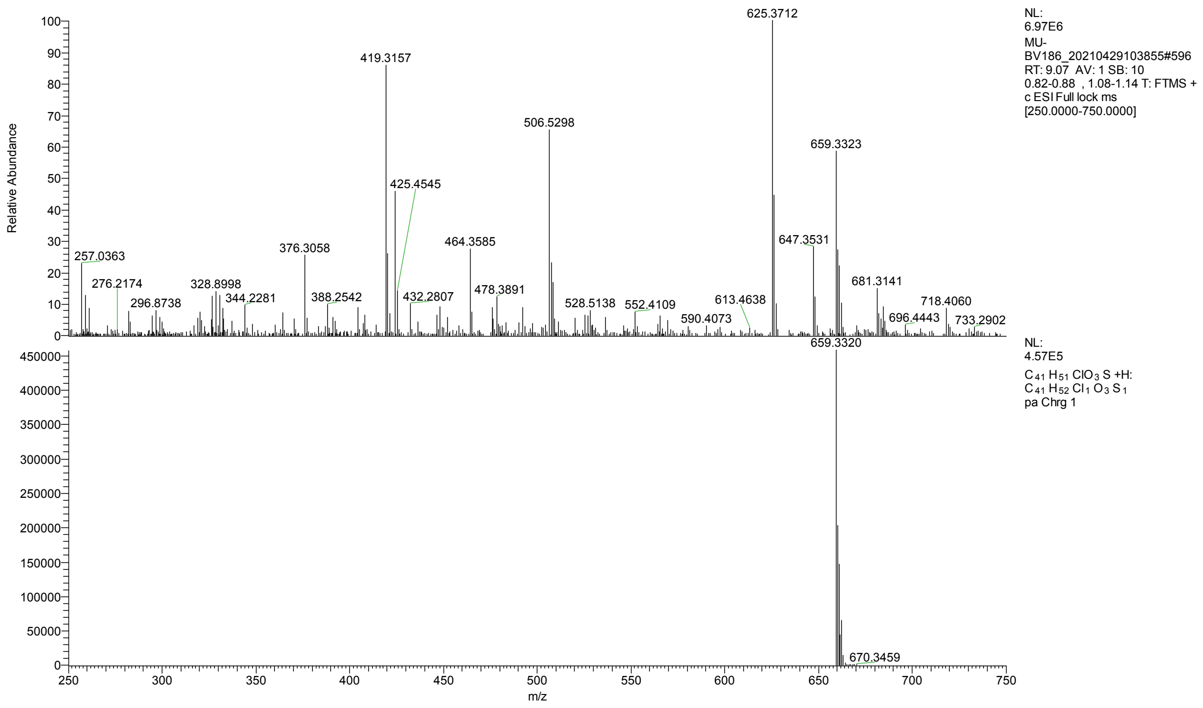

Figure S86. HRMS spectrum of the compound **10e**.

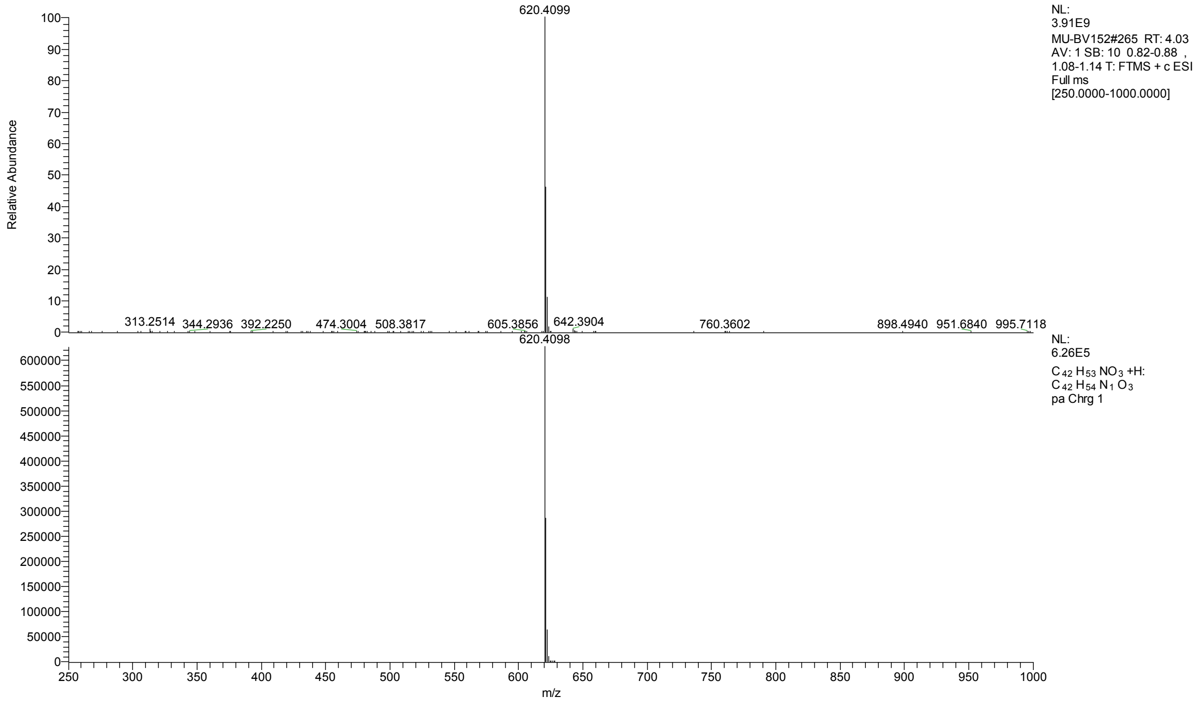

Figure S87. HRMS spectrum of the compound **10g**.

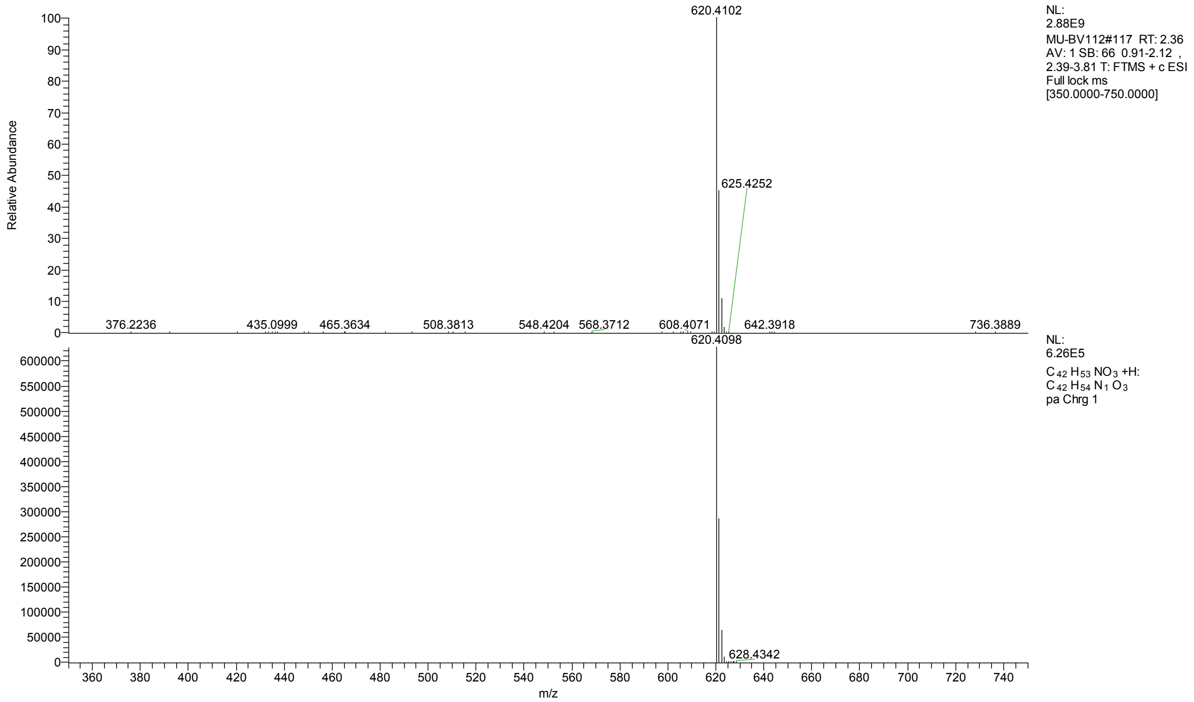

Figure S88. HRMS spectrum of the compound **10h**.

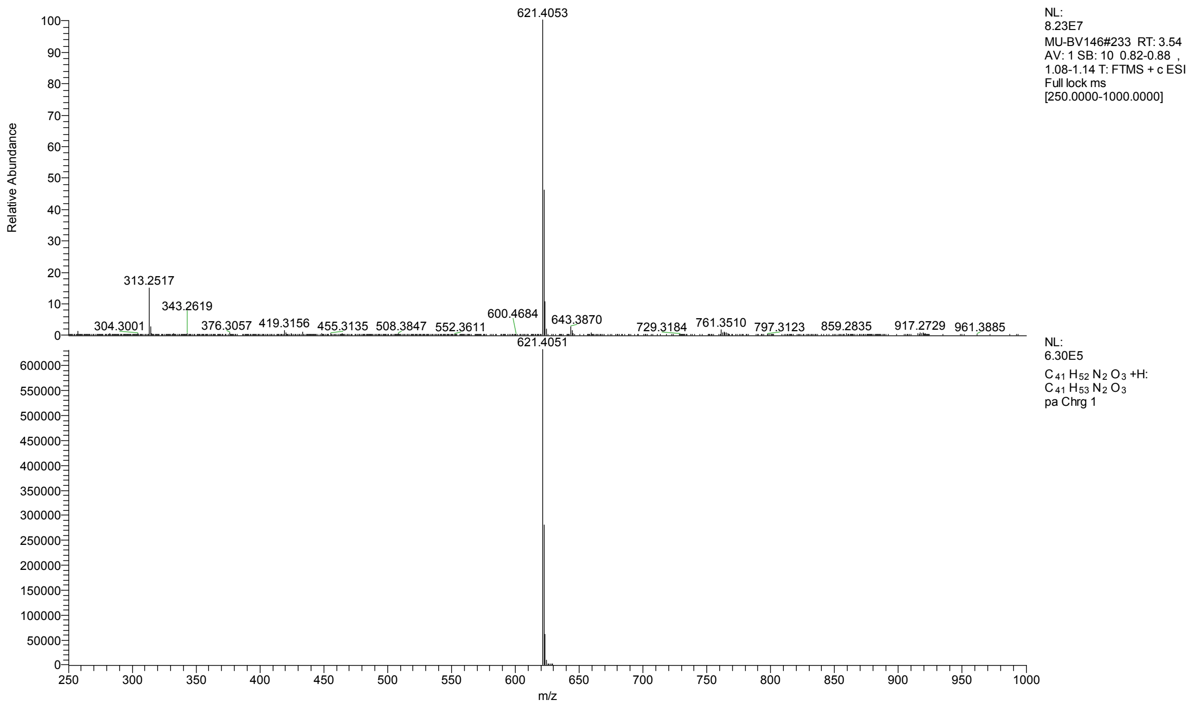

Figure S89. HRMS spectrum of the compound **10i**.

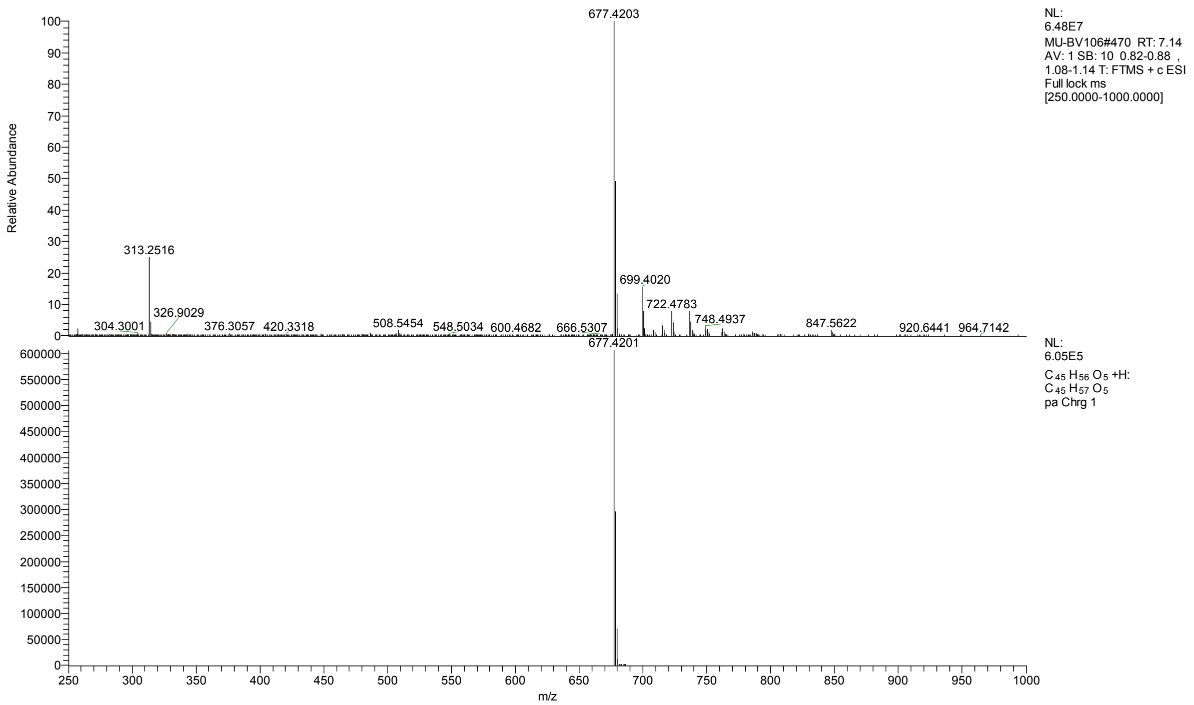

Figure S90. HRMS spectrum of the compound **10j**.

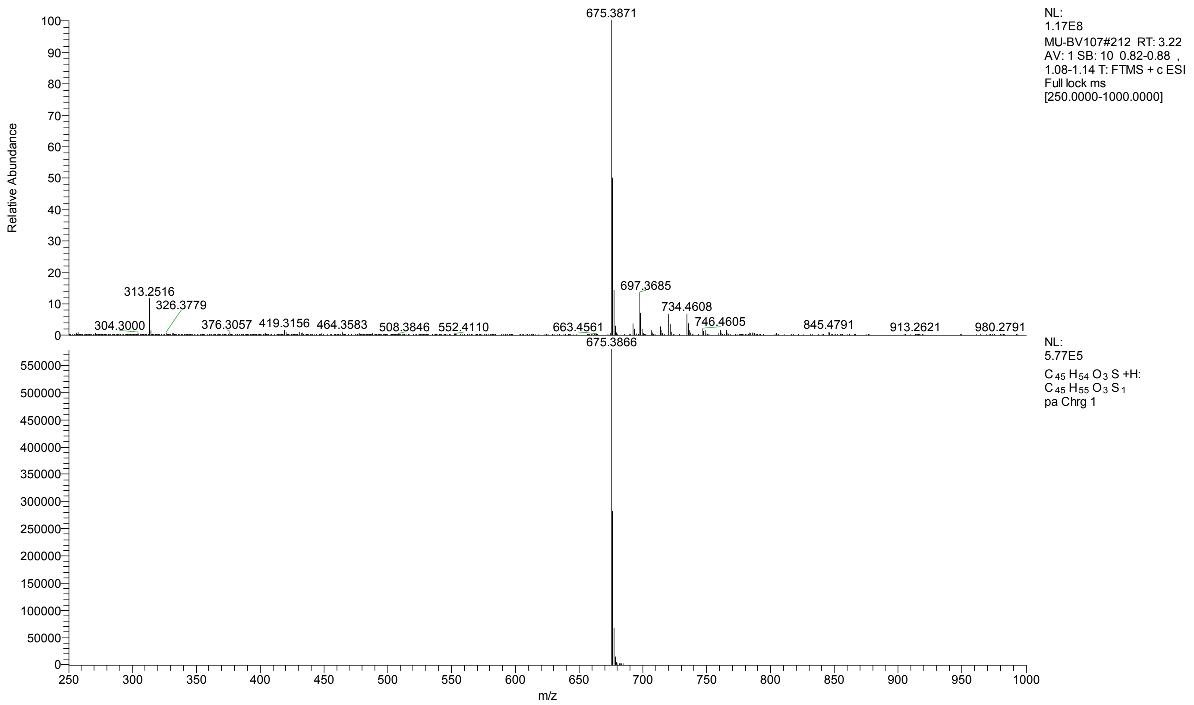

Figure S91. HRMS spectrum of the compound **10k**.

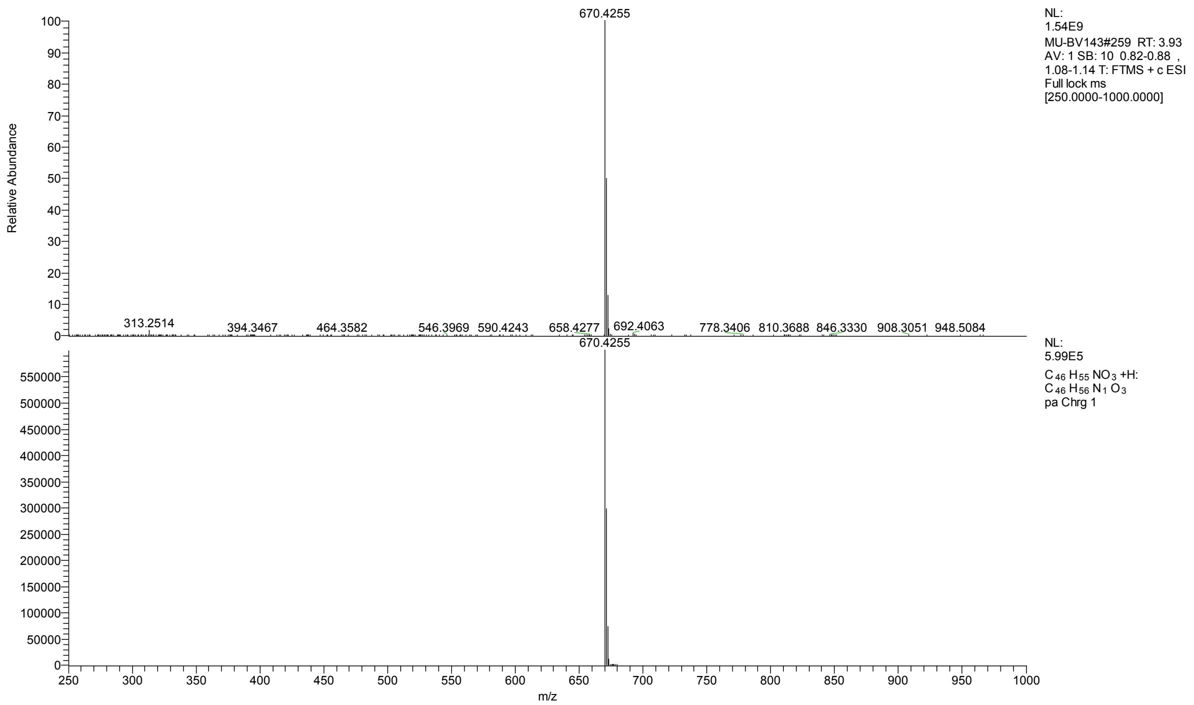

Figure S92. HRMS spectrum of the compound **10l**.

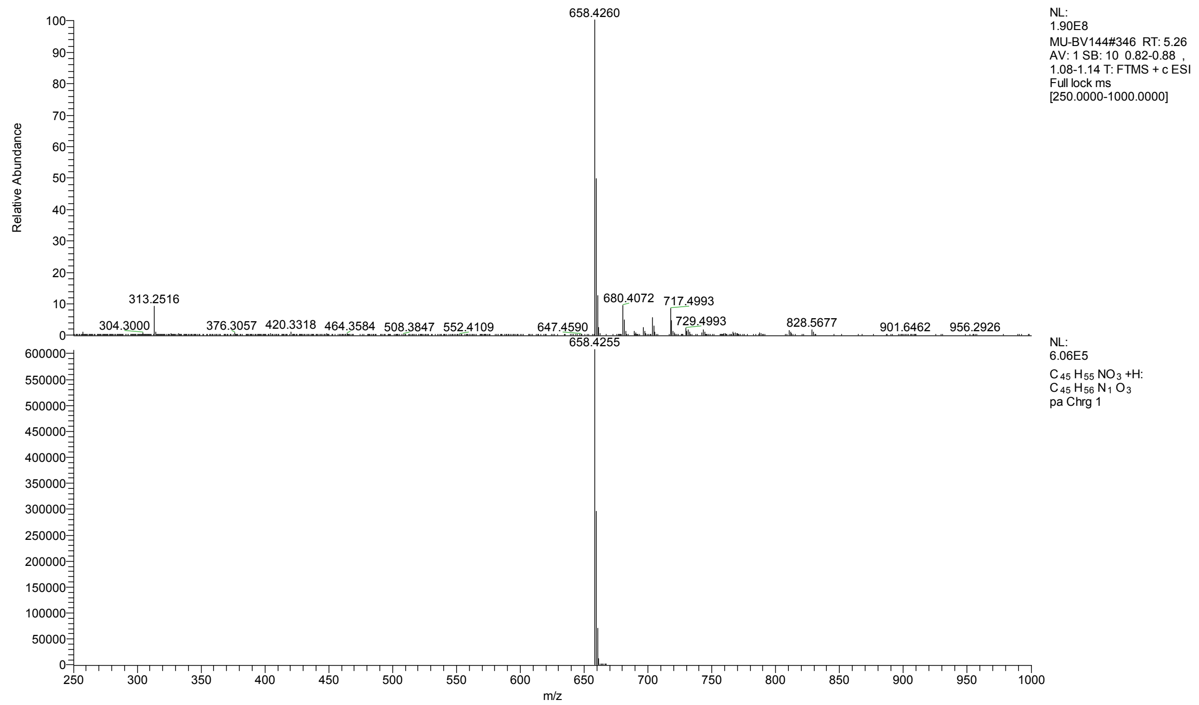

Figure S93. HRMS spectrum of the compound **10m**.

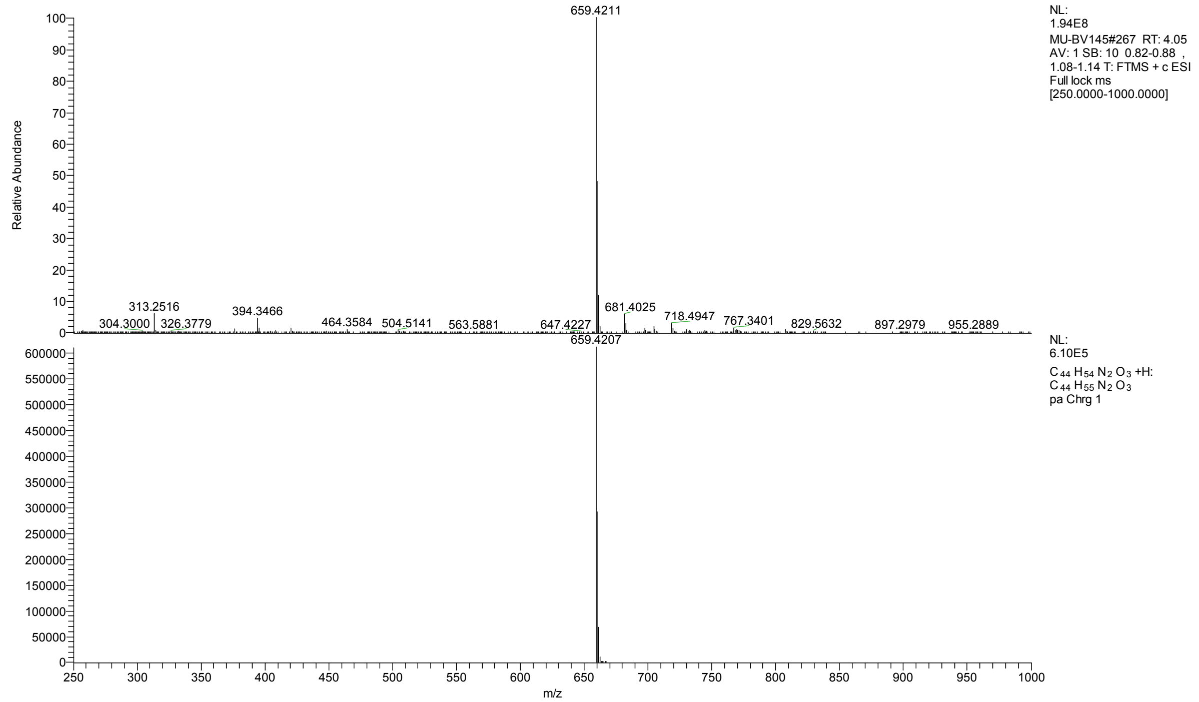

Figure S94. HRMS spectrum of the compound **10n**.

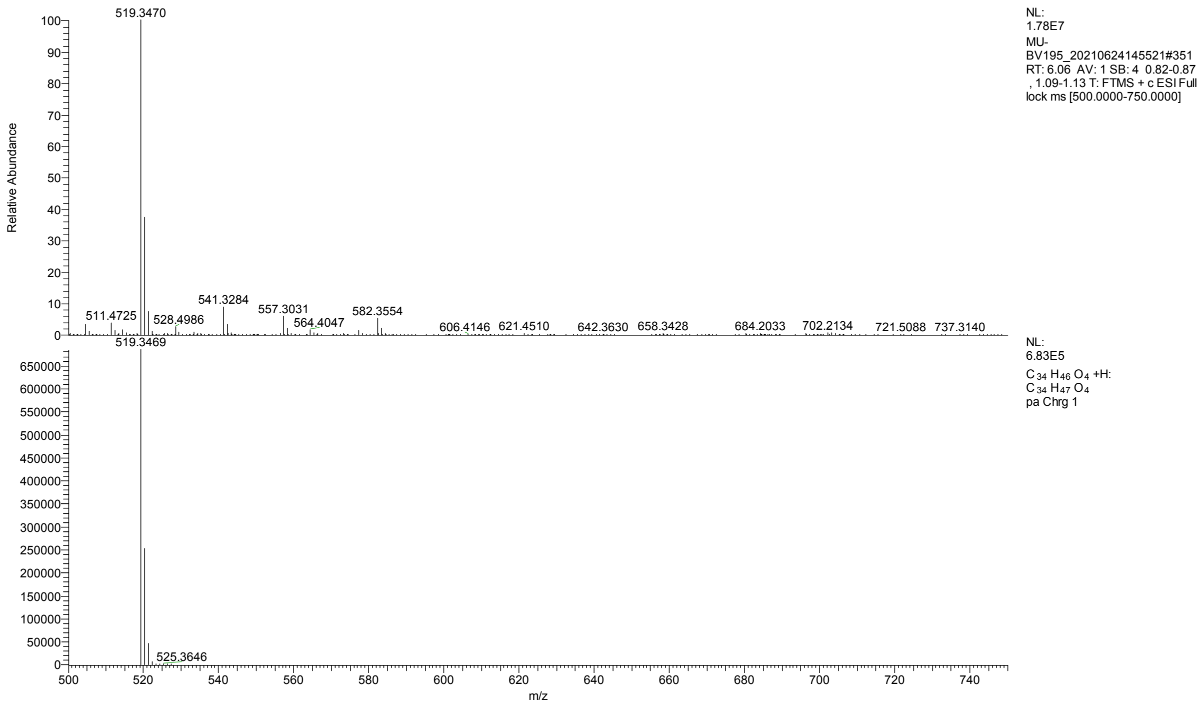

Figure S95. HRMS spectrum of the compound **11a**.

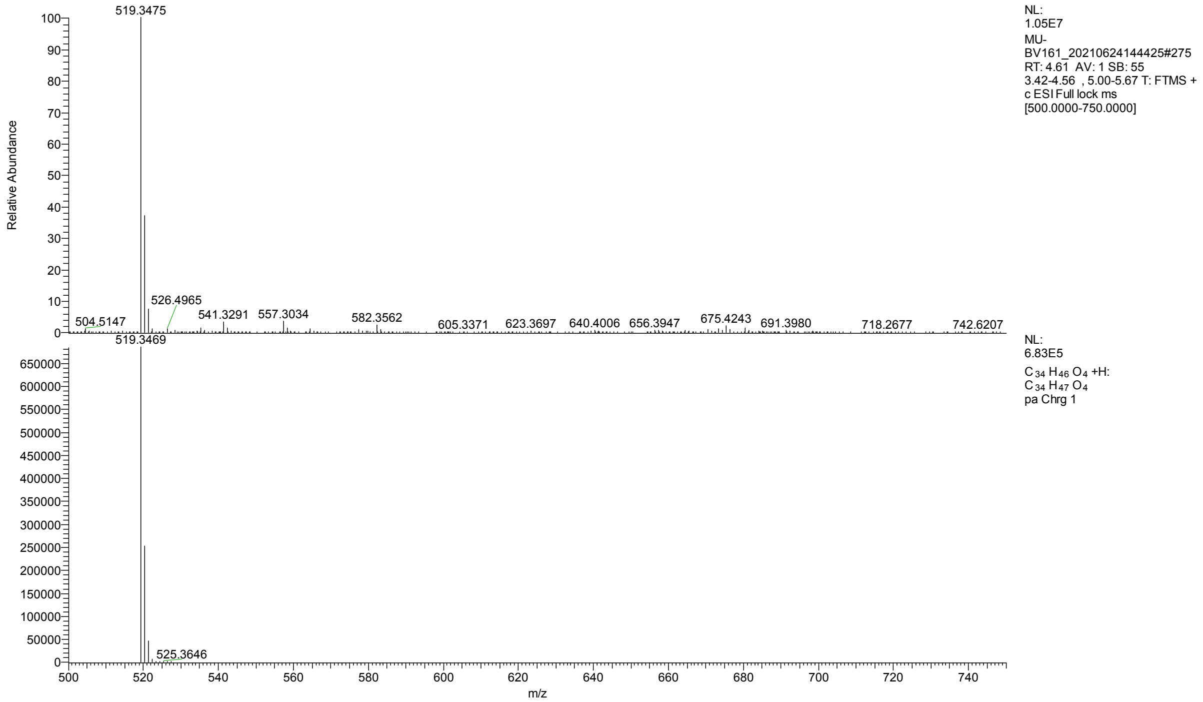

Figure S96. HRMS spectrum of the compound **11b**.

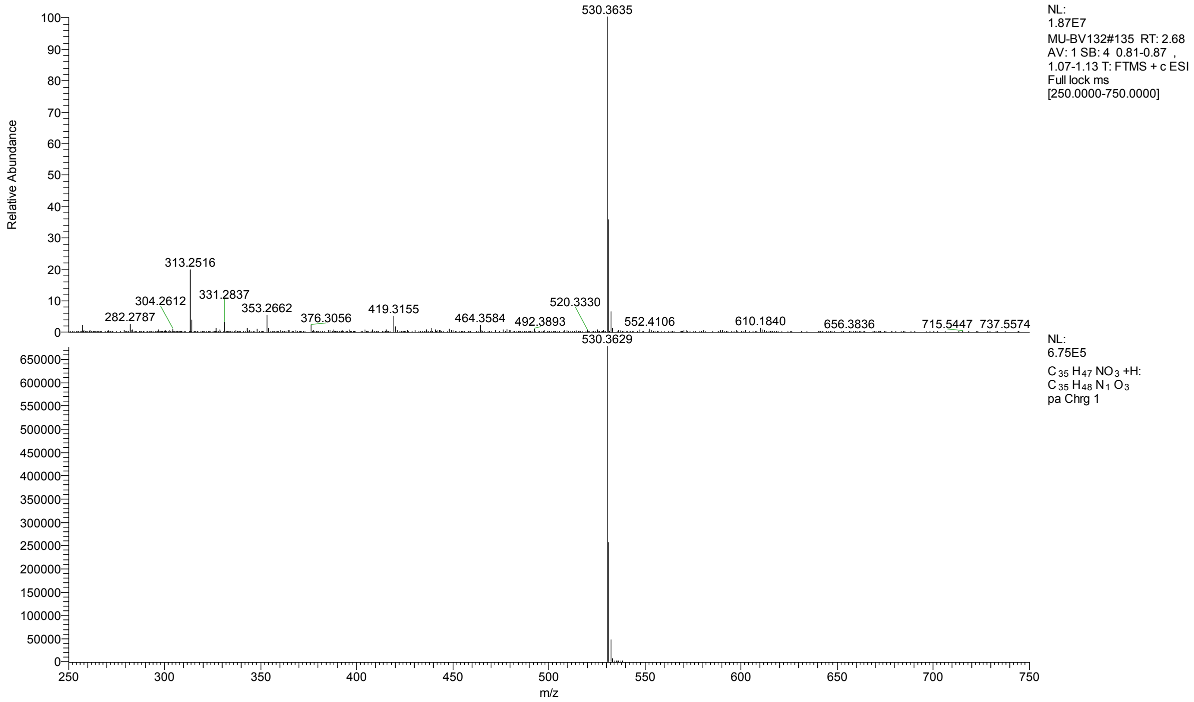

Figure S97. HRMS spectrum of the compound **11g**.

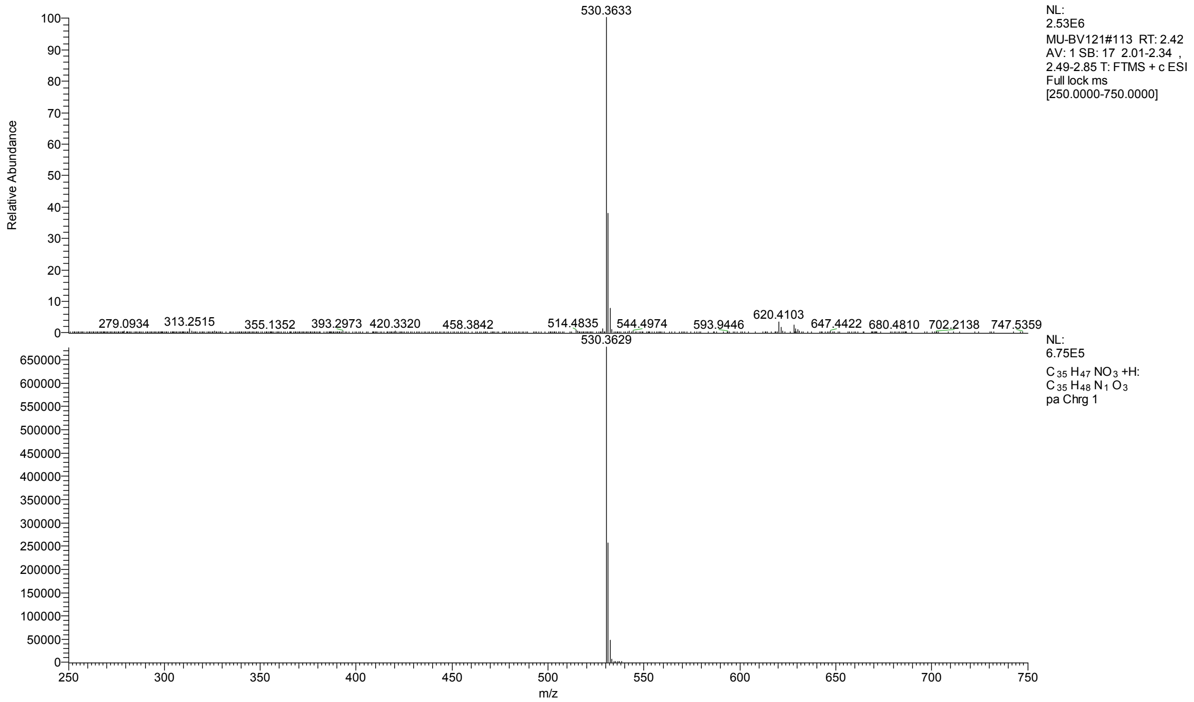

Figure S98. HRMS spectrum of the compound **11h**.

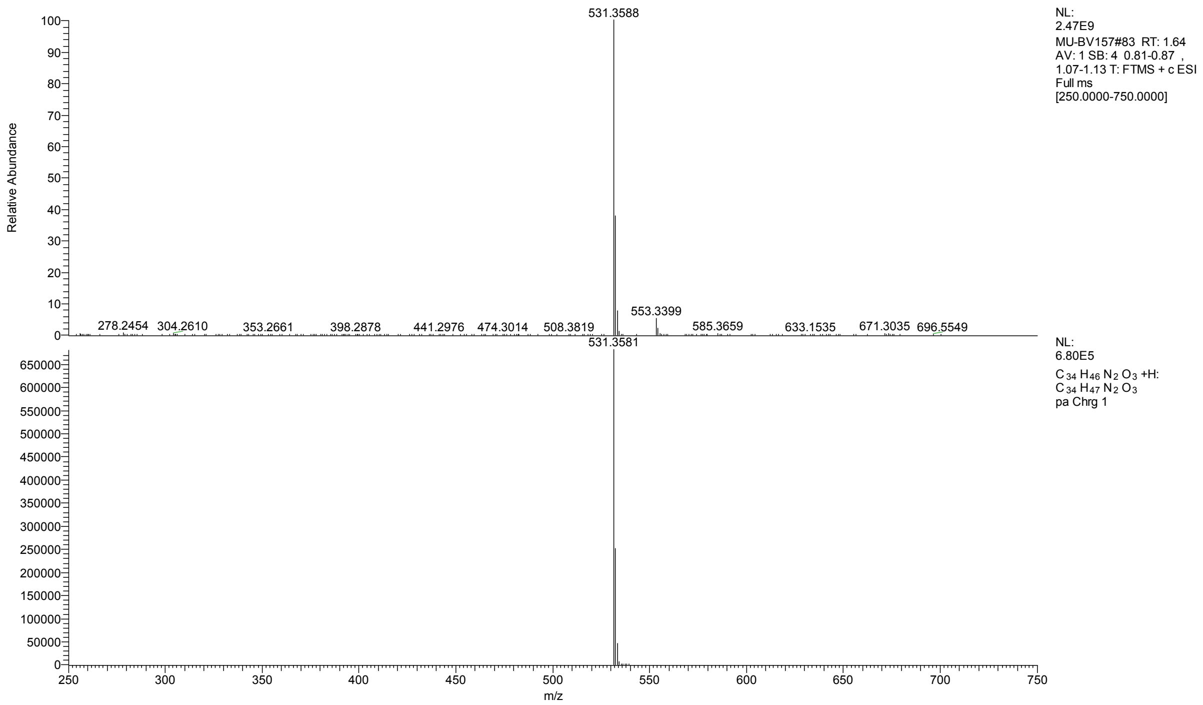

Figure S99. HRMS spectrum of the compound **11i**.

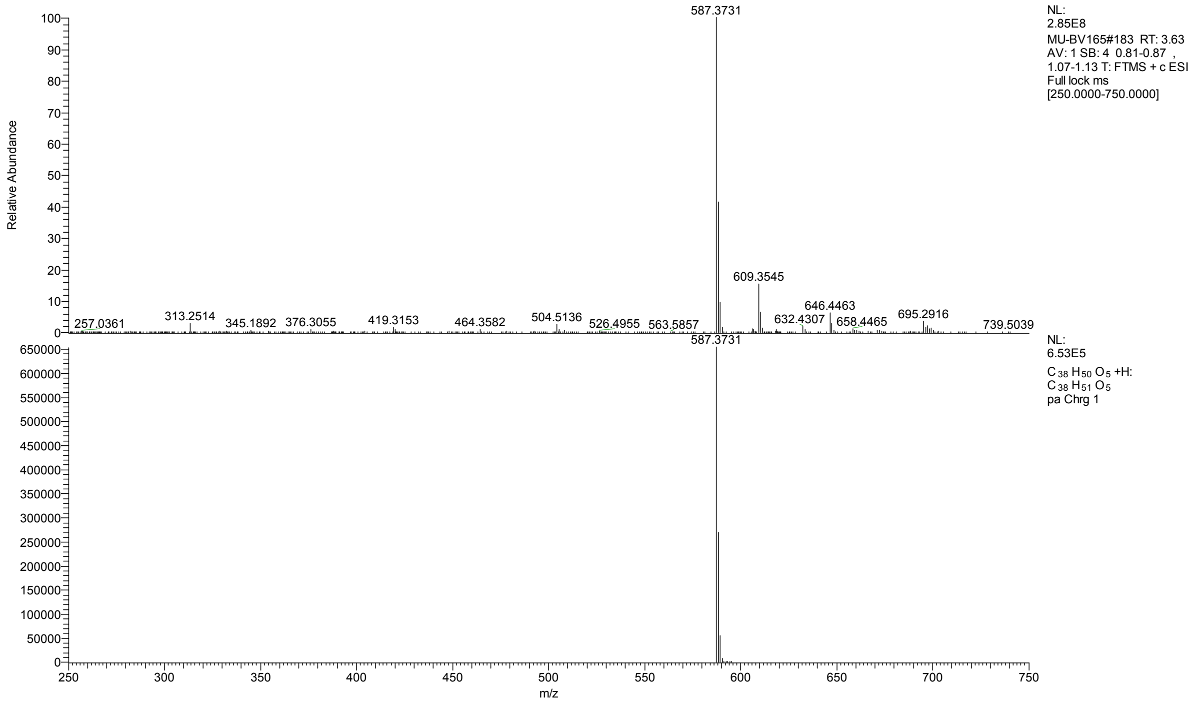

Figure S100. HRMS spectrum of the compound **11j**.

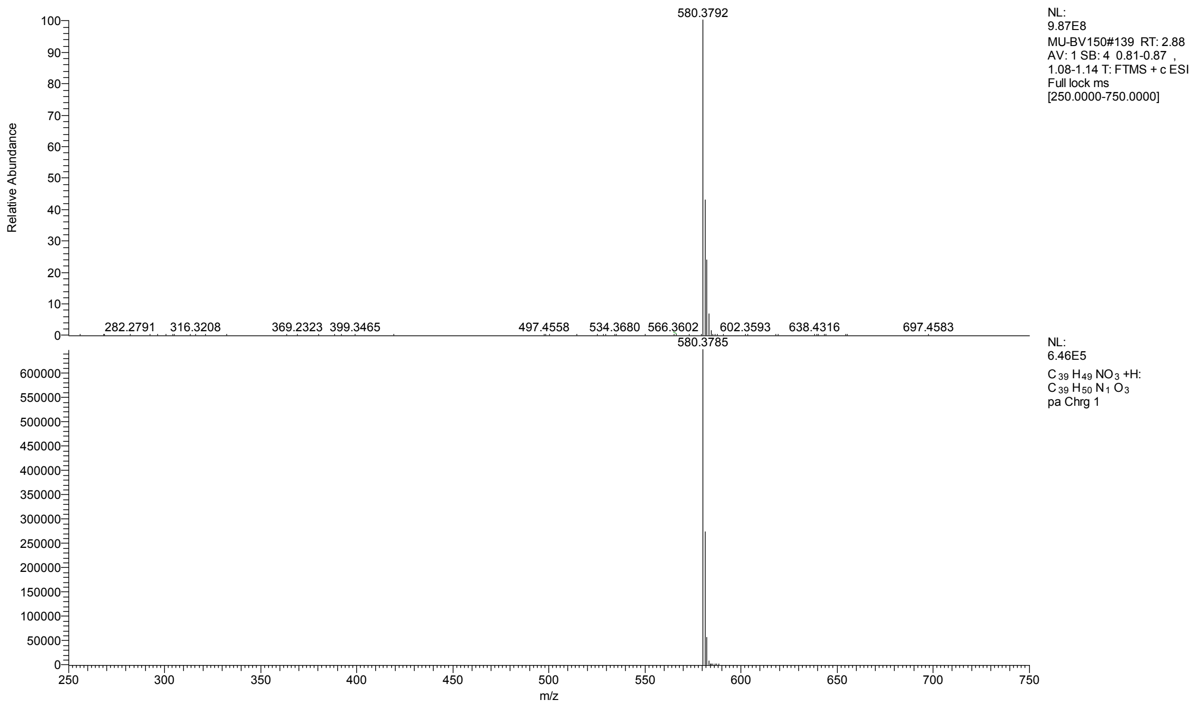

Figure S101. HRMS spectrum of the compound **11l**.

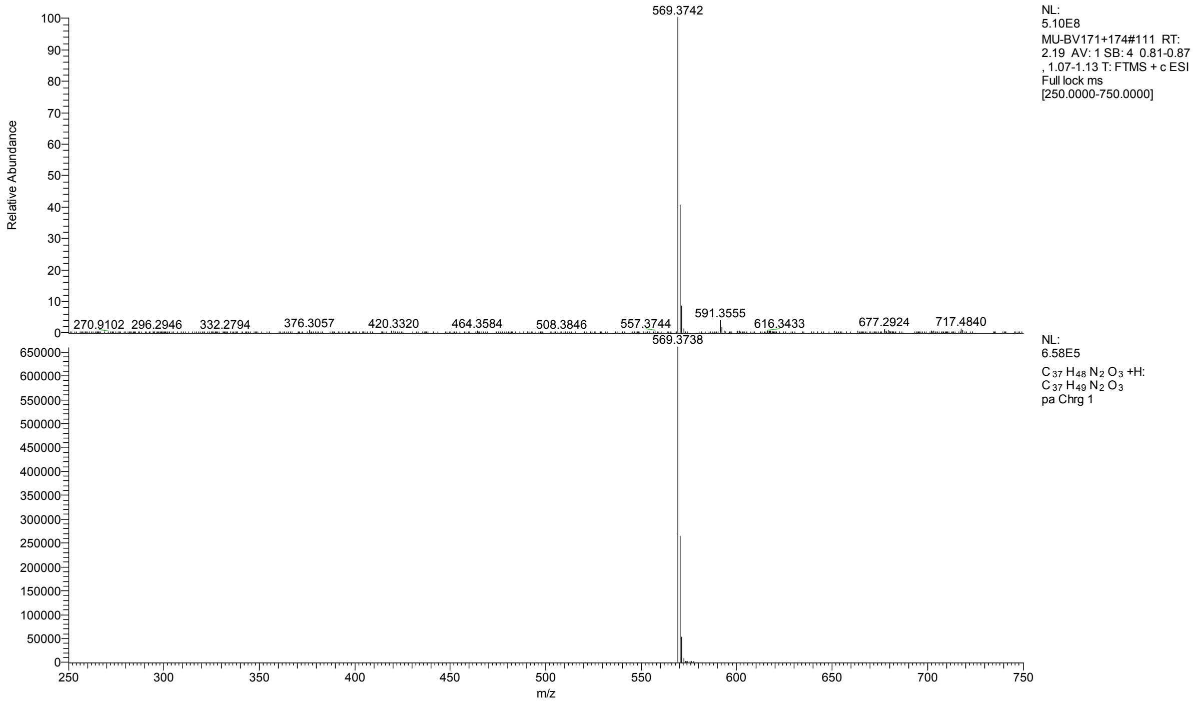

Figure S102. HRMS spectrum of the compound **11n**.
